# Lesser-known types of violence: Helping nurses and midwives to signal and act

**DOI:** 10.1016/j.ijnsa.2022.100098

**Published:** 2022-09-17

**Authors:** Roderik F. Viergever, Peter Griffiths

**Affiliations:** a Coördinatiecentrum tegen Mensenhandel (CoMensha), Dutch National Coordinating Centre against Human Trafficking, Amersfoort, The Netherlands; b Executive Editor International Journal of Nursing Studies, University of Southampton, Southampton, England, United Kingdom

Nurses, midwives and other health, education and legal professionals regularly meet people who are subjected to, or who commit, violence. Such meetings provide opportunities for identifying violence that is taking place and for taking appropriate steps to help people get out of a violent situation. Unfortunately, such violence is only identified by health professionals in a small minority of cases (Ali et al., 2016). There is a danger the violence is equated with physical violence and viewed in isolation as a single act. Rather, violence is something that typically emerges in the context of a complex social relationship as part of an ongoing pattern of behaviour. It is not limited to physical acts and can cause harm in many domains. Violence may present itself differently depending on the types and it occurs in the context of a society and relationships between people. These types, contexts and relationships can make it hard to understand the causes of resulting injury and to offer appropriate support to prevent further harm.

A non-exhaustive list of types and contexts of violence could include intimate (ex-)partner violence, elder abuse, parent abuse (children who abuse their parents), male abuse (violence that targets men), honour-based violence (violence that is committed to protect or defend the honour of an individual, family or community, such as forced marriages, forced abandonment or forced isolation), online sexual intimidation (various forms of sexual intimidation, such as shame-sexting, grooming, revenge porn and sextortion), stalking, human trafficking, female genital mutilation, and child abuse, including various specific types of the latter, such as violence against the unborn child, shaken baby syndrome, pediatric condition falsification, youth prostitution and witnessing of high-conflict separations. We refer to all these types and forms of violence jointly as ‘violence’ in this editorial (see Fig. 1.)

**Fig. 1:**
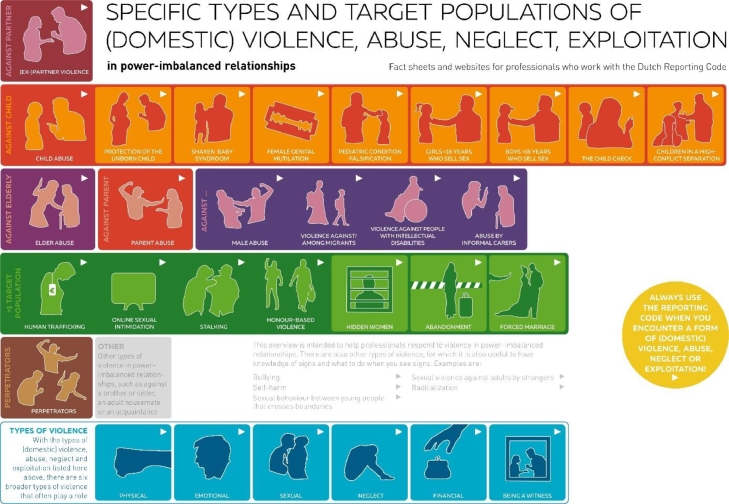
Overview fact sheets about types of violence.

In many cases violence is inflicted upon people by others with whom they have a close relationship of intimacy or trust, such as partners or former partners, parents or other family members, teachers, colleagues, neighbours, carers and spiritual/religious guides (Viergever et al., 2018). Recognition of violence is particularly low when nurses or other professionals have limited knowledge of the type of violence in question, as may be the case for lesser-known types of violence such as human trafficking (Ahn et al., 2013), elder abuse (Alt et al., 2011) and female genital mutilation (Balfour et al., 2016). While up to 88% of people who are trafficked into sexual exploitation encounter a health worker during their exploitation, less than 20% of health workers knows enough to identify and appropriately support this group. (Ross et al., 2015; Dutch National Rapporteur on Human Trafficking and Sexual Violence against Children, 2017)

To improve the degree to which violence is identified and to help professionals act appropriately, the “Signalling and Acting upon Lesser-known Violence” project, a joint project with more than 50 organisations in the Netherlands, produced guidance on identifying and acting on a range of types of violence, including many lesser-known types. The results of this project were recently published in the *British Journal of General Practice* (Viergever, et al., 2022). The publication presents the two main results of the project: first, an overview of various types of violence relevant to the Dutch context (Fig. 1), which in addition to the types of violence noted above, highlights violence in or against certain subpopulations such as migrants or people with a mental disability, which can have specific characteristics and points of attention.

A second output of the project was 24 factsheets that provide an overview of several lesser-known types of violence, including the signs, risk factors and special things to pay attention to for that type of violence. The fact sheets also identify organisations that can provide support. The points of attention for professionals in responding to each type of violence may vary. For example, some types of violence tend to present very late, such as parent abuse and honour-based violence. They therefore require swift action. Considerations around breaking confidentiality to report the violence vary for some situations, for example with child abuse and violence against adults with mental disabilities. By helping professionals to deepen their knowledge about each type of violence, factsheets such as these make it more likely that each type of violence is identified and appropriately responded to.

While the “Signalling and Acting upon Lesser-known Violence” project was conducted in the Netherlands, violence in all its forms is a global health problem. The global burden of disease attributable to violence is hard to estimate, in part because it is so often unrecognised; but it is estimated that annually half of the world's children may experience violence each year (Stanton et al., 2021) and that one in five women have been physically or sexually abused by a man at some time in their lives (Sarah Venis, 2002). The *International Journal of Nursing Studies* has published much on workplace violence towards staff, and research recognises how prevalent such violence is (Havaei et al., 2020; Pihl-Thingvad, 2021). Whilst the role of nurses in addressing wider societal issues in violence has received much less attention in our pages, the issue has begun to receive the attention it deserves with papers in recent years addressing the underlying causes of violence, including the perpetrators of intimate partner violence (Nesset, 2021) and police violence (Jeffers, 2021).

As a resource to professionals around the world the materials from the “Signalling and Acting upon Lesser-known Violence” project have been made freely available in Dutch and in English under a CC BY 4.0 license, meaning anyone may use and adapt these materials. They can be accessed as supplementary materials to this editorial (supplementary file 1). Country-specific adaptations to these materials to fit local circumstances are encouraged together with the addition of local resources. In addition, the format could be used to create expanded materials addressing other types of violence. In recognition of the vital importance of this topic this editorial will be published in both the *International Journal of Nursing Studies* and in an open access format in the *International Journal of Nursing Studies Advances*. Other journals will also be free to adapt and republish this editorial and the linked material.

Nurses and midwives work at the centre of health care provision and, as a result, have extensive contact with patients. This makes them uniquely well-situated to identify signs of violence. Therefore, we hope professional nursing and midwifery associations will use, adapt, expand, and improve the factsheets developed for this project, so that ALL types of violence, including the lesser-known types, may be better identified and addressed in the future.

## Funding

The “Signalling and Acting upon Lesser-known Violence” project described here, and the resources produced, were funded by grants from the Dutch Ministry of Health, Welfare and Sports and by contributions of the organizations working on the project.

## Declaration of Competing Interest

We have no conflicts of interest to report.

## Appendix Supplementary materials

Image, application 1

## Footnotes

Supplementary file 1. Zip file that contains factsheets about specific types of violence. Supplementary material associated with this article can be found, in the online version, at doi:10.1016/j.ijnsa.2022.100098.
