# Supplementary material for: Lesser-known types of violence: Helping nurses and midwives to signal and act
Source: Int J Nurs Stud Adv. 2022 Sep 17;4:100098. doi: 10.1016/j.ijnsa.2022.100098 (PMC11080451; doi:10.1016/j.ijnsa.2022.100098)
Supplement: Supplementary file 1 [file mmc1.zip › Factsheets Dutch/achterlating.pdf]

GEDWONGEN ACHTERLATING

GEBRUIK BIJ ELKE VORM VAN HUISELIJK GEWELD EN KINDER-MISHANDELING DE MELDCODE! ▶

WAT IS EEN GEDWONGEN ACHTERLATING?

Bij achterlating wordt een kind, jongere, vrouw of man tegen haar of zijn wil in het buitenland achtergelaten terwijl de rest van het gezin/familie terugkeert naar Nederland. Er is een onvrijwillige breuk met de sociale omgeving in Nederland. Achterlating gebeurt op initiatief van echtgenoot, ouder(s) of familie. Door afname van reis- of verblijfsdocumenten wordt geprobeerd terugkeer te voorkomen. Het slachtoffer verkeert in het buitenland veelal in een afhankelijke en/of geïsoleerde positie. Bij jongeren zijn motieven voor achterlating vaak verwesteren of voorkomen van wangedrag (verkeerde vrienden, LHBTI). Bij vrouwen speelt vaak huwelijksproblematiek of aantasting van de familie-eer. Het kan een manier zijn om van de vrouw af te komen om een nieuw huwelijk mogelijk te maken.

SIGNALEN: HOE KAN IK ZIEN DAT IEMAND SLACHTOFFER IS?

Preventie van achterlating vereist dat je alert bent op signalen en deze bespreekt met de cliënt. De signalen zijn aspecifiek. Mogelijke signalen zijn:

- Mag niet beschikken over eigen identiteitsbewijs
- Plotseling of eerder op vakantie gaan dan gepland
- Bang op vakantie te gaan, niet terugkomen van vakantie/familiebezoek
- Onverklaarbare afwezigheid
- Gedragsverandering: teruggetrokken, verlegen, bang, boos
- Puberteit: opgroeien tussen culturen met botsende waarden/normen
- Verkeerde vrienden in de ogen van ouders
- Conflicten tussen ouders en kind
- Weglopen van huis
- Hulpverlening past onvoldoende bij vraag van ouders
- Onderzoek noodzaak bemoeienis jeugdzorg

RISICOFACTOREN: WIE IS EXTRA KWETSBAAR VOOR DIT GEWELD?

Mogelijk risico op achterlating als een meisje/vrouw of jongen/man in één of meer risicogroepen valt:

- Kinderen, jongeren of getrouwde vrouwen met en zonder kinderen afkomstig uit een gesloten gemeenschap waar traditionele denkbeelden heersen over rol, positie, opvoeding en seksualiteit van vrouwen en mannen.
- Jonge vrouwen met een partnerafhankelijke verblijfsvergunning (en hun jonge kinderen), jongeren met een LVB en/of die speciaal onderwijs volgen.

AANDACHTSPUNTEN VOOR DIT TYPE GEWELD BIJ HET DOORLOPEN VAN DE 5 STAPPEN IN DE MELDCODE

Maak gebruik van de meldcode en de meldcode eergerelateerd geweld. Daarnaast:

- Vraag advies en raadpleeg altijd een deskundige. Het voorkomen van en reageren op achterlating vraagt specifieke expertise.
- Neem direct contact op met de politie bij acute dreiging van de veiligheid.
- Voer i.v.m. veiligheid van het slachtoffer het gesprek met de partner, ouders/familie nadat de situatie en de risico's van zo'n gesprek met behulp van een deskundige getaxeerd zijn.
- Slachtoffer in het buitenland: voer i.v.m. de veiligheid geen gesprek met ouders, partner of familie.

MEER INFORMATIE

Zie de bronnen en de factsheet eergerelateerd geweld.

FEITEN EN CIJFERS

- Aantal slachtoffers wordt geschat tussen 182 en 815 per jaar. Dat is meer dan het aantal meldingen van 178 per jaar.
- Vooral jongeren tussen 11 en 20 jaar en volwassen vrouwen tussen 20 en 35 jaar.
- Achterlating komt voor in landen in Noord Afrika (Marokko, Algerije, Egypte), West Afrika (Guinee, Ghana, Nigeria), Oost Afrika (Somalië, Kenia, Ethiopië, Soedan) en het Midden Oosten (Irak, Iran, Afghanistan, Turkije, Pakistan, India, Saoedi-Arabië, Syrië).
- Opleidingsniveau: zowel hoog als laagopgeleide jongeren kunnen slachtoffer worden.

ADVIES / MELDEN

Voor advies, melden en/of doorverwijzing naar opvang en/of andere hulp, bel:

- Veilig Thuis **0800 20 00**
- Landelijk Knooppunt Huwelijksdwang en Achterlating (LKHA) **070 34 54 319**

Bij acuut gevaar bel **112**.

Als het slachtoffer in het buitenland is neem direct contact op met het LKHA. Hulp nodig in het buitenland, neem contact op met de Nederlandse ambassade.

ENGELSE VERTALING

Zie hier.

Deze factsheet is ontwikkeld door het Landelijk Knooppunt Huwelijksdwang en Achterlating i.s.m. diverse ketenpartners.

november 2018
