# Supplementary material for: Lesser-known types of violence: Helping nurses and midwives to signal and act
Source: Int J Nurs Stud Adv. 2022 Sep 17;4:100098. doi: 10.1016/j.ijnsa.2022.100098 (PMC11080451; doi:10.1016/j.ijnsa.2022.100098)
Supplement: Supplementary file 1 [file mmc1.zip › Factsheets Dutch/achterlating-bronnen.pdf]

BRONNEN GEDWONGEN ACHTERLATING

Dit bestand geeft een overzicht van organisaties die betrokken zijn geweest bij de ontwikkeling van de bijbehorende factsheet en van beschikbare achtergrondinformatie (bronnen).

BETROKKEN ORGANISATIES

In het maken van deze factsheet over gedwongen achterlating voor professionals in alle beroepen die een meldcode huiselijk geweld en kindermishandeling hanteren, hebben de volgende organisaties input geleverd:

- Het Landelijk Knooppunt Huwelijksdwang en Achterlating. Voor vragen en/of opmerkingen over de factsheet, kunt u emaillen met de hoofdauteur: Diny Flierman, d.flierman@veiligthuishaaglanden.nl
- Augeo Foundation, Edith Geurts
- Bureau Tangram, Suzanne Tan
- CoMensha, Rik Viergever
- Fier - expertise en behandelcentrum op het terrein van geweld in afhankelijkheidsrelaties, Achille van Hees
- GGD GHOR Nederland, Annette Duenk en Sandra Hamming
- Landelijk Expertisecentrum Eergerelateerd Geweld, Korps nationale politie, Janine Janssen.
- Leger des Heils Jeugdbescherming & Reclassering, Juul Polders
- Movisie, Oka Storms
- Sterk Huis, Diane de Winter
- Veilig Thuis, Sabina van der Meer
- Vereniging Vertrouwensartsen Kindermishandeling (VVAK)/ Veilig Thuis, Juliette Heetman
- Verwey-Jonker Instituut, Eliane Smits van Waesberghe.

BRONNEN

De volgende documenten en informatiebronnen geven meer informatie over de signalen van gedwongen achterlating, risicofactoren, en dingen om op te letten bij het doorlopen van de 5 stappen van de meldcode huiselijk geweld en kindermishandeling.

Documenten

- Bakker, H., Storms, O. (2015). De Meldcode bij (vermoedens van) eergerelateerd geweld. https://www.movisie.nl/publicatie/meldcode-vermoedens-eergerelateerd-geweld
- Bartels, E. A. C. (2005). Onderzoeksnotitie over migrantenvrouwen en kinderen die gedwongen zijn achtergelaten in landen van herkomst. (Voorstudie; No. 6). Den Haag: Advies Commissie Vreemdelingenzaken.
- Checklist EGG (eergerelateerd geweld).
- https://www.politie.nl/themas/eergerelateerd-geweld-voor-professionals.html
- Corringting, P. (2013). Going back 'home' Somali parents sending back their children from the Netherlands to Somalia. Master's thesis Social and Cultural Anthropology. Amsterdam: VU University.
- Herken de signalen en ga in gesprek. Tips voor professionals.
- http://www.huwelijksdwangenachterlating.nl/sites/www.huwelijksdwangenachterlating.nl/files/downloads/signaalkaart.pdf
- Janssen, J. (2017). Focus op eer. Een verkenning van eerzaken voor politieambtenaren en andere professionals. Den Haag: Boom criminologie. Onder meer voor relatie tussen eergerelateerd geweld en achterlating.
- Smits van Waesberghe, E., Sportel, I., Drost, E., Eijk, E. van, & Diepenbrock, E. (2014). Zo zijn we niet getrouwd. Een onderzoek naar omvang en aard van huwelijksdwang, achterlating en huwelijkse gevangenschap. Utrecht: Verweij-Jonker Instituut. https://www.verwey-jonker.nl/doc/vitaliteit/7414_Zo%20zijn%20we%20niet%20getrouwd_web.pdf

Websites

- Website Landelijk Knooppunt Huwelijksdwang en Achterlating: www.huwelijksdwangenachterlating.nl
- https://www.politie.nl/themas/eergerelateerd-geweld.html
- https://www.nederlandwereldwijd.nl/hulp-bij-nood/huwelijksdwang
