# Supplementary material for: Lesser-known types of violence: Helping nurses and midwives to signal and act
Source: Int J Nurs Stud Adv. 2022 Sep 17;4:100098. doi: 10.1016/j.ijnsa.2022.100098 (PMC11080451; doi:10.1016/j.ijnsa.2022.100098)
Supplement: Supplementary file 1 [file mmc1.zip › Factsheets Dutch/eergerelateerd-geweld.pdf]

EERGERELATEERD GEWELD

EER

Eer heeft te maken met de reputatie van integriteit en betrouwbaarheid. In sommige families en gemeenschappen speelt de zedelijke familie-eer een grote rol. Morele normen ten aanzien van seksualiteit bepalen de omgang tussen mannen en vrouwen. Worden deze normen gehanteerd dan 'hoor je er bij'. Wanneer als gevolg van moreel wangedrag iemands reputatie niet goed is, dan wordt hij/zij gezien als immoreel en dus slecht. Gevolg is dat het individu kan worden uitgesloten. Door immoreel gedrag van een familielid kan ook de reputatie van integriteit en betrouwbaarheid (familie-eer) van andere familieleden ernstig beschadigd raken met risico op uitsluiting van de familie door de gemeenschap. De familie zal er alles aan doen om uitsluiting te voorkomen.

Individuele keuzen zijn dus van directe invloed op de positie van familieleden en de positie in de gemeenschap. In deze families is het schamen voor "ongewenst" gedrag en angst dat dit gedrag bekend wordt bij derden, groot.

EERGERELATEERD GEWELD

Eergerelateerd geweld is de overkoepelende term voor vormen van intimidatie, dwang, psychisch en fysiek geweld gepleegd vanuit een eermotief. Het geweld moet voorkomen dat een familielid gedrag vertoont dat de familie-eer kan schaden. Er zijn veelal orthodoxe opvattingen over de rol van vrouwen en mannen, en over de seksualiteit en reproductieve rechten van vrouwen. De eer van de vrouw is gekoppeld aan haar seksualiteit. En de seksuele eer van de vrouw is gekoppeld aan de familie-eer. Het betreft gedrag dat bekend is geworden in de omgeving.

Een veel gehanteerde definitie: 'eergerelateerd geweld omvat elke vorm van geestelijk of lichamelijk geweld, gepleegd vanuit een collectieve mentaliteit in reactie op een (dreiging van) schending van de eer van een man of een vrouw en daarmee van zijn of haar familie, waarvan de buitenwereld op de hoogte is of dreigt te raken' (Werkdefinitie ministerie van Justitie, Beke 2005).

Eergerelateerd geweld kan zowel tussen een individu en de groep als tussen verschillende groepen onderling spelen. Wanneer eerschending dreigt, moet deze door de familie voorkomen worden. Wanneer de eer is geschonden, moet deze door de familie worden hersteld. De familie wordt aangesproken door de gemeenschap als zij onvoldoende reageert op een inbreuk op de familie-eer. Meisjes en vrouwen hebben de verantwoordelijkheid om de familie-eer hoog te houden, ouders, mannen en jongens om deze te bewaken en zo nodig te herstellen.

VORMEN VAN EERGERELATEERD GEWELD

Eergerelateerd geweld kent verschillende uitingen en gradaties van ernst, bijvoorbeeld dreigen, grote psychische druk, beperken vrijheden, controleren, isoleren, genezingsrituelen en bezweringen (gedwongen) abortus / afstand van een kind, lichamelijk geweld, gedwongen huwelijk, huwelijkse gevangenschap, verstoting, achterlaten in het buitenland, (vrouwelijke genitale) verminking, gedwongen worden tot zelfmoord, eermoord.

Het is belangrijk dat onderscheid gemaakt wordt tussen eergerelateerd geweld, huiselijk geweld, geweld om de individuele eer, opvoedingsonmacht en kindermishandeling. Bij eergerelateerd geweld speelt namelijk expliciet een eermotief in een collectieve context en zijn daarom andere interventies van toepassing.

FEITEN EN CIJFERS

De politie kent jaarlijks 2500- 3.000 delicten waarbij familie-eer een (vermoedelijke)rol speelt. Gemiddeld 460 delicten vanwege de complexiteit opgepakt door het Landelijk Expertisecentrum Eergerelateerd Geweld. Hiervan eindigen 7 tot 17 zaken fataal (moord, doodslag of suïcide.

ADVIES / MELDEN

Voor advies, melden en/of doorverwijzing naar opvang en/of andere hulp, bel:

- Veilig Thuis **0800 20 00**
  Dit kan ook anoniem.
- Bij acuut gevaar bel **112**

Opvang, hulp kunnen in de regio plaatsvinden. Voor consult / advies en specifieke opvang en behandeling: bel landelijke expertisecentra: Sterk Huis 013 54 33 073 of Fier 088 20 80 000 of www.chatmetfier.nl

Voor advies over huwelijksdwang en achterlating, bel:

- Landelijk Knooppunt Huwelijksdwang en Achterlating (LKHA) op **070 34 54 319**

ENGELSE VERTALING

Zie hier.

Deze factsheet is ontwikkeld door Fier i.s.m. diverse ketenpartners.

november 2018

EERGERELATEERD GEWELD

GEBRUIK BIJ ELKE VORM VAN HUISELIJK GEWELD EN KINDER-MISHANDELING DE MELDCODE! ▶

SIGNALEN

Signalen zijn aspecifiek, dat wil zeggen dat er geen specifieke signalen eergerelateerd geweld zijn.
Signalen die *kunnen* duiden op eergerelateerd geweld:
De angst voor geweld vanuit familie, gedragsveranderingen en of klachten zoals depressie, negatief zelfbeeld, zelfbeschadiging, eetproblemen en slecht slapen, wegloopgedrag, (structureel) verzuim van school en of dagbesteding, verminderde prestaties, op zien tegen komende vakantie, (plotseling) gehaald en /of gebracht worden (surveillance) door familieleden, het onthouden van medische zorg, verbreken van sociale contacten zonder duidelijke reden, verandering van kleding, plotselinge aankondiging van verloving en /of huwelijk, roddels in de gemeenschap, geen beschikking meer hebben over identiteitsdocumenten.
Let op: er is vrijwel altijd sprake van schuld- en schaamtegevoelens en loyaliteitsconflicten.

RISICOFACTOREN

- Vrouwen: een 'vermeende' relatie, seks voor het huwelijk, een [veronderstelde] buitenechtelijke relatie, ongehuwd zwanger, weigeren van een gearrangeerd huwelijk, lesbische identiteit en/of relatie;
- Mannen: relatie/seks met een meisje wat door haar familie niet is toegestaan, relatie/seks met een getrouwde vrouw, weigeren van een gearrangeerd huwelijk, homoseksuele identiteit en/of relatie, weigeren eergerelateerd geweld met betrekking tot . zus, nicht, familielid uit te voeren;
- Foute contacten die bijvoorbeeld dreigen compromitterende beelden op internet te plaatsen (sexting / digitale chantage);
- Scherpe sociale controle, geroddel over (vermeend) wangedrag, wangedrag is bekend in de omgeving;
- (Dreigend) eergerelateerd geweld wordt besproken met politie / hulpverlening (en kan bekend worden);
- Grote onderlinge afhankelijkheid binnen een familie waar familie-eer belangrijk is;
- Eerder eergerelateerd geweld in de familie;
- Strenge / traditionele morele normen t.a.v. zedelijke familie-eer;
- Sociaaleconomische problematiek, sociale isolatie, multiproblematiek.

AANDACHTSPUNTEN VOOR DIT TYPE GEWELD BIJ HET DOORLOPEN VAN DE 5 STAPPEN IN DE MELDCODE

Bij elke vorm van huiselijk geweld en kindermishandeling dien je als professional de meldcode te gebruiken. Algemene meldcode richtlijnen (zoals de 5 stappen) staan niet op deze factsheet beschreven – bezoek daarvoor de link. Wél staan hier aandachtspunten specifiek voor deze vorm van geweld:

- Risicotaxatie en –analyse altijd door experts van politie met behulp van de checklist eergerelateerd geweld;
- Stappen van de meldcode worden anders ingevuld – het is van groot belang niet direct ouders te betrekken of te informeren vanwege het risico op escalatie en gezichtsverlies;
- Er is een specifieke meldcode voor eergerelateerd geweld, zie bij de Bronnen;
- Via de politie en Veilig Thuis kan er consultatie plaats vinden bij, en taxatie en analyse door, het Landelijk Expertisecentrum Eergerelateerd geweld;
- Bij twijfel: zie bij *Advies en melden*.

MEER INFORMATIE

Zie de Bronnen en de 4 andere factsheets over in deze serie over specifieke vormen van eergerelateerd geweld:

- huwelijksdwang
- gedwongen achterlating
- vrouwelijke genitale verminking
- verborgen vrouwen

Deze factsheet is ontwikkeld door Fier i.s.m. diverse ketenpartners. november 2018
