# Supplementary material for: Lesser-known types of violence: Helping nurses and midwives to signal and act
Source: Int J Nurs Stud Adv. 2022 Sep 17;4:100098. doi: 10.1016/j.ijnsa.2022.100098 (PMC11080451; doi:10.1016/j.ijnsa.2022.100098)
Supplement: Supplementary file 1 [file mmc1.zip › Factsheets Dutch/eergerelateerd-geweld-bronnen.pdf]

BRONNEN EERGERELATEERD GEWELD

Dit bestand geeft een overzicht van organisaties die betrokken zijn geweest bij de ontwikkeling van de bijbehorende factsheet en van beschikbare achtergrondinformatie (bronnen).

BETROKKEN ORGANISATIES

In het maken van deze factsheet over eergerelateerd geweld voor professionals in alle beroepen die een meldcode huiselijk geweld en kindermishandeling hanteren, hebben de volgende organisaties input geleverd:

- Fier - expertise en behandelcentrum op het terrein van geweld in afhankelijkheidsrelaties. Voor vragen en/of opmerkingen over de factsheet, kunt u emailen met de hoofdauteur: Gerda de Groot, GdeGroot@fier.nl
- Samen Veilig (Veilig Thuis regio Utrecht)
- Suzanne Tan, bureau TANGRAM
- Diny Flierman, Landelijk Knooppunt Huwelijksdwang en Achterlating
- Sandra Hamming, GGD GHOR
- Marga Haagmans, AUGEO
- Janine Janssen, LEC EGG

BRONNEN

De volgende documenten en informatiebronnen geven meer informatie over de signalen van eergerelateerd geweld, risicofactoren, en dingen om op te letten bij het doorlopen van de 5 stappen van de meldcode huiselijk geweld en kindermishandeling:

Achtergrond

- Bakker, H., Storms, O. (2016). Factsheet De meldcode bij (vermoedens van) eergerelateerd geweld. Utrecht: Movisie.
- Brekelmans, I. & Groot, G. de (2014). De kleuren van Eva en Zahir. Amsterdam: SWP Uitgeverij.
- Dijke, A. van & Terpstra, L. (2010). De dochters van Zahir. Tussen traditie en wereldburgerschap. Amsterdam: SWP Uitgeverij.
- Ermers, R. (2018). Honor Related Violence. A new social psychological Perspective). Routledge, www.routledge.com
- Ferwerda, H.B & Leiden, I. van (2005). Eerwraak of eergerelateerd geweld? Naar een werkdefinitie. Arnhem: Advies- en Onderzoekgroep Beke.
- Janssen, J. & Sanberg, R. (2013) Uniformiteit in cijfers. Mogelijke eerzaken in 2010, 2011 en 2012. Den Haag: Landelijk Expertise Centrum Eer Gerelateerd Geweld.
- Janssen, J. (2013). De rol van religie bij het afbakenen, verklaren en aanpakken van eergerelateerd geweld. Tijdschrift voor Religie, Recht en Beleid (4)1: 5-15.
- Janssen, J. (2017). Focus op eer. Een verkenning van eerzaken voor politieambtenaren en andere professionals. Den Haag: Boom Criminologie.
- Vlamings, B., Geijn. R. van, Brekelmans, I. (2012). Methodiek Safe and Streetwise. Tilburg: Kompaan en De Bocht.
- Werson, S., Lamers, F., Pers, M. van der, & Dijke, A. van (2015). Fier en verder. Meiden over hun leven na de hulpverlening. Amsterdam: Uitgeverij SWP.

Factsheets over specifieke vormen van eergerelateerd geweld

- VGV
- gedwongen huwelijken
- gedwongen achterlating
- gedwongen isolement

Landelijke expertise- en behandelcentra

- www.fier.nl
- www.sterkhuis.nl

Internet

- https://www.huiselijkgeweld.nl/dossiers/eergerelateerd-geweld
- https://www.rijksoverheid.nl/onderwerpen/eergerelateerd-geweld
- www.huwelijksdwangenachterlating.nl
- https://www.movisie.nl/publicatie/eergerelateerd-geweld-seksuele-genderdiversiteit
- https://www.politie.nl/themas/eergerelateerd-geweld.html
- https://www.politie.nl/themas/eergerelateerd-geweld-voor-professionals.html
- https://www.eerenvrijheid.nl
- https://www.kis.nl/trefwoorden/eergerelateerd-geweld
- https://www.huiselijkgeweld.nl/nieuws/2018/270218_-eergerelateerd-geweld-is-geen-huiselijk-geweld-
- https://www.huiselijkgeweld.nl/doc/feiten/factsheet_EG_20p_aug%202011.pdf

Signaleren

- http://www.leceergerelateerdgeweld.nl/herkennen

Meldcode

- https://www.meldcode.nu/eergerelateerd-geweld/
