# Supplementary material for: Lesser-known types of violence: Helping nurses and midwives to signal and act
Source: Int J Nurs Stud Adv. 2022 Sep 17;4:100098. doi: 10.1016/j.ijnsa.2022.100098 (PMC11080451; doi:10.1016/j.ijnsa.2022.100098)
Supplement: Supplementary file 1 [file mmc1.zip › Factsheets Dutch/financiele-uitbuiting.pdf]

FINANCIEEL MISBRUIK

WAT IS FINANCIEEL MISBRUIK

Financieel misbruik is ongewenst en ongeoorloofd gebruik van geld en/of goederen of andere bezittingen van een oudere door iemand uit de huiselijke kring. De mate kan variëren van het zich niet-realiseren tot aan een strafbaar feit. Voorbeelden van financieel misbruik zijn boodschappen voor zichzelf betalen met de pinpas van een ander, bedragen overmaken naar de eigen rekening, diefstal van - al of niet waardevolle en dierbare - bezittingen, pinpasfraude, gedwongen testamentwijziging, wilsbeschikking of hypotheekop-name, verkoop van eigendommen, misbruik van machtigingen, koop op naam van slachtoffer, financieel kort houden.

WIE ZIJN DE PLEGERS?

Plegers kunnen familieleden zijn, zoals (ex) partners en (klein) kinderen, maar ook huisvrienden. Daarnaast kunnen het ook personen zijn van wie het slachtoffer afhankelijk is zoals professionele hulpverleners of een vrijwilliger.

SIGNALEN

De signalen dat er sprake kan zijn van financiële uitbuiting zijn de volgende:

- Verdwijnen van geld, goederen of waardevolle spullen
- Onverklaarbare geldopnames of kosten
- Plotseling geldgebrek
- Betaalachterstand: huur, energie, rekeningen
- Afgesloten van gas en elektra
- Toenemend aantal schuldeisers
- Brieven van incassobureaus
- Plotselinge verzoeken tot testamentswijzigingen in aanwezigheid van een familielid (pleger)
- Vermijden of weigeren informatie te geven over financiële omstandigheden door oudere of mantelzorger (pleger)
- Weerstand en/of verbaal geweld (door de vermoedelijke pleger) als vermoedens besproken worden
- Verbaal geweld of dreigementen (door de vermoedelijke pleger) tegen de oudere als deze zich wil verzetten
- Verslaving of financiële problemen bij de vermoedelijke pleger
- Verwaarloosde omgeving en/of uiterlijk
- Afwezigheid van voeding, signalen van ondervoeding

N.B. Deze laatste twee signalen kunnen ook duiden op geestelijke achteruitgang bij een gebrekkig sociaal netwerk en/of op zelfverwaarlozing (iemand is niet meer in staat is goed voor zichzelf te zorgen).

RISICOFACTOREN [i]

Belangrijkste risicofactoren zijn de mate van (zorg)afhankelijkheid, de loyaliteitsrelatie die er is tussen pleger en slachtoffer en de omstandigheden waarin de pleger verkeert, zoals schulden, psychische problemen, verslaving. Daarnaast speelt ook mee dat ouderen te goed van vertrouwen kunnen zijn en hun financiële zaken niet goed geregeld hebben voordat hun gezondheid zodanig achteruitgaat, dat zij niet goed meer in staat zijn om hun financien zelf te regelen. Er is een checklist [iv] die ouderen zelf kunnen invullen. Op basis daarvan kunnen zij, al dan niet in overleg met hun naasten, of de bank of een notaris, besluiten of zij verdere zaken willen of moeten vastleggen om het risico op financieel misbruik te verkleinen.

Onwetendheid: Geen kennis en kunde van het digitaal bankverkeer waardoor men financiële en administratieve zaken over laat aan anderen; Te weinig actuele kennis over de toenemende complexe regelgeving

Loyaliteit en familiebanden: Door loyaliteit, bloedband, schaamte, schuldgevoel, onzekerheid (heb ik het wel juist?) en/of angst voor vermindering van contact durven ouderen hun zorgen en

HOE VAAK KOMT FINANCIEEL MISBRUIK VOOR?

In het prevalentie onderzoek naar ouderenmishandeling van Regioplan komt naar voren dat één op de twintig ouderen (170.000) vanaf het 65ste levensjaar te maken krijgt met een vorm van ouderenmishandeling. De meest gerapporteerde vorm van ouderenmishandeling door ouderen zelf is financieel misbruik (3% van alle ouderen. Financieel misbruik kan ook voorkomen bij andere groepen kwetsbare burgers zoals mensen met een verstandelijke beperking of andere mensen die een sterke afhankelijkheidsrelatie hebben met de pleger. In ons land zijn op dit moment 3,1 miljoen ouderen. Uit de gezondheidsmonitor uit 2016 [ii] komt naar voren dat landelijk gezien 1,1% van de onderzochte groep ouderen te maken heeft gehad met financieel misbruik. Dit komt neer op 34.100 ouderen. De regionale verschillen liepen uiteen van 0,7 – 2,0%. Het onderzoek van Regioplan [iii] vond in het onderzoek dat zij verrichten een jaarprevalentie van 0,9% en sinds het 65e levensjaar 3,0%. Dit laatste percentage komt overeen met 93.000 ouderen. De resultaten van beide onderzoeken komen dus in grote mate overeen. Beide onderzoeken gebruiken de term financiële benadeling en de leeftijdsgrens van 65 jaar.

FINANCIEEL MISBRUIK

signalen niet bespreekbaar te maken met een professional of vrijwilliger. Er zijn harmonieuze en conflictrijke families. Financiële benadeling is mede afhankelijk van hoe familieleden met elkaar omgaan en tot elkaar staan. Dit heeft te maken met loyaliteit en gehechtheid. Ouders kunnen vanwege het gevoel dat zij vroeger hun kind tekort gedaan hebben (door ziekte of andere redenen) vanuit schuldgevoel langer toelaten dat zij financieel uitgebuit worden.
Er zijn kinderen die menen dat ze in hun leven tekort zijn gedaan, achter zijn gesteld, verwaarloosd of mishandeld. Zij kunnen wraak nemen door hun ouder(s) financieel te benadelen.
Ook kunnen familieruzies ontstaan door de aanspraken die kinderen menen te hebben. Bij familievetes gebeurt het vaak dat één van de kinderen een centrale rol gaat spelen en de overige familieleden buiten spel zet met als risico: financieel misbruik van de oudere.

Fysieke beperkingen: Door beperkingen in horen, zien en/of mobiliteit kan men een verhoogd risico lopen doordat men niet (meer) in staat zijn eigen financiële belangen (goed)te behartigen
Sociale beperkingen: Mensen met een klein sociaal netwerk en weinig contacten buitenshuis lopen een verhoogd risico om slachtoffer te worden van financieel misbruik. Het verlangen naar contacten zorgt er soms voor dat niet kritisch gekeken wordt met wie dat contact aangegaan wordt. Dit heeft als risico dat ze een onbetrouwbaar iemand in de arm nemen, en dat die handig gebruik maakt van hun afhankelijkheid en behoefte.
Psychische problemen: Bij ouderen speelt vaak mee dat ze nog rouwen over het verlies van hun partner en zelfstandigheid, waarbij depressieve gevoelens invloed hebben op hun energie om hun financiën goed te regelen. Daarnaast is geestelijke achteruitgang een risicofactor.

Laagbegaafd of laaggeletterd zijn; cognitieve veranderingen: lopen een grotere kans om te maken te krijgen met financieel misbruik, omdat ze geen overzicht meer hebben en ook de vaardigheden missen om de regie over eigen financiën zelf in handen te houden.

AANDACHTSPUNTEN

Gaat het om financiële uitbuiting **in familieverband of huiselijke sfeer** dan is Wet meldcode van toepassing: bij elke vorm van huiselijk geweld en kindermishandeling dien je als professional de meldcode te gebruiken (zie deze link). Algemene meldcode richtlijnen (zoals de 5 stappen) staan niet op deze factsheet beschreven – bezoek daarvoor de link. Wél staan hier aandachtspunten specifiek voor deze vorm van geweld:

- Banken en notarissen spelen al een belangrijke rol in het voorkomen van financiële benadeling.
- Bewindvoering en aangifte doen bij de politie zijn (uiterste) mogelijkheden om financiële benadeling te stoppen.
- Wanneer het gaat om diefstal door medecliënten in een intramurale instelling, moet altijd worden gekeken naar de geestelijke gesteldheid van de pleger. Psychogeriatrische problematiek, zoals dementie, kan ten grondslag liggen aan het gedrag. Vaak komen voorwerpen weer terecht.
- Financiële uitbuiting kan ook gepleegd worden door een professional of door een vrijwilliger.
- Beide vallen onder de Wet kwaliteit, klachten en geschillen in de zorg (Wkkgz) en ernstige incidenten moeten gemeld worden bij de Inspectie Gezondheidszorg en Jeugdzorg (IGJ).

ADVIES / MELDEN

Voor advies, melden en/of doorverwijzing naar opvang en/of andere hulp, bel:

- Veilig Thuis **0800 20 00**

Bij acuut gevaar bel **112**

MEER INFORMATIE

Zie de bronnen.

ENGELSE VERTALING

Zie hier.

Deze factsheet is ontwikkeld door Movisie i.s.m. diverse ketenpartners.
