# Supplementary material for: Lesser-known types of violence: Helping nurses and midwives to signal and act
Source: Int J Nurs Stud Adv. 2022 Sep 17;4:100098. doi: 10.1016/j.ijnsa.2022.100098 (PMC11080451; doi:10.1016/j.ijnsa.2022.100098)
Supplement: Supplementary file 1 [file mmc1.zip › Factsheets Dutch/financiele-uitbuiting-bronnen.pdf]

BRONNEN FINANCIEEL MISBRUIK

Dit bestand geeft een overzicht van organisaties die betrokken zijn geweest bij de ontwikkeling van de bijbehorende factsheet en van beschikbare achtergrondinformatie (bronnen).

BETROKKEN ORGANISATIES

In het maken van deze factsheet over financieel misbruik hebben de volgende organisaties input geleverd:

- Movisie. Voor vragen en/of opmerkingen over de factsheet, kunt u emailen met de hoofdauteur: Nico van Oosten, N.vanOosten@movisie.nl
- Veilig Thuis. Marianne van der Krans, Cathelijne Berkvens, Sigrid van den Boer, Anne-Marie Raat, Laura van der Voorn en Gerda Rosman

BRONNEN

De volgende documenten en informatiebronnen geven meer informatie over de signalen van financieel misbruik, risicofactoren, en dingen om op te letten bij het doorlopen van de 5 stappen van de meldcode huiselijk geweld en kindermishandeling:

i https://www.aanpak-ouderenmishandeling.nl/doc/Factsheet-financieel-misbruik-en-uitbuiting-ouderen.pdf (2009)

ii https://www.volksgezondheidenzorg.info/onderwerp/sociale-omgeving/ouderenmishandeling#node-financieel-benadeeld-de-thuissituatie-ggd-regio (2016)

iii Bakker, L., Witkamp, B., Timmermans, M., Janssen, J., Lindenberg, J. (2018). Aard en omvang ouderenmishandeling. Amsterdam: Regioplan, Avans Hogeschool, Leyden Academy on Vitality and Ageing. https://www.regioplan.nl/publicaties/slug/type/rapporten/slug/aard_en_omvang_ouderenmishandeling

iv Checklist voor ouderen ten behoeve van het voorkomen vanfinanciële uitbuiting. April 2016. Ministerie van Volksgezondheid, Welzijn en Sport in samenwerkingmet de Brede Alliantie 'Veilig financieel ouder worden'. https://www.aanpak-ouderenmishandeling.nl/doc/Checklist-ouder-voorkomen-van-financiele-uitbuiting.pdf
