# Supplementary material for: Lesser-known types of violence: Helping nurses and midwives to signal and act
Source: Int J Nurs Stud Adv. 2022 Sep 17;4:100098. doi: 10.1016/j.ijnsa.2022.100098 (PMC11080451; doi:10.1016/j.ijnsa.2022.100098)
Supplement: Supplementary file 1 [file mmc1.zip › Factsheets Dutch/gedwongen-isolatie.pdf]

VERBORGEN VROUWEN

GEBRUIK BIJ ELKE VORM VAN HUISELIJK GEWELD EN KINDERMISHANDELING DE MELDCODE! ▶

WAT / WIE ZIJN VERBORGEN VROUWEN?

Verborgen vrouwen zijn vrouwen die door de partner en/of (schoon)familie gedwongen worden om geïsoleerd te leven. Vaak gaat dit gepaard met psychische intimidatie of (dreiging met) fysiek geweld. Zij worden veelal tegen hun eigen wil thuis opgesloten en mogen geen of slechts zeer beperkt contact hebben met anderen. Als er buitenshuis contact is, gebeurt dit vaak onder begeleiding en controle van partner en/of familie. Verborgen vrouwen hebben weinig tot geen mogelijkheden om te participeren in de samenleving, zijn niet of nauwelijks zichtbaar voor hulpverlening, en weten ook zelf de weg naar hulp niet te vinden (of pas na lange tijd).

SIGNALEN: INDICATIES VAN MOGELIJKE VERBORGENHEID

- De vrouw komt niet of nauwelijks buiten.
- De vrouw komt niet naar de gemaakte afspraken.
- De vrouw wordt als zij naar buiten gaat gecontroleerd of vergezeld door partner of familielid.
- De partner of familielid voert (altijd) in gesprekken met professionals het woord.
- Psychosomatische klachten.
- Huiselijk geweld.
- De deur blijft dicht en ramen zijn verduisterd.
- De vrouw oogt angstig, timide en/of maakt een levenloze indruk.
- Buurtkinderen of klasgenootjes van kinderen mogen nooit komen spelen.
- Kinderen komen onverzorgd, angstig en/ of vermijdend over.
- De vrouw geeft aan bang te zijn voor haar man of familie.
- De vrouw wil geen hulp, uit angst voor meer problemen of geweld.
- Gedwongen inwoning bij (schoon)familie.

RISICOGROEPEN: WELKE VROUWEN ZIJN HET KWETSBAARST OM IN EEN SITUATIE VAN VERBORGENHEID TE BELANDEN?

- Gedwongen inwoning bij (schoon)familie.
- Vrouwen afkomstig uit een gesloten gemeenschap waarin traditionele denkbeelden heersen over de rol en de positie van de vrouw. Ook kunnen tradities en familie eer binnen de gemeenschap een grote rol spelen.
- Vrouwen die voor een huwelijk naar Nederland migreren.
- Vrouwen zonder verblijfsstatus of die voor een verblijfsvergunning afhankelijk zijn van hun partner.
- Vrouwen voor wie een huwelijk gearrangeerd wordt met een man met een psychische of lichamelijke beperking.
- Vrouwen die onder druk van de familie trouwen met een partner (huwelijksdwang).
- Vrouwen die getrouwd zijn met een partner met psychiatrische problemen, zoals extreme achterdocht, jaloezie of paranoïde gedrag.

RISICOFACTOREN: WELKE RISICO'S KUNNEN DE PROBLEMATIEK VAN VERBORGEN VROUWEN BEVORDEREN?

- Sociale druk en controle door partner en/of (schoon)familie en gemeenschap.
- Familie-eer.

FEITEN EN CIJFERS

Cijfers

Naar schatting wonen er alleen al in de grote steden Rotterdam, Amsterdam en Den Haag totaal 600-900 verborgen vrouwen. Landelijke cijfers ontbreken.

Feiten

- De meeste verborgen vrouwen zijn afkomstig uit Marokko en Turkije.
- Verborgen vrouwen zijn vrouwen van alle leeftijden.
- De echtgenoot en zijn familie spelen vaak een grote rol in het verborgen houden van de vrouw.
- De komst of aanwezigheid van kinderen maakt het voor vrouwen nog lastiger om een isolement te doorbreken.
- Verborgen vrouwen hebben vaker te maken met psychische intimidatie en fysiek geweld.

ADVIES / MELDEN

Voor advies, melden en/of doorverwijzing naar opvang en/of andere hulp, bel:

- Veilig Thuis **0800 20 00**
- Bij acuut gevaar bel **112**

ENGELSE VERTALING

Zie hier.

Deze factsheet is ontwikkeld door Verwey-Jonker Instituut / Kennisplatform Integratie en Samenleving i.s.m. diverse ketenpartners.

november 2018

- Laaggeletterdheid, analfabetisme en/of de Nederlandse taal niet spreken.
- Financiële afhankelijkheid.
- Inwoning bij de schoonfamilie.
- Nieuw in Nederland en de weg hier niet kennen.
- Afhankelijke verblijfsvergunning.

DINGEN OM OP TE LETTEN BIJ DIT TYPE GEWELD BIJ HET DOORLOPEN VAN DE 5 STAPPEN IN DE MELDCODE

Bij elke vorm van huiselijk geweld en kindermishandeling dien je als professional de meldcode te gebruiken. Algemene meldcode richtlijnen (zoals de 5 stappen) staan niet op deze factsheet beschreven – bezoek daarvoor de link. Wél staan hier aandachtspunten specifiek voor deze vorm van geweld:

- Professionals in de eerstelijnszorg en in het onderwijs zijn de belangrijkste mogelijke signaleerders van verborgen vrouwen, omdat deze vrouwen nauwelijks participeren in de samenleving.
- Zowel de vrouwen zelf, als hun mogelijke kinderen, dienen op het netvlies te blijven van de hulpverlening.
- Is een verborgen vrouw 'in beeld', dan is het voor een professional van belang om aan te sluiten bij de hulpbehoeften van de vrouw en eventuele kinderen: zij bepaalt het tempo.
- Een professional kan expliciteren dat mannen èn vrouwen in Nederland gelijke rechten hebben om te kunnen participeren in de maatschappij (conform het VN-Vrouwenverdrag). Sommige verborgen vrouwen weten niet dat gedwongen isolement strafbaar is in Nederland en hulp voorhanden is.
- Geeft de vrouw expliciet aan haar isolement te willen opheffen, zorg dan als professional in afstemming met Veilig Thuis voor veilige vervolgstappen.

MEER INFORMATIE

Zie de bronnen en de factsheet over eergerelateerd geweld.

Deze factsheet is ontwikkeld door Verwey-Jonker Instituut / Kennisplatform Integratie en Samenleving i.s.m. diverse ketenpartners.
