# Supplementary material for: Lesser-known types of violence: Helping nurses and midwives to signal and act
Source: Int J Nurs Stud Adv. 2022 Sep 17;4:100098. doi: 10.1016/j.ijnsa.2022.100098 (PMC11080451; doi:10.1016/j.ijnsa.2022.100098)
Supplement: Supplementary file 1 [file mmc1.zip › Factsheets Dutch/gedwongen-isolatie-bronnen.pdf]

BRONNEN VERBORGEN VROUWEN

Dit bestand geeft een overzicht van organisaties die betrokken zijn geweest bij de ontwikkeling van de bijbehorende factsheet en van beschikbare achtergrondinformatie (bronnen).

BETROKKEN ORGANISATIES

In het maken van deze factsheet over verborgen vrouwen voor professionals in alle beroepen die een meldcode huiselijk geweld en kindermishandeling hanteren, hebben de volgende organisaties input geleverd:

- Verwey-Jonker Instituut / Kennisplatform Integratie en Samenleving. Voor vragen en/of opmerkingen over de factsheet, kunt u emailen met de hoofdauteurs: Lisanne Drost en Eliane Smits van Waesberghe, op secr@verwey-jonker.nl
- Sterk Huis, Diane de Winter
- Bureau Tangram, Suzanne Tan
- Politie, Janine Janssen
- Movisie, Oka Storms / Wilma Schakenraad
- Landelijk Knooppunt Huwelijksdwang en Achterlating, Diny Flierman
- Veilig Thuis, Juliette Heetman
- GGD GHOR, Sandra Hamming

BRONNEN

De volgende documenten en informatiebronnen geven meer informatie over de signalen van verborgen vrouwen, risicofactoren, en dingen om op te letten bij dit type geweld bij het doorlopen van de 5 stappen van de meldcode huiselijk geweld en kindermishandeling:

- Drost, L., Smits van Waesberghe, E., Los, V. (2015). Opgesloten in eigen huis. Een onderzoek naar aard en omvang van verborgen vrouwen in Den Haag. Utrecht: Verwey-Jonker Instituut. https://www.verwey-jonker.nl/doc/2015/115002-verborgen-vrouwen-denhaag.pdf
- Drost, L., Goderie, M., Flikweert, M., & Tan, S. (2012). Leven in gedwongen isolement: Een verkennend onderzoek naar verborgen vrouwen in Amsterdam. Utrecht: Verwey-Jonker Instituut. https://www.verwey-jonker.nl/doc/vitaliteit/Verborgen_vrouwen_7249_web.pdf
- Informatieblad verborgen vrouwen gemeente Rotterdam: https://www.huiselijkgeweld.nl/doc/Informatieblad%20verborgen%20vrouwen%202016.pdf
- J. Janssen, Focus op eer. Een verkenning van eerzaken voor politieambtenaren en andere professionals, Den Haag: Boom Criminologie, 2017.
- Musa, S., Diepenbrock, E. (2013). Verborgen vrouwen: een vergeten groep. Een verkennend onderzoek naar aard, omvang en aanpak van de problematiek van verborgen vrouwen in de deelgemeente Delfshaven (Rotterdam). S.l.: Stichting Femmes For Freedom.
- Nieuwsbrief gemeente Rotterdam verborgen vrouwen, juli 2017. https://www.rotterdam.nl/wonen-leven/schadelijke-praktijken/NB2-verborgen-vrouwen.pdf
- https://www.politie.nl/themas/eergerelateerd-geweld-voor-professionals.html
