# Supplementary material for: Lesser-known types of violence: Helping nurses and midwives to signal and act
Source: Int J Nurs Stud Adv. 2022 Sep 17;4:100098. doi: 10.1016/j.ijnsa.2022.100098 (PMC11080451; doi:10.1016/j.ijnsa.2022.100098)
Supplement: Supplementary file 1 [file mmc1.zip › Factsheets Dutch/Geweld-mensen-met-beperking.pdf]

(SEKSUEEL) GRENSOVERSCHRIJDEND GEDRAG JEGENS MENSEN MET EEN

VERSTANDELIJKE BEPERKING

OVER WELKE VORMEN VAN GRENSOVERSCHRIJDEND GEDRAG HEBBEN WE HET?

Mensen met een verstandelijke beperking kunnen – net als mensen zonder deze beperking - te maken krijgen met ontspoorde mantelzorg, financiële uitbuiting, fysieke mishandeling, psychische en emotionele mishandeling, seksueel grensoverschrijdend gedrag, schending van mensen- en burgerlijke rechten en discriminatie. Zij kunnen hiermee te maken krijgen op alle mogelijke plekken. Leidend voor een eventuele melding is de relatie waarin pleger en slachtoffer tot elkaar staan, niet de locatie. Het kan gaan om kindermishandeling, partnergeweld, ouderenmishandeling, kind-oudermishandeling en eergerelateerd geweld. Met name jonge meiden met een (licht) verstandelijke beperking kunnen te maken krijgen met pooierboys.

OM WELKE DOELGROEP GAAT HET?

Bij mensen met een verstandelijke beperking kan deze beperking aangeboren of niet aangeboren zijn (bijvoorbeeld Niet Aangeboren Hersenletsel). Mensen kunnen naast hun verstandelijke beperking ook nog andere beperkingen hebben. In dat geval spreken we van meervoudige beperking. Tot slot kan de verstandelijke beperking of de combinatie van beperkingen in ernst variëren. In deze factsheet beperken we ons tot mensen met een aangeboren verstandelijke beperking en wat daarover bekend is. Door hun beperking zijn deze mensen minder weerbaar. Ze zijn vaak (sterk) afhankelijk van zorg en ondersteuning, met name op het sociale vlak. Door hun verstandelijke beperking zijn ze minder goed in staat om sociale situaties goed in te schatten (EQ), is hun begripsvermogen minder (IQ) en kunnen ze zich verbaal vaak minder goed uitdrukken. Grenzen aangeven is daardoor lastig voor ze. Aan de andere kant worden mensen met een verstandelijke beperking nogal eens overschat. Mede omdat hun beperking minder zichtbaar is en ze zelf hun beperking verbergen en omdat ze geen inzicht hebben in hun beperkingen.

FEITEN EN CIJFERS: HOE VAAK KOMT GRENSOVERSCHRIJDEND GEDRAG VOOR?

De verhoogde kwetsbaarheid voor diverse vormen van grensoverschrijdend gedrag wordt in een groot aantal binnen- en buitenlandse studies bevestigd (zie o.m. Platt et al., 2017; Van der Heijden, 2014; Hughes et al., 2011; Van Berlo et al., 2011;). Grensoverschrijdend gedrag door bekenden (waaronder tussen mensen met een verstandelijke beperking onderling) komt het meest voor (o.a. FRA, 2015).

- De VN vermoedt dat kinderen met een beperking vier keer zoveel te maken hebben met geweld dan kinderen zonder beperking (FRA, 2015).
- Een apart probleem vormt getuige zijn van geweld tussen de ouders of overige familieleden.
- De cijfers voor mishandeling van mensen met een verstandelijke beperking bedragen volgens buitenlands onderzoek 42% voor jongens en 39% voor meisjes, voor volwassenen 44% voor mannen en 46% voor vrouwen (Platt et al., 2017). Een eerdere review laat zien dat mensen met een verstandelijke beperking de meest kwetsbare groep zijn. In deze review komt naar voren dat 26-90% van de vrouwen en 29-86% van de mannen ooit te maken heeft gehad met een vorm van mishandeling (Hughes et al., 2011).
- Het onderzoek van Van Berlo et al. (2011) naar seksueel grensoverschrijdend gedrag onder mensen met een beperking is het meest recente in ons land op dit gebied. Het laat zien dat hoe

GEBRUIK BIJ ELKE VORM VAN HUISELIJK GEWELD EN KINDERMISHANDELING DE MELDCODE! ▶

ADVIES / MELDEN

Voor advies, melden en/of doorverwijzing naar opvang en/of andere hulp, bel:

- Veilig Thuis **0800 20 00**

Grensoverschrijdend gedrag door professionals kan gemeld worden bij:

- het Landelijk Meldpunt Zorg van de Inspectie voor Gezondheidszorg en Jeugdzorg (IGJ), tel. **088 120 50 20**

Bij acuut gevaar bel **112**.

ENGELSE VERTALING:

Zie hier.

MEER INFORMATIE

Zie de bronnen.

Deze factsheet is ontwikkeld door Movisie i.s.m. diverse ketenpartners.

specifieker wordt gevraagd naar verschillende vormen van seksueel grensoverschrijdend gedrag, de percentages toenemen. De life time prevalentie voor volwassen vrouwen met een (lichte) verstandelijke beperking bedraagt 72% en voor mannen 44%. 23% van de vrouwen is ooit verkracht en 7% van de mannen. Beneden de 16 jaar zijn de percentages voor meisjes 28% en voor jongens 19%.

- Voor pesten zijn cijfers gevonden voor de vergelijking tussen leerlingen met en zonder handicap (Kennisplein gehandicaptensector). Een gehandicapte leerling wordt vijf maal vaker gepest, 10,8% tegen 2,2%. Kinderen met een beperking worden 2 tot 3 keer zo vaak gepest als andere kinderen. De helft van de kinderen met een beperking is bang voor hun leeftijdsgenoot. 82% van kinderen en jonge mensen (7-19 jaar) met een leerstoornis hebben een ervaring met pesten.

SIGNALEN: HOE KAN IK ZIEN DAT IEMAND SLACHTOFFER IS?

Signalen bij het slachtoffer

De belangrijkste signalen voor fysieke mishandeling zijn verwondingen, blauwe plekken verspreid over het lichaam, en een combinatie van oude en nieuwe botbreuken. Ook brandwonden komen voor. Ook bij mensen met een verstandelijke beperking zijn plotselinge gedragsveranderingen, zoals terugtrekgedrag en agressie signalen. Voor seksuele grensoverschrijding zijn signalen dat het slachtoffer zelf grensoverschrijdend gedrag gaat vertonen en het gebruik van seksuele woorden die niet leeftijdsadequaat zijn. Toch is signaleren bij mensen met een verstandelijke beperking lastig. Zij onttrekken zich wat meer aan het oog vanwege het leven met hun beperkingen in het algemeen en in het bijzonder die in hun communicatie en inschatting van wat ze hebben meegemaakt, zo blijkt uit het onderzoek van Van Berlo et al. (2011). Mensen met een verstandelijke beperking hebben over het algemeen minder zelfvertrouwen, zodat dit niet als een specifiek signaal kan worden gezien.

Signalen bij de pleger

Wat geldt voor slachtoffers, geldt ook voor plegers: signalen wijken niet af van de signalen bij de zorg voor andere mensen met afwijkend gedrag.

RISICOFACTOREN

Er zijn bij mensen met een beperking risicofactoren op slachtofferniveau (ernst van de beperking en afwijkend gedrag), plegerniveau (bijvoorbeeld stress, ouders met een licht verstandelijke beperking) en – als degene met een verstandelijke beperking in een zorginstelling verblijft of gebruik maakt van een woning of ondersteuning van een instelling – op het organisatieniveau van de zorginstelling. Als er bijvoorbeeld te weinig deskundig personeel is, is het risico op onderling grensoverschrijdend gedrag tussen cliënten en ook op familiaal geweld groter. Ook wordt het gepleegde gedrag minder vaak en goed gesignaleerd.

AANDACHTSPUNTEN

Gaat het om grensoverschrijdend gedrag in familieverband of huiselijke sfeer, inclusief medebewoners dan is Wet Meldcode van toepassing en moeten de vijf stappen daarin worden gezet. Speciale aandacht verdient de kindcheck bij ouders met een (licht) verstandelijke beperking en/of laag geletterdheid. Veilig Thuis is dan de instelling waar een melding moet plaatsvinden. Het landelijke telefoonnummer daarvoor is 0800 20 00.

Deze factsheet is ontwikkeld door Movisie i.s.m. diverse ketenpartners.
