# Supplementary material for: Lesser-known types of violence: Helping nurses and midwives to signal and act
Source: Int J Nurs Stud Adv. 2022 Sep 17;4:100098. doi: 10.1016/j.ijnsa.2022.100098 (PMC11080451; doi:10.1016/j.ijnsa.2022.100098)
Supplement: Supplementary file 1 [file mmc1.zip › Factsheets Dutch/Geweld-mensen-met-beperking-bronnen.pdf]

Dit bestand geeft een overzicht van organisaties die betrokken zijn geweest bij de ontwikkeling van de bijbehorende factsheet en van beschikbare achtergrondinformatie (bronnen).

BETROKKEN ORGANISATIES

In het maken van deze factsheet over (seksueel) grensoverschrijdend gedrag jegens mensen met een verstandelijke beperking voor professionals in alle beroepen die een meldcode huiselijk geweld en kindermishandeling hanteren, hebben de volgende organisaties input geleverd:

- Movisie. Voor vragen en/of opmerkingen over de factsheet, kunt u emailen met de hoofdauteur: Nico van Oosten, N.vanOosten@movisie.nl
- Bertine Spooren – GGD Amsterdam
- Sandra Hamming – GGD GHOR Nederland
- Hilair Balsters – Vilans
- Jolanda den Hartog – SIEN
- Marijke Lammers – Lammers Advies en Training
- Rianne van Beurden – Prisma
- Susan Dijkman – Veilig Thuis Kennemerland
- Wilma Schakenraad – Movisie

BRONNEN

De volgende documenten en informatiebronnen geven meer informatie over de signalen van (seksueel) grensoverschrijdend gedrag jegens mensen met een verstandelijke beperking, risicofactoren, en dingen om op te letten bij het doorlopen van de 5 stappen van de meldcode huiselijk geweld en kindermishandeling:

- Berlo, W. van, Haas. S. de., Oosten, N. van, Dijk, L. van, Brants, L., Tonnon, S., & Storms, O. (2011). Beperkt Weerbaar - Een onderzoek naar seksueel geweld bij mensen met een lichamelijke, zintuiglijke of verstandelijke beperking, Utrecht: Rutgers WPF/MOVISIE.
- Casteel, C. Martin, S.L., Smith, J.B., Gurka, K.K. en Kupper, L.L. (2008). National study of physical and sexual assault among women with disabilities. Injury Prevention; vol. 14: 87-90
- FRA (2015). Violence against children with disabilities: easy read version.
- Heijden, I. van der (2014). What works to prevent violence against women with disabilities.
- Hughes, K., Bellis, M. A., Jones, L., Wood, S., Bates, G., Eckley, L., et al. (2012). Prevalence and risk of violence against adults with disabilities: a systematic review and meta-analysis of observation-al studies. Lancet, 379(9826), 1621-1629.
- Hughes, R. B., Lund, E. M., Gabrielli, J., Powers, L. E., & Curry, M. A. (2011). Prevalence of interper-sonal violence against community-living adults with disabilities: a literature review. Rehabilitation Psy-chology, 56(4), 302-319.
- Jones, L., Bellis, M. A., Wood, S., Hughes, K., McCoy, E., Eckley, L., et al. (2012). Prevalence and risk of violence against children with disabilities: a systematic review and meta-analysis of observa-tional studies. The Lancet, 380(9845), 899-907.
- Khemka, I., Hickson, L., Reynolds, G. (2005). Evaluation of a Decision-Making Curriculum Designed to Empower Women With Mental Retardation to Resist Abuse. American Journal On Mental Retardation, Volume 110, Number 3: 193–204, May 2005.
- Krnjacki, L., Emerson, E., Llewellyn, G., Kavanagh, A.M. (2016). Prevalence and risk of violence against people with and without disabilities: findings from an Australian population-based study. Australian and New Zealand Journal of Public Health, 40:16-21.
- Marsland, D., Oakes, P., & White, C. (2007). Abuse in care? The identification of early indicators of the abuse of people with learning disabilities in residential settings. The Journal of Adult Protection, 9(4), 6-20.
- Platt, L., Powers, L., Leotti, S., Hughes, R.B., Robinson, Whelen, S., Osborn, S. Ashkenazy, E., Beers, L., Lund, E. Nicolaidis, Ch., Partnering With People With Disabilities to Address Violence
- Consortium (2017). The Role of Gender in Violence Experienced by Adults With Developmental Disa-bilities. Journal of Interpersonal Violence, Vol. 32(1) 101–129.
- Plummer, S.B., Findley, P.A. (2012). Women With Disabilities' Experience With Physical and Sexual Abuse: Review of the Literature and Implications for the Field. Trauma Violence Abuse, 13(1) 15-29
- Robinson-Whelen, S., Hughes, R.B., Gabrielli, J., Lund, E.M., Abramson, W., Swank, P.R. (2014)
- Sobsey, D. (2005). Violence & disability. In: W. M. Nehring (Ed.), Health promotion for persons with intellectual/developmental disabilities: The state of scientific evidence. Washington, DC: American Association on Mental Retardation.
- Strand, M.L., Benzein, E., Saveman, B. (2004). Violence in the care of adult persons with intellectual disabilities. Journal of Clinical Nursing, 13, 506–514
- Trevellion, K., Oram, S., Feder, G., Howard, L.M. (2012). Experiences of Domestic Violence and Men-tal Disorders: A Systematic Review and Meta-Analysis. PLOS ONE, December, Volume 7 Issue 12
