# Supplementary material for: Lesser-known types of violence: Helping nurses and midwives to signal and act
Source: Int J Nurs Stud Adv. 2022 Sep 17;4:100098. doi: 10.1016/j.ijnsa.2022.100098 (PMC11080451; doi:10.1016/j.ijnsa.2022.100098)
Supplement: Supplementary file 1 [file mmc1.zip › Factsheets Dutch/Geweld-tegen-migranten.pdf]

GEWELD BIJ MIGRANTEN IN KWETSBARE SITUATIES

GEBRUIK BIJ ELKE VORM VAN HUISELIJK GEWELD EN KINDERMISHANDELING DE MELDCODE! ▶

WAAROM EEN APARTE FACTSHEET OVER MIGRANTEN IN KWETSBARE SITUATIES?

Migranten is de verzamelnaam voor mensen die om verschillende redenen vanuit het buitenland naar Nederland zijn verhuisd om zich hier tijdelijk of permanent te vestigen. Deze factsheet gaat specifiek over migranten in kwetsbare situaties die een verhoogd risico lopen om slachtoffer te worden van huiselijk geweld en waarbij het stoppen van de relatie gevolgen kan hebben voor hun verblijfsrechtelijke situatie.

NB Deze factsheet gaat niet over slachtoffers mensenhandel, daar is een aparte factsheet over.

Hieronder worden de specifieke kenmerken genoemd van een aantal groepen migranten ín kwetsbare situaties – de groepen waar deze factsheet over gaat.

Categorie	Kenmerken
Asielzoekers	Zijn in afwachting van een beslissing op aanvraag voor verblijfsvergunning en verblijven in een asielzoekerscentrum (AZC). COA personeel is getraind in signaleren van huiselijk geweld en zij kunnen ook hulp inschakelen van Veilig Thuis en vrouwenopvang.
Statushouders	Hebben een verblijfvergunning ontvangen en een woning toegewezen gekregen of verblijven nog in een AZC in afwachting van toewijzing van een woning. Bij statushouders kunnen gezinsleden nareizen in het kader van gezinshereniging. Die gezinsleden krijgen een zelfstandig verblijfsvergunning.
Huwelijksmigranten / Partnermigranten	Wonen bij de partner en hebben van die partner afhankelijke verblijfsvergunning. Pas na 5 jaar kunnen zij een onafhankelijke vergunning krijgen.
Europeanen	Kunnen hier zijn als partnermigrant of hier zijn om werken.
Ongedocumenteerden	Het kan gaan om migranten die illegaal Nederland binnenkwamen, migranten die langer bleven dan hun visum toeliet, afgewezen asielzoekers, of migranten die bleven nadat hun verblijfsrecht op andere gronden was beëindigd. Ze zijn grotendeels onzichtbaar, erg kwetsbaar en afhankelijk van degene die hen onderdak en eten geeft. Als zij in beeld komen bij de overheid lopen zij risico om opgepakt en uitgezet te worden. Daarom zijn ze bang voor contact met formele instanties en kennen hun rechten veelal niet. Ze hebben als slachtoffer van huiselijk geweld op grond van internationale verdragen wel recht op bescherming, medische zorg, hulp en opvang.

FEITEN EN CIJFERS

In Nederland verblijven, naar schatting van het WODC in 2015 ongeveer 35.000 mensen zonder verblijfsvergunning, waarvan een derde vrouw en de helft met een afgewezen asielverzoek. Het aantal slachtoffers huiselijk geweld zonder verblijfsvergunning zou volgens Significant (zie Bronnen) tussen de 70 en 800 per jaar kunnen liggen. Jaarlijks worden zo'n 30-40 slachtoffers van huiselijk geweld zonder verblijfsvergunning opgevangen door opvangorganisaties.

MEER INFORMATIE

Zie de bronnen en:

- Folder IND over verblijfsrecht voor slachtoffers van geweld in afhankelijkheidsrelaties:
- Informatie over vrouwenopvang en mannenopvang: www.opvang.nl, www.opvangatlas.nl, www.mannen-opvang.nl
- Over rechten van mensen zonder verblijfsvergunning: www.basicrights.nl, www.stichtinglos.nl of www.iLegale-vrouw.nl
- Informatie over opvang asielzoekers

Deze factsheet is ontwikkeld door de Federatie Opvang en de Stichting LOS i.s.m. diverse ketenpartners.

november 2018

GEWELD BIJ MIGRANTEN IN KWETSBARE SITUATIES

HUISELIJK GEWELD BIJ MIGRANTEN IN KWETSBARE SITUATIES

Huiselijk geweld is geweld dat wordt gepleegd door iemand uit de huiselijke of familiekring. Het gaat om geweld in een afhankelijkheidsrelatie. Die afhankelijkheid maakt het voor het slachtoffer vaak lastig om het geweld te stoppen of er aan te ontkomen. Bij migranten in kwetsbare situaties is vaak sprake van een vergrote mate van afhankelijkheid, zie onder risicofactoren.

Hulp en opvang voor slachtoffers huiselijk geweld moet, volgens internationaal recht (Istanbul verdrag en Europese richtlijn voor slachtoffers) toegankelijk zijn, onafhankelijk van de verblijfsrechtelijke status.

Huiselijk geweld en daaropvolgende scheiding kan gevolgen hebben voor het verblijfsrecht:

Categorie	Gevolgen voor verblijfsrecht
Statushouders	Vluchtelingen en eventuele nareizende gezinsleden houden hun asielstatus ook na scheiding.
Huwelijksmigranten/ Partnermigranten	In geval van huiselijk geweld krijgt de afhankelijke partner een tijdelijke vergunning. Die wordt alleen verlengd als terugkeer een risico op mensenrechtenschending in het herkomstland zou betekenen.
Asielzoekers	Als het vluchtverhaal gebonden is aan één van de twee partners, kan in geval van scheiding de andere partner mogelijk geen asielvergunning meer krijgen. Als scheiding in het herkomstland leidt tot een risico op mensenrechtenschending, kan dat wel een zelfstandige reden voor een asielvergunning zijn.
Europeanen	Mensen uit EU landen kunnen in Nederland blijven als ze eigen middelen hebben van bestaan. Contact met IND is van belang voor Europeanen die een beroep doen op een uitkering en of opvangvoorzieningen.
Ongedocumenteerden	Ongedocumenteerden kunnen een vergunning krijgen als slachtoffer van huiselijk geweld indien terugkeer een risico op mensenrechtenschending in het herkomstland zou betekenen.

ADVIES/MELDEN

Voor advies, melden en/of doorverwijzing naar opvang en/of andere hulp, bel:

- Veilig Thuis **0800 20 00**

Bij acuut gevaar bel **112**

Voor advies over ongedocumenteerden, hun rechten en adviezen over beschikbare hulpverlening, bel:

- de Stichting LOS **010 74 70 156**

ENGELSE VERTALING

Zie hier.

Deze factsheet is ontwikkeld door de Federatie Opvang en de Stichting LOS i.s.m. diverse ketenpartners.

GEWELD BIJ MIGRANTEN IN KWETSBARE SITUATIES

SIGNALEN: HOE KAN IK ZIEN DAT IEMAND SLACHTOFFER IS?

In principe zijn de signalen bij migranten in kwetsbare situaties dezelfde als bij mensen met een Nederlandse achtergrond. Het is echter bij migranten extra belangrijk om goed oog te hebben voor signalen omdat die vaker verborgen blijven. Zeker bij degenen, met name ongedocumenteerden, die geïsoleerd leven, die bang zijn voor instanties en vanuit hun cultuur niet makkelijk open zijn over problemen.

RISICOFACTOREN: WIE HEEFT MEER KANS OM SLACHTOFFER TE ZIJN?

Bij migranten in kwetsbare situaties kan door verschillende omstandigheden sprake zijn van een verhoogd risico op ontstaan en voortbestaan van huiselijk geweld:

- Onzeker verblijfsrecht kan mensen extra afhankelijk maken van partner of familie en kan mensen terughoudend maken om open te zijn naar instanties. Dit geldt extra voor mensen zonder verblijfsrecht.
- Economische en sociaal-maatschappelijke afhankelijkheid kan groot zijn als men financieel niet onafhankelijk is, laagopgeleid is en/of de taal niet spreekt.
- Culturele aspecten kunnen een rol spelen, bijvoorbeeld de man-vrouw verhoudingen en/of terughoudendheid om met problemen naar instanties te gaan, onbekendheid met instanties en/of schaamte over de situatie.
- Onbekendheid met het Nederlandse stelsel van zorg en welzijn.
- Migranten hebben veelal een beperkter netwerk en missen daardoor de beschermende factor die daarvan uit kan gaan.

AANDACHTSPUNTEN VOOR DIT TYPE GEWELD BIJ HET DOORLOPEN VAN DE 5 STAPPEN IN DE MELDCODE

Bij elke vorm van huiselijk geweld en kindermishandeling dien je als professional de meldcode te gebruiken. Algemene meldcode richtlijnen (zoals de 5 stappen) staan niet op deze factsheet beschreven – bezoek daarvoor de link. Wél staan hier aandachtspunten specifiek voor deze vorm van geweld:

- Probeer het slachtoffer alleen te spreken
- Schakel een tolk in als het slachtoffer de Nederlandse taal onvoldoende beheerst.
- Migranten met een onzeker verblijfsrecht en zeker ongedocumenteerden zijn vaak bang voor instanties en zeker voor de politie
- Bespreek goed door wat de mogelijkheden zijn om weg te gaan uit de situatie van geweld en wat de eventuele consequenties kunnen zijn voor het verblijfsrecht. Bij Meer informatie en Advies/ melden op de vorige 2 pagina's staat waar hierover informatie is te vinden.
- Vaak is na melding of aangifte opvang nodig. Belangrijk om dit direct in gang te zetten.

Deze factsheet is ontwikkeld door de Federatie Opvang en de Stichting LOS i.s.m. diverse ketenpartners.
