# Supplementary material for: Lesser-known types of violence: Helping nurses and midwives to signal and act
Source: Int J Nurs Stud Adv. 2022 Sep 17;4:100098. doi: 10.1016/j.ijnsa.2022.100098 (PMC11080451; doi:10.1016/j.ijnsa.2022.100098)
Supplement: Supplementary file 1 [file mmc1.zip › Factsheets Dutch/Geweld-tegen-migranten-bronnen.pdf]

BRONNEN GEWELD BIJ MIGRANTEN IN KWETSBARE SITUATIES

Dit bestand geeft een overzicht van organisaties die betrokken zijn geweest bij de ontwikkeling van de bijbehorende factsheet en van beschikbare achtergrondinformatie (bronnen)

BETROKKEN ORGANISATIES

In het maken van deze factsheet over huiselijk geweld tegen/bij migranten in kwetsbare situaties voor professionals in alle beroepen die een meldcode huiselijk geweld en kindermishandeling hanteren, hebben de volgende organisaties input geleverd:

- Federatie Opvang en de Stichting LOS. Voor vragen en/of opmerkingen over de factsheet, kunt u emailen met de auteurs: Liesbeth van Bemmel, l.vanbemmel@opvang.nl , en Rian Ederveen, rian.ederveen@stichtinglos.nl

BRONNEN

De volgende documenten en informatiebronnen geven meer informatie over de signalen van huiselijk geweld tegen/bij migranten in kwetsbare situaties, risicofactoren, en dingen om op te letten bij het doorlopen van de 5 stappen van de meldcode huiselijk geweld en kindermishandeling:

- Significant: Toegang tot de opvang van slachtoffers zonder eerdere verblijfsstatus, 3.11.17
- Ombudsman : Vrouwen in de knel (rapport 2017/075), 7.7.17
- KIS: Huiselijk geweld en veiligheid in asielopvangcentra
- Keygnaert, I. et al: Hidden Violence is a Silent Rape: Prevention of Sexual & Gender-Based Violence against Refugees & Asylum Seekers in Europe: a Participatory Approach Report. ICRH,Ugent, Ghent, 2008
- IND : Evaluatie gendergerelateerd vreemdelingenbeleid in Nederland, 2008
- WODC: Schattingen illegaal in Nederland verblijvende vreemdelingen 2012-2013, 2015
