# Supplementary material for: Lesser-known types of violence: Helping nurses and midwives to signal and act
Source: Int J Nurs Stud Adv. 2022 Sep 17;4:100098. doi: 10.1016/j.ijnsa.2022.100098 (PMC11080451; doi:10.1016/j.ijnsa.2022.100098)
Supplement: Supplementary file 1 [file mmc1.zip › Factsheets Dutch/grensoverschrijdend-gedrag-jongeren.pdf]

SEKSUEEL GRENSOVERSCHRIJDEND GEDRAG TUSSEN JONGEREN

WAT IS SEKSUEEL GRENSOVERSCHRIJDEND GEDRAG?

Bij seksueel grensoverschrijdend gedrag gaat het om gedrag of toenaderingen die seksueel van aard zijn en de grenzen van het slachtoffer overschrijden. Dit kan al dan niet fysiek zijn (De Haas, 2012). Om te bepalen of seksueel gedrag al dan niet grensoverschrijdend is, gaan we uit van een aantal fundamentele criteria of maatschappelijke waarden, gebaseerd op rechten, wetgeving en regelgeving. Er wordt hierbij uitgegaan van diegene die verantwoordelijk is voor het gedrag (Frans, De Wilde, Janssens, van Berlo & Storms, 2016): (1) wederzijdse toestemming; (2) vrijwilligheid; (3) gelijkwaardigheid; (4) passend voor de leeftijd of ontwikkeling of leeftijd; (5) passend bij de context; (6) zelfrespect (zie ook het Sensoa Vlaggensysteem© , Frans & Franck; 2010; 2014). Bij positief en acceptabel seksueel gedrag van kinderen en jongeren wordt aan alle criteria voldaan. Wanneer er aan één of meerdere van de criteria niet wordt voldaan is er sprake van seksueel grensoverschrijdend gedrag. We spreken van seksueel misbruik voor elke vorm van seksueel grensoverschrijdend gedrag in verbale of fysieke zin, opzettelijk of onopzettelijk, waar duidelijk geen wederzijdse toestemming voor bestaat; en/of dat op één of andere manier afgedwongen is en/of waarbij het slachtoffer veel jonger is of in een afhankelijke relatie staat (Frans et a., 2016; Frans & Franck, 2014).

HANDS-OFF EN HANDS-ON

Bij seksuele grensoverschrijdingen onderscheiden we zogenoemd hands-off en hands-on gedrag. Als er sprake is van strafbaar gedrag spreken we van een delict. Bij hands-off gedrag is er geen sprake van fysiek contact en betreft het bijvoorbeeld kwetsende seksuele opmerkingen of sexting (het maken en versturen van seksueel getinte berichten of pikante foto's en video's) of gedwongen worden te kijken naar porno. Bij grensoverschrijdend gedrag dat 'hands-on' plaatsvindt, is er wel sprake van fysiek contact. Dit betreft het seksueel betasten, het binnendringen, variërend van zoenen (gedwongen tongzoen) tot verkrachting, de ernstigste vorm van seksueel grensoverschrijdend gedrag.

JONGEREN EXPERIMENTEREN EN GAAN SOMS OVER GRENZEN

Veel seksuele grensoverschrijdingen bij jongeren vinden plaats door leeftijdsgenoten. Jongeren zijn volop in ontwikkeling; daar hoort experimenteergedrag bij. En soms gaan ze over hun eigen grenzen of die van een ander. Jongeren zijn zich soms onvoldoende bewust van de grenzen van zichzelf en van een ander, ze weten niet hoe ze dit bij een ander moeten herkennen of ze vinden het moeilijk om hardop uit te spreken wat ze wel of niet prettig vinden. Ook groepsdruk kan hierbij een rol spelen; en/of er kan sprake zijn van de invloed van alcohol of andere drugs. (Professionele) opvoeders hebben dan ook de taak jongeren hierin te begeleiden, en jongeren bewust te maken van wensen en grenzen op seksueel gebied. Wanneer het gaat om seks met een minderjarige dan geldt het strafrecht (zie onder).

RISICOGROEPEN

Risicogroepen voor seksuele grensoverschrijding zijn onder meer:

- Meisjes lopen over het algemeen meer risico dan jongens op seksueel grensoverschrijdend gedrag.
- Lesbische, homoseksuele, biseksuele of transgenderjongeren.

CIJFERS

Uit het onderzoek Seks onder je 25e van Rutgers (De Graaf et al., 2017) blijkt dat 2% van de jongens en 11% van de meisjes wel eens gedwongen zijn tot seksuele dingen die de betreffende jongere niet wilde.

ADVIES / MELDEN

- Centrum Seksueel Geweld: Het Centrum Seksueel Geweld biedt medische, forensische en psychologische hulp aan slachtoffers die kort geleden (minder dan zeven dagen) een aanranding of een verkrachting hebben meegemaakt. Bel **0800-0188**.
- Veilig Thuis: Als het seksueel geweld langer geleden heeft plaatsgevonden: neem contact op met Veilig Thuis. Doe dit ook in situaties van grensoverschrijdend gedrag waarin de veiligheid van de betrokkenen in het geding is, zodat niet alleen hulpverlening geboden kan worden maar ook de veiligheid in het systeem geborgd kan worden, ook voor broertjes en zusjes. **0800 – 2000** (gratis).
- Bij acuut gevaar, neem contact op met de politie, bel **112**.

Deze factsheet is ontwikkeld door Movisie i.s.m. diverse ketenpartners. november 2018

SEKSUEEL GRENSOVERSCHRIJDEND GEDRAG TUSSEN JONGEREN

- Laagopgeleide jongeren.
- Jongeren met een beperking (fysiek of verstandelijk, ook een lichte verstandelijke beperking).
- Jongeren die in een instelling voor jeugdzorg verblijven.
- Jongeren met negatieve jeugdervaringen met zowel fysieke mishandeling, emotionele verwaarlozing als seksueel misbruik.

RISICOFACTOREN

Naast de bovengenoemde risicogroepen zijn er ook *individuele en relationele risicofactoren*. Dit zijn onder meer gebrek aan kennis en vaardigheden bij kinderen en jongeren om seksueel gedrag te beoordelen, geen vertrouwensrelatie met de ouders hebben, en eerdere ervaringen hebben met seksueel grensoverschrijdend gedrag in het verleden, de zogenoemde revictimisatie. Ook jongeren met een laag zelfbeeld, ADHD, of een autismespectrumstoornis waardoor ze onvoldoende kunnen invoelen wat een ander beleeft, lopen meer risico.

Maatschappelijke gegevenheden en ontwikkelingen die risico's met zich meebrengen zijn onder meer het hebben van traditionele rolopvattingen over mannen en vrouwen, genderstereotypen, een dubbele moraal en een veranderende realiteit: seksualiteit komt meer voor in massamedia (Römkens, 2017), en de 'biologische rijping' start vroeger bij kinderen/jongeren dan in het verleden het geval was (Storms & Doornink, 2016). Ook het toegenomen experimenteergedrag en alcohol- en drugsgebruik bij jongeren vallen hieronder.

HANDVATTEN VOOR DE PROFESSIONAL

Het Sensoa Vlaggensysteem© (Frans & Franck, 2010; 2014) biedt professionele opvoeders handvatten om seksueel gedrag adequaat te beoordelen, het bespreekbaar te maken en om gepast te reageren. Dit gebeurt aan de hand van zes criteria om te bepalen of het gedrag gezond of grensoverschrijdend is: toestemming, vrijwilligheid, gelijkwaardigheid, leeftijds- of ontwikkelingsadequaat , contextadequaat en zelfrespect. Er is specifieke aandacht voor gender- en cultuuraspecten; kinderen en jongeren met een beperking en/of trauma (Frans et al., 2016).

WAT ZEGT DE WET?

Seks met een minderjarige is in principe strafbaar, ook als het contact door beiden wordt gewenst. Tussen 12 en 16 jaar geldt echter dat het Openbaar Ministerie de jongere vraagt om zijn of haar mening, dit 'zo mogelijk' en in bepaalde gevallen (art. 167a Sv). Een jongen van 17 jaar die seksueel contact heeft met een meisje van 15 dat daar geen bezwaar tegen heeft, zal hoogstwaarschijnlijk geen problemen krijgen met justitie. Seks met een kind jonger dan 12 is altijd strafbaar.

MEER INFORMATIE

Zie de bronnen en deze websites:
- www.act4respect.nl (wordt eind 2018 gelanceerd)
- www.rutgers.nl
- www.atria.nl
- www.vlaggensysteem.nl

ENGELSE VERTALING

Zie hier.
