# Supplementary material for: Lesser-known types of violence: Helping nurses and midwives to signal and act
Source: Int J Nurs Stud Adv. 2022 Sep 17;4:100098. doi: 10.1016/j.ijnsa.2022.100098 (PMC11080451; doi:10.1016/j.ijnsa.2022.100098)
Supplement: Supplementary file 1 [file mmc1.zip › Factsheets Dutch/grensoverschrijdend-gedrag-jongeren-bronnen.pdf]

BRONNEN SEKSUEEL GRENSOVERSCHRIJDEND GEDRAG TUSSEN JONGEREN

Bij alle vormen van huiselijk geweld en kindermishandeling moet de meldcode huiselijk geweld en kindermishandeling volgens de wet toegepast worden door de groepen professionals die in de wet over de meldcode staan benoemd. Seksueel grensoverschrijdend gedrag tussen jongeren valt niet onder de definitie van huiselijk geweld of kindermishandeling en de meldcode hoeft hierbij dus niet toegepast te worden. Echter, de meldcode mag hier wel bij gebruikt worden! En omdat het belangrijk is dat professionals (bijv docenten of huisartsen) seksueel grensoverschrijdend gedrag tussen jongeren wel kunnen signaleren en de juiste stappen kunnen nemen, is de factsheet die hoort bij dit bronnenbestand opgesteld.

Dit bestand geeft een overzicht van organisaties die betrokken zijn geweest bij de ontwikkeling van de factsheet en van beschikbare achtergrondinformatie (bronnen).

BETROKKEN ORGANISATIES

In het maken van deze factsheet over seksueel grensoverschrijdend gedrag tussen jongeren hebben de volgende organisaties input geleverd:

- Movisie. Voor vragen en/of opmerkingen over de factsheet, kunt u emailen met de hoofdauteur: Wilma Schakenraad, w.schakenraad@movisie.nl
- Veilig Thuis
- Kennisinstituut voor Emancipatie en Vrouwengeschiedenis (Atria)

BRONNEN

De volgende documenten en informatiebronnen geven meer informatie over de signalen van seksueel grensoverschrijdend gedrag tussen jongeren, risicofactoren, en dingen om op te letten:

Publicaties

- Berlo van, W. & Beek I. van (2015). Whitepaper Seksuele grensoverschrijding en seksueel geweld. Feiten en cijfers Utrecht: Rutgers en Movisie.
- Frans, E., De Wilde, K., Janssens, K., Van Berlo, W., & Storms, O. (2016). Buiten de lijnen. Sensoa Vlaggensysteem voor kinderen en jongeren met bijzondere behoeften. Antwerpen/Apeldoorn: Garant Uitgevers.
- Frans, E., & Franck, T. (2010, 3e dr. 2014). Sensoa Vlaggensysteem. Praten met kinderen en jongeren over seks en seksueel grensoverschrijdend gedrag. Antwerpen/Apeldoorn: Garant Uitgevers.
- Graaf, H. de, Borne, M. van den, Nikkelen, S., Twisk, D., & Meijer, S. (2017). Seks onder je 25e. Utrecht / Amsterdam: Rutgers / Soa Aids Nederland.
- Haas, S. de (2012). Seksueel grensoverschrijdend gedrag onder jongeren en volwassenen in Nederland. Tijdschrift voor Seksuologie, 36-2, p. 136-145.
- Höing, M., & Janssen, J. (2017). Seksueel grensoverschrijdend gedrag. In: Höing, M., & Janssen, J., Boer, A., & Liebregts, M. (red.). Bespreekbaar maken van seksualiteit en intimiteit. Handboek voor professionals in zorg en welzijn. Bussum: Coutinho.
- Nationaal Rapporteur Mensenhandel en Seksueel Geweld tegen Kinderen (2014). Op goede grond. De aanpak van seksueel geweld tegen kinderen. Den Haag: Nationaal Rapporteur.
- Römkens, R. (2017). Factsheet Online seksuele intimidatie. Amsterdam: Atria. Zie: www.atria.nl/sites/atria/files/atoms/files/factsheet-cybergeweld-onlineversiedef.pdf
- Storms, O. & Doornink, N. (2016). Vlaggensysteem: Reageren op seksueel (grensoverschrijdend) gedrag van kinderen en jongeren. Effectieve sociale interventies en Effectieve interventies huiselijk en seksueel geweld. Utrecht: Movisie.

Websites

- http://seksonderje25e.nl (Rutgers)
- Kennisdossier seksuele grensoverschrijding van Rutgers: https://www.rutgers.nl/feiten-en-cijfers/kennisdossiers/kennisdossier-seksuele-grensoverschrijding
- www.seksueelgeweld.info/ Website voor slachtoffers van seksueel geweld, en voor betrokkenen en verwijzers. Zie hierop ook de sociale kaart met hulpaanbod voor slachtoffers en plegers van seksueel geweld.
- www.vlaggensysteem.nl
- www.act4respect.nl (wordt eind 2018 gelanceerd)
- www.atria.nl
