# Supplementary material for: Lesser-known types of violence: Helping nurses and midwives to signal and act
Source: Int J Nurs Stud Adv. 2022 Sep 17;4:100098. doi: 10.1016/j.ijnsa.2022.100098 (PMC11080451; doi:10.1016/j.ijnsa.2022.100098)
Supplement: Supplementary file 1 [file mmc1.zip › Factsheets Dutch/huiselijk-geweld-tegen-mannen.pdf]

HUISELIJK GEWELD TEGEN MANNEN

WAT IS HUISELIJK GEWELD JEGENS MANNEN OFTEWEL MANNENMISHANDELING?

Mannenmishandeling is een vorm van partnergeweld, het betreft huiselijk geweld jegens mannen, zie de factsheet (ex)-partnergeweld. Geschat wordt dat in 40% van de huiselijk geweld casuïstiek, er (ook) sprake is van mannenmishandeling. Er worden ongeveer 80.000 mannen per jaar ernstig mishandeld in huiselijke kring.

Huiselijk geweld gericht op mannen heeft veel overeenkomsten met huiselijk geweld zoals beschreven in de factsheet (ex)-partnergeweld.

Er zijn ook verschillen, waarmee bij de signalering en aanpak rekening gehouden moet worden:

- De beleving van de mannen. Mannenmishandeling wordt door de mannen die dit betreft niet altijd gezien als een probleem, noch als een vorm van huiselijk geweld. Verder hebben deze mannen vaak het idee dat er weinig tot geen lotgenoten zijn oftewel dat zij de enige zijn dit meemaken. Een feit is dat slecht 3% van deze mannen zich meldt bij de politie.
- Maatschappelijke beelden en normen. Denk hierbij aan denkbeelden als: "Een man laat zich niet slaan (en zeker niet door een vrouw)".
- De schaamte is groot. Hierdoor komen deze mannen minder snel naar voren, praten er minder over en vragen minder om hulp. Deze schaamte wordt ook gevoed door de eerder genoemde maatschappelijke beelden en normen.

AANDACHT VOOR DE SPECIFIEKE DOELGROEP: LHBTI+

Huiselijk geweld komt in alle relaties voor of deze nu heteroseksueel, homoseksueel, biseksueel of transgender van aard zijn. Ook op huiselijk geweld onder LHBTI+ heerst een groot taboe. Uit Brits onderzoek blijkt dat één op de vier homo's slachtoffer is van partnergeweld. Mensen hebben veelal het idee dat geweld in partnerrelaties van homo's en lesbiennes minder serieus is of minder ernstig is, omdat beide partners in kracht en macht ongeveer gelijk zijn aan elkaar ("de ander kan terugslaan of op een andere manier van zich afbijten"). Ook blijkt dat heteroseksuelen er vaak van uit gaan dat homo's en lesbiennes een relatie sneller zullen verbreken als er geweld in voorkomt. De meeste heteroseksuelen zien economische afhankelijkheid en het gezamenlijk grootbrengen van kinderen niet als kernbegrippen van homo- en lesbische relaties. Er zou daarom minder zijn wat de homoseksuele of lesbische partners aan elkaar bindt. Het tegendeel is waar. Uit onderzoek blijkt dat homoseksuele mannen jarenlang geweld in hun relatie verdragen, juist omdat zij streven naar een langdurige stabiele liefdesrelatie.

SIGNALEN

Bij mannenmishandeling zijn geen andere signalen dan beschreven in de factsheet (ex)-partnergeweld en de bijbehorende signalenlijsten .

RISICOFACTOREN

Een aantal factoren kunnen ervoor zorgen dat het risico op (herhaling van) huiselijk geweld jegens mannen toeneemt.

- Demografische factoren: dit gaat om de leeftijd, opvoeding, karakter, armoede en huisvesting. De kans op mishandeling is bijvoorbeeld groter als de sociaaleconomische status lager is.

ADVIES / MELDEN

Voor advies, melden of het regelen van opvang en/of andere hulp, bel:

- Veilig Thuis **0800 20 00**
  Dit is gratis en 24/7 bereikbaar. Bellen kan ook anoniem. Bellen kan ook alleen voor advies (zonder te melden).

Bij acuut gevaar bel **112**

In Nederland zijn er 6 opvanglocaties (40 plekken) voor mannen.

MEER INFORMATIE

Zie de bronnen en:

- www.mannenmishandeling.nl
- www.huiselijkgeweld.nl

Deze factsheet is ontwikkeld door Blijf Groep, Stichting Wende en Veilig Thuis, i.s.m. diverse ketenpartners.

HUISELIJK GEWELD TEGEN MANNEN

- Mannen die in hun jeugd getuige of slachtoffer zijn geweest van huiselijk geweld of kindermishandeling lopen een verhoogd risico om in volwassenheid opnieuw slachtoffer te worden.
- Dynamische risicofactoren: dit gaat om middelengebruik, psychische problemen, stress, relatie-gezinsproblemen. Financiële stress is een van de meest voorkomende reden dat er partnergeweld in huiselijke sfeer plaatsvindt. Migratiestress is ook een van de risicofactoren evenals wanneer een man vanuit het buitenland naar Nederland komt om te trouwen.
- Statische risicofactoren: dit gaat om persoonlijke problematiek van de man, zoals een licht verstandelijke beperking.
- Voorwaardelijke factoren zoals sociaal isolement, echtscheidingsproblematiek.
- De mate waarin geweld naar mannen wordt genormaliseerd en/of als minder bedreigend wordt geschetst vanuit maatschappij en/of media.
- Problematiek bij de partner van de man, te denken aan beperkte agressiebeheersing, verslaving, persoonlijkheidsproblematiek, moeite met communiceren of conflicthantering, jaloezie of neiging tot controleren.

AANDACHTSPUNTEN BIJ MANNENMISHANDELING BIJ HET DOORLOPEN VAN DE 5 STAPPEN IN DE MELDCODE.

Bij elke vorm van huiselijk geweld en kindermishandeling dien je als professional de meldcode en bijbehorende afwegingskaders te gebruiken. Dit geldt ook voor huiselijk geweld jegens mannen. De volgende aandachtspunten zijn specifiek voor deze vorm van geweld:

- Genderspecifieke presentatie. In het algemeen kan gesteld worden dat mannen vanuit genderspecifiek perspectief andere manieren hebben van hulp zoeken, van hun problemen presenteren, van omgaan met problemen. Ook is er een andere probleembeleving. Mannen zoeken pas hulp als ze er zelf niet meer uitkomen, zoeken zelf oplossingen en proberen deze uit, zoeken oorzaken van problemen buiten zichzelf, beschrijven problemen vanuit een toeschouwer positie, presenteren problemen in context van werk, verwoorden problemen in zaken en feiten
- De veroordelen en maatschappelijke normen. Ook de hulpverlener kan (onbewust, onbedoeld) van hieruit redeneren, wat de hulpverlening kan belemmeren.
- Het taboe. Hierdoor zullen de mannen zichzelf bv. niet snel als slachtoffer beschouwen.
- Overnemen van regie. Deze mannen hebben er veelal baat bij zelf zo veel regie als mogelijk te behouden. Heb ook begrip voor de wijze waarop deze mannen omgaan met de problemen.

Deze factsheet is ontwikkeld door Blijf Groep, Stichting Wende en Veilig Thuis, i.s.m. diverse ketenpartners.
