# Supplementary material for: Lesser-known types of violence: Helping nurses and midwives to signal and act
Source: Int J Nurs Stud Adv. 2022 Sep 17;4:100098. doi: 10.1016/j.ijnsa.2022.100098 (PMC11080451; doi:10.1016/j.ijnsa.2022.100098)
Supplement: Supplementary file 1 [file mmc1.zip › Factsheets Dutch/huiselijk-geweld-tegen-mannen-bronnen.pdf]

BRONNEN HUISELIJK GEWELD TEGEN MANNEN

Dit bestand geeft een overzicht van organisaties die betrokken zijn geweest bij de ontwikkeling van de bijbehorende factsheet en van beschikbare achtergrondinformatie (bronnen).

BETROKKEN ORGANISATIES

In het maken van deze factsheet over Huiselijk geweld tegen mannen voor professionals in alle beroepen die een meldcode huiselijk geweld en kindermishandeling hanteren, hebben de volgende organisaties input geleverd:

- Blijf Groep, Stichting Wende en Veilig Thuis. Voor vragen en/of opmerkingen over de factsheet, kunt u emailen met de hoofdauteurs: Claire Loeber, C.Loeber@blijfgroep.nl , Carla Scherpenhuijzen, c.scherpenhuijzen@perspektief.nl , Floor van Niekerk, en Ries Wilschut, RWilschut@samen-veilig.nl
- Robert Weinberg, Blijf groep

BRONNEN

De volgende documenten en informatiebronnen geven meer informatie over de signalen van Huiselijk geweld tegen mannen, risicofactoren, en dingen om op te letten bij dit type geweld bij het doorlopen van de 5 stappen van de meldcode huiselijk geweld en kindermishandeling:

- Nanhoe, A., *Pionieren in* de mannenopvang, Een evaluatiestudie na 2½ jaar ervaring met de opvang en hulpverlening aan mannelijk slachtoffers van (dreiging van) geweld in afhankelijkheidsrelaties in Amsterdam, Rotterdam, Den Haag en Utrecht, Rijksoverheid, Gemeente Amsterdam, Gemeente Rotterdam, Gemeente Den Haag, Gemeente Utrecht (Uitgevoerd door GGD Rotterdam-Rijnmond), 2011
- Adrie Vermeulen, Necla Kilic, Bert Visser, Magda Vogelezang, 'Als man heb je al gauw de schijn tegen' Beschrijving expertise Pilot G4 Mannenopvang Huiselijk geweld, Eergerelateerd geweld & Mensenhandel 2014
- Van Dijk, D., Hoekstra, L., & Nieuwenhout, Y. (2010). Als de nood aan de man is: Een verkennende studie naar de opvang van en hulpverlening aan mannelijke slachtoffers van (dreiging van) geweld in afhankelijkheidsrelaties. Rotterdam: Sociale Zaken en Werkgelegenheid.
- Janssen, J. H. L. J., & Sanberg, R. (2013). Als de nood aan de man komt. Slachtofferschap van mannen bij eergerelateerd geweld.
- Ligtenberg, D. (2018). Mijn Leven, geslagen man het taboe op mannenmishandeling. Libelle, 33, 74-78
- Oosten, van, N, Visser, A., Hazebroek, L., Daru, s. (2015). Dossier: wat werkt bij partnergeweld. Utrecht: Movisie.
- Renzetti, C. M., & Miley, C. H. (2014). Violence in gay and lesbian domestic partnerships. Routledge.
- Römkens, R. (2010). Omstreden gelijkheid. B. Den Haag, Huiselijk geweld, 11-32.
- Schuyf, J. (2009). Geweld tegen homoseksuele mannen en lesbische vrouwen. Movisie.
- Tsui, V., Cheung, M., & Leung, P. (2010). Help-seeking among male victims of partner abuse: men's hard times. Journal of community psychology, 38(6), 769-780.
- Yanez, P. C. (2018). A Treatment Model for Male Victims of Domestic Violence: A Support Group for Men with Abusive Partners (Doctoral dissertation, The Chicago School of Professional Psychology).
- www.mannenmishandeling.nl
- www.huiselijkgeweld.nl
- https://signalenkaart.nl/
