# Supplementary material for: Lesser-known types of violence: Helping nurses and midwives to signal and act
Source: Int J Nurs Stud Adv. 2022 Sep 17;4:100098. doi: 10.1016/j.ijnsa.2022.100098 (PMC11080451; doi:10.1016/j.ijnsa.2022.100098)
Supplement: Supplementary file 1 [file mmc1.zip › Factsheets Dutch/huwelijksdwang.pdf]

HUWELIJKSDWANG

GEBRUIK BIJ ELKE VORM VAN HUISELIJK GEWELD EN KINDERMISHANDELING DE MELDCODE! ▶

WAT IS EEN GEDWONGEN HUWELIJK?

Bij huwelijksdwang dwingen ouders, familie of de gemeenschap een meisje/vrouw of jongen/man tot een huwelijk. Eén of beide huwelijkspartner(s) hebben geen zeggenschap over de sluiting van het huwelijk en stemmen er niet mee in. Er is geen vrije partnerkeuze of ze moeten voor een bepaalde leeftijd trouwen. Het huwelijk kan formeel (wettig) of informeel zijn. Familieleden of de gemeenschap kunnen druk uitoefenen. Dit kan subtiel tot zeer dwingend zijn. Huwelijksdwang is een vorm van huiselijk geweld, mogelijk eergerelateerd en kan strafbaar zijn.

SIGNALEN: HOE KAN IK ZIEN DAT IEMAND SLACHTOFFER IS?

Voor de buitenwereld is huwelijksdwang meestal niet zichtbaar. Soms tonen slachtoffers licht verzet, maar uit angst, schaamte of loyaliteit aan de familie schikken zij zich meestal naar bij de keuze van ouders of gemeenschap. Deze signalen kunnen een indicatie zijn voor huwelijksdwang:

- Gedragsverandering: teruggetrokken, bang, boos, agressief
- Minder tijd doorbrengen met vrienden of vriendinnen
- Beantwoordt telefoon of berichten niet
- Opeens andere kleding
- Tekenen van mishandeling, geweld
- Zelfverwonding, suïcidepogingen, eetstoornis
- Vroege of ongewenste zwangerschap.

Signalen school

- Vaak (langdurig) afwezig
- Verzoek lang familiebezoek buitenland
- Onverwachte reis naar buitenland
- Wordt in de gaten gehouden
- Plotseling van school gehaald
- Geen buitenschoolse activiteiten
- Slechte schoolprestaties

Signalen werk

- Mag niet of niet flexibel werken
- Vaak (langdurig) afwezig
- Slechte prestaties
- Beperkte carrièremogelijkheden
- Wordt financieel gecontroleerd
- Inkomen afgenomen
- Wordt opgehaald van het werk

RISICOFACTOREN: WIE IS EXTRA KWETSBAAR VOOR DIT GEWELD?

Mogelijk huwelijksdwang als een slachtoffer in een of meer risicogroepen valt:

- Jonge vrouwen (vaker) en mannen uit een gesloten gemeenschap met traditionele denkbeelden over rol, positie en seksualiteit van meisjes
- Migratieachtergrond, eercultuur, orthodox religieus of hoger milieu
- Jonge vrouwen en mannen in een sterk afhankelijke positie, zonder netwerk buiten de familie of eigen inkomen
- Personen met een afhankelijke verblijfsvergunning.

AANDACHTSPUNTEN VOOR DIT TYPE GEWELD BIJ HET DOORLOPEN VAN DE 5 STAPPEN IN DE MELDCODE

Maak gebruik van de meldcode en de meldcode eergerelateerd geweld. Daarnaast:

- Vraag advies en raadpleeg altijd een deskundige. Het collectieve karakter van huwelijksdwang vraagt specifieke expertise.
- Neem direct contact op met de politie bij acute dreiging van de veiligheid.
- Voer veiligheidshalve het gesprek met de partner, ouders/familie na taxatie van risico's door een deskundige.
- Slachtoffer in het buitenland: voer i.v.m. de veiligheid geen gesprek met ouders, partner of familie.

MEER INFORMATIE

Zie de bronnen en de factsheet eergerelateerd geweld.

FEITEN EN CIJFERS

- Aantal slachtoffers wordt geschat tussen 338 en 957 per jaar. Dat is meer dan het aantal meldingen van 181 per jaar.
- Huwelijksdwang komt vooral voor bij jongeren tussen 16 en 25 jaar.
- Komt voor in verschillende etnische en religieuze gemeenschappen: Afghanistan, Bosnië, Bulgarije, Egypte, India, Indonesië, Irak, Iran, Koerdisch, Marokko, Pakistan, Polen, Somalië, Turkije, en Suriname/ Hindoestaans en onder Sikhs en Roma
- Vindt plaats binnen familietradities, omwille van belangen of probleemoplossing.

ADVIES / MELDEN

Voor advies, melden en/of doorverwijzing naar opvang en/of andere hulp, bel:

- Veilig Thuis **0800 20 00**
- Landelijk Knooppunt Huwelijksdwang en Achterlating (LKHA) **070 34 54 319**
- Bij acuut gevaar bel **112**.

Als het slachtoffer in het buitenland is neem direct contact op met het LKHA. Jongeren kunnen anoniem chatten en Eva en Zahir zijn landelijke expertise- en behandelcentra.

ENGELSE VERTALING

Zie hier.

Deze factsheet is ontwikkeld door het Landelijk Knooppunt Huwelijksdwang en Achterlating i.s.m. diverse ketenpartners.

november 2018
