# Supplementary material for: Lesser-known types of violence: Helping nurses and midwives to signal and act
Source: Int J Nurs Stud Adv. 2022 Sep 17;4:100098. doi: 10.1016/j.ijnsa.2022.100098 (PMC11080451; doi:10.1016/j.ijnsa.2022.100098)
Supplement: Supplementary file 1 [file mmc1.zip › Factsheets Dutch/jongensprostitutie.pdf]

JONGENSPROSTITUTIE

WAT IS JONGENSPROSTITUTIE?

Jongensprostitutie is een vorm van jeugdprostitutie. Aangezien er voor meisjes (zie factsheet meisjesprostitutie) en jongens andere adviezen geleden is er voor gekozen jeugdprostitutie op te delen in twee factsheets. In Nederland verstaat men onder jongensprostitutie het verrichten van seksuele diensten in ruil voor een persoonlijk voordeel op het financiële of materiele vlak, door mannen of jongens. Prostitutie is legaal in Nederland.
In de volgende gevallen is jongensprostitutie strafbaar (deze factsheet gaat over die gevallen):

- Het kopen van seks van iemand jonger dan 18 (jeugdprostitutie).
- Iemand (18-/18+) die door een ander wordt gedwongen om in de prostitutie te werken (mensenhandel).

SIGNALEN: HOE KAN IK ZIEN DAT IEMAND JONGENSPROSTITUEE IS?

- Vage uitleg voor verwondingen
- Trekt zich terug
- Stiller/gesloten
- Isolatie van familie / vrienden + afhankelijkheid van een ander
- Plotseling veel geld / andere kleding
- Afpersing (bijv. met filmpjes)
- Rondgereden, gehaald en gebracht worden in auto (door nieuw / onbekend persoon)
- Psychosomatische klachten
- Vertoont sociaal wenselijk gedrag
- Hebben geheimen
- Fel "Anti homoseksueel"/plotseling erg "openlijk" homoseksueel
- Bedreiging (van het slachtoffer zelf of van familieleden)
- Drug/alcohol verslaving: meer middelen misbruik dan bij meisjes
- Heeft meerdere telefoons
- Veel gebeld worden (controle)
- Aanwezig op locatie die geassocieerd kan worden met prostitutie
- Hebben van anale klachten/ andere lichamelijke klachten die vaag worden uitgelegd
- Gedragsveranderingen: heeft totaal ander gedrag op verschillende plekken (school/vrienden/thuis)
- Dag en nachtritme is verstoord
- Concentratie problemen
- Verslechterende schoolprestaties

RISICOFACTOREN: WIE IS EXTRA KWETSBAAR?

Faseproblematiek (Puberteit), identiteitsproblematiek, zeer beïnvloedbare jongens/LVB, getraumatiseerde jongens, uit multi-problem gezinnen, niet-westerse-migratie-achtergrond (eergerelateerd geweld), (uitgeprocedeerde) asielzoekers, jonge leeftijd en het leven in twee werelden (zie "Jongensprostitutie in beeld").

AANDACHTSPUNTEN VOOR DIT TYPE GEWELD BIJ HET DOORLOPEN VAN DE 5 STAPPEN IN DE MELDCODE

Bij elke vorm van huiselijk geweld en kindermishandeling dien je als professional de meldcode te gebruiken (zie deze link). Algemene meldcode richtlijnen (zoals de 5 stappen) staan niet op deze factsheet beschreven – bezoek daarvoor de link. Wél staan hier aandachtspunten *specifiek voor deze vorm* van geweld:

- Niet alle "werkzaamheden" gebeuren vrijwillig. Bij seksuele uitbuiting kunnen de daders (mensenhandelaren) familieleden, vrienden of partners zijn
- Spreek iemand alleen!
- Let extra op eigen veiligheid, die van het slachtoffer en diens familie
- Buitenlandse slachtoffers hebben vaak weinig kennis van hun rechten en van de betrouwbaarheid van de politie
- De jongens ervaren niet altijd erkenning voor hun slachtofferschap en kunnen zichzelf niet altijd te zien als slachtoffer. Zij hebben behoefte aan een speciale benadering die niet veroordelend of stigmatiserend is.

FEITEN EN CIJFERS

- Er zijn geen precieze cijfers omtrent het aantal jongensprostituees. Geschat wordt dat er 1500 jongens dagelijks "werken".
- Taboes (op homoseksualiteit, op betaalde seks en op slachtofferschap bij jongens) maken het voor jongens moeilijker om hulp te vragen.
- Mannelijke slachtoffers van uitbuiting maken vaker de gang naar daderschap (vanuit slachtofferschap).
- Jongere jongens (14-16jr) worden meer gevraagd dan oudere(18+).
- Niet alle jongensprostituees zijn homoseksueel.
- Jongensprosituees hebben relatief meer kans op SOA's!

Zie voor betrokken organisaties www.wegwijzermensenhandel.nl

ADVIES / MELDEN

- Voor advies, melden of het regelen van opvang en/of andere hulp, bel:
- Veilig Thuis 0800 20 00
- CoMensha 033 44 81 186

een meldpunt loverboys
Bij acuut gevaar bel 112

ENGELSE VERTALING

Zie hier.

MEER INFORMATIE

Zie "Jongensprostitutie in beeld" en de bronnen

Deze factsheet is ontwikkeld door het Expertisecentrum Mensenhandel en (Jeugd)Prostitutie i.s.m. diverse ketenpartners.

december 2018
