# Supplementary material for: Lesser-known types of violence: Helping nurses and midwives to signal and act
Source: Int J Nurs Stud Adv. 2022 Sep 17;4:100098. doi: 10.1016/j.ijnsa.2022.100098 (PMC11080451; doi:10.1016/j.ijnsa.2022.100098)
Supplement: Supplementary file 1 [file mmc1.zip › Factsheets Dutch/jongensprostitutie-bronnen.pdf]

BRONNEN JONGENSPROSTITUTIE

Dit bestand geeft een overzicht van organisaties die betrokken zijn geweest bij de ontwikkeling van de bijbehorende factsheet en van beschikbare achtergrondinformatie (bronnen).

BETROKKEN ORGANISATIES

In het maken van deze factsheet over jongensprostitutie hebben de volgende organisaties input geleverd:

- Expertisecentrum Mensenhandel en (jeugd)Prostitutie. Voor vragen en/of opmerkingen over de factsheet, kunt u emailen met de hoofdauteur: Glenn van den Akker, g.vd.akker@lumenswerkt.nl
- CoMensha, het landelijk coördinatiecentrum tegen mensenhandel
- Bureau Nationaal Rapporteur Mensenhandel en Seksueel Geweld tegen Kinderen (BNRM)
- MOVISIE
- GGD GHOR
- Veilig Thuis

BRONNEN

De volgende documenten en informatiebronnen geven meer informatie over de signalen van jongensprostitutie, risicofactoren, en dingen om op te letten bij het doorlopen van de 5 stappen van de meldcode huiselijk geweld en kindermishandeling:

- Signalerings-protocol mensenhandel. Veilig Thuis en Moviera. 2016. Ede. http://www.moviera.nl/wp-content/uploads/2016/07/Signaleringsprotocol-mensenhandel-augustus-2016.pdf
- Signaleringsprotocol Loverboys. Steunpunt Huiselijk Geweld. 2014. http://www.moviera.nl/wp-content/uploads/2014/02/20140224-signaleringsprotocol-loverboys-definitief.pdf
- Hoe signaleer je slachtoffers? Stappenplan voor professionals. Aanpak van loverboy/mensenhandel problematiek in de zorg voor jeugd. Nederlands Jeugdinstituut, 2015. https://www.jeugdzorgnederland.nl/contents/documents/2016-actieplan-azough---handreiking-en-stappenplan-signalering.pdf
- Minderjarige jongens die hun lichaam exploiteren: Jongensprostitutie. Ruilseks signaleren, bespreekbaar maken en motiveren tot stoppen. MOVISIE, 2013. https://www.movisie.nl/publicaties/jongensprostitutie-minderjarige-jongens-die-hun-lichaam-exploiteren
- Slachtoffers loverboys: signalering. NJi. https://www.nji.nl/nl/Kennis/Dossier/Slachtoffers-loverboys/Aanpak/Signalering
- Slachtoffers loverboys: Risicoprofiel slachtoffers. NJi. https://www.nji.nl/nl/Kennis/Dossier/Slachtoffers-loverboys/Achtergrond/Risicoprofiel-slachtoffers
- Seksuele uitbuiting van jongens in Nederland. Paul van Gelder et al, SHOP Den Haag, Amsterdam, 2017. https://www.rijksoverheid.nl/documenten/rapporten/2017/05/08/tk-bijlage-1-eindrapport-seksuele-uitbuiting-van-jongens-in-nederland
- Mensenhandel: vijfde rapportage van de Nationaal Rapporteur. Bureau Nationaal Rapporteur Mensenhandel, Den Haag. https://www.nationaalrapporteur.nl/binaries/rapportage-5-(ned)-2006_tcm23-34835.pdf
- Anika Boersma et al. Signalenkaart mannelijke slachtoffers in de seksuele uitbuiting.
- Repetur, L.; Veenstra, J. Vrijbuiters uitgebuit. SWP, 2010. https://www.movisie.nl/publicaties/vrijbuiters-uitgebuit
- Wetsartikel 237F, beschrijving mensenhandel.
- https://www.mensenhandelweb.nl/system/files/documents/10%20feb%202014/Artikel%20273f.pdf
- Signalenlijst jeugdprostitutie
- http://expertisecentrum-mensenhandel-jeugdprostitutie.nl/wp-content/uploads/2016/07/Signalenlijst-Mensenhandel-en-jeugdprostitutie.pdf
- Went, D. van en Castelijns, H. Jongensprostitutie in Beeld, Projectrapportage 2015-2017. (2018) Expertisecentrum Mensenhandel en (jeugd)Prostitutie.
- Movisie: Factsheet Jongensprostitutie, 2009.
- https://www.movisie.nl/sites/movisie.nl/files/publication-attachment/Factsheet%20jongensprostitutie%20%5BMOV-239227-0.4%5D.pdf
- http://www.bonger.nl/PDF/Samenvattingen%20bonger/samenvattingVERBORGEN%20WERELDEN.pdf
- https://www.nationaalrapporteur.nl/binaries/Slachtoffermonitor%20mensenhandel%202012-2016_Nationaal%20Rapporteur%20(i)_tcm23-285357.pdf
- https://www.movisie.nl/publicaties/jongensprostitutie-minderjarige-jongens-die-hun-lichaam-exploiteren
- Vrijbuiters uitgebuit: Minderjarige jongens in de prostitutie. SWP, 2010.
