# Supplementary material for: Lesser-known types of violence: Helping nurses and midwives to signal and act
Source: Int J Nurs Stud Adv. 2022 Sep 17;4:100098. doi: 10.1016/j.ijnsa.2022.100098 (PMC11080451; doi:10.1016/j.ijnsa.2022.100098)
Supplement: Supplementary file 1 [file mmc1.zip › Factsheets Dutch/kindermishandeling.pdf]

KINDERMISHANDELING

GEBRUIK BIJ ELKE VORM VAN HUISELIJK GEWELD EN KINDER-MISHANDELING DE MELDCODE! ▶

WAT IS KINDERMISHANDELING?

De Jeugdwet definieert kindermishandeling als: 'Elke vorm van voor een minderjarige bedreigende of gewelddadige interactie van fysieke, psychische of seksuele aard, die de ouders of andere personen ten opzichte van wie de minderjarige in een relatie van afhankelijkheid of van onvrijheid staat, actief of passief opdringen, waardoor ernstige schade wordt berokkend of dreigt te worden berokkend aan de minderjarige in de vorm van fysiek of psychisch letsel.'

VORMEN

Kindermishandeling kent verschillende vormen, zoals lichamelijke mishandeling of verwaarlozing, psychische mishandeling of verwaarlozing, seksueel misbruik (ook online) en het getuige zijn van (partner)geweld. Specifieke vormen van lichamelijke mishandeling zijn schudden van een baby met hersenletsel als gevolg, pediatric condition falsification en meisjesbesnijdenis.

VEILIGHEID EN RISICO'S INSCHATTEN

Veel kennis over risico- en beschermende factoren is samengevat in diverse Richtlijnen kindermishandeling.

Enkele aandachtspunten:

- Risicofactoren vergroten de kans op kindermishandeling.
- Hoe meer risicofactoren, hoe groter de kans op het ontstaan of herhaling van kindermishandeling.
- De belangrijkste risicofactoren hebben te maken met kenmerken van de ouders en de leefomstandigheden van het gezin.
- Partnergeweld is een belangrijke risicofactor.

Verschillende instrumenten kunnen helpen om een risicotaxatie te maken. In de praktijk blijken er soms misverstanden te zijn over risicotaxatie. Lees hier meer over in de publicatie 'Veiligheid en risico's inschatten: wat helpt'.

Verschil tussen acute en structurele onveiligheid

- Bij **acute onveiligheid** kan het o.a. gaan om direct fysiek gevaar of afwezigheid van basale verzorging bij zorgafhankelijke kinderen
- Bij **structurele onveiligheid** gaat het om gezinnen of huishoudens waar sprake is van zich herhalende of voortdurende onveilige gebeurtenissen en situaties. Deze structurele onveiligheid wordt (soms bij gebrek aan 'kindsignalen') door veel beroepskrachten niet gesignaleerd.

SIGNALEN: HOE ZIE IK DAT EEN KIND SLACHTOFFER KAN ZIJN?

Kinderen laten soms geen signalen zien en lijken goed te functioneren, ondanks veel problemen bij de ouders. Toch kan er sprake zijn van onveiligheid in de opvoedingssituatie. Er bestaan veel overzichten van signalen van kindermishandeling. Vrijwel alle signalen van kindermishandeling zijn aspecifiek: niet één kenmerk in uiterlijk, gedrag of ontwikkeling is specifiek voor kindermishandeling en alle kenmerken kunnen ook een signaal zijn voor andere problemen. Belangrijker dan kennis over signalen is daarom misschien wel een 'gevoeligheid' voor signalen en het wegen ervan.

Professionals die met volwassenen werken dienen de kindcheck uit te voeren. Dit maakt deel uit van de meldcode en is dus niet vrijblijvend. Professionals zijn er verantwoordelijk voor om de meldcode te volgen bij signalen en feiten die een vermoeden van kindermishandeling onderbouwen.

FEITEN EN CIJFERS

- Volgens de Nationale Prevalentiestudie Mishandeling (NPM) zijn in **2010 in Nederland** naar schatting bijna **119.000 kinderen en jongeren van 0 tot 18 jaar** blootgesteld aan een vorm van kindermishandeling. Dit is ruim **3 procent** van alle kinderen.
- De meest voorkomende vormen van kindermishandeling zijn emotionele en fysieke verwaarlozing, met respectievelijk 36 en 24 procent van de gevallen.
- Meer cijfers uit de Nationale Prevalentiestudie vindt u hier.
- In het **Scholierenonderzoek Kindermishandeling 2016** onder leerlingen in het voortgezet onderwijs (klas 1-4) zegt bijna **25 procent van de leerlingen** ooit in het leven **slachtoffer geweest** te zijn van kindermishandeling. Dit komt overeen met gegevens uit onderzoek naar adverse childhood experiences (ACE's) onder leerlingen uit groep 7 en 8 van het primair onderwijs.

ADVIES / MELDEN

Voor advies, melden en/of doorverwijzing naar opvang en/of andere hulp, bel: Veilig Thuis **0800 20 00**
Bij acuut gevaar bel **112**

ENGELSE VERTALING

Zie hier.

Deze factsheet is ontwikkeld door het Nederlands Jeugdinstituut (NJi) in samenwerking met ketenpartners en deskundigen.

november 2018

KINDERMISHANDELING

AANDACHTSPUNTEN

Bij elke vorm van huiselijk geweld en kindermishandeling dien je als professional de meldcode te gebruiken. Algemene meldcode richtlijnen (zoals de 5 stappen) staan niet op deze factsheet beschreven – bezoek daarvoor de link.

Wijziging meldcode per 1 januari 2019: Het bij Veilig Thuis melden van vermoedens van kindermishandeling is met ingang van 1 januari 2019 noodzakelijk in alle gevallen waarbij sprake is van acute en/of structurele onveiligheid. Lees meer over werken met de gewijzigde meldcode.

MEER WETEN?

- Definitie en vormen kindermishandeling (Dossier kindermishandeling, NJi)
- Lichamelijke mishandeling (Dossier kindermishandeling, NJi)
- Richtlijnen kindermishandeling (Dossier kindermishandeling, NJi)
- Signaleren van kindermishandeling (Dossier kindermishandeling, NJi)
- Signalenkaart Huiselijk geweld en kindermishandeling
- Veiligheid en risico's inschatten: wat helpt? De zin en onzin van risicotaxatie-instrumenten
- Leren van calamiteiten 2 - Veiligheid van kinderen in kwetsbare gezinnen (Inspectie Justitie en Veiligheid)
- De kindcheck (Augeo)
- Risicofactoren, beschermende factoren en signalen – Aanbevelingen (Richtlijn: kindermishandeling, NCJ 2016)
- Het afwegingskader in de Meldcode huiselijk geweld en kindermishandeling (Rijksoverheid, 2017)
- Veilig Thuis (Rijksoverheid)

Deze factsheet is ontwikkeld door het Nederlands Jeugdinstituut (NJi) in samenwerking met ketenpartners en deskundigen.

november 2018
