# Supplementary material for: Lesser-known types of violence: Helping nurses and midwives to signal and act
Source: Int J Nurs Stud Adv. 2022 Sep 17;4:100098. doi: 10.1016/j.ijnsa.2022.100098 (PMC11080451; doi:10.1016/j.ijnsa.2022.100098)
Supplement: Supplementary file 1 [file mmc1.zip › Factsheets Dutch/kindermishandeling-obv-oudersignalen.pdf]

DE KINDCHECK

SIGNALEREN VAN KINDERMISHANDELING OP BASIS VAN OUDERSIGNALEN

GEBRUIK BIJ ELKE VORM VAN HUISELIJK GEWELD EN KINDER-MISHANDELING DE MELDCODE! ▶

WAT IS DE KINDCHECK

De Kindcheck is onderdeel van de Wet meldcode huiselijk geweld en kindermishandeling. Doel van de Kindcheck is om meer kinderen in beeld te brengen die ernstig risico lopen mishandeld of verwaarloosd te worden.

De Kindcheck is speciaal bedoeld voor mensen die werken met volwassen cliënten/patiënten, zoals (huis)artsen, verpleegkundigen, maatschappelijk werkers, psychiaters en psychologen. De Kindcheck houdt in dat je in je contacten met volwassen cliënten/patiënten nagaat of er kinderen in het gezin zijn en inschat of zij veilig zijn.

SIGNALEN: OP WELKE OUDERSIGNALEN LET JE?

Je doet de Kindcheck bijvoorbeeld bij volwassen cliënten/patiënten:

- met ernstige psychische problemen
- na een suïcidepoging
- met een drugs- en/of alcoholverslaving
- die slachtoffer of pleger zijn van huiselijk geweld
- die zeer agressief zijn en/of vuurwapengevaarlijk
- die erg verwaarloosd zijn en/of geen vaste woon of verblijfplaats hebben
- met een verstandelijke beperking

Ook wanneer een volwassene wordt vastgehouden door politie/justitie is het van belang om de Kindcheck te doen.

AANDACHTSPUNTEN BIJ HET DOORLOPEN VAN DE 5 STAPPEN IN DE MELDCODE

Bij elke vorm van huiselijk geweld en kindermishandeling dien je als professional de meldcode te gebruiken. Algemene meldcode richtlijnen (zoals de 5 stappen) staan niet op deze factsheet beschreven – bezoek daarvoor de link. Wél staan hier aandachtspunten specifiek voor deze vorm van geweld.

Je doet de Kindcheck in stap 1 van de meldcode bij het in kaart brengen *van signalen*.

Heb je twijfels over de veiligheid van de kinderen, bijvoorbeeld omdat de patiënt een beperkt sociaal netwerk heeft of er geen andere ouder is? Dan doorloop je de stappen van de meldcode. Je legt eerst vast door welke signalen bij de ouder je twijfelt over de veiligheid van de kinderen.

Na het uitvoeren van een positieve Kindcheck (d.w.z. zorgen blijven bestaan na stap 4), ATLIJD melden bij Veilig Thuis. Daarna kan in overleg met Veilig Thuis besproken worden of dan wel welke mogelijkheden de melder heeft om veiligheid te organiseren.

Belangrijk: Om te waarborgen dat de Kindcheck altijd wordt uitgevoerd bij zorgen over volwassen cliënten/patiënten, is het van belang om standaard Kindcheck vragen op te nemen in het cliënten/patiënten dossier. Zo wordt het niet vergeten en kunnen collega's eerder ondernomen stappen terugvinden.

FEITEN EN CIJFERS

Onderzoek naar de Kindcheck laat zien dat er in 91% van de gevallen een vorm van kindermishandeling speelt. Driekwart van deze kinderen waren nog niet bekend bij Veilig Thuis (Diderich et al., 2013). Door het signaleren op **oudersignalen** i.p.v. alleen **kindsignalen** kan er eerder hulp worden ingezet in een gezin. Sinds 2013 is de Kindcheck verplicht onderdeel van de meldcode. Kinderen van ouders met een psychische stoornis (KOPP) en/of een verslaving (KVO) lopen een verhoogd risico op verwaarlozing doordat ouders als gevolg van persoonlijke problematiek onvoldoende in staat zijn om de zorg te bieden voor hun kind die nodig is (basiszorg, emotionele en affectieve ondersteuning).

MEER INFORMATIE

Zie de Bronnen en:

- Augeo. De Kindcheck.
- Augeo magazine. De Kindcheck voor medici: signalen van ouders

ADVIES/MELDEN

Neem contact op met de aandachtsfunctionaris van de instelling en/of bel voor advies, melden en/of doorverwijzing naar opvang en/of andere hulp:

- Veilig Thuis **0800 20 00**

Bij acuut gevaar bel **112**.

Engelse vertaling: Zie hier.

Deze factsheet is ontwikkeld door De Kindcheck i.s.m. diverse ketenpartners.

november 2018
