# Supplementary material for: Lesser-known types of violence: Helping nurses and midwives to signal and act
Source: Int J Nurs Stud Adv. 2022 Sep 17;4:100098. doi: 10.1016/j.ijnsa.2022.100098 (PMC11080451; doi:10.1016/j.ijnsa.2022.100098)
Supplement: Supplementary file 1 [file mmc1.zip › Factsheets Dutch/kindermishandeling-obv-oudersignalen-bronnen.pdf]

BRONNEN DE KINDCHECK

Dit bestand geeft een overzicht van organisaties die betrokken zijn geweest bij de ontwikkeling van de bijbehorende factsheet en van beschikbare achtergrondinformatie (bronnen).

BETROKKEN ORGANISATIES

In het maken van deze factsheet over de Kindcheck voor professionals in alle beroepen die een meldcode huiselijk geweld en kindermishandeling hanteren, hebben de volgende organisaties input geleverd:

- De Kindcheck. Voor vragen en/of opmerkingen over de factsheet, kunt u emailen met de hoofdauteur: Hester Diderich, h.diderich@kindcheck-ggz.nl
- Veilig Thuis, Wanda Lansbergen
- Radboud umc, Karin van Rosmalen – Nooijens
- Jolanda den Hartog, SIEN, voor mensen met een verstandelijke beperking
- Augeo

BRONNEN

De volgende documenten en informatiebronnen geven meer informatie over de Kindcheck:

- Diderich et al., 2013
- Augeo. De Kindcheck.
- Augeo magazine. De Kindcheck voor medici: signalen van ouders
