# Supplementary material for: Lesser-known types of violence: Helping nurses and midwives to signal and act
Source: Int J Nurs Stud Adv. 2022 Sep 17;4:100098. doi: 10.1016/j.ijnsa.2022.100098 (PMC11080451; doi:10.1016/j.ijnsa.2022.100098)
Supplement: Supplementary file 1 [file mmc1.zip › Factsheets Dutch/kind-oudergeweld.pdf]

OUDER-MISHANDELING

GEBRUIK BIJ ELKE VORM VAN HUISELIJK GEWELD EN KINDER-MISHANDELING DE MELDCODE! ▶

WAT IS OUDER-MISHANDELING?

Ouder-mishandeling (ook wel: 'kind-ouder geweld' of 'huiselijk geweld van jongeren tegen hun ouders'; niet te verwarren met ouderENmishandeling) is: niet-incidenteel geweld in het gezin, gepleegd door een (doorgaans thuiswonende afhankelijke) jeugdige of jongvolwassene, dat gericht is op (een van) de ouders. Het gaat om herhaaldelijk en ernstig geweld (geen 'normale' puberteitsconflicten). Het geweld kan psychisch, fysiek en seksueel zijn, met financiële afpersing en materiele schade.

SIGNALEN: HOE HERKEN IK OUDER-MISHANDELING?

Er is veel schaamte bij ouders (gevoel van falen). Ouders vragen doorgaans pas hulp in vergevorderd stadium. Mogelijke signalen:

- Ouder(s) is (zijn) bang voor eigen kind.
- Toename verbaal geweld (vaak rond 12-14 jaar) en escalatie naar ernstige dreiging en fysiek geweld.
- Brandstichting, (sporen van) materiele schade.
- Heftige woede-uitbarstingen bij jongere n.a.v. triggers (bv. alcohol, drugs, internet- en gameverslaving).
- Geringe dagbesteding van de jongere, omkering dag-nachtritme.
- (Ernstige) gedragsproblemen, agressie en woede i.v.m. psychopathologie bij de jongere.
- Voor ouder(s) onverwachte gedragsverandering bij de jongere ("was vroeger helemaal niet zo").
- Meerdere vormen van huiselijk geweld in het gezin, o.a. naar siblings.
- Geweld door jongere buiten gezin (bv. mishandeling (ex-)partner).
- Negatieve communicatie- en interactiepatronen in het gezin.
- Mannelijke dominantie als sociale norm binnen het gezin.
- Probleemgedrag bij jongere op school, (langdurig) verzuim, schorsingen, pesten en gepest worden.
- Schulden bij jongere en financiële uitbuiting en afpersing van de ouder(s).
- Huisverbod, melding(en) of aangifte bij politie door ouder(s) of familie.
- Ouder isoleert zich en raakt uitgeput.

RISICOFACTOREN: WIE IS EXTRA KWETSBAAR VOOR DIT GEWELD?

Er is weinig bekend over risicofactoren voor ouder-mishandeling. Het algemene beeld uit zowel praktijk als de wetenschappelijke literatuur is dat ouder-mishandeling wordt gepleegd door thuiswonende jongens vanaf 14/15 jaar, dat het veelal gericht is op alleenstaande (gescheiden) moeders, en dat dit gezinnen in alle lagen van de bevolking kan treffen.

Jongeren met psychiatrische problematiek, met name wanneer dit zich pas in de puberteit openbaart en dit gepaard gaat met gedragsproblemen, lage frustratietolerantie en gering inlevings- en aanpassingsvermogen lijken een specifieke groep plegers te vormen.

Verstoorde ouder-kind interactie: een zeer permissieve opvoeding (geen grenzen); ontbreken van gezinscohesie of extreme bescherming; verschuiving van gezagsverhoudingen binnen het gezin (bv. door scheiding); geweld in het gezin; onvoorspelbaarheid.

FEITEN EN CIJFERS

- Bij naar schatting 10% van alle incidenten van huiselijk geweld die jaarlijks ter kennis komen van de politie, zijn de eigen ouders slachtoffers.
- Meer dan 80% van de plegers zijn jongens.
- In twee derde van de gevallen is de biologische moeder het slachtoffer.

ADVIES / MELDEN

Voor advies, melden en/of doorverwijzing naar opvang en/of andere hulp, bel:

- Veilig Thuis **0800 20 00**

Bij acuut gevaar bel **112**

ENGELSE VERTALING

Zie hier.

Deze factsheet is ontwikkeld door TNO i.s.m. diverse ketenpartners.

november 2018

OUDER-MISHANDELING

AANDACHTSPUNTEN

Bij elke vorm van huiselijk geweld en kindermishandeling dien je als professional de meldcode te gebruiken. Algemene meldcode richtlijnen (zoals de 5 stappen) staan niet op deze factsheet beschreven – bezoek daarvoor de link. Wél staan hier aandachtspunten specifiek voor deze vorm van geweld:

- Vraag aan ouder(s) hoe het met ze gaat; neem hen serieus. Vraag door.
- Ga na bij GGZ-behandeling van een jongere of er sprake is van agressie. Vraag door.
- Let ook op de andere kinderen in het gezin (als slachtoffers of plegers).
- Om ernstig geweld te doen stoppen kan het nodig zijn de politie in te schakelen (aangifte of huisverbod).
- Overleg met Veilig Thuis over mogelijkheid van uithuisplaatsing.
- Overweeg opvoedings-ondersteuning, MST, Relationele Gezinstherapie, MDFT, Geweldloos Verzet of Agressieregulatie programma.

MEER INFORMATIE

Zie de bronnen en:

- De website Holes in the wall
- De databank van Effectieve interventies huiselijk geweld en seksueel geweld
- Dit rapport van TNO en Movisie over ouder-mishandeling

Deze factsheet is ontwikkeld door TNO i.s.m. diverse ketenpartners.

november 2018
