# Supplementary material for: Lesser-known types of violence: Helping nurses and midwives to signal and act
Source: Int J Nurs Stud Adv. 2022 Sep 17;4:100098. doi: 10.1016/j.ijnsa.2022.100098 (PMC11080451; doi:10.1016/j.ijnsa.2022.100098)
Supplement: Supplementary file 1 [file mmc1.zip › Factsheets Dutch/kind-oudergeweld-bronnen.pdf]

BRONNEN OUDER-MISHANDELING

Dit bestand geeft een overzicht van organisaties die betrokken zijn geweest bij de ontwikkeling van de bijbehorende factsheet en van beschikbare achtergrondinformatie (bronnen).

BETROKKEN ORGANISATIES

In het maken van deze factsheet over ouder-mishandeling voor professionals in alle beroepen die een meldcode huiselijk geweld en kindermishandeling hanteren, hebben de volgende organisaties input geleverd:

- TNO (hoofdauteur Remy Vink, voor vragen of opmerkingen: remy.vink@tno.nl)
- MOVISIE
- GGD GHOR Nederland
- Augeo Foundation
- Veilig Thuis
- VVAK
- Het Lorentzhuis

BRONNEN

De volgende documenten en informatiebronnen geven meer informatie over de signalen van ouder-mishandeling, risicofactoren, en dingen om op te letten bij dit type geweld bij het doorlopen van de 5 stappen van de meldcode huiselijk geweld en kindermishandeling:

- Beckmann, L., Bergmann, M. C., Fischer, F. and Mosel, T. (2017) Risk and Protective Factors of Child-to-Parent Violence: A Comparison Between Physical and Verbal Aggression, Journal of Interpersonal Violence, first published online 12th December 2017
- Biehal, N. (2012). Parent abuse by young people on the edge of care: a child welfare perspective. Social policy & society, 11(2),251-263.
- Calvete E, Orue I, Gamez-Guadix M, Bushman BJ. (2015) Predictors of child-to-parent aggression: A 3-year longitudinal study. Dev Psychol. 2015 May;51(5):663-76. doi: 10.1037/a0039092. Epub 2015 Mar 30.
- Cottrell, B. & Monk, P. (2004). Adolescent-to-parent abuse: A qualitative overview of common themes. J.Fam. Issues, 25(8), 1072-1095.
- Eckstein, N.J. (2004). Emergent issues in families experiencing adolescent-to-parent abuse. Western Journal of Communication, 68(4), 365-388.
- Holt, A. (2013). Adolescent-to-Parent Abuse: Current Understandings in Research, Policy and Practice. Policy Press, Bristol University.
- Holt, A. (2015) Working with Adolescent Violence and Abuse Towards Parents: Approaches and Contexts for Intervention. London. Routledge.
- Ibabe, I. & Jaureguizar, J. (2010). Child-to-parent violence: Profile of abusive adolescents and their families. Journal of Criminal Justice, 38(4), 616-624.
- Kennair, N. & Mellor, D. (2007). Parent abuse: a review. Child Psychiatry Hum. Dev., 38(3), 203-219.
- Weinblatt, U. & Omer, H. (2008). Geweldloos verzet: een behandeling voor ouders van kinderen met ernstige gedragsproblemen. Gezinstherapie Wereldwijd, 19(4): 389-418.
- Pagani, L., Larocque, D., Vitaro, F., & Tremblay, R.E. (2003). Verbal and Physical Abuse Toward Mothers: The Role of Family Configuration, Environment, and Coping Strategies. Journal of Youth and Adolescence,32(3), 215-222.
- Routt, G., & Anderson L. (2011). Adolescent violence towards parents. Journal of Aggression, Maltreatment and Trauma, 20(1), 1-18.
- Ulman, A., & Straus, M.A. (2003). Violence by children against mothers in relation to violence between parents and corporal punishment by parents. J of Comparative Family Studies 34(1), 41-60.
- Vink, R.M., Goes, A., Doornink, N., Broerse, A., Pannebakker, F., Zwan, van der R., Schakenraad, W. (2014) Huiselijk geweld door kinderen en jongeren tegen hun ouders. Utrecht/Leiden. Movisie/TNO
