# Supplementary material for: Lesser-known types of violence: Helping nurses and midwives to signal and act
Source: Int J Nurs Stud Adv. 2022 Sep 17;4:100098. doi: 10.1016/j.ijnsa.2022.100098 (PMC11080451; doi:10.1016/j.ijnsa.2022.100098)
Supplement: Supplementary file 1 [file mmc1.zip › Factsheets Dutch/kwetsbare-zwangeren.pdf]

KWETSBARE ZWANGEREN EN BESCHERMING VAN HET ONGEBOREN KIND

GEBRUIK BIJ ELKE VORM VAN HUISELIJK GEWELD EN KINDER-MISHANDELING DE MELDCODE! ▶

WIE ZIJN KWETSBARE ZWANGEREN?

Kwetsbare zwangeren hebben verhoogd risico op gezondheidsschade voor zichzelf en ongeboren kind, op een slechte zwangerschapsuitkomst en op mishandeling en verwaarlozing van het kind. Een slechte start van een baby werkt levenslang door. Ongunstige opgroei-omstandigheden voor kinderen kunnen al in de zwangerschapsperiode worden gesignaleerd en betreffen zowel gedrag en leefstijl van de zwangere als die van de (ex)partner/mede-opvoeder.
Situaties waarin kwetsbaarheid bestaat van moeder en (ongeboren) kind zijn bijvoorbeeld situaties waarin aanstaande ouders: slachtoffer/pleger zijn van huiselijk geweld, psychiatrisch patiënt zijn of waarbij bepaalde psychiatrische problemen spelen, een Licht Verstandelijke Beperking (LVB) hebben, een verslaving hebben, erg jong zijn (tienerzwangerschappen), of problemen ervaren op meerdere levensgebieden ('multiproblematiek'; zie *Signalen en risicofactoren* voor een uitgebreider overzicht).

SIGNALEN EN RISICOFACTOREN

- Partnergeweld voorafgaand aan en gedurende de zwangerschap.
- Ervaringen van aanstaande ouder(s) met kindermishandeling (waaronder seksueel misbruik) in de eigen jeugd.
- Ondertoezichtstelling (OTS) eerder kind.
- Psychiatrische problemen bij aanstaande ouders en/of Licht Verstandelijke Beperking (LVB).
- (Postnatale) depressie.
- Roken, alcohol- en drugsgebruik tijdens de zwangerschap.
- Alcohol- en drugsmisbruik / verslaving bij de partner/mede-opvoeder.
- Tienerzwangerschappen, onbedoeld zwanger, laag opgeleid en alleenstaand (vooral in combinatie: een groep die veel in Voorzorg komt).
- Vroeggeboorte en laag geboortegewicht.
- Negatieve opvattingen over ongeboren kind en/of eerdere kinderen.
- Niet of laat in zorg komen, therapie-ontrouw, vaak afspraak afzeggen of niet verschijnen, onvoldoende zelfzorg.
- Problemen en stress op meerdere levensgebieden: huisvesting, financiële problemen en schulden, werkloosheid, onverzekerd zijn, criminaliteit, geweld in het gezin, geen of niet-helpend sociaal netwerk.

HULP BIJ SIGNALEREN VOOR DIVERSE GROEPEN PROFESSIONALS

- *Verloskundige zorgverleners*: screen op basis van de anamnese en ALPHA-NL, R4U of Mind2Care .
- *Kraamverzorgenden*: gebruik de TNO Checklist Vroegsignalering in de kraamtijd.
- *Jeugdgezondheidszorg*: screen gedurende reguliere contactmomenten en/of op basis van (Pre)SPARK, GIZ, SamenStarten.
- *Iedereen*: doe de Kindcheck, bespreek je zorgen met de aanstaande ouders, bespreek je zorgen in het MDO (multidisciplinair overleg), overleg met Veilig Thuis.

FEITEN EN CIJFERS

- Van alle zwangeren in Nederland drinkt 8,9% wel eens alcohol en 0,8% wel eens meer dan vier glazen op één dag.
- Van alle laagopgeleide vrouwen rookt 22,1% dagelijks gedurende de hele zwangerschap, en respectievelijk 5,5% en 0,9% van de midden- en hoogopgeleide vrouwen.
- 12% van de Nederlandse zwangeren ervaart tijdens of na de zwangerschap een depressieve stoornis
- Per jaar bevallen rond de 1500 tieners (< 20 jaar) (0.9 % van het totaal).

ADVIES / MELDEN

Voor advies, melden en/of doorverwijzing naar opvang en/of andere hulp, bel:

- Veilig Thuis: 0800 20 00

Bij acuut gevaar: 112

ENGELSE VERTALING

Zie hier.

Deze factsheet is ontwikkeld door de Nederlandse Vereniging voor Obstetrie en Gynaecologie (NVOG), het Erasmus MC , TNO en Veilig Thuis in samenwerking met diverse ketenpartners.

december 2018

KWETSBARE ZWANGEREN EN BESCHERMING VAN HET ONGEBOREN KIND

BESCHIKBARE HULP TIJDENS DE ZWANGERSCHAP

Laagdrempelig:

- Prenatale Huisbezoeken Jeugdverpleegkundige (JGZ) of (Prenataal) Stevig Ouderschap
- Sociaal wijkteam (praktische hulp op alle leefdomeinen, lichte opvoedingsondersteuning, toegang specialistische hulp)
- Relatietherapie (via huisarts of sociaal wijkteam)
- Andere afspraken die gemaakt zijn in het Verloskundig SamenwerkingsVerband

Specifiek:

- www.voorzorg.info
- (Jeugd-)GGZ, Verslavingszorg, plegerbehandeling
- POP-poli (Psychiatrie, Obstetrie en Pediatrie (kindergeneeskunde)): begeleiding zwangerschap in relatie tot psychiatrie
- Vormen van thuisbegeleiding, diverse programma's voor tienermoeders, LVB-ouders, ouders met psychiatrische problematiek.

AANDACHTSPUNTEN

- Prenatale ondertoezichtstelling (OTS) kan gedurende de hele zwangerschap worden uitgesproken. Een gezinsvoogd kan dan aanwijzingen geven zoals het meewerken aan verloskundige controle en andere vormen van zorg.
- Bij suïcidaliteit en verslaving aan harddrugs is opname op grond van de Wet BOPZ mogelijk. In geval van wilsonbekwaamheid kan bij de rechter mentorschap of curatele worden aangevraagd.
- (Anoniem) advies vragen bij Veilig Thuis kan gedurende de gehele zwangerschap, vraag desgewenst naar de vertrouwensarts. Acute en structurele onveiligheid en niet meewerken aan hulp zijn redenen om te melden.

MEER INFORMATIE

Zie de bronnen en:

- www.fiom.nl
- www.tienermoeders.nl

Deze factsheet is ontwikkeld door de Nederlandse Vereniging voor Obstetrie en Gynaecologie (NVOG), het Erasmus MC , TNO en Veilig Thuis in samenwerking met diverse ketenpartners.

december 2018
