# Supplementary material for: Lesser-known types of violence: Helping nurses and midwives to signal and act
Source: Int J Nurs Stud Adv. 2022 Sep 17;4:100098. doi: 10.1016/j.ijnsa.2022.100098 (PMC11080451; doi:10.1016/j.ijnsa.2022.100098)
Supplement: Supplementary file 1 [file mmc1.zip › Factsheets Dutch/kwetsbare-zwangeren-bronnen.pdf]

BRONNEN KWETSBARE ZWANGEREN EN BESCHERMING VAN HET ONGEBOREN KIND

Dit bestand geeft een overzicht van organisaties die betrokken zijn geweest bij de ontwikkeling van de bijbehorende factsheet en van beschikbare achtergrondinformatie (bronnen).

BETROKKEN ORGANISATIES

Bij het maken van deze factsheet hebben de volgende organisaties input geleverd:

- Veilig Thuis, Anne-Marie Raat, vertrouwensarts
- TNO, Remy Vink, remy.vink@tno.nl
- Erasmus MC / Nederlandse Vereniging voor Obstetrie en Gynaecologie (NVOG), Hans J. Duvekot

BRONNEN

websites

- Veilig Thuis
- www.voorzorg.info
- www.tienermoeders.nl
- www.nunietzwanger.nl
- www.RSJ.nl (advies prenatale kinderbescherming en de rol van de overheid, juni 2015)
- www.kinderbescherming.nl (Informatieblad Bescherming nog niet geboren kinderen en voorkomen kwetsbare zwangerschappen. Raad voor de Kinderbescherming, januari 2017)
- www.TNO.nl (De eerste duizend dagen)
- www.NVAVG.nl (Handreiking kinderwens en anticonceptie bij mensen met een verstandelijke beperking. Oktober 2016)
- www.fiom.nl en www.siriz.nl (hulp bij onbedoelde zwangerschap)
- www.lkpz.nl (Kenniscentrum Psychiatrie en zwangerschap)
- www.trimbos.nl
- ALPHA-NL
- R4U
- Mind2Care
- TNO Checklist Vroegsignalering
- Verloskundig SamenwerkingsVerband
- (Pre)SPARK
- GIZ
- SamenStarten
- Kindcheck
- Nji: cijfers over tienermoeders
- TNO over roken
- TNO over alcoholgebruik

publicaties

- Ahmadabadi, Z. e.a. (2018) Maternal intimate partner violence victimization and child maltreatment. Child Abuse & Neglect, 23-33
- Austin, A. e.a. (2018) Using time-to-event analysis to identify preconception and prenatal predictors of child protective services contact. Child Abuse & Neglect, 83-91
- Chen, H. e.a. (2018) Prenatal smoking and post partum depression: a meta- analysis. Journal of Psychosomatic Obstetrics & Gynecology, , 1-9
- Guterman, K., (2015) Unintended pregnancy as a predictor of child maltreatment. Child Abuse & Neglect, 160-169
- Hafekost, K. e.a. (2017) Maternal alcohol use disorder and subsequent child protection contact: A record-linkage population cohort study. Child Abuse & Neglect, 206-214
- Huizink, A.C. (2013) Prenatal cannabis exposure and infant outcomes: Overview of studies. Prog Neuro-Psychopharmacol Biol Psychiatry 2013
- Lambregtse-Van den Berg, M., Kamp, I. van, Wennink, H. (red.) Handboek psychiatrie en zwangerschap. ISBN 9789058982698. Utrecht. Uitgeverij De Tijdstroom (2015)
- Mejdoubi, J., Heijkant, S.C.C.M., van den, Struijf, E., Leerdam, J.M., van, HiraSing, R.A., Crijnen, A.M. (2013) Risicofactoren voor kindermishandeling bij jonge hoogrisicozwangeren: design van het VoorZorg onderzoek. Tijdschrift voor Jeugdgezondheidszorg, pp 26–31
- Prindle, P. e.a Prenatal substance exposure diagnosed at birth and infant involvement with child protective services. Child Abuse & Neglect 2018, 75-83
- Steegers, E. (2017) Sociale verloskunde: gelijke kansen op een gezonde start. Ned Tijdschr Geneesk;161: 1-4
- Taylor, J. & A. Lazenbatt (2014) Child Maltreatment and High Risk Families, Dunedin Academic Press, Edinburgh. ISBN 978-1-78046-031-4
- VWS, Actieprogramma Kansrijke Start, september 2018. Den Haag. Ministerie van Volksgezondheid, Welzijn en Sport
- Wall-Wieler, E. e.a. (2018) Predictors of having a first child taken into care at birth: a population based retrospective cohort study. Child Abuse & Neglect, 1-9
- Wewerinke, A., Honig, A., Heres, M.H.B., en Wennink, J.M.B. Psychiatrische stoornissen bij zwangeren en kraamvrouwen. Ned Tijdschr Geneeskd. 2006;150:294-8.
