# Supplementary material for: Lesser-known types of violence: Helping nurses and midwives to signal and act
Source: Int J Nurs Stud Adv. 2022 Sep 17;4:100098. doi: 10.1016/j.ijnsa.2022.100098 (PMC11080451; doi:10.1016/j.ijnsa.2022.100098)
Supplement: Supplementary file 1 [file mmc1.zip › Factsheets Dutch/meisjesprostitutie.pdf]

MEISJESPROSTITUTIE

GEBRUIK BIJ ELKE VORM VAN HUISELIJK GEWELD EN KINDER-MISHANDELING DE MELDCODE! ▶

WAT IS MEISJESPROSTITUTIE?

Meisjesprostitutie is een vorm van jeugdprostitutie. Aangezien er voor jongens (zie factsheet jongensprostitutie) en meisjes verschillende adviezen gelden is er voor gekozen jeugdprostitutie op te delen. Meisjesprostitutie is het verrichten van seksuele handelingen door meisjes onder de 18 jaar voor geld, goederen of een tegenprestatie (Movisie 2009). Wanneer meisjes betaalde seks hebben hoeft dit geen mensenhandel (loverboyproblematiek) te zijn. Seks met minderjarigen kan afhankelijk van de omstandigheden kwalificeren als een zedendelict (zie *Feiten en cijfers).*

SIGNALEN: HOE KAN IK ZIEN DAT EEN MEISJE ZICH PROSTITUEERT?

Uiterlijke en fysieke gesteldheid. Ziet er overdreven verzorgd uit, ziet er plotseling uitdagend en sexy uit en verandering in kleding en make-up.
Gedrag (algemeen). Heeft (opeens) veel geld/dure spullen, vermijdt het onderwerp prostitutie of projecteert dit op anderen en eventueel drugs en/of drankmisbruik.
Gedragssignalen op seksueel gebied. Veel kennis op het gebied van seksualiteit, plotselinge gedragsverandering, normvervaging ten opzichte van seksualiteit en het hebben van wisselende contacten.
Gedrag en houding t.o.v. anderen. Vertoont sociaal wenselijk gedrag (om geen wantrouwen te wekken), vertoont totaal ander gedrag op verschillende plekken (bv. op school of in het bijzijn van vrienden) en er zijn thuis en op school problemen.
Lichamelijke signalen/gezondheidsklachten. Pijn/bloed aan geslachtsdelen, andere vaginale en genitale klachten, seksueel overdraagbare aandoeningen, blaasontsteking en ongewenste zwangerschap.
Voor meer informatie over signalen zie deze website.

RISICOFACTOREN: WIE IS EXTRA KWETSBAAR?

Meisjes lopen extra risico wanneer ze te maken hebben met:
Faseproblematiek: conflict met hun ouders, ontworstelen aan overmatige bescherming, foute vrienden ontmoeten.
Grote beïnvloedbaarheid: als gevolg van een verstandelijke beperking, psychiatrisch beeld.
(ernstige) traumatische ervaring: als gevolg van verwaarlozing, mishandeling of (seksueel) misbruik.
Multi problem: hechtingsproblematiek, uithuisplaatsingen, schoolproblemen, een laag zelfbeeld of gebrekkig sociaal netwerk.
Eergeralateerde problematiek & alleenstaande minderjarige vluchteling (AMV): steun van ouders en familie ontbreekt. (Bron: Pharos)

AANDACHTSPUNTEN VOOR DIT TYPE GEWELD BIJ HET DOORLOPEN VAN DE 5 STAPPEN IN DE MELDCODE

Bij elke vorm van huiselijk geweld en kindermishandeling dien je als professional de meldcode te gebruiken. Algemene meldcode richtlijnen (zoals de 5 stappen) staan niet op deze factsheet beschreven – bezoek daarvoor de link. Wél staan hier aandachtspunten specifiek voor deze vorm van geweld:

- Gebruik de Ris-L. Dit helpt bij signalering en screening van evt. mensenhandel.
- Contacteer een expert op dit gebied, intern of extern (zie Advies/melden).
- Ga open het gesprek in en oordeel niet over het meisje, wees betrouwbaar.

FEITEN EN CIJFERS

De wet

In Nederland is prostitutie onder de 18 jr. verboden. Er wordt gewerkt aan een wet om deze leeftijd te verhogen naar 21 jr. Wie seks heeft met of koopt van een minderjarige, loopt het risico veroordeeld te worden. Seks met iemand onder de 16 jr. is vaak strafbaar zonder dat er sprake is van betaling. Het is de verantwoordelijkheid van de klant om te controleren of een prostituee wel 18 is. Er is in deze gevallen niet altijd sprake van mensenhandel. Daar is sprake van als een ander de jeugdprostitutie faciliteert en het verdiende geld afneemt.

Omvang

Over de omvang is nog steeds weinig bekend. Minderjarigen werken vaak in verborgen circuits. Deze jongeren zijn moeilijk bereikbaar voor o.a. hulpverleners. Jongeren leven met een "geheim".

Deze factsheet is ontwikkeld door SHOP Den Haag i.s.m. diverse ketenpartners. november 2018

MEISJESPROSTITUTIE

- Bij vermoedens van jeugdprostitutie is het van belang om open en zonder vooroordeel het gesprek in te gaan. Zorg dat de jongeren zich laten testen op oa. Blaasontsteking, soa's en weten hoe ze veilig moeten werken. Zoek contact met een expert in de regio.
- Leg stappen en/of zorgen vast in het medisch dossier.

MEER INFORMATIE

Zie dit stappenplan en de bronnen.

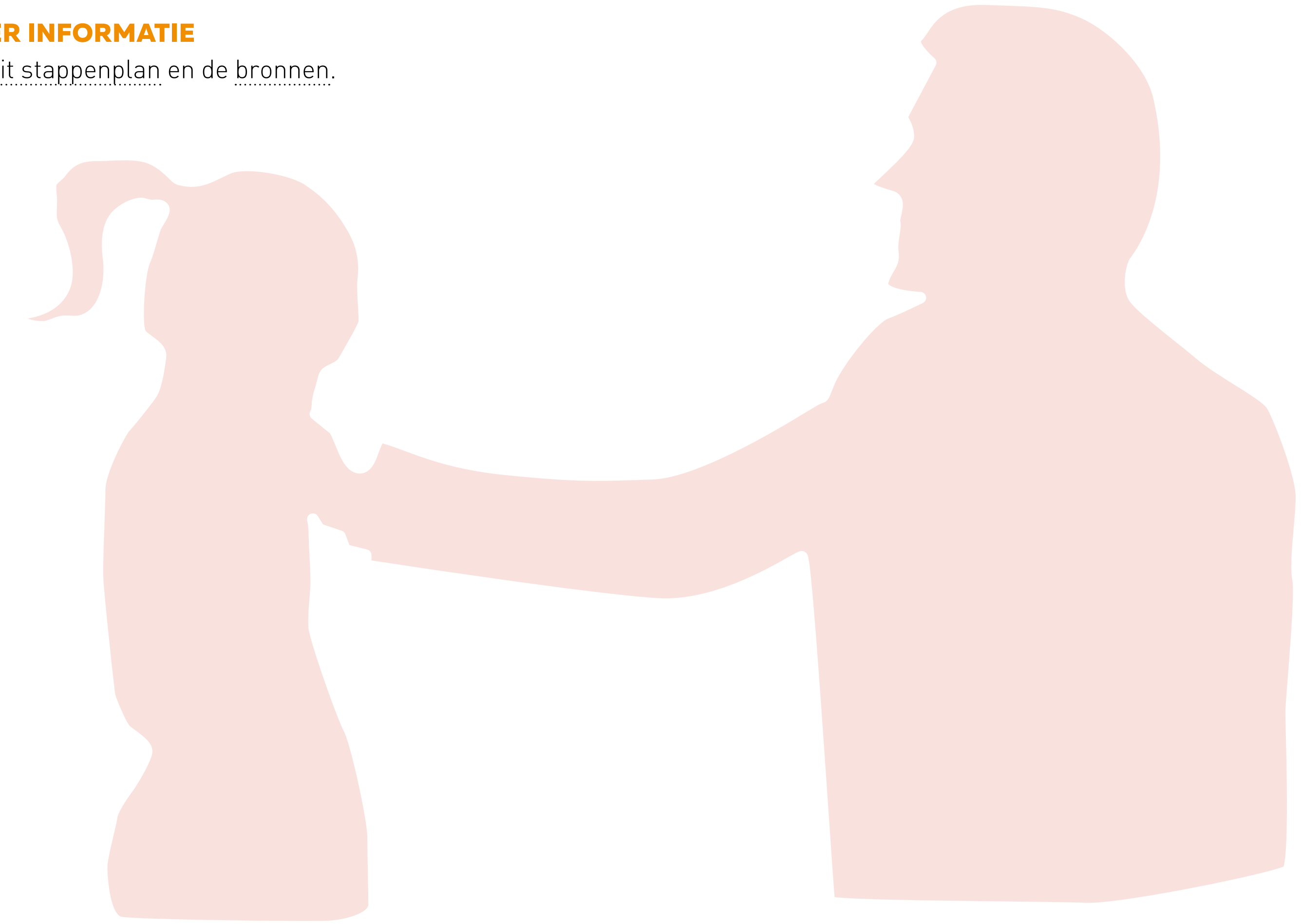

ADVIES / MELDEN

Voor advies, melden en/of doorverwijzing naar opvang en/of andere hulp, bel:

- Veilig Thuis **0800 20 00**
- CoMensha **033 44 81 186**
- een meldpunt loverboys

Bij acuut gevaar bel **112**

ENGELSE VERTALING

Zie hier.

Deze factsheet is ontwikkeld door SHOP Den Haag i.s.m. diverse ketenpartners.

november 2018
