# Supplementary material for: Lesser-known types of violence: Helping nurses and midwives to signal and act
Source: Int J Nurs Stud Adv. 2022 Sep 17;4:100098. doi: 10.1016/j.ijnsa.2022.100098 (PMC11080451; doi:10.1016/j.ijnsa.2022.100098)
Supplement: Supplementary file 1 [file mmc1.zip › Factsheets Dutch/meisjesprostitutie-bronnen.pdf]

BRONNEN MEISJESPROSTITUTIE

Dit bestand geeft een overzicht van organisaties die betrokken zijn geweest bij de ontwikkeling van de bijbehorende factsheet en van beschikbare achtergrondinformatie (bronnen).

BETROKKEN ORGANISATIES

In het maken van deze factsheet over meisjesprostitutie voor professionals in alle beroepen die een meldcode huiselijk geweld en kindermishandeling hanteren, hebben de volgende organisaties input geleverd:

- SHOP Den Haag. Voor vragen en/of opmerkingen over de factsheet, kunt u emailen met de hoofdauteur: Nicole Harms, n.harms@shop-denhaag.nl
- CoMensha, het landelijk coördinatiecentrum tegen mensenhandel
- Expertisecentrum Mensenhandel en (jeugd)Prostitutie
- Augeo
- Movisie
- Bureau Nationaal Rapporteur Mensenhandel en Seksueel Geweld tegen Kinderen
- GGD GHOR Nederland
- GGD haaglanden
- Lumens
- Veilig Thuis

BRONNEN

De volgende documenten en informatiebronnen geven meer informatie over de signalen van meisjesprostitutie, risicofactoren, en dingen om op te letten bij het doorlopen van de 5 stappen van de meldcode huiselijk geweld en kindermishandeling:

- https://www.nji.nl/nl/Download-NJi/Hoe-signaleer-je-slachtoffers-Stappenplan-voor-professionals.pdf
- https://www.nji.nl/nl/Download-NJi/Publicatie-NJi/Actieplan-Hun-verleden-is-niet-hun-toekomst.pdf
- https://www.movisie.nl/publicatie/factsheet-meisjes-prostitutie
- https://www.shop-jeugd-denhaag.nl/signalenlijst
- https://www.nji.nl/nl/Download-NJi/Hoe-signaleer-je-slachtoffers-Stappenplan-voor-professionals.pdf
- http://www.pharos.nl/nl/kenniscentrum/gezond-opgroeien/kindermishandeling/kennisdossier-kindermishandeling/exploitatie/jeugdprostitutie
