# Supplementary material for: Lesser-known types of violence: Helping nurses and midwives to signal and act
Source: Int J Nurs Stud Adv. 2022 Sep 17;4:100098. doi: 10.1016/j.ijnsa.2022.100098 (PMC11080451; doi:10.1016/j.ijnsa.2022.100098)
Supplement: Supplementary file 1 [file mmc1.zip › Factsheets Dutch/mensenhandel.pdf]

MENSENHANDEL

GEBRUIK BIJ ELKE VORM VAN HUISELIJK GEWELD EN KINDERMISHANDELING DE MELDCODE! ▶

WAT IS MENSENHANDEL?

In Nederland verstaat men onder mensenhandel:

- **wat:** het werven (voor werk), vervoeren of huisvesten van iemand,
- **hoe:** met gebruik van dwang, geweld, misleiding of misbruik makende van een kwetsbare positie (zoals bij kinderen),
- **het doel:** iemands inkomsten afnemen of niet uitbetalen *(uitbuiting)*.

Dit kan zich in de seksindustrie afspelen, maar ook in andere sectoren (zoals de (land)bouw, huishoudelijk werk of de criminaliteit (bijv. wietteelt / drugs dealen). Orgaanhandel en gedwongen draagmoederschap zijn ook vormen van mensenhandel. Mensenhandel kan over grenzen heen gebeuren of binnen Nederland ('binnenlandse mensenhandel'). Een voorbeeld van binnenlandse mensenhandelaren zijn 'loverboys'.

SIGNALEN:
HOE KAN IK ZIEN DAT IEMAND SLACHTOFFER IS?

Bij alle vormen van mensenhandel:

- er is meestal een begeleider bij de persoon
- vage uitleg voor verwondingen
- bedreiging (van het slachtoffer zelf of van familieleden)
- klachten door het werk (bijv. ongelukken bij werk in de bouw en meerdere zwangerschappen, abortussen, soa's of vaginale en genitale klachten zoals blaasontsteking bij sekswerk)
- psychosomatische klachten (psychische klachten die zich lichamelijk uiten)
- drugs-/alcoholverslaving
- tatoeages (vraag naar de betekenis en de manier waarop ze zijn geplaatst)

Mensenhandel binnen Nederland:

- wordt geïsoleerd van familie/vrienden + afhankelijkheid van ander
- plotseling veel geld / andere kleding
- gedragsveranderingen
- afpersing (bijv. met filmpjes)
- verslechterende schoolprestaties

Mensenhandel over grenzen:

- wonen op de werkplek
- niet zelf reis geregeld
- opgebouwde 'schulden'
- paspoort niet in eigen bezit
- werkadres niet bekend
- ongedocumenteerd zijn

RISICOFACTOREN:
WIE IS EXTRA KWETSBAAR VOOR DIT GEWELD?

Eerder slachtoffer van (seksueel) geweld, personen met psychische klachten, LVB, afkomstig uit multiprobleem- of gebroken gezinnen, wees zijn, laag zelfbeeld, dakloosheid, voorgeschiedenis van geweld of trauma / jeugdhulpverleningsverleden, vluchteling/ongedocumenteerd zijn, armoede, makkelijk beïnvloedbaar zijn, leeftijd 12-24 jaar, LHBTI+.

FEITEN EN CIJFERS

- 88% van slachtoffers van mensenhandel bezoeken een zorgverlener tijdens de periode van uitbuiting
- Het aantal slachtoffers van mensenhandel in Nederland per jaar wordt geschat op 6.250
- Dat is 6x meer dan het aantal meldingen; veel mensenhandel is verborgen
- 56% van alle mensenhandel is binnenlands; het grote merendeel daarvan is seksuele uitbuiting
- Ongeveer de helft van de binnenlandse slachtoffers is minderjarig
- Buitenlandse slachtoffers komen vaak uit Roemenië, Polen, Hongarije, Bulgarije en Nigeria
- Er zijn ICD-10 codes voor mensenhandel
- Buitenlandse slachtoffers van mensenhandel hebben altijd recht op een tijdelijke verblijfsvergunning
- Er zijn opvangcentra voor buitenlandse en binnenlandse slachtoffers
- Zie voor organisaties www.wegwijzermensenhandel.nl

Deze factsheet is ontwikkeld door het landelijk Coördinatiecentrum tegen Mensenhandel (CoMensha) i.s.m. diverse ketenpartners

november 2018

MENSENHANDEL

AANDACHTSPUNTEN VOOR DIT TYPE GEWELD BIJ HET DOORLOPEN VAN DE 5 STAPPEN IN DE MELDCODE

Bij elke vorm van huiselijk geweld en kindermishandeling dien je als professional de meldcode te gebruiken. Algemene meldcode richtlijnen (zoals de 5 stappen) staan niet op deze factsheet beschreven – bezoek daarvoor de link. Wél staan hier aandachtspunten specifiek voor deze vorm van geweld:

- Mensenhandelaren zijn vaak familieleden/vrienden/partners/bekenden (bij 68% van alle gevallen van mensenhandel in Nederland!)
- Spreek iemand alleen! indien nodig via een externe tolk
- Ook mannen worden slachtoffer, ook van seksuele uitbuiting
- Let extra op eigen veiligheid, die van het slachtoffer en diens familie
- Mensen zien zichzelf niet altijd als slachtoffer
- Mensenhandel en mensensmokkel zijn niet hetzelfde!
- Buitenlandse slachtoffers hebben vaak weinig kennis van hun rechten en weinig vertrouwen in de politie

ADVIES/MELDEN

Voor advies, melden of het regelen van opvang en/of andere hulp, bel:

- Veilig Thuis **0800 20 00**
- CoMensha **033 44 81 186**
- een meldpunt loverboys
- een lokale zorgcoördinator
- meldarbeidsuitbuiting.nl

Bij acuut gevaar bel **112**

MEER INFORMATIE

Zie de bronnen.

ENGELSE VERTALING

Zie hier.
