# Supplementary material for: Lesser-known types of violence: Helping nurses and midwives to signal and act
Source: Int J Nurs Stud Adv. 2022 Sep 17;4:100098. doi: 10.1016/j.ijnsa.2022.100098 (PMC11080451; doi:10.1016/j.ijnsa.2022.100098)
Supplement: Supplementary file 1 [file mmc1.zip › Factsheets Dutch/mensenhandel-bronnen.pdf]

BRONNEN MENSENHANDEL

Dit bestand geeft een overzicht van organisaties die betrokken zijn geweest bij de ontwikkeling van de bijbehorende factsheet en van beschikbare achtergrondinformatie (bronnen).

BETROKKEN ORGANISATIES

In het maken van deze factsheet over Mensenhandel voor professionals in alle beroepen die een Meldcode huiselijk geweld en kindermishandeling hanteren, hebben de volgende organisaties input geleverd:

- CoMensha, het landelijk coördinatiecentrum tegen mensenhandel. Voor vragen en/of opmerkingen over de factsheet, kunt u emailen met de hoofdauteurs: Rik Viergever, r.viergever@comensha.nl en Sandra van den Berg, s.vandenbergh@comensha.nl
- Augeo Foundation
- Bureau Nationaal Rapporteur Mensenhandel en Seksueel Geweld tegen Kinderen
- Centrum tegen Kinderhandel en Mensenhandel (CKM)
- FairWork
- GGD GHOR Nederland
- Nederlands Jeugdinstituut (NJi)

BRONNEN

De volgende documenten en informatiebronnen geven meer informatie over de signalen van Mensenhandel, risicofactoren, en dingen om op te letten bij het doorlopen van de 5 stappen van de Meldcode huiselijk geweld en kindermishandeling:

- E-learning 'Herkenning Mensenhandel'. Het Rode Kruis.
- E-learning 'Signaleren van dwang en uitbuiting in de prostitutie'. SOAAIDS Nederland.
- E-learning 'signalering mensenhandel'. CCV.
- www.signalenkaart.nl
- Signalerings-protocol mensenhandel. Veilig Thuis en Moviera. 2016. Ede.
- Marjan Wijers en Marcia Albrecht. Handreiking Signalering Mensenhandel voor werkers in de gezondheidszorg. SOA aids, 2014.
- Signaleringsprotocol Loverboys. Steunpunt Huiselijk Geweld. 2014.
- Hoe signaleer je slachtoffers? Stappenplan voor professionals. Aanpak van loverboy/mensenhandel problematiek in de zorg voor jeugd. Nederlands Jeugdinstituut, 2015.
- Minderjarige jongens die hun lichaam exploiteren: Jongensprostitutie. Ruilseks signaleren, bespreekbaar maken en motiveren tot stoppen. MOVISIE, 2013.
- Slachtoffers loverboys: signalering. NJi.
- Slachtoffers loverboys: Risicoprofiel slachtoffers. NJi.
- Seksuele uitbuiting van jongens in Nederland. Paul van Gelder et al, SHOP Den Haag, Amsterdam, 2017.
- Mensenhandel: vijfde rapportage van de Nationaal Rapporteur. Bureau Nationaal Rapporteur Mensenhandel, Den Haag.
- Sue Berelowitz et al. "I thought I was the only one. The only one in the world" The Office of the Children's Commissioner's Inquiry into Child Sexual Exploitation In Gangs and Groups. Interim report. Office of the Childrens'Commissioner, November 2012.
- Anika Boersma et al. Signalenkaart mannelijke slachtoffers in de seksuele uitbuiting.
- Slachtoffermonitor mensenhandel 2012-2016. Nationaal Rapporteur Mensenhandel en Seksueel Geweld tegen Kinderen, 2017, Den Haag.
- Repetur, L.; Veenstra, J. Vrijbuiters uitgebuit. SWP, 2010.
- Raad voor de Kinderbescherming en Jeugdbescherming en Reclassering. Landelijke aanpak Raad voor de Kinderbescherming en Gecertificeerde Instellingen bij vermoedelijke slachtoffers van criminele uitbuiting.
- BNRM, 2012. Mensenhandel: met het oogmerk van orgaanverwijdering en gedwongen commercieel draagmoederschap. (Nederlands) (engels)
- Orgaanhandel en mensenhandel met het oogmerk van orgaanverwijdering: Een verkennend onderzoek naar de betrokkenheid van Nederland en Europa. Jessica de Jong. 2014, Politie, Woerden.
- Wat is mensenhandel? CoMensha. Bekeken op 2 oktober 2018.
- Oosterse teelt: Vietnamezen in de hennepteelt. Yvette Schoenmakers, Bo Bremmers, Anton van Wijk. 2012, Bureau Beke.
- Mensenhandel in en uit beeld II. Cijfermatige rapportage 2008-2012. Nationaal Rapporteur Mensenhandel en Seksueel Geweld tegen Kinderen, Den Haag, 2014.
- Mensensmokkel en mensenhandel. Amnesty international. Bekeken op 2 oktober 2018.
- CoMensha. Hoeveel slachtoffers van mensenhandel zijn er? Bekeken op 2 oktober 2018.
- CoMensha. Mensenhandel in Nederland: het beeld van 2016.
- Office on trafficking in persons. CDC Adds New Human Trafficking Data Collection Fields for Health Care Providers. Bekeken op 2 oktober 2018.
- Vreemdelingencirculaire 2000 (B). Bekeken op 2 juni 2018.
- Een veilige opvangplek vanwege mensenhandel. Slachtofferwijzer.nl. Bekeken op 2 oktober 2018.
- Wegwijzer mensenhandel. Bekeken op 2 oktober 2018. www.wegwijzermensenhandel.nl
