# Supplementary material for: Lesser-known types of violence: Helping nurses and midwives to signal and act
Source: Int J Nurs Stud Adv. 2022 Sep 17;4:100098. doi: 10.1016/j.ijnsa.2022.100098 (PMC11080451; doi:10.1016/j.ijnsa.2022.100098)
Supplement: Supplementary file 1 [file mmc1.zip › Factsheets Dutch/online-seksuele-intimidatie.pdf]

ONLINE SEKSUELE INTIMIDATIE

WAT IS ONLINE SEKSUELE INTIMIDATIE?

De European Union Agency for Fundamental Rights (FRA) definieert online seksuele intimidatie als: het ontvangen van ongewenste, beledigende, seksueel getinte e-mails of sms-berichten of het meemaken van ongepaste seksuele toenaderingen via sociale media of online chatrooms[1]. Er is sprake van een continuüm van gedragingen die verschillen in aard en ernst, waarbij de meningen variëren over wat wel of niet sociaal of juridisch toelaatbaar[2] is.

Vormen van online seksuele intimidatie zijn:

- Shame-sexting[3]: seksueel getint beeldmateriaal (vaak door afgebeelde persoon zelf gemaakt) wordt zonder toestemming van de afgebeelde persoon via sociale media met derden gedeeld[4]
- Grooming: het actief benaderen en verleiden door meerderjarigen van minderjarigen via internet en social networking sites, chatrooms of webcams met als doel seksueel getinte contacten
- Wraakporno: shame-sexting waarbij wraak het motief is, vaak na het verbreken van de relatie
- Sextortion: het (vaak onder valse voorwendselen) verkregen seksueel getint beeldmateriaal wordt gebruikt als chantagemiddel
- Online verspreiden van seksueel geweld: opnames of beelden van gedwongen seksuele handelingen worden online onder een groter publiek verspreid
- Ongevraagd toesturen of plaatsen van online seksueel getinte berichten: hier kan het gaan om seksueel getinte roddels, getreiter of pesten.

SIGNALEN: HOE KAN IK ZIEN DAT IEMAND SLACHTOFFER IS?

Slachtoffers ervaren vaak gevoelens van schaamte, waardoor zij een incident vaak niet melden. Angst voor reacties van naasten bemoeilijken de signalering. Slachtoffers hebben vaak last van psychische klachten zoals angst en stress[5], zij trekken zich terug en vermijden sociale activiteiten[6]. Daarnaast is er bij slachtoffers vaak sprake van afname in productiviteit op school/werk en bij jongeren van schoolverzuim[7].

RISICOFACTOREN: WIE IS EXTRA KWETSBAAR VOOR DIT GEWELD?

Jongeren en meisjes lopen een verhoogd risico met online seksuele intimidatie te worden geconfronteerd. Internationale onderzoeken wijzen erop dat meisjes en vrouwen in vergelijking met jongens en mannen tot drie keer zo vaak het doelwit zijn van seksueel getinte en ongewenste toenadering. Daarnaast wijzen verschillende onderzoeken uit dat jongeren die 'offline' het risico lopen op ongewenste seksuele toenadering, hier ook online vaker mee te maken zullen hebben[8].

AANDACHTSPUNTEN VOOR DIT TYPE GEWELD BIJ HET DOORLOPEN VAN DE 5 STAPPEN IN DE MELDCODE

- Spreek iemand bij voorkeur alleen
- Slachtoffers weten vaak niet waar ze terecht kunnen: geef daarom uitleg waar ze hulp kunnen krijgen
- Slachtoffers hebben mogelijk te kampen met schaamte en schuldgevoelens
- Slachtoffers zijn zich vaak niet bewust van hun rechten: geef hier dus goed uitleg over
- Bij minderjarigen: bespreek hoe ouders kunnen ondersteunen. Volg de beroepscode bij afweging of het geheim gehouden kan worden voor ouders.
- Voor advies kan altijd contact opgenomen worden met Veilig Thuis.
- In sommige gevallen valt online seksuele intimidatie wel onder de meldcode huiselijk geweld en kindermishandeling en in sommige gevallen niet: neem contact op met Veilig Thuis voor advies.

GEBRUIK BIJ ELKE VORM VAN HUISELIJK GEWELD EN KINDERMISHANDELING DE MELDCODE! ▶

FEITEN EN CIJFERS

Uit verschillende onderzoeken uitgevoerd in Nederland blijkt dat:

- 14% van de meisjes en 6% van de jongens in de leeftijd 12-25 jaar tenminste één ervaring met sexting heeft gehad die ze vervelend vonden[9].
- meiden ongeveer drie keer vaker dan jongens ongewenst seksueel zijn benaderd op internet[10].
- meiden seksueel getinte communicatie vaker vervelend vinden dan jongens[11].

ADVIES / MELDEN

Voor advies, melden en/of doorverwijzing naar opvang en/of andere hulp, bel:

- Veilig Thuis **0800 20 00**

of zie bovenstaande websites

Bij acuut gevaar bel **112**

MEER INFORMATIE

Zie de Bronnen, de factsheet online seksuele intimidatie van Atria en de volgende websites:

- Atria
- Centrum Seksueel Geweld
- Meldknop
- Politie

Engelse vertaling: Zie hier

Deze factsheet is ontwikkeld door Atria, Kennisinstituut voor Emancipatie en Vrouwengeschiedenis i.s.m. diverse ketenpartners.

november 2018
