# Supplementary material for: Lesser-known types of violence: Helping nurses and midwives to signal and act
Source: Int J Nurs Stud Adv. 2022 Sep 17;4:100098. doi: 10.1016/j.ijnsa.2022.100098 (PMC11080451; doi:10.1016/j.ijnsa.2022.100098)
Supplement: Supplementary file 1 [file mmc1.zip › Factsheets Dutch/online-seksuele-intimidatie-bronnen.pdf]

BRONNEN ONLINE SEKSUELE INTIMIDATIE

Dit bestand geeft een overzicht van organisaties die betrokken zijn geweest bij de ontwikkeling van de bijbehorende factsheet en van beschikbare achtergrondinformatie (bronnen).

BETROKKEN ORGANISATIES

In het maken van deze factsheet over online seksuele intimidatie voor professionals in alle beroepen die een meldcode huiselijk geweld en kindermishandeling hanteren, hebben de volgende organisaties input geleverd:

- Atria, Kennisinstituut voor Emancipatie en Vrouwengeschiedenis. Deze factsheet is geschreven door W. Krebbekx, J. Mejdoubi & R. Römkens. Contact: w.krebbekx@atria.nl
- GGD GHOR Nederland, Annette Duenk
- GGD GHOR Nederland, Sandra Hamming
- Radboud umc, Karin van Rosmalen - Nooijens
- Movisie, Hanan Nhass
- Fier - expertise en behandelcentrum op het terrein van geweld in afhankelijkheidsrelaties, Gerda de Groot

BRONNEN

Overzicht

- Een goed overzicht wordt gegeven in: factsheet online seksuele intimidatie. Renée Römkens, Jamila Mejdoubi, Tim de Jong. Juli 2017, Atria.

Referenties in de factsheet

1. European Union Agency for Fundamental Rights (2014). Violence against women: An EU-wide survey – Main results report. Luxemburg: Publications Office of the European Union
2. Zie https://www.om.nl/onderwerpen/sexting/ indien u meer wil weten over strafbaarheid van sexting
3. Als dit gebeurt in online-groepen die voor dit doel in het leven geroepen zijn spreekt men van exposing en expose-groepen
4. Een groot deel van de jongeren experimenteert met online seksueel gedrag zoals het sturen van seksueel getinte foto's en het merendeel van hen vindt dit normaal of plezierig. Misbruik vindt plaats als dergelijke foto's zonder toestemming verspreid worden. Zie ook: Naezer, M. (2018) From risky behaviour to sexy adventures: reconceptualising young people's online sexual activities, Culture, Health & Sexuality, 20:6, 715-729
5. Lenhart, A., Ybarra, M., Zickuhr, K., & Price-Feeney, M. (2016). Online Harassment, Digital Abuse, and Cyberstalking in America. New York: Data & Society Research Institute.
6. Halder, D., & Jaishankar, K. (2011). Cyber gender harassment and secondary victimization: A comparative analysis of the United States, the UK, and India. Victims & Offenders, 6(4), 386-398.
7. Kowalski, R. M., Giumetti, G. W., Schroeder, A. N., & Lattanner, M. R. (2014). Bullying in the digital age: A critical review and meta-analysis of cyberbullying research among youth. Psychological bulletin, 140(4), 1073.
8. Zetterström Dahlqvist, H. & Gillander Gådin, K. (2018). Online sexual victimization in youth: predictors and cross-sectional associations with depressive symptoms. European Journal of Public Health, cky102, https://doi.org/10.1093/eurpub/cky102. Livingstone, S., Davidson, J. & Bryce, J. (2017). Children's Online Activities, Risks and Safety: A Literature Review by the UKCCIS Evidence Group. London: LSE
9. Rutgers en Soa Aids Nederland (2017). Belangrijkste conclusies Seks onder je 25e 2017. Verkregen van http://seksonderje25e.nl/files/uploads/Seks%20onder%2025%202017%20 samenvatting%20(2).pdf.
10. Baumgartner, S., Valkenburg, P., & Peter, J. (2010). Unwanted online sexual solicitation and risky sexual online behavior across the lifespan. Journal of Applied Developmental Psychology, 31(6), 439-447
11. Kerstens, J., & De Graaf, H. (2012). Jongeren en online seksuele activiteiten. In: J. Kerstens en W. Stol (red.), Jeugd en Cybersafety. Online slachtoffer-en daderschap onder Nederlandse jongeren. Den Haag: Boom Lemma uitgevers

Websites

- www.act4respect.nl
- www.atria.nl
- www.centrumseksueelgeweld.nl
- www.helpwanted.nl
- www.meldknop.nl
- www.meldpunt-kinderporno.nl
- www.qpido.nl/thema/sexting-grooming
- www.rutgers.nl
- www.sense.info
- www.vraaghetdepolitie.nl/seks-en-loverboys/naaktfotos-en-filmpjes/wat-kan-ik-doen-als-mijn-naaktfoto-verspreid-is.html
- www.watchnederland.nl
