# Supplementary material for: Lesser-known types of violence: Helping nurses and midwives to signal and act
Source: Int J Nurs Stud Adv. 2022 Sep 17;4:100098. doi: 10.1016/j.ijnsa.2022.100098 (PMC11080451; doi:10.1016/j.ijnsa.2022.100098)
Supplement: Supplementary file 1 [file mmc1.zip › Factsheets Dutch/ouderenmishandeling-mantelzorg.pdf]

OUDERENMISHANDELING EN ONTSPOORDE MANTELZORG

GEBRUIK BIJ ELKE VORM VAN HUISELIJK GEWELD EN KINDER-MISHANDELING DE MELDCODE! ▶

Ouderenmishandeling is "al het handelen of het nalaten van handelen van al degenen die in een terugkerende persoonlijke of professionele relatie met de oudere (iemand van 65 jaar of ouder) staan, waardoor de oudere persoon lichamelijke en/of psychische en/of materiële schade lijdt en waarbij van de kant van de oudere sprake is van een vorm van gedeeltelijke of volledige afhankelijkheid."

Bij ontspoorde mantelzorg overschrijdt de mantelzorger de grens van goede zorg door overbelasting, onmacht, onkunde of onwetendheid. Een kenmerk van ontspoorde mantelzorg is het ontbreken van opzet.

VORMEN EN MOGELIJKE SIGNALEN VAN OUDERENMISHANDELING

Vorm	Mogelijke signalen
**Lichamelijke mishandeling** (o.a. slaan, duwen, schoppen, bedreigen of letsel toebrengen)	onverklaarde onderhuidse bloeduitstortingen, botbreuken, letsel van verschillende ouderdom, letsel past niet bij gerapporteerde toedracht
**Psychische mishandeling** (o.a. herhaaldelijk uitschelden en kleineren, beperken bewegingsvrijheid, bezoek ontzeggen, post achterhouden)	gedragsveranderingen, depressieve- en angstklachten
**Financieel misbruik** (o.a. veranderen van het testament, zonder toestemming verkopen van goederen, onbevoegd gebruik van iemands pinpas of creditcard, misbruik PGB)	gebrek aan standaard (medische) voorzieningen, verdwijnen van bezittingen, plotselinge schulden (zie ook de factsheet specifiek over financieel misbruik)
**Verwaarlozing** (o.a. onthouden van voeding, lichamelijke verzorging of toegang tot medische zorg, affectieve verwaarlozing	onbehandelde doorligplekken, ondervoeding, slechte lichamelijk hygiëne, smetplekken, onvoldoende toezicht op oudere
**Seksueel misbruik** (o.a. verbale grensoverschrijding, ongewenste seksuele handelingen verrichten met of in het bijzijn van de oudere)	onverklaarde onderhuidse bloeduitstortingen in genitaal gebied, bloedvlekken in ondergoed, onverklaarde seksueel overdraagbare aandoeningen

MEER INFORMATIE

Zie de bronnen.

FEITEN EN CIJFERS

- Gezondheidsmonitor 2016
  Uit deze monitor (over 12 maanden) blijkt dat psychische mishandeling het vaakst voorkomt en 4.0% van de ouderen van 65 jaar en ouder in Nederland hiermee te maken krijgt, gevolgd door financieel misbruik (1.1%) en verwaarlozing (0.6%).

- Regioplan 2018
  Deze interviewstudie (uitgevoerd in Rotterdam, Tilburg en Boxtel) concludeert dat 1 op de 20 thuiswonende ouderen van 65 jaar en ouder ooit te maken krijgt met ouderenmishandeling en 1 op de 50 op jaarbasis te maken heeft met ouderenmishandeling. De meest gerapporteerde vorm blijkt in beide gevallen financieel misbruik, gevolgd door psychische en lichamelijke mishandeling.

ADVIES / MELDEN

- Voor advies, melden en/of doorverwijzing naar opvang en/of andere hulp, bel: Veilig Thuis **0800 20 00**

ENGELSE VERTALING

Zie hier.

Deze factsheet is ontwikkeld door de Nederlandse Vereniging voor Klinische Geriatrie (NVKG) i.s.m. diverse ketenpartners.

november 2018

OUDERENMISHANDELING EN ONTSPOORDE MANTELZORG

SIGNALEN PASSENDE BIJ OVERBELASTE/ONTSPOORDE MANTELZORG

- Overbelasting en frustratie bij mantelzorger, zich bijv. uitend in grensoverschrijdend gedrag naar de oudere of anderen toe
- Compassiemoeheid bij de mantelzorger
- Weigeren hulp(verlening) voor oudere, isoleren van de oudere

MOGELIJKE RISICOFACTOREN OP OUDEREN-MISHANDELING

Bij slachtoffers:

- aanwezigheid cognitieve stoornissen
- psychische/psychiatrische aandoeningen
- een slechte fysieke gezondheid
- functionele beperkingen en afhankelijkheid van zorg
- beperkt sociaal netwerk of eenzaamheid
- lagere sociaal-economische status

Bij plegers:

- psychische-en psychiatrische aandoeningen
- alcohol- en middelengebruik
- overbelasting in de mantelzorgsituatie
- geweld in de familiegeschiedenis

EXTRA AANDACHTSPUNTEN

- Schaamte/taboe bij slachtoffer en pleger
- Bij elke vorm van huiselijk geweld en kindermishandeling dien je als professional de meldcode te gebruiken - dus ook bij een vermoeden van ouderenmishandeling of ontspoorde mantelzorg. Algemene meldcode richtlijnen (zoals de 5 stappen) staan niet op deze factsheet beschreven – bezoek daarvoor de link.

Deze factsheet is ontwikkeld door de Nederlandse Vereniging voor Klinische Geriatrie (NVKG) i.s.m. diverse ketenpartners.

november 2018
