# Supplementary material for: Lesser-known types of violence: Helping nurses and midwives to signal and act
Source: Int J Nurs Stud Adv. 2022 Sep 17;4:100098. doi: 10.1016/j.ijnsa.2022.100098 (PMC11080451; doi:10.1016/j.ijnsa.2022.100098)
Supplement: Supplementary file 1 [file mmc1.zip › Factsheets Dutch/ouderenmishandeling-mantelzorg-bronnen.pdf]

# BRONNEN OUDERENMISHANDELING EN ONTSPOORDE MANTELZORG

Dit bestand geeft een overzicht van organisaties die betrokken zijn geweest bij de ontwikkeling van de bijbehorende factsheet en van beschikbare achtergrondinformatie (bronnen).

## BETROKKEN ORGANISATIES

In het maken van deze factsheet over ouderenmishandeling en ontspoorde mantelzorg voor professionals in alle beroepen die een meldcode kindermishandeling en huiselijk geweld hanteren, hebben de volgende organisaties input geleverd:

- Nederlandse Vereniging voor Klinische Geriatrie (NVKG) (hoofdauteur factsheet namens de NVKG: Mw. Drs. M.E. van Houten, voorzitter landelijke richtlijn Vermoeden van ouderenmishandeling in het medisch-specialistische zorgdomein)
- Augeo
- GGD GHOR Nederland
- Movisie
- Veilig thuis
- Verenso

Voor vragen en/of opmerkingen over de factsheet, kunt u e-mailen met info@nvkg.nl

## BRONNEN

De volgende documenten en informatiebronnen geven meer informatie over de signalen van ouderenmishandeling en ontspoorde mantelzorg, risicofactoren, en dingen om op te letten bij het doorlopen van de 5 stappen van de meldcode kindermishandeling en huiselijk geweld:


- Actieplan 'Ouderen in veilige handen'. Brief aan de Tweede Kamer van 11 maart 2011, Kamerstukken II 2010/11, 29389, 30
- Comijs HC, Pot AM, Smit JH, et al. Elder abuse in the community: prevalence and consequences. J Am Geriatr Soc. 1998;46(7):885-8.PubMed PMID:9670877.
- Comijs, HC 1999, 'Elder mistreatment: prevalence, risk indicators and consequences', PhD, Vrije Universiteit Amsterdam.
- Gezondheidsmonitor Volwassenen en ouderen, 2016: https://bronnen.zorggegevens.nl/Bron?naam=Gezondheidsmonitor-Volwassenen-en-Ouderen%2C-GGD%E2%80%99en%2C-CBS-en-RIVM
- https://www.rijksoverheid.nl/binaries/rijksoverheid/documenten/rapporten/2015/06/15/ontspoorde-mantelzorg/ontspoorde-mantelzorg.pdf
- https://www.movisie.nl/sites/movisie.nl/files/publication-attachment/Factsheet%20Ouderenmishandeling%20III%20Financieel%20misbruik%20%5BMOV-287580-0.2%5D.pdf
- https://www.movisie.nl/sites/movisie.nl/files/publication-attachment/Factsheet%20ontspoorde%20mantelzorg%20%5BMOV-695455-1.1%5D.pdf
- https://www.movisie.nl/sites/movisie.nl/files/publication-attachment/Signalenkaart-Ontspoorde-Mantelzorg%20%5BMOV-458810-1.1%5D.pdf
- https://www.movisie.nl/sites/movisie.nl/files/publication-attachment/Factsheet-Ouderenmishandeling-Algemeen%20%5BMOV-225838-0.7%5D.pdf
- Dong X, Simon MA. Elder abuse as a risk factor for hospitalization in older persons. JAMA Intern Med. 2013;173(10):911-7. doi: 10.1001/jamainternmed.2013.238. PubMed PMID: 23567991.
- Dong XQ. Elder Abuse: Systematic Review and Implications for Practice. J Am Geriatr Soc. 2015;63(6):1214-38. doi: 10.1111/jgs.13454. Epub 2015 Jun 11. Review. PubMed PMID: 26096395.
- Handleiding ouderenmishandeling mei 2017, Samen Veilig Midden-Nederland, Utrecht
- Johannesen M, LoGiudice D. Elder abuse: a systematic review of risk factors in community-dwelling elders. Age Ageing. 2013;42(3):292-8. doi:10.1093/ageing/afs195. Epub 2013 Jan 22. Review. PubMed PMID: 23343837.
- McCausland B, Knight L, Page L, et al. A systematic review of the prevalence and odds of domestic abuse victimization among people with dementia. Int Rev Psychiatry. 2016;28(5):475-484. Epub 2016 Aug 26. PubMed PMID:27564566.
- Naughton C, J. Drennan, M.P. Treacy, A. Lafferty, I. Lyons, A. Phelan, S. Quin, O'Loughlin, A., Delaney, L. (2010) Abuse and Neglect of Older People in Ireland.
- Publicatie ouderen Veilig thuis, een beschrijving van de nieuwe werkwijze rondom ouderenmishandeling. Veilig thuis Utrecht. 2015,
- Prevalentieonderzoek naar aard en omvang van ouderenmishandeling, Regioplan, 2018
- Pillemer K, Burnes D, Riffin C, et al. Elder Abuse: Global Situation, Risk Factors, and Prevention Strategies. Gerontologist. 2016;56Suppl2:S194-205. doi: 10.1093/geront/gnw004. Review. PubMed PMID: 26994260; PubMed Central PMCID: PMC5291158.
- SCP-rapport Ouderenmishandeling in Nederland, 2015
- Yon Y, Mikton CR, Gassoumis ZD, Wilber KH. Elder abuse prevalence in community settings: a systematic review and meta-analysis. Lancet Glob Health. 2017 Feb;5(2):e147-e156. doi: 10.1016/S2214-109X(17)30006-2. Review. PubMed PMID: 28104184
