# Supplementary material for: Lesser-known types of violence: Helping nurses and midwives to signal and act
Source: Int J Nurs Stud Adv. 2022 Sep 17;4:100098. doi: 10.1016/j.ijnsa.2022.100098 (PMC11080451; doi:10.1016/j.ijnsa.2022.100098)
Supplement: Supplementary file 1 [file mmc1.zip › Factsheets Dutch/partnergeweld.pdf]

(EX-)PARTNERGEWELD

GEBRUIK BIJ ELKE VORM VAN HUISELIJK GEWELD EN KINDERMISHANDELING DE MELDCODE! ▶

WAT IS (EX-)PARTNERGEWELD?

(Ex-)Partnergeweld is iedere vorm van geweld tussen partners of ex-partners. Ook een (constante) geweldsdreiging wordt beschouwd als (ex-)partnergeweld. Hierbij gaat het om alle vormen van geweld; fysiek, emotioneel/psychisch, seksueel, financieel of online, en om stalking.

We kunnen verschillende categorieën van partnergeweld onderscheiden, waaronder:

1. Veelvoorkomend partnergeweld ('common couple violence')

Deze vorm van geweld ontstaat vaak uit onmacht, door verlies van controle en regie. De pleger kan zowel de man als de vrouw zijn. Er kan verschil zijn in ernst van het geweld en de consequenties hiervan verschillen per persoon. Consequenties zijn mogelijk groter voor vrouwen dan voor mannen (Daru et al., 2016).

2. Intiem terrorisme ('intimate terrorism')

Eenzijdig (dreigen met) ernstig geweld van doorgaans de man. Het gaat om opzettelijk geweld, partnerterreur, gericht op controle over de partner.

SIGNALEN

Signalen bij slachtoffers die kunnen wijzen op partnergeweld kunnen onderscheiden worden in:

- **lichamelijke signalen**
  Herhaaldelijke verwondingen: blauwe plekken, snij-, bijt- of hoofdwonden, kneuzingen, fracturen (met name neus, pijpbeenderen, ribben), ontwrichtingen (met name kaak en schouder), brandwonden, verlies van tanden, schedelletsel en genitale beschadigingen.

- **signalen op psychosomatisch vlak**
  Bijvoorbeeld depressiviteit, trillen, vaak hoofdpijn of maagpijn, vermoeidheid, angst, slaapstoornissen, hyperventilatie, hartkloppingen.

- **gedragsmatige signalen**
  Bijvoorbeeld moeite met oogcontact, afspraken steeds uitstellen, een onderdanige houding, onduidelijke hulpvragen, schrikachtig, vrouwen die altijd worden vergezeld door hun partner, en/of consulten op het laatste moment afzeggen, anderzijds meer dan gemiddeld gebruikmaken van zorgverleners (huisarts), meer gebruik van slaapmiddelen en pijnstillers, meer dan gemiddeld op zoek zijn naar een verklaring voor klachten.

Zie hier voor een volledig overzicht van signalen, hier zijn ook kindsignalen opgenomen.

RISICOGROEPEN

Partnergeweld komt binnen alle lagen en groeperingen van de bevolking voor. Stressfactoren verhogen de kans op problemen en geweld in de relatie, daarom zien we in de praktijk meer partnergeweld bij stellen die te maken hebben met stressoren zoals armoede, werkloosheid of ander problemen. Vaak gaat het om een complex van factoren die (ex-)partnergeweld tot gevolg hebben.

FEITEN EN CIJFERS

Volgens een rapport van de FRA (European Union Agency for Fundamental Rights, 2014) dat zich uitsluitend richtte op vrouwelijke slachtoffers, heeft 9% van de Nederlandse vrouwen sinds het 15e jaar te maken gehad met lichamelijk of seksueel geweld door de huidige partner en 25% door een ex-partner:

- Eén op de vijf vrouwen is ooit fysiek mishandeld door een partner of ex-partner.
- 11% van de vrouwen maakte seksueel geweld door een partner of ex-partner mee.
- 3% van de vrouwen heeft het afgelopen jaar uit angst voor geweld het eigen huis wel eens vermeden.

Ruim 60% van het huiselijk geweld betreft (ex-)partnergeweld. Vrouwen (60%) worden vaker slachtoffer van (ex-)partnergeweld dan mannen (40%). Het geweld dat mannen plegen is vaker structureel van aard dan het geweld dat vrouwen plegen. Zowel voor mannen als vrouwen is sekse specifieke hulpverlening en opvang mogelijk.

Deze factsheet is ontwikkeld door Movisie i.s.m. diverse ketenpartners. november 2018

(EX-)PARTNERGEWELD

RISICOFACTOREN VOOR (EX-)PARTNERGEWELD

We kunnen de volgende (groepen van) risicofactoren onderscheiden:

1. Machtsverschil en afhankelijkheid. Relationele afhankelijkheid, economische afhankelijkheid, verblijfsstatus, zorgafhankelijkheid of een combinatie daarvan.
2. Genderongelijkheid.
3. Gebrek aan weerbaarheid en aan sociale vaardigheden.
4. Isolement.
5. Problematische echtscheiding.
6. Stress als gevolg van een veelvoud aan problemen in het gezin, zoals geldzorgen en schulden, problemen op het werk of juist problemen door gebrek aan werk, slechte huisvesting.
7. Drank- en drugsgebruik.
8. Eerdere mishandeling (in eerder relaties of in de huidige relatie).
9. Gedragsproblemen op jonge leeftijd.
10. Cultureel bepaalde tolerantie ten aanzien van geweld.
11. Persoonlijke problemen, waaronder psychische of psychiatrische problematiek en/of een verstandelijke beperking.
12. Zwangerschap (hierbij is het risico op partnergeweld hoog).
13. Zelf geweld meegemaakt in het verleden of getuige geweest zijn van partnergeweld bij ouders (meisjes hebben dan meer kans om slachtoffer te zijn en jongens pleger).

BELANGRIJKE AANDACHTSPUNTEN

Bij elke vorm van huiselijk geweld en kindermishandeling dien je als professional de meldcode te gebruiken. Algemene meldcode richtlijnen (zoals de 5 stappen) staan niet op deze factsheet beschreven – bezoek daarvoor de link.

Wanneer je een vermoeden hebt van partnergeweld, voer dan altijd de Kindcheck uit. Hierover is een afzonderlijk factsheet verschenen.

RELATIEWIJS

Hoe weet je als professional of gedrag in een relatie over een grens gaat? Wat te doen als er sprake lijkt te zijn van (ex-)partnergeweld? Hoe maak je het bespreekbaar? RelatieWijs is een hulpmiddel bij het doorlopen van alle stappen van de meldcode. RelatieWijs helpt bij het verzamelen van signalen en het beoordelen van relationeel (grensoverschrijdend) gedrag vanuit objectiveerbare criteria. Deze criteria ondersteunen het bespreekbaar maken van het gesignaleerde gedrag: ze maken het mogelijk gedrag concreet te benoemen en laten zien wanneer nader onderzoek nodig is. RelatieWijs hanteert zeven criteria om na te gaan of relationeel gedrag 'gezond' is of grensoverschrijdend: wederzijdse toestemming, vrijwilligheid, gelijkwaardigheid, zelfbeschikking, passend bij de context, zelfrespect en respect voor de ander.

Zie voor meer informatie: www.movisie.nl/publicatie/relatiewijs

MEER INFORMATIE

Zie deze bronnen.

ADVIES / MELDEN

Voor advies, melden en/of doorverwijzing naar opvang en/of andere hulp, bel:

- Veilig Thuis **0800 20 00**. Dit is gratis en 24/7 bereikbaar. Bellen kan ook anoniem. Bellen kan ook alleen voor advies (zonder te melden).

Bij acuut gevaar bel **112**

ENGELSE VERTALING

Zie hier.

Deze factsheet is ontwikkeld door Movisie i.s.m. diverse ketenpartners. november 2018
