# Supplementary material for: Lesser-known types of violence: Helping nurses and midwives to signal and act
Source: Int J Nurs Stud Adv. 2022 Sep 17;4:100098. doi: 10.1016/j.ijnsa.2022.100098 (PMC11080451; doi:10.1016/j.ijnsa.2022.100098)
Supplement: Supplementary file 1 [file mmc1.zip › Factsheets Dutch/partnergeweld-bronnen.pdf]

BRONNEN (EX-)PARTNERGEWELD

Dit bestand geeft een overzicht van organisaties die betrokken zijn geweest bij de ontwikkeling van de bijbehorende factsheet en van beschikbare achtergrondinformatie (bronnen).

BETROKKEN ORGANISATIES

In het maken van deze factsheet over (ex-)partnergeweld voor professionals in alle beroepen die een meldcode huiselijk geweld en kindermishandeling hanteren, hebben de volgende organisaties input geleverd:

- Movisie. Voor vragen en/of opmerkingen over de factsheet, kunt u emailen met de hoofdauteur: Wilma Schakenraad, w.schakenraad@movisie.nl
- Danielle van den Heuvel – Sterk Huis
- Suzanne Tan – Bureau Tangram
- Willemijn Krebbekx – Atria
- Karin van Rosmalen-Nooijens – Radboud UMC
- Marga Nicolaij – Veilig Thuis
- Mireille Bartelomij – Veilig Thuis

BRONNEN

De volgende documenten en informatiebronnen geven meer informatie over de signalen van (ex-)partnergeweld, risicofactoren, en dingen om op te letten bij het doorlopen van de 5 stappen van de meldcode huiselijk geweld en kindermishandeling:

- Daru, S., J. Mejdoubi, K. de Vaan en A. Visser (2016), Huiselijk geweld verklaard vanuit genderperspectief: Literatuurstudie. Amsterdam/Utrecht: Atria, Movisie en Regioplan.
- Janssen, H., Wentzel, W., & Vissers, B. (2015). Basisboek huiselijk geweld: signaleren, melden en aanpakken. 3e herz. druk. Bussum: Couthinho.
- Janssens, K., Visser, A., & Oosten, N. van (2017). RelatieWijs. Beoordelen en aanpakken van (ex-)partnergeweld. Utrecht: Movisie.
- Römkens, R., de Jong, T., & Harthoorn, H. (2014). Geweld tegen vrouwen. Europese onderzoeksgegevens in Nederlandse context. Amsterdam: Atria.
- R. Römkens, A. van den Brink en T. de Jong, (2018 Welk geweld telt? Opvattingen van Nederlanders over partnergeweld. Amsterdam: Atria, kennisinstituut voor emancipatie en vrouwengeschiedenis en Blijf Groep 2018.
- Steketee, M., R. Römkens, T. Pels, K. Lünnemann, E. Smits van Waesberghe, J. Mak, J. Mejdoubi & H. Harthoorn (2016). Preventie van intergenerationeel geweld Nederland en EU. Verkenning van wat werkt. Kennisinstituut voor emancipatie en vrouwengeschiedenis: Atria en Utrecht: Verweij-Jonker Instituut.
- Veen, H.C.J. van der, & Bogaerts, S. (2010). Huiselijk geweld in Nederland: overkoepelend synthese-rapport van het vangst-hervangst-, slachtoffer- en daderonderzoek 2007-2010. Den Haag, WODC.
- Verwijs, R., & Lünnemann, K. (2012). Partnergeweld. Achtergrond en risicofactoren. Utrecht: Verwey-Jonker Instituut.
- Signalenkaarten van Kadera, zie https://signalenkaart.nl/, Deze bevatten signalen die kunnen wijzen op huiselijk geweld, zowel bij slachtoffers als plegers. De signalenkaart is ingedeeld naar leeftijd met daarbinnen risicofactoren in acht leefgebieden. Een van de kaarten is gericht op volwassen plegers en slachtoffers.
