# Supplementary material for: Lesser-known types of violence: Helping nurses and midwives to signal and act
Source: Int J Nurs Stud Adv. 2022 Sep 17;4:100098. doi: 10.1016/j.ijnsa.2022.100098 (PMC11080451; doi:10.1016/j.ijnsa.2022.100098)
Supplement: Supplementary file 1 [file mmc1.zip › Factsheets Dutch/pesten.pdf]

PESTEN

WAT IS PESTEN?

Pesten is stelselmatige agressief gedrag waarbij één of meer personen in een machtspositie proberen een andere persoon schade toe brengen. Pesten kan plaatsvinden in de vorm van fysiek, verbaal of psychologisch gedrag. Stelselmatig uitsluiten valt ook onder pesten. Pesten vindt vaak plaats in een groep, bijvoorbeeld in de klas, of digitaal, maar kan ook plaatsvinden op andere plekken zoals de sportclub.

Pesten kan leiden tot allerlei negatieve gevolgen, waaronder psychosomatische klachten, angsten en depressie, een laag zelfvertrouwen, eenzaamheid, slechte schoolprestaties en schoolverzuim. Sommige van deze gevolgen kunnen doorwerken tot in de volwassenheid. Naast de pestslachtoffers kunnen ook pesters problemen krijgen in hun ontwikkeling. Het onaangepaste gedrag en gebrek aan empathie vergroot de kans op probleemgedrag op latere leeftijd.

SIGNALEN: HOE KAN IK ZIEN DAT IEMAND SLACHTOFFER IS?

Hieronder staan signalen die kunnen samengaan met gepest worden. **Professionals kunnen vragen naar deze klachten en naar het sociaal functioneren om het gesprek over pesten aan te gaan:**

- Psychosomatische klachten zoals buikpijn, hoofdpijn, bedplassen, eczeem
- Angstig of depressief gedrag
- Teruggetrokken, stil, sub-assertief gedrag
- Geen plezier in school, niet naar school willen
- Geen vrienden hebben

RISICOFACTOREN: WIE HEEFT MEER KANS OM SLACHTOFFER TE ZIJN?

Hier staan kenmerken beschreven die kinderen en jongeren meer kwetsbaar maken voor pestgedrag:

- Kinderen die angstig, depressief, teruggetrokken of verlegen zijn
- Kinderen die motorisch onhandig zijn of stotteren
- Kinderen met overgewicht, ADHD of autisme
- Kinderen met geen of weinig goede vrienden
- LHBTQ-jongeren
- Kinderen die qua leerniveau buiten het gemiddelde vallen (HB of juist minderbegaafd of ZB)
- Kinderen van ouders met een agressieve/autoritaire opvoedstijl zijn vaker pester

5-STAPPENPLAN BIJ PESTEN

Bij kinderen waar het vermoeden bestaat van een pestprobleem dient expliciet te worden gevraagd of het kind gepest wordt. Bij een kind dat individuele aandacht behoeft, kan een individuele interventie worden gestart. Pesten is vaak een probleem van de groep en moet daar ook meestal worden opgelost, ook bij cyberpesten. Het is daarom van belang om ouders en de school te betrekken zodat zij de verantwoordelijkheid bij het oplossen van het pestprobleem oppakken. In de richtlijn voor Jeugdgezondheidszorg wordt het volgende 5-stappenplan aanbevolen:

1. Inschatting behoeftes van het kind
2. Overleg met ouders
3. Overleg met school, sportclub of andere organisatie
4. Verwijzen voor individuele hulp
5. Follow-up

FEITEN EN CIJFERS

Jaarlijks is ongeveer 10% van de leerlingen in het basisonderwijs slachtoffer van pesten. Op het middelbaar onderwijs is dit ongeveer 8%.

Meisjes worden ongeveer even vaak gepest als jongens.

Homoseksuele, lesbische en transgender jongeren worden tweemaal zo vaak gepest en krijgen ook veel vaker te maken met gewelddadig gedrag. Verschillende schoolprogramma's zijn effectief in het verminderen van pesten.

MEER INFORMATIE

Zie de bronnen en:

- www.pestweb.nl
- de JGZ-richtlijn Pesten https://www.ncj.nl/richtlijnen/alle-richtlijnen/richtlijn/pesten
- Vermande, M., van der Meulen, M. & Reijntjes, A. (Red.) Pesten op school, Boom Uitgevers Amsterdam.
- voor interventies: https://www.uu.nl/sites/default/files/eindrapport-wat-werkt-tegen-pesten.pdf

ENGELSE VERTALING

Zie hier

Deze factsheet is ontwikkeld door TNO i.s.m. diverse ketenpartners. november 2018
