# Supplementary material for: Lesser-known types of violence: Helping nurses and midwives to signal and act
Source: Int J Nurs Stud Adv. 2022 Sep 17;4:100098. doi: 10.1016/j.ijnsa.2022.100098 (PMC11080451; doi:10.1016/j.ijnsa.2022.100098)
Supplement: Supplementary file 1 [file mmc1.zip › Factsheets Dutch/pesten-bronnen.pdf]

BRONNEN PESTEN

Bij alle vormen van huiselijk geweld en kindermishandeling moet de meldcode huiselijk geweld en kindermishandeling volgens de wet toegepast worden door de groepen professionals die in de wet over de meldcode staan benoemd. Pesten valt niet onder de definitie van huiselijk geweld of kindermishandeling en de meldcode hoeft hierbij dus niet toegepast te worden. Echter, de meldcode *mag* hier wel bij gebruikt worden! En omdat het belangrijk is dat professionals (bijv huisartsen) pesten wel kunnen signaleren en de juiste stappen kunnen nemen, is de factsheet die hoort bij dit bronnenbestand opgesteld.

Dit bestand geeft een overzicht van organisaties die betrokken zijn geweest bij de ontwikkeling van de factsheet en van beschikbare achtergrondinformatie (bronnen).

BETROKKEN ORGANISATIES

In het maken van deze factsheet over pesten hebben de volgende organisaties input geleverd:

- TNO. Voor vragen en/of opmerkingen over de factsheet, kunt u emailen met de hoofdauteur: Minne Fekkes, minne.fekkes@tno.nl .
- Augeo, Edith Geurts
- Radboud umc, Karin van Rosmalen-Nooijens
- GGD GHOR, Sandra Hamming
- Veilig Thuis / VVAK, Juliette Heetman

BRONNEN

De volgende documenten en informatiebronnen geven meer informatie over de signalen van pesten, risicofactoren, en dingen om op te letten:

- www.pestweb.nl
- de JGZ-richtlijn Pesten https://www.ncj.nl/richtlijnen/alle-richtlijnen/richtlijn/pesten
- voor interventies: https://www.uu.nl/sites/default/files/eindrapport-wat-werkt-tegen-pesten.pdf
- Vermande, M., van der Meulen, M. & Reijntjes, A. (Red.) Pesten op school, Boom Uitgevers Amsterdam.
