# Supplementary material for: Lesser-known types of violence: Helping nurses and midwives to signal and act
Source: Int J Nurs Stud Adv. 2022 Sep 17;4:100098. doi: 10.1016/j.ijnsa.2022.100098 (PMC11080451; doi:10.1016/j.ijnsa.2022.100098)
Supplement: Supplementary file 1 [file mmc1.zip › Factsheets Dutch/radicalisering.pdf]

RADICALISERING

WAT IS RADICALISERING?

Sinds halverwege de 20e eeuw zijn er vele studies gedaan naar radicalisering en extremisme in relatie tot verschillende vormen van gewelddadig extremisme. Een belangrijk punt wat hieruit is voorgekomen is dat het een moeilijk fenomeen is om in een eenduidig proces te beschrijven. Vanuit elke politieke of religieuze ideologie[1] kunnen gewelddadige vormen voortkomen. Sommigen organiseren zich tot terroristische[2] of paramilitaire groeperingen, anderen tot kleine activistische groepjes, maar soms ook tot gevaarlijke eenlingen. Daarnaast uit niet elke vorm van radicalisering zich in geweld. Mensenrechtenactivisten als Martin Luther King werden ook als radicaal bestempeld. Bepaalde ecologische of religieuze levensstijlen kunnen als radicaal of als polariserend worden gezien, maar zijn niet automatisch illegaal. Wat radicaal is kan daarom heel subjectief zijn. In deze beschrijving wordt ervan uitgegaan dat de radicalisering zich beweegt naar een vorm van (gewelddadig) extremisme. Er wordt in het algemeen gesproken over vier hoofdvormen van extremisme: religieus extremisme, links-extremisme, rechts-extremisme, en milieu-extremisme. Enkele voorbeelden van hedendaagse definities van radicalisering en extremisme zijn:

"Radicalisering is een grillig verlopend proces. Een jongere die geraakt wordt door radicaal gedachtegoed drijft af van de democratie en groeit toe naar gewelddadig extremisme. Een jongere raakt geïnspireerd en gaat geloven in een extremistisch wij-zij-verhaal. Hij gaat daar steeds meer persoonlijke consequenties aan verbinden en verheerlijkt geweld om zijn idealen te bereiken. Radicalisering is een mogelijk spoor van ontsporing." (Stichting School & Veiligheid)

"Extremisme is het fenomeen waarbij personen of groepen bij het streven naar hun idealen bewust over de grenzen van de wet gaan. Extremisme is iets anders dan activisme. Activisten komen soms luidruchtig maar geweldloos voor hun mening uit en houden zich aan de grenzen van de wet. Activisme wordt extremisme als er sprake is van het bewust plegen van strafbare feiten, zoals bedreigingen en vernieling." (NCTV)[3]

Er zijn meerdere beschrijvingen van radicaliseringsprocessen, waarbij de een nadruk legt op psychologische factoren en de ander op sociologische of economische factoren. Omdat radicalisering een grillig en dynamisch fenomeen is, zeker bij jongeren, zijn deze beschrijvingen niet op alle situaties toepasbaar.[4] Er is dus geen checklist om radicalisering te herkennen.

WELKE SIGNALEN KUNNEN DUIDEN OP RADICALISERING?

Omdat er geen checklist is voor radicalisering is het lastig om aan te geven bij welke signalen er nu daadwerkelijk sprake is van zorgelijk gedrag. Vaak zien we dat verschillende factoren invloed kunnen hebben op het radicaliseringsproces en dat het proces niet volgens een vast patroon verloopt. Er zijn allerlei signalen die op zichzelf geen duidelijkheid geven, maar die samen een bepaalde situatie wel zorgelijk maken[5]:

- De persoon isoleert zich van familie en oude vrienden, en gaat om met een nieuw netwerk.
- De persoon bezoekt zorgelijke social mediagroepen, websites of bijeenkomsten.
- De persoon is intensief met een nieuwe ideologische of religieuze identiteit bezig.[6]
- De persoon gebruikt wij-tegen-zij-termen, leest anderen de les, vooral betreffende politieke onderwerpen.

CIJFERS

Er zijn geen cijfers over geradicaliseerde personen, wel over geradicaliseerde personen die (gewelddadig) extremistische strafbare feiten hebben gepleegd. Er zijn bijvoorbeeld enkele honderden personen, vooral jongeren, die zijn uitgereisd of hebben geprobeerd uit te reizen naar strijdgebieden.[9] Ook zijn er veroordelingen voor extremistisch activisme zoals bedreigingen, vernielingen, en brandstichting. Het percentage mensen die strafbare feiten plegen vanwege extremisme is niet hoog, maar vaak komen deze personen uit extremistische netwerken die uit meerdere personen bestaan die zelf (nog) niet overgegaan zijn tot (gewelddadig) extremistische activiteiten. Het aantal mensen die op een zorgelijk manier zijn geradicaliseerd ligt dus veel hoger dan het aantal mensen die een bekend strafbaar feit hebben gepleegd.

MEER INFORMATIE

Zie de bronnen. Verder:

PlatformJEP biedt antwoord op de belangrijkste vragen en geeft een overzicht van verschillende informatiebronnen: https://www.platformjep.nl.

Deze factsheet is ontwikkeld door het Landelijk Steunpunt Extremisme (LSE) i.s.m. diverse ketenpartners.

- Extreem spijbelgedrag of het beëindigen van studie, werk, sport, of andere hobby's.
- Er heeft onlangs een ingrijpende gebeurtenis plaatsgevonden in het leven van de persoon; dit kan variëren van een verhuizing of een wisseling van school of verlies van werk tot een overlijden.

RISICOFACTOREN: WIE HEEFT MEER KANS OM TE RADICALISEREN?

Door de verschillende vormen van radicalisering is er geen vast profiel, maar gevoeligheid voor radicalisering wordt vergroot wanneer er sprake is van psychopathologie (psychosociale problemen, licht verstandelijke beperking, gedragsproblemen) en instabiele leefomstandigheden (ontwricht gezin/relatie, werk-en huisvestingsproblemen, drank en/of drugsgebruik). Daarnaast komt radicalisering vooral voor bij jongeren. Een mogelijke factor hierin is de hersenontwikkeling betreffende rationale afwegingen die tijdens de adolescentiefase zich langzamer ontwikkelt dan andere hersengebieden. Hierdoor hebben jongeren tussen ongeveer 14 en 23 jaar moeite met het maken van weloverwogen keuzes, abstract denken, en de consequenties van hun keuzes te overzien.[7]

STAPPENPLAN BIJ SIGNALEN VAN RADICALISERING

Blijf goed in contact en wijs de persoon niet af op gedrag en uitspraken. Dit kan het gedrag versterken en ervoor zorgen dat een persoon zich (verder) terugtrekt. Omgang met niet-radicale personen is een belangrijke beschermfactor, en het aanwezige niet-radicale netwerk geeft ook de mogelijkheid voor andere personen en professionals om met de persoon in contact te kunnen komen. Als er sprake is van radicalisering zal er naar meerdere factoren en manieren van aanpak gekeken moeten worden om de persoon te bereiken en beschermfactoren aan te brengen. Daarbij zijn er wel gesprekstechnieken en interventies die u toe zou kunnen passen.[8]

ADVIES

Medewerkers van het Landelijk Steunpunt Extremisme (LSE) kunnen u te woord staan en samen met u de situatie bespreken. Zij kunnen u adviseren wat u zelf kunt doen en aangeven waar professionele hulp en mogelijke meldingen bij instanties nodig zijn. Blijkt de situatie inderdaad zorgelijk te zijn, dan kunnen de casemanagers u helpen. Zij zijn gespecialiseerd in het ondersteunen en bijstaan van personen, families en professionals die te maken hebben met radicalisering. Website: www.landelijksteunpuntextremisme.nl.
Telefoon: **088 - 20 80 080**.

ENGELSE VERTALING

Zie hier.

Deze factsheet is ontwikkeld door het Landelijk Steunpunt Extremisme (LSE) i.s.m. diverse ketenpartners.
