# Supplementary material for: Lesser-known types of violence: Helping nurses and midwives to signal and act
Source: Int J Nurs Stud Adv. 2022 Sep 17;4:100098. doi: 10.1016/j.ijnsa.2022.100098 (PMC11080451; doi:10.1016/j.ijnsa.2022.100098)
Supplement: Supplementary file 1 [file mmc1.zip › Factsheets Dutch/radicalisering-bronnen.pdf]

BRONNEN RADICALISERING

Bij alle vormen van huiselijk geweld en kindermishandeling moet de meldcode huiselijk geweld en kindermishandeling volgens de wet toegepast worden door de groepen professionals die in de wet over de meldcode staan benoemd. Radicalisering valt niet onder de definitie van huiselijk geweld of kindermishandeling en de meldcode hoeft hierbij dus niet toegepast te worden. Echter, de meldcode mag hier wel bij gebruikt worden! En omdat het belangrijk is dat professionals (bijv docenten of huisartsen) radicalisering wel kunnen signaleren en de juiste stappen kunnen nemen, is de factsheet die hoort bij dit bronnenbestand opgesteld.

Dit bestand geeft een overzicht van organisaties die betrokken zijn geweest bij de ontwikkeling van de factsheet en van beschikbare achtergrondinformatie (bronnen).

BETROKKEN ORGANISATIES

In het maken van deze factsheet over Radicalisering voor professionals in alle beroepen die een meldcode huiselijk geweld en kindermishandeling hanteren, hebben de volgende organisaties input geleverd:

- Het Landelijk Steunpunt Extremisme (LSE), ook lid van het Platform JeP, heeft de leiding gehad in het ontwikkelen van deze factsheet. Voor vragen en/of opmerkingen over de factsheet kunt u emailen met: info@hetlse.nl
- Stichting School en Veiligheid
- Veilig Thuis

BRONNEN

De volgende documenten en informatiebronnen geven meer informatie over de signalen van Radicalisering, risicofactoren, en dingen om op te letten bij het doorlopen van de 5 stappen van de meldcode huiselijk geweld en kindermishandeling:

1 Een ideologie is een pakket aan ideeën over wereldbeeld en zingeving die zich focust op mensbeeld en de inrichting van de maatschappij, zoals fascisme, anarchisme, nationalisme, (neo)liberalisme, socialisme, Islamisme, Christendemocratie, en conservatisme. Een ideologie is daarmee wel altijd politiek gericht, maar is niet altijd een antiliberale of gewelddadige beweging.

2 "Terrorisme is het uit ideologische motieven plegen van op mensenlevens gericht geweld, dan wel het aanrichten van maatschappij-ontwrichtende zaakschade, met als doel maatschappelijke ondermijning en destabilisatie te bewerkstelligen, de bevolking ernstige vrees aan te jagen of politieke besluitvorming te beïnvloeden." CT-Strategie, NCTV, https://www.nctv.nl/

3 Zie voor een overzicht PlatformJEP: https://www.platformjep.nl/documenten/vragen-en-antwoorden/wat-zijn-de-definities-van-radicalisering-extremisme-en-polarisatie. NCTV: https://www.nctv.nl/organisatie/ct/terrorismebestrijding/extremisme.

4 Voor voorbeelden van radicaliseringsprocessen, zie bijvoorbeeld: Understanding Radicalisation: Review of Literature, Dzhekova et al., Center for the Study of Democracy, 2016, http://www.csd.bg/artShow.php?id=17560. Triggerfactoren in het Radicaliseringsproces, Feddes et al., Expertise-unit Sociale Stabiliteit en Universiteit van Amsterdam, 2015, https://www.socialestabiliteit.nl/professionals/documenten/publicaties/2015/10/13/triggerfactoren-in-het-radicaliseringsproces. Ontstaan van radicalisering, Wienke en Ramadan, NJI, 2011, https://www.nji.nl/nl/Producten-en-diensten/Publicaties/.

5 Voor een overzicht van signalen en factoren, zie: Triggerfactoren Radicalisering, https://www.socialestabiliteit.nl/professionals/triggerfactoren. En Herkenning en duiding, Wienke en Ramadan, NJI, 2011, https://www.nji.nl/nl/Download-NJi/Publicatie-NJi/.

6 Bekering naar een nieuwe religie of andere levensbeschouwelijke identiteit kan als polariserend worden ervaren, maar is op zichzelf geen signaal van radicalisering.

7 Voor een discussie hierover, zie: NJI, https://www.nji.nl/nl/Kennis/Dossier/Radicalisering/Achtergrond/Ontwikkeling, en, https://www.nji.nl/nl/Download-NJi/Publicatie-NJi/Pol_Rad_Ontstaan_radicalisering.pdf.

8 Voor voorbeelden, zie: NJI, https://www.nji.nl/nl/Kennis/Dossier/De-rol-van-jeugdhulp-bij-het-tegengaan-van-radicalisering-van-jongeren.

9 Deze cijfers gaan voornamelijk over uitreizigers die vanaf 2013 zich aansloten bij Jihadistische groeperingen in Syrië en Irak, maar uitreizen naar strijdgebieden bestond al voor 2013 en geldt ook voor personen die zich aansloten bij bijvoorbeeld Koerdische of andere militair activistische groeperingen. Van de enkele honderden uitreizigers naar Syrië en Irak zijn nu enkele tientallen teruggekeerd. Bij deze terugkeerders kan er sprake zijn van personen die door hun ervaring gedesillusioneerd zijn geraakt met de extremistische ideologie en netwerk, en anderen die nog steeds actief zijn. Deze factsheet richt zich vooral op het signaleren van radicalisering bij personen die nog geen strafbare feiten hebben gepleegd, en waarbij radicalisering wordt bezien in de volle breedte en niet alleen vanuit religieus extremistische vormen als Jihadisme.
