# Supplementary material for: Lesser-known types of violence: Helping nurses and midwives to signal and act
Source: Int J Nurs Stud Adv. 2022 Sep 17;4:100098. doi: 10.1016/j.ijnsa.2022.100098 (PMC11080451; doi:10.1016/j.ijnsa.2022.100098)
Supplement: Supplementary file 1 [file mmc1.zip › Factsheets Dutch/seksueel-geweld.pdf]

SEKSUEEL GEWELD TEGEN VOLWASSENEN DOOR ONBEKENDEN

WAT IS SEKSUEEL GEWELD?

Seksueel geweld is wat in de wet als aanranding en verkrachting is gedefinieerd, dat wil zeggen penetratie (verkrachting) of andere seksuele handelingen (aanranding) waarbij geweld is gebruikt, dreiging met geweld of gebruik is gemaakt van een situatie of toestand waardoor iemand niet in staat was te weigeren (bijv. door middelengebruik) (Van Beek & Van Berlo, 2015).[1]
In het Istanbul Verdrag wordt de volgende definitie gehanteerd: Het zonder wederzijds goedvinden op seksuele wijze vaginaal, anaal of oraal met een lichaamsdeel of object binnendringen van het lichaam van een ander. Het gaat er hierbij dus om dat de seksuele handeling zonder toestemming van de ander wordt verricht.

WAAROM IS HET MOEILIJK SIGNALEN VAN SEKSUEEL GEWELD TE HERKENNEN?

Het is moeilijk om signalen van seksueel geweld te herkennen omdat de meeste signalen aspecifiek zijn: signalen als ongewoon gedrag, opvallend, maar ook te stil of juist te druk gedrag, verwijzen niet specifiek naar één oorzaak. De combinatie van signalen kan echter wel een vermoeden versterken. Slachtoffers onthullen het seksueel geweld doorgaans niet, ook niet bij hun huisarts.

RISICOGROEPEN EN RISICOFACTOREN

Seksueel geweld (waarbij het in dit factsheet gaat om seksueel geweld buiten de partnerrelatie) kan iedereen treffen, maar er zijn groepen die een groter risico lopen. Dit geldt voor vrouwen en lesbische vrouwen, homoseksuele mannen, biseksuelen, transgender- en intersekse-personen (LHBTI's), maar ook voor mensen met negatieve jeugdervaringen. Eerdere ervaringen met seksueel geweld vormen een risicofactor: bijna een kwart van de mannen en bijna de helft van de vrouwen die seksueel geweld hebben meegemaakt, maken dit later in hun leven nog een keer mee (revictimisatie). Daarnaast kunnen maatschappelijke normen (zoals maatschappelijk gangbare genderstereotiepe opvattingen, breed gedragen dubbele moraal, algemene negatieve opvattingen over vrouwen en meisjes en traditionele rolopvattingen) de kans op seksueel geweld vergroten (Act4Respect).
Ten slotte is middelengebruik een risicofactor: bij 31% van de vrouwen en 23% van de mannen was er tijdens het meemaken van seksueel geweld alcohol of drugs in het spel, bij het slachtoffer en/of de pleger (De Graaf & Wijsen, 2017).

HULPVERLENING

Bij een recente aanranding of verkrachting (bij voorkeur maximaal zeven dagen geleden) wordt slachtoffers geadviseerd zich te melden bij het Centrum Seksueel Geweld (CSG) via 0800-0188. Het CSG biedt in 16 regio's medische, forensische en psychologische hulp direct na seksueel geweld. Een team van medici, politie, en hulpverleners werkt hierin samen. Het is daarmee een integraal zorgsysteem, zoveel mogelijk op één locatie. Het slachtoffer krijgt medische hulp, sporen kunnen worden veilig gesteld, er kan aangifte bij de politie worden gedaan (maar dat hoeft niet) en zo nodig start psychologische hulpverlening na een periode

1 In dit factsheet wordt de terminologie gevolgd van het Whitepaper seksuele grensoverschrijding en seksueel geweld van Movisie en Rutgers (2015). Deze wijkt af van de terminologie die Veilig Thuis gebruikt.

FEITEN EN CIJFERS

Melden en aangifte doen

Uit onderzoek van het Centraal Bureau voor de Statistiek (CBS) blijkt dat veruit de meeste seksuele delicten niet worden gemeld aan de politie (In 2011 9 procent). Als de dader een onbekende is, wacht het slachtoffer gemiddeld 10 dagen met melden; als het een bekende is daarentegen duurt het gemiddeld 8 maanden (Nationaal Rapporteur Mensenhandel en Seksueel Geweld tegen Kinderen, 2014).

Relatie met pleger

Bij ca. 30% van de plegers van seksueel geweld bij vrouwen gaat het om de eigen partner of ex-partner, ca. 15% van de plegers worden in het uitgaansleven ontmoet. Bij ca. 19% van de vrouwelijke slachtoffers gaat het om een onbekende pleger. Mannen worden vooral slachtoffer van seksueel geweld waarbij een vriend of vriendin de pleger is, waarbij het niet hoeft te gaan om een (voormalige) intieme relatie (28%). Partners of ex-partners zijn in 18,5% van de gevallen de plegers van seksueel geweld tegen mannen en bij 17,4% van de mannelijke slachtoffers gaat het om een onbekende pleger (De Haas, 2012). LHBTI's maken relatief veel seksueel geweld mee (Act4Respect). Een op de vijf homo- en biseksuele mannen heeft ooit een vorm van seksueel geweld meegemaakt, tegenover 6% van de heteromannen. Ook lesbische vrouwen

SEKSUEEL GEWELD TEGEN VOLWASSENEN DOOR ONBEKENDEN

van watchfull waiting (monitoren van het verwerkingsproces). Het slachtoffer hoeft het verhaal niet vaker dan nodig te vertellen (Bicanic, Engelhard & Sijbrandij, 2014).

Gaat het niet om recent seksueel geweld, dan kan het slachtoffer contact opnemen met de politie of met Slachtofferhulp. De hulplijn Verbreek de Stilte is een onderdeel van onder andere Slachtofferhulp Nederland en telefonisch of via chat bereikbaar. Hier bieden sociale professionals slachtoffers een luisterend oor; ook zoeken zij naar passende hulp. Slachtofferhulp biedt daarnaast ook juridische, praktische en emotionele ondersteuning; de hulpverlening is gratis en in de buurt.

Slachtoffers kunnen ook contact opnemen met Veilig Thuis.

MEER INFORMATIE

Zie de bronnen.
Over geweld in de (ex-)partnerrelatie, over seksueel grensoverschrijdend gedrag tussen jongeren onderling en over online seksuele intimidatie zijn afzonderlijke factsheets ontwikkeld.
Zie voor meer informatie over (verschillende vormen van) seksueel geweld deze pagina.

zijn relatief vaak slachtoffer van seksueel geweld: 37% van de lesbische vrouwen heeft ervaringen met een vorm van seksueel geweld (De Haas, 2014; Rutgers WPF, 2013).

Overige cijfers

- Bijna driekwart (73%) van alle Nederlandse vrouwen is ooit seksueel geïntimideerd;
- Eén op de tien vrouwen is ooit in haar leven verkracht (FRA, 2014);
- 11% van alle Nederlandse vrouwen heeft seksueel geweld meegemaakt door een (ex-) partner;
- 12% van alle Nederlandse vrouwen heeft seksueel geweld gemaakt door iemand die niet de partner was.

ADVIES

Het Centrum Seksueel Geweld is het expertise centrum van Nederland voor slachtoffers van seksueel geweld in de acute fase (<7 dagen). Bij het CSG werkt een team van artsen, verpleegkundigen, politie, psychologen, maatschappelijk werkers en seksuologen samen om slachtoffers van aanranding en verkrachting specialistische zorg te geven. Bel **0800-0188**. Wanneer het niet gaat om recent seksueel geweld, neem dan contact op met Slachtofferhulp, **0900-0101**, of met Veilig Thuis: **0800 – 2000**. Bij acuut gevaar bel **112**.

ENGELSE VERTALING

Zie hier.

Deze factsheet is ontwikkeld door Movisie i.s.m. diverse ketenpartners in de strijd tegen seksueel geweld.
