# Supplementary material for: Lesser-known types of violence: Helping nurses and midwives to signal and act
Source: Int J Nurs Stud Adv. 2022 Sep 17;4:100098. doi: 10.1016/j.ijnsa.2022.100098 (PMC11080451; doi:10.1016/j.ijnsa.2022.100098)
Supplement: Supplementary file 1 [file mmc1.zip › Factsheets Dutch/seksueel-geweld-bronnen.pdf]

BRONNEN SEKSUEEL GEWELD TEGEN VOLWASSENEN DOOR ONBEKENDEN

Bij alle vormen van huiselijk geweld en kindermishandeling moet de meldcode huiselijk geweld en kindermishandeling volgens de wet toegepast worden door de groepen professionals die in de wet over de meldcode staan benoemd. Seksueel geweld door onbekenden valt niet onder de definitie van huiselijk geweld of kindermishandeling en de meldcode hoeft hierbij dus niet toegepast te worden. Echter, de meldcode *mag* hier wel bij gebruikt worden! En omdat het belangrijk is dat professionals (bijv docenten of huisartsen) seksueel geweld door onbekenden wel kunnen signaleren en de juiste stappen kunnen nemen, is de factsheet die hoort bij dit bronnenbestand opgesteld.

Dit bestand geeft een overzicht van organisaties die betrokken zijn geweest bij de ontwikkeling van de factsheet en van beschikbare achtergrondinformatie (bronnen).

BETROKKEN ORGANISATIES

In het maken van deze factsheet over seksueel geweld door onbekenden hebben de volgende organisaties input geleverd:

- Movisie. Voor vragen en/of opmerkingen over de factsheet, kunt u emailen met de hoofdauteur: Wilma Schakenraad, w.schakenraad@movisie.nl
- Veilig Thuis
- Atria, kennisinstituut voor emancipatie en vrouwengeschiedenis

BRONNEN

De volgende documenten en informatiebronnen geven meer informatie over de signalen van seksueel geweld door onbekenden, risicofactoren, en dingen om op te letten:

Websites

- Kennisdossier seksuele grensoverschrijding van Rutgers: https://www.rutgers.nl/feiten-en-cijfers/kennisdossiers/kennisdossier-seksuele-grensoverschrijding
- www.seksueelgeweld.info/ . Website voor slachtoffers van seksueel geweld, en voor betrokkenen en verwijzers. Zie hierop ook de sociale kaart met hulpaanbod in Nederland voor slachtoffers en plegers van seksueel geweld.
- Dossier seksueel geweld op https://www.huiselijkgeweld.nl/dossiers/seksueel-geweld
- www.act4respect.nl (website wordt eind 2018 gelanceerd).

Publicaties

- Berlo van, W. & Beek I. van (2015). Whitepaper Seksuele grensoverschrijding en seksueel geweld. Feiten en cijfers Utrecht: Rutgers en Movisie.
- Bicanic, I., Jongh, A., de, Lagro-Janssen, T. & Leusink, P. (2016). Centrum seksueel geweld voor acute slachtoffers. Huisarts & Wetenschap. 59 (6), 265-267.
- Centraal Bureau voor de Statistiek (2012). Integrale Veiligheidsmonitor 2011. Landelijke rapportage. Den Haag: Centraal Bureau voor de Statistiek.
- European Union Agency for Fundamental Rights (FRA) (2014). Violence against women: an EU-wide survey. Luxemburg: publications Office of the European Union.
- Graaf, H. de , & Wijsen, C. (red.) (2017). Seksuele gezondheid in Nederland. Utrecht: Rutgers i.s.m. RIVM. Zie: https://www.rutgers.nl/sites/rutgersnl/files/PDF-Onderzoek/Seksuele_Gezondheid_in_NL_2017_23012018.pdf
- Haas, S. de (2012). Seksueel grensoverschrijdend gedrag onder jongeren en volwassenen in Nederland. In Tijdschrift voor Seksuologie, 36(2), 136-145.
- Haas, S. de (2014). Seksueel geweld en seksuele grensoverschrijding. In H. de Graaf, B. Bakker & C. Wijsen, Een wereld van verschil. Seksuele gezondheid van LHBT's in Nederland 2013. Utrecht: Rutgers WPF.Kühl, M., Schakenraad, W., & Beek, I. van (2017). Werken met volwassen slachtoffers van seksueel geweld. In: Höing, M., & Janssen, J., Boer, A., & Liebregts, M. (red.). Bespreekbaar maken van seksualiteit en intimiteit. Handboek voor professionals in zorg en welzijn. Bussum: Coutinho.
- Nationaal Rapporteur Mensenhandel en Seksueel Geweld tegen Kinderen (2014). Op goede grond. De aanpak van seksueel geweld tegen kinderen. Den Haag: Nationaal Rapporteur.
- Römkens, R., Jong, T. de en Harthoorn, H. (2014). Geweld tegen vrouwen. Europese onderzoeksgegevens in de Nederlandse context. Amsterdam: Atria.
- Rutgers WPF (2013). Wat maakt het verschil? Diversiteit in de seksuele gezondheid van LHBT's, een verkenning. Utrecht: Rutgers WPF
