# Supplementary material for: Lesser-known types of violence: Helping nurses and midwives to signal and act
Source: Int J Nurs Stud Adv. 2022 Sep 17;4:100098. doi: 10.1016/j.ijnsa.2022.100098 (PMC11080451; doi:10.1016/j.ijnsa.2022.100098)
Supplement: Supplementary file 1 [file mmc1.zip › Factsheets Dutch/stalking-belaging.pdf]

# STALKING

GEBRUIK BIJ ELKE VORM VAN HUISELIJK GEWELD EN KINDERMISHANDELING DE MELDCODE! ▶

Deze factsheet geeft slechts een beknopt overzicht. Het verdient de aanbeveling om de publicatie "Als u wordt gestalkt" van de Politie in samenwerking met Movisie te lezen.

## WAT IS STALKING?

Stalking (ook wel belaging genoemd) is iemand bewust stelselmatig lastigvallen, waardoor die persoon zich niet meer veilig voelt. Het gaat vaak om onzichtbare en moeilijk te vatten terreur; een opeenstapeling van op zichzelf niet altijd strafbare gedragingen. Voorbeelden van stalking zijn achtervolgen, dreigen met geweld, ongewenste telefoontjes en berichten sturen of op naam van iemand anders dingen bestellen. Stalking kan thuis, elders en/of online plaatsvinden. Stalking is strafbaar in Nederland.[1]
Stalking is per definitie een vorm van geweld omdat het een grote inbreuk maakt op de veiligheidsbeleving in en op het leven van slachtoffers.
Een stalker kan een (intieme) relatie met het slachtoffer hebben (gehad), maar dit hoeft niet.

## SIGNALEN: HOE KAN IK ZIEN DAT IEMAND SLACHTOFFER IS?

Er wordt voortdurend en opzettelijk inbreuk gemaakt op de persoonlijke levenssfeer van het slachtoffer met bijvoorbeeld het doel het slachtoffer ergens toe te dwingen (bijvoorbeeld te reageren of de relatie te herstellen), bang te maken of te hinderen.[2]
Stalking betreft een patroon van gedrag waarbij ieder incident op zichzelf niet heel heftig en/of strafbaar hoeft te zijn, maar de combinatie van al die incidenten dat wel is. Voor dienstverleners is het belangrijk zich te realiseren dat stalking gevolgen heeft voor het gevoel van veiligheid en het welbevinden van het slachtoffer. Er is sprake van spanning en stress. Het slachtoffer wordt belemmerd in zijn dagelijks functioneren.

Om het risico in te schatten dat de gestalkte persoon loopt, kunnen verschillende vragen behulpzaam zijn (zie p4 en 5 van de publicatie "Als u wordt gestalkt").

## AANDACHTSPUNTEN VOOR DIT TYPE GEWELD BIJ HET DOORLOPEN VAN DE 5 STAPPEN IN DE MELDCODE

Bij elke vorm van huiselijk geweld en kindermishandeling dien je als professional de meldcode te gebruiken. Algemene meldcode richtlijnen (zoals de 5 stappen) staan niet op deze factsheet beschreven – bezoek daarvoor de link. Wél staan hier aandachtspunten specifiek voor deze vorm van geweld:

- Een belangrijke vuistregel: neem het gevoel van het slachtoffer serieus: als zij écht bang zijn, dan is er daadwerkelijk een risico tot gevaar/dreiging.
- Er is een aantal vuistregels voor slachtoffers zelf in wat zij kunnen doen wanneer ze gestalkt worden: zie p7 en 8 van de publicatie "Als u wordt gestalkt".
- Het is belangrijk om bewijs te verzamelen.[3]
- Stem af met de Politie. Bel in een acute gevaarlijke of bedreigende situatie direct de politie.
- Slachtoffers van stalking hebben recht op bescherming. Zo kan bijvoorbeeld de officier van justitie of rechter een gebiedsverbod en/of contactverbod opleggen aan de stalker. Lees: meer over rechten in geval van stalking.[4]
- Betrek buren, familieleden, vrienden en collega's die kunnen opletten en ondersteunen.

## MEER INFORMATIE

Zie de bronnen en de publicatie "Als u wordt gestalkt" van de Politie in samenwerking met Movisie.

## ADVIES / MELDEN

Voor advies, melden en/of doorverwijzing naar opvang en/of andere hulp, bel:

- Veilig Thuis **0800 20 00**

Bij acuut gevaar bel **112**

Indien nodig kan een mobiel alarmsysteem (AWARE) of een plaatsing binnen de opvang gerealiseerd worden.

- Slachtofferhulp Nederland (0900 01 01) biedt praktische steun maar kan ook verwijzen voor hulp bij verwerking of lotgenotengroepen.

## ENGELSE VERTALING

Zie hier.



Deze factsheet is ontwikkeld door Slachtofferhulp Nederland i.s.m. diverse ketenpartners. november 2018
