# Supplementary material for: Lesser-known types of violence: Helping nurses and midwives to signal and act
Source: Int J Nurs Stud Adv. 2022 Sep 17;4:100098. doi: 10.1016/j.ijnsa.2022.100098 (PMC11080451; doi:10.1016/j.ijnsa.2022.100098)
Supplement: Supplementary file 1 [file mmc1.zip › Factsheets Dutch/stalking-belaging-bronnen.pdf]

BRONNEN STALKING

Dit bestand geeft een overzicht van organisaties die betrokken zijn geweest bij de ontwikkeling van de bijbehorende factsheet en van beschikbare achtergrondinformatie (bronnen).

BETROKKEN ORGANISATIES

In het maken van deze factsheet over stalking voor professionals in alle beroepen die een meldcode huiselijk geweld en kindermishandeling hanteren, hebben de volgende organisaties input geleverd:

- Slachtofferhulp Nederland. Voor vragen en/of opmerkingen over de factsheet, kunt u emailen met de hoofdauteur: Franck Wagemakers, f.wagemakers@slachtofferhulp.nl
- AROSA: Magda Vogelezang
- Nationale politie: Cleo Brandt en Berdien Zuurveen
- Veilig Thuis: Inge Sauvé

BRONNEN

De volgende documenten en informatiebronnen geven meer informatie over de signalen van stalking, risicofactoren, en dingen om op te letten bij het doorlopen van de 5 stappen van de meldcode huiselijk geweld en kindermishandeling:

1. https://www.slachtofferhulp.nl/gebeurtenissen/stalking/
2. https://www.movisie.nl/publicatie/u-wordt-gestalkt "Als u wordt gestalkt", Movisie
3. https://www.slachtofferhulp.nl/gebeurtenissen/stalking/bewijs-verzamelen-gestalkt/
4. https://www.slachtofferhulp.nl/gebeurtenissen/stalking/rechten/#bekijk-uw-rechten.
