# Supplementary material for: Lesser-known types of violence: Helping nurses and midwives to signal and act
Source: Int J Nurs Stud Adv. 2022 Sep 17;4:100098. doi: 10.1016/j.ijnsa.2022.100098 (PMC11080451; doi:10.1016/j.ijnsa.2022.100098)
Supplement: Supplementary file 1 [file mmc1.zip › Factsheets Dutch/vechtscheiding.pdf]

KINDEREN IN EEN CONFLICTSCHEIDING

GEBRUIK BIJ ELKE VORM VAN HUISELIJK GEWELD EN KINDERMISHANDELING DE MELDCODE! ▶

WAT IS EEN CONFLICTSCHEIDING?

We spreken van een conflictscheiding als kinderen door hevige en complexe conflicten tussen ouders in de knel komen, en de inzet zijn geworden van de strijd tussen de ouders. Voor het welzijn van kinderen maakt de samenlevingsvorm van de ouders niet uit (zie het bronnen bestand voor een gedetailleerdere definitie).

Iedere scheiding zet het gezinssysteem onder druk. Ouders lijden ook onder een conflictscheiding: ze kunnen gevoelens van schaamte en schuld ervaren, en het gevoel hebben te falen als ouder. Ouders nemen hun ouderpositie niet meer in. Daarnaast zijn ouders in een scheiding kwetsbaarder voor het ontwikkelen van psychische- en verslavingsproblematiek, waar hun kinderen dan weer aan blootgesteld worden.

Gevolgen van een conflictscheiding voor kinderen kunnen o.a. zijn:

- Psychische klachten, zoals angst, depressie en posttraumatische stress.
- Emotionele verwaarlozing door één of beide ouders (met als gevolg eenzaamheid).
- Loyaliteitsconflicten: ouders doen een tegengesteld beroep op het kind. Kinderen hebben het gevoel niet trouw te kunnen/mogen zijn aan beide ouders, wat tot interne conflicten leidt.
- School en ontwikkelproblemen zoals internaliserende; bijv. teruggetrokken gedrag en externaliserende gedragsproblemen; bijv. moeite met gezag, problemen in de omgang met leeftijdsgenoten, verslechterde schoolprestaties en (seksueel) risicogedrag.
- Intergenerationele transmissie, wat betekent dat kinderen zelf ook in een problematische relatie terecht komen.
- Parentificatie, waarbij het kind zich verantwoordelijk voelt (of wordt gemaakt) voor het welzijn van de ouders.

SIGNALEN: HOE KAN IK ZIEN OF IEMAND SLACHTOFFER IS?

Kinderen in een conflictscheiding zijn altijd slachtoffer. De signalen voor kinderen in een conflictscheiding zijn vergelijkbaar met de signalen bij kindermishandeling en huiselijk geweld. Signalen worden vaak pas zichtbaar als er al gevolgen zijn door de scheiding, zoals hierboven beschreven. Is er daarom sprake van een scheiding en zijn er kinderen betrokken? Ben dan altijd alert.

Een specifiek en laat signaal van een conflictscheiding is ouderafwijzing of oudervervreemding, waarbij het kind een van de ouders volledig afwijst en als het ware partij kiest.

RISICOFACTOREN: WIE HEEFT MEER KANS OM SLACHTOFFER TE ZIJN?

Ieder kind met scheidende ouders heeft kans om in een conflictscheiding terecht te komen en hierdoor klachten te ontwikkelen. Toch zijn er enkele risico's te benoemen op het ontwikkelen van problemen bij kinderen in een scheidingssituatie.

- Het voorkomen van eerder en/of ander geweld en kindermishandeling.
- Verslaving of psychische of psychiatrische problematiek bij (een van) de scheidende ouders.
- Psychisch geweld in het gezin, in het bijzonder psychologische oorlogsvoering tussen de ouders; ernstige en langdurige ouderlijke conflicten.
- Als het kind een slechte band heeft met (een van) de ouder(s) of stiefouder(s).

FEITEN EN CIJFERS

Een conflictscheiding wordt ook wel een complexe scheiding of een vechtscheiding genoemd.

In 2017 zijn er 18.178 echtscheidingen waarbij minderjarige kinderen betrokken zijn. Een scheiding heeft altijd invloed op een kind, waarbij de gevolgen per leeftijd verschillen.

Jaarlijks zijn er ongeveer 3500 kinderen betrokken bij een conflictscheiding van hun ouders in Nederland.

Ongeveer de helft betreft een scheiding van niet gehuwde partners, waardoor deze groep moeilijker in beeld is.

De kinderombudsman heeft in 2014 geschat dat ongeveer 16.000 kinderen in Nederland lijden onder een conflictscheiding.

Ongeveer 50% van de kinderen in een conflictscheiding ontwikkelt posttraumatische stress klachten. Ongeveer een derde van de kinderen in een conflictscheiding blijft klachten houden.

ADVIES / MELDEN

Voor advies, melden en/of doorverwijzing naar opvang en/of andere hulp, bel:

- Veilig Thuis op **0800-2000**.

Bij acuut gevaar bel **112**

ENGELSE VERTALING

Zie hier.

Deze factsheet is ontwikkeld door het Radboudumc, afdeling eerstelijnsgeneeskunde, gender in transmural care i.s.m. diverse ketenpartners.

november 2018

KINDEREN IN EEN CONFLICTSCHEIDING

- Veranderingen zoals verhuizing, schoolverandering en nieuwe partners.
- Financiële problemen (in het algemeen achteruitgang in SES en in het bijzonder als er sprake is van armoede)

AANDACHTSPUNTEN

Bij elke vorm van huiselijk geweld en kindermishandeling dien je als professional de meldcode te gebruiken. Algemene meldcode richtlijnen (zoals de 5 stappen) staan niet op deze factsheet beschreven – bezoek daarvoor de link. Wél staan hier aandachtspunten specifiek voor deze vorm van geweld.

Bij een conflictscheiding spelen er veel verschillende belangen, waarbij ouders de impact op kinderen vaak onderschatten.
Is er mogelijk sprake van een conflictscheiding?

- Praat met de ouders. U kunt de ouders bijvoorbeeld vertellen wat u bij het kind ziet of wat u hen ziet doen.
- Praat met het kind. Kinderen verwerken de scheiding beter als zij mensen om zich heen hebben die hen het gevoel geven er niet alleen voor te staan en hen uitleggen wat er aan de hand is op hun eigen niveau. Denk hierbij ook aan het inschakelen van een Kindbehartiger.
- Een conflictscheiding is een vorm van kindermishandeling. Overleg daarom met Veilig Thuis wat je het beste kunt doen.
- Beide ouders met ouderlijk gezag hebben recht op informatie over hun kind tot 16 jaar, maar dit is geen recht op informatie over de andere ouder. Als hulpverlener is het extra belangrijk om daarom neutraal te blijven en de informatieverstrekking duidelijk maar summier te houden.
- Hulpverleners kunnen de belangen van zowel ouders als kinderen in de gaten houden. De beste resultaten worden behaald bij een samenwerking tussen het juridische - en het hulpverleningsdeel. Hierbij is er aandacht voor het welzijn van ouders en kinderen, en wordt er tevens overeenstemming bereikt over de juridische conflictpunten mbv mediators, advocaten en rechters.
- Er zijn verschillende erkende interventies bij scheidingen. Tevens is het goed kennis te nemen van wat werkt. Voor ouders kan laagdrempelige informatie zinvol zijn, bijvoorbeeld met de nieuwe ScheidingsATLAS. Kinderen kunnen ook preventief ondersteund worden met bijvoorbeeld groepsprogramma's.

MEER INFORMATIE

Zie de bronnen.
Lees ook de Factsheet over de Kindcheck voor gerelateerde informatie.
Verder:

- www.nji.nl
- www.richtlijnenjeugdhulp.nl
- www.vooreenveiligthuis.nl

Deze factsheet is ontwikkeld door het Radboudumc, afdeling eerstelijnsgeneeskunde, gender in transmural care i.s.m. diverse ketenpartners.

november 2018
