# Supplementary material for: Lesser-known types of violence: Helping nurses and midwives to signal and act
Source: Int J Nurs Stud Adv. 2022 Sep 17;4:100098. doi: 10.1016/j.ijnsa.2022.100098 (PMC11080451; doi:10.1016/j.ijnsa.2022.100098)
Supplement: Supplementary file 1 [file mmc1.zip › Factsheets Dutch/vechtscheiding-bronnen.pdf]

BRONNEN KINDEREN IN EEN CONFLICTSCHEIDING

Dit bestand geeft extra informatie over de definitie van een conflictscheiding, een overzicht van organisaties die betrokken zijn geweest bij de ontwikkeling van de bijbehorende factsheet, en een overzicht van beschikbare achtergrondinformatie (bronnen).

EXTRA INFORMATIE

De definitie van een conflict- of vechtscheiding loopt uiteen. Wij gaan uit van de volgende indeling:
Er is sprake van een conflictscheiding als de communicatie tussen ouders over opvoeding en omgang conflictueus verloopt, waardoor de kinderen langdurig geen ontspannen contact kunnen onderhouden met een of beide ouders, lijdensdruk ervaren of er parentificatie ontstaat en het kind in zijn ontwikkeling bedreigd wordt. **Het komt regelmatig voor dat ouder(s) ontkennen dat er sprake is van een conflictscheiding.**
Je zou hierbij kunnen denken aan:

- **Psychische mishandeling**
  Bijvoorbeeld: Ouders voeren handelingen uit die geestelijk en/of emotioneel kwetsend zijn voor de andere ouder waar kinderen getuige van zijn (uitschelden, bedreigen, manipuleren, diskwalificeren, vernederen, kleineren of pesten); ouders laten zich tegen de kinderen negatief uit over de andere ouder; kinderen mogen geen contacten onderhouden met de andere ouder: ouderverstoting
- **Pedagogische verwaarlozing**
  Bijvoorbeeld: Er bestaat onduidelijkheid en onenigheid over de wijze waarop ouders invulling geven aan de verzorging en opvoeding van hun kinderen. Ouders hebben geen overeenstemming over de hulpverlening, die nodig is om onveiligheid weg te nemen of die noodzakelijk is voor de ontwikkeling van het kind.
- **Getuige van huiselijk geweld**
  Bijvoorbeeld: Kinderen zijn getuige van conflicten, die mogelijk gepaard gaan met fysiek geweld, tussen ouders. Er zijn veel verschillende manieren van blootstelling: variërend van directe blootstelling (het direct getuige zijn door geweld te zien of te horen) tot indirecte blootstelling (het zien en ervaren van de gevolgen van het geweld, zoals een blauw oog of de spanning in huis).

BETROKKEN ORGANISATIES

In het maken van deze factsheet over Kinderen in een vechtscheiding voor professionals in alle beroepen die een meldcode huiselijk geweld en kindermishandeling hanteren, hebben de volgende organisaties input geleverd:

- Radboudumc, afdeling eerstelijnsgeneeskunde, gender in transmural care. Voor vragen en/of opmerkingen over de factsheet, kunt u emailen met de hoofdauteur: Karin van Rosmalen-Nooijens, Karin.vanRosmalen-Nooijens@radboudumc.nl
- HMC Westeinde, Hesther Diderich
- TNO, Fieke Pannebakker
- GGD GHOR Nederland, Sandra Hamming
- Augeo, Marga Haagmans
- Sterk Huis, Cindy de Rijke

BRONNEN

De volgende documenten en informatiebronnen geven meer informatie over de signalen van Kinderen in een conflictscheiding, risicofactoren, en dingen om op te letten bij het doorlopen van de 5 stappen van de meldcode huiselijk geweld en kindermishandeling:

- https://www.nji.nl/Mogelijke-effecten-van-echtscheiding-op-het-kind
- https://www.nji.nl/Scheiding-Praktijk-Erkende-interventies
- https://www.nji.nl/Scheiding-Praktijk-Wat-werkt
- http://www.nji.nl/wegwijzer-kind-en-scheiding
- https://www.rijksoverheid.nl/onderwerpen/scheiden/vraag-en-antwoord/kind-bij-vechtscheiding
- https://www.rijksoverheid.nl/documenten/rapporten/2018/02/22/rapport-scheiden...en-de-kinderen-dan
- https://hetlock.nl/wp-content/uploads/2017/03/Vechtscheidingen-Belevingen-en-ervaringen-van-ouders-en-kinderen-en-veranderingen-na-Kinderen-uit-de-knel.pdf
- https://www.kinderbescherming.nl/themas/g/gezag-en-omgang/innovaties-voor-behandeling-conflict-scheidingen
- https://www.dekinderombudsman.nl/ul/cms/fck-uploaded/KOM003.2014Kinderombudsmanadviesrapport-vechtscheidingen.pdf
- http://richtlijnenjeugdhulp.nl/scheiding/
- https://www.villapinedo.nl/
- https://vooreenveiligthuis.nl/ik-maak-me-zorgen-om-iemand/ik-ben-ouder-dan-18-jaar/ik-maak-me-zorgen-om-een-kind-in-een-vechtscheiding/
- http://kindbehartiger.nl
- http://www.tno.nl/atlas
