# Supplementary material for: Lesser-known types of violence: Helping nurses and midwives to signal and act
Source: Int J Nurs Stud Adv. 2022 Sep 17;4:100098. doi: 10.1016/j.ijnsa.2022.100098 (PMC11080451; doi:10.1016/j.ijnsa.2022.100098)
Supplement: Supplementary file 1 [file mmc1.zip › Factsheets Dutch/vrouwelijke-genitale-verminking.pdf]

VROUWELIJKE GENITALE VERMINKING

GEBRUIK BIJ ELKE VORM VAN HUISELIJK GEWELD EN KINDERMISHANDELING DE MELDCODE! ▶

WAT IS VROUWELIJKE GENITALE VERMINKING?

Vrouwelijke genitale verminking (VGV), ook wel bekend als meisjes- of vrouwenbesnijdenis, is een ingreep aan de uitwendige vrouwelijke geslachtsorganen zonder medische noodzaak. Er zijn verschillende manieren waarop de vrouwelijke geslachtsorganen worden besneden. Dit hangt af van het lokale gebruik in land van herkomst, de wens van de ouder(s) en de besnijd(st)er. VGV komt onder bepaalde groep migranten voor. De meeste vrouwen en meisjes zijn besneden in land van herkomst maar wonen nu in Nederland (zie feiten en cijfers). De World Health Organization (WHO) onderscheidt vier types VGV. Voor meer informatie over de verschillende types, de gevolgen en klachten hiervan, zie de brochure Focalpoint meisjesbesnijdenis. VGV is in Nederland (net als in vele andere landen) strafbaar en is een vorm van kindermishandeling.

SIGNALEN: HOE KAN IK ZIEN DAT IEMAND SLACHTOFFER IS?

Signalen die kunnen duiden op een recent uitgevoerde besnijdenis:

- Het meisje is ziek geweest in de vakantie.
- Er is sprake van schoolverzuim.
- Het meisje ziet er moe/uitgeput/vaal uit.
- Ze gaat lang naar het toilet.
- Ze klaagt over buikpijn.
- Ze kan zich niet goed concentreren.
- Ze is stil en teruggetrokken.
- Ze reageert gesloten of afstandelijk.
- Ze blijft weg van gezondheidsonderzoeken.
- Ze kan een periode niet gymmen.
- Ze heeft moeilijkheden met lopen.

RISICOFACTOREN: WIE IS EXTRA KWETSBAAR VOOR DIT GEWELD?

Signalen die kunnen wijzen op een besnijdenis die mogelijk plaats gaat vinden:

- Er circuleren geruchten over een komende besnijdenis van een meisje.
- Er is een buitenlandse vakantie gepland, naar het land van herkomst.
- Familieleden en/of gezinsleden die besneden zijn.
- Mensen uit risicolanden die kort in Nederland zijn en beperkte kennis hebben over de Nederlandse wetgeving over VGV.
- Gezin ervaart druk vanuit familie en/of omgeving om VGV uit te voeren.
- Het meisje laat voorzichtig zelf iets los.

AANDACHTSPUNTEN VOOR DIT TYPE GEWELD BIJ HET DOORLOPEN VAN DE 5 STAPPEN IN DE MELDCODE

Bij elke vorm van huiselijk geweld en kindermishandeling dien je als professional de meldcode te gebruiken. Algemene meldcode richtlijnen (zoals de 5 stappen) staan niet op deze factsheet beschreven – bezoek daarvoor de link. Wél staan hier aandachtspunten specifiek voor deze vorm van geweld:

Voor een gesprek met de betrokkene(n) over het vermoeden van VGV zijn de volgende aandachtspunten van essentieel belang:

- Toon begrip voor de persoon, de cultuur en de situatie in een veilige sfeer.
- Investering van tijd en moeite om een vertrouwensrelatie op te bouwen met betrokkene(n). Overweeg de mogelijkheid om een getraind sleutelpersoon in te zetten. Raadpleeg FSAN voor inzet

FEITEN EN CIJFERS

Prevalentie VGV in Nederland: Er wonen in Nederland naar schatting ruim 29.000 vrouwen die een besnijdenis hebben ondergaan. Ongeveer 80% van deze vrouwen is afkomstig uit Somalië, Egypte, Ethiopië/Eritrea en de Koerdische autonome regio in Noord Irak. Voor meer informatie over waar VGV nog meer voorkomt zie prevalentie VGV wereldkaart.

Risico in Nederland: Naar schatting lopen jaarlijks tussen de 40 à 50 in Nederland woonachtige meisjes het risico besneden te worden, meestal tijdens vakantie en of familiebezoek naar land van herkomst. Deze cijfers komen uit 2013. In 2019 publiceert Pharos geactualiseerde cijfers.

ADVIES / MELDEN

Voor advies, melden en/of doorverwijzing naar opvang en/of andere hulp, bel:

- Veilig Thuis **0800 20 00**
- Pharos Focal Point VGV (alleen advies)

Bij acuut gevaar bel **112**

ENGELSE VERTALING

Zie hier.

Deze factsheet is ontwikkeld door Pharos i.s.m. diverse ketenpartners in de strijd tegen vrouwelijke genitale verminking.

november 2018

getrainde sleutelpersonen VGV.

- Zorg voor duidelijkheid en uitleg over de consequenties en ieders rol daarin.
- Omdat VGV een vorm van kindermishandeling is, is de Meldcode van toepassing. Bij vermoedens van een recent uitgevoerde besnijdenis of een dreigende besnijdenis moet men een melding doen bij Veilig Thuis. Bij Veilig Thuis zijn er aandachtsfunctionarissen VGV, meestal de vertrouwensartsen.
- Wees u bewust van uw eigen opvattingen en gevoelens tijdens een gesprek en breng deze, wanneer passend, naar voren.
- Bij gezondheidsklachten na VGV kunt u een meisje of vrouw verwijzen naar een van de spreekuren waar gespecialiseerde professionals werken.

MEER INFORMATIE

Zie de bronnen.
Zie ook de factsheet Eergerelateerd geweld.

De volgende 2 documenten zijn in het bijzonder nuttig:

- Bijlage 1 in de meldcode eergerelateerd geweld
- Het handelingsprotocol VGV bij minderjarigen

Deze factsheet is ontwikkeld door het Landelijk Expertisecentrum Gezondheidsverschillen Pharos i.s.m. diverse ketenpartners.

november 2018
