# Supplementary material for: Lesser-known types of violence: Helping nurses and midwives to signal and act
Source: Int J Nurs Stud Adv. 2022 Sep 17;4:100098. doi: 10.1016/j.ijnsa.2022.100098 (PMC11080451; doi:10.1016/j.ijnsa.2022.100098)
Supplement: Supplementary file 1 [file mmc1.zip › Factsheets Dutch/vrouwelijke-genitale-verminking-bronnen.pdf]

BRONNEN VROUWELIJKE GENITALE VERMINKING

Dit bestand geeft een overzicht van organisaties die betrokken zijn geweest bij de ontwikkeling van de bijbehorende factsheet en van beschikbare achtergrondinformatie (bronnen).

BETROKKEN ORGANISATIES

In het maken van deze factsheet over vrouwelijke genitale verminking voor professionals in alle beroepen die een meldcode huiselijk geweld en kindermishandeling hanteren, hebben de volgende organisaties input geleverd:

- Landelijk Expertisecentrum Gezondheidsverschillen Pharos. Voor vragen en/of opmerkingen over de factsheet, kunt u emailen met de hoofdauteurs: Yodit Jacob, Y.Jacob@pharos.nl, en Diana Geraci, D.Geraci@pharos.nl
- Knooppunt huwelijksdwang en achterlating, Diny Flierman
- Radboud umc, Karin van Rosmalen-Nooijens
- Fier - expertise en behandelcentrum op het terrein van geweld in afhankelijkheidsrelaties, Anke van Dijke
- Bureau Tangram, Suzanne Tan
- GGD GHOR, Annette Duenk en Sandra Hamming
- LEC EGG, Jeanine Janssen
- Veilig Thuis, Juliette Heetman

BRONNEN

De volgende documenten en informatiebronnen geven meer informatie over de signalen van vrouwelijke genitale verminking, risicofactoren, en dingen om op te letten bij het doorlopen van de 5 stappen van de meldcode huiselijk geweld en kindermishandeling:

- Factsheet Vrouwelijke Genitale Verminking. PHAROS, Utrecht, 2016. http://www.pharos.nl/documents/doc/factsheet_vgv.pdf
- Factsheet Vrouwelijke Genitale Verminking en de Nederlandse ketenaanpak. PHAROS, Utrecht, 2016.
- http://www.pharos.nl/documents/doc/factsheet-vgv-nederlandse_ketenaanpak.pdf
- Focal point meisjesbesnijdenis. PHAROS, Utrecht 2017 http://www.pharos.nl/documents/doc/webshop/vgvfolder-2017.pdf
- Handelingsprotocol Vrouwelijke Genitale Verminking bij minderjarigen: Uitleg en handvatten bij aanpak VGV voor Veilig Thuis, Raad voor de Kinderbescherming en Politie. PHAROS, Utrecht, 2016. http://www.pharos.nl/documents/doc/pharos-handelingsprotocol_vrouwelijke_genitale_verminking_bij_minderjarigen.pdf
- Factsheet: De Meldcode bij (vermoedens van) eergerelateerd geweld. Hilde Bakker en Oka Storms. MOVISIE, Utrecht, 2014. Bijlage 1 : Handelen bij (vermoedens van) meisjesbesnijdenis. P14 en volgende. https://www.huiselijkgeweld.nl/doc/publicaties/Meldcode_bij_vermoedens_van_eergerelateerd_geweld.pdf
- Richtlijn: Kindermishandeling (2016), thema 13 Vrouwelijke Genitale Verminking (VGV). NCJ. https://www.ncj.nl/richtlijnen/alle-richtlijnen/richtlijn/?richtlijn=12&rlpag=1643
- Richtlijn: Checklist Eer gerelateerd geweld https://www.politie.nl/themas/eergerelateerd-geweld-voor-professionals.html
- Vrouwelijke genitale verminking in Nederland, omvang, risico's en determinanten. http://www.pharos.nl/documents/doc/webshop/vrouwelijkegenitaleverminkinginnederland.pdf
- NVOG, AJN, KAMG, KNMG, KNOV, LHV, NHG, NVK, NVPC, NVU, NVVS, VVAK, Pharos (2010). Modelprotocol medische zorg voor vrouwen en meisjes met vrouwelijke genitale verminking (VGV), 2010. Pharos. Versie Juni 2010. Dit protocol is in herziening. Een update wordt in 2018 verwacht.
- Pijpers, F.I.M., M. Exterkate en M. de Jager (2010) Standpunt Preventie van Vrouwelijke Genitale Verminking (VGV) door de Jeugdgezondheidszorg. Centrum Jeugdgezondheid (RIVM).

OVERIG

- Raadpleeg FSAN voor inzet getrainde sleutelpersonen VGV
- Bij gezondheidsklachten na VGV kunt u een meisje of vrouw verwijzen naar een van de spreekuren waar gespecialiseerde professionals werken. Kijk hier voor een overzicht: https://www.pharos.nl/nl/kenniscentrum/meisjesbesnijdenis/focal-point-meisjesbesnijdenis/spreekuren
