# Supplementary material for: Lesser-known types of violence: Helping nurses and midwives to signal and act
Source: Int J Nurs Stud Adv. 2022 Sep 17;4:100098. doi: 10.1016/j.ijnsa.2022.100098 (PMC11080451; doi:10.1016/j.ijnsa.2022.100098)
Supplement: Supplementary file 1 [file mmc1.zip › Factsheets Dutch/zelfbeschadiging.pdf]

ZELFBESCHADIGING

WAT IS ZELFBESCHADIGING?

Zelfbeschadiging is het zichzelf lichamelijke verwondingen of schade toebrengen in een zich herhalend patroon, zonder bewuste suïcidale intentie. Het is vaak een uiting van een intense, overweldigende golf aan emoties of leegte die voor degene die zich beschadigt nauwelijks te verdragen is. Meestal heeft het weinig te maken met suïcidaliteit, zelfbeschadiging is eerder een overlevingsstrategie bij gebrek aan betere alternatieven.

SIGNALEN: HOE KAN IK ZIEN DAT IEMAND ZICHZELF BESCHADIGT?

Het is vaak niet zichtbaar dat iemand zichzelf beschadigt. Specifieke signalen zijn littekens van (bijv.) snij-en brandwonden, het dragen van bedekkende kleding (bijv. lange mouwen in de zomer) en het niet meedoen bij activiteiten waarbij iemand zich om moet kleden, zoals sporten, zwemmen. Er kunnen ook algemene signalen zijn dat het niet goed gaat met iemand, zoals zich terug trekken, emotionele uitbarstingen en een verslechtering in concentratie en prestaties.

RISICOFACTOREN: WIE HEEFT MEER KANS OM ZICHZELF TE (GAAN) BESCHADIGEN?

Er is niet één reden waarom mensen zichzelf beschadigen. Voorbeelden van risicofactoren die mensen kwetsbaar *kunnen* maken voor zelfbeschadigend gedrag zijn: vroegkinderlijk trauma (kindermishandeling, welke vorm dan ook). Het ontbreken van steun in moeilijke levensfasen. Perfectionisme, impulsiviteit. Gepest worden. Onvermogen om gevoelens te uiten. Lichte verstandelijke beperking. Allerlei psychiatrische stoornissen zoals psychotische-, stemming- en persoonlijkheidsstoornissen, verslaving, eetstoornis, post traumatische stress stoornis, autisme.

WAT DOE JE BIJ (VERMOEDENS VAN) ZELFBESCHADIGING?

Iemand die zichzelf beschadigt, schaamt zich vaak en is angstig over openheid richting anderen. In het eerste contact met iemand die zichzelf beschadigt is het daarom essentieel dat er een open, empathische, niet-oordelende houding is, met begrip voor het onderliggende lijden. Het contact dient zoveel mogelijk gelijkwaardig te zijn gericht op het opbouwen van een samenwerking, zie verder het document *Bejegening* (zie Bronnen).
Daarnaast:

- Bespreek (een vermoeden van) zelfbeschadiging rechtstreeks met degene die zich beschadigt.
- Als iemand zichzelf net beschadigd heeft, ga dan samen na of er (medische) zorg nodig is voor de wond(en) en spreek af wie dat doet
- Meestal is er geen sprake van suïcidaliteit, maar vraag dit wel na. Ook dit kan door rechtstreeks te vragen: wil je dood? Als hier sprake van is, zie verder de Handreiking 113 zelfmoordpreventie
- Ga samen op zoek naar betekenis en vervolgstappen, soms kan dit lotgenotencontact (Stichting Zelfbeschadiging) en/of een paar coachende gesprekken zijn, maar soms ook een langdurig therapeutisch proces. Als verwijzing nodig is, begeleid dan dit proces en blijf op de achtergrond aanwezig tot iemand goed terecht gekomen is (warme overdracht).
- Heb oog voor de impact op familie en naasten, als die in beeld zijn. Ondersteun hen ook.
- Zelfbeschadiging is in eerste instantie geen zaak voor Veilig Thuis en de meldcode. Toch zijn er situaties waar dit wel zo kan zijn, zie de volgende paragraaf.

FEITEN EN CIJFERS

- Zelfbeschadiging komt voor onder 5,5 % van de volwassen bevolking en onder ruim 15% van de adolescenten.
- Vrouwen beschadigen zich 1,5 keer zo vaak dan mannen.
- Ruim 4 % van de jongeren in Nederland heeft zich ooit beschadigd.
- De beginleeftijd ligt gemiddeld tussen de 12 en 14 jaar, de piek wordt gezien bij adolescenten.
- De meest voorkomende methode is snijden, krassen en branden.
- Er zijn geen cijfers over het verloop van zelfbeschadigend gedrag,
- in de praktijk wordt gezien dat een meerderheid voor het 30ste levensjaar stopt met zichzelf beschadigen.

ADVIES EN HULP

Voor meer informatie en advies over zelfbeschadiging kun je terecht bij:

- Je huisarts voor basiszorg en verwijzing
- de Stichting Zelfbeschadiging voor lotgenotencontact en ervaringsdeskundigheid
- Fivoor (info@fivoor.nl , **088 02 82 220**, vragen naar Nienke Kool) voor professionele en specifieke vragen
- Veilig Thuis **0800 20 00** (zie kopje in factsheet wanneer contact met Veilig Thuis belangrijk is)

Bij acuut gevaar: bel **112**.

ENGELSE VERTALING

Zie hier.

Deze factsheet is ontwikkeld door Fivoor i.s.m. diverse ketenpartners. november 2018

ADVIES EN HULP: RELATIE MET VEILIG THUIS / HUISELIJK GEWELD EN KINDERMISHANDELING

In de volgende situaties is het belangrijk om contact op te nemen met Veilig Thuis.

- De oorzaak voor de zelfbeschadiging hangt samen met een onveilige thuissituatie/ huiselijk geweld en/of kindermishandeling. Het is heel goed mogelijk dat dit pas na een paar gesprekken duidelijk wordt, omdat er eerst iets van vertrouwen opgebouwd moet worden. Als duidelijk is dat er een onveilig thuis is, dan moet de meldcode worden gevolgd.
- Er kunnen twijfels bestaan of de letsels zelf zijn aangebracht of door een ander. Wanneer men hierover twijfels heeft, bespreek dit dan met degene die zich beschadigd heeft. Is er sprake van geweld door anderen, neem dan contact op met Veilig Thuis voor een advies of melding. De volgende signalen kunnen wijzen op geweld door anderen:
    - letsel dat niet bij het verhaal past,
    - letsels zijn toegebracht op plekken waar cliënt moeilijk bij kan.
- Als degene die zich beschadigt volwassen is, is het belangrijk om na te gaan of iemand zelf kinderen heeft. Overweeg in dat geval een consultatie bij Veilig Thuis.

MEER INFORMATIE

Zie de bronnen.

Deze factsheet is ontwikkeld door Fivoor i.s.m. diverse ketenpartners. november 2018
