# Supplementary material for: Lesser-known types of violence: Helping nurses and midwives to signal and act
Source: Int J Nurs Stud Adv. 2022 Sep 17;4:100098. doi: 10.1016/j.ijnsa.2022.100098 (PMC11080451; doi:10.1016/j.ijnsa.2022.100098)
Supplement: Supplementary file 1 [file mmc1.zip › Factsheets Dutch/zelfbeschadiging-bronnen.pdf]

BRONNEN ZELFBESCHADIGING

Dit bestand geeft een overzicht van organisaties die betrokken zijn geweest bij de ontwikkeling van de bijbehorende factsheet en van beschikbare achtergrondinformatie (bronnen).

BETROKKEN ORGANISATIES

In het maken van deze factsheet over zelfbeschadiging hebben de volgende organisaties input geleverd:

- Fivoor, forensische en intensieve psychiatrische zorg. Voor vragen en/of opmerkingen over de factsheet, kunt u emailen met de hoofdauteur: Nienke Kool, n.kool@fivoor.nl , verpleegkundige en onderzoeker op het gebied van zelfbeschadigend gedrag.
- Stichting Zelfbeschadiging, organisatie voor en door mensen die zichzelf beschadigen en hun omgeving, Dwayne Meijnckens, dwayne@zelfbeschadiging.nl / www.zelfbeschadiging.nl
- Veilig Thuis, Juliette Heetman, heetman@xs4all.nl
- Nadine Callens, nadine.callens1@telenet.be, "auteur van boek 'zelfverwonding bij jongeren, een gids voor leerkrachten, leerlingenbegeleiders, ouders en vrienden", uitgeverij Garant-Maklu.

BRONNEN

De volgende documenten en informatiebronnen geven meer informatie over zelfbeschadiging:

- Handreiking 113 zelfmoordpreventie voor de huisartsenpraktijk. https://www.113.nl/sites/default/files/113/preventie/Handreiking%20113%20zefmoordpreventie%20huisartsen.pdf
- Nienke Kool, 2011. Bejegening. – te verkrijgen op verzoek bij de hoofdauteur Nienke Kool.
- Landelijke Stichting Zelfbeschadiging. Alternatieven voor zelfbeschadiging. https://www.zelfbeschadiging.nl/alternatieven-voor-zelfbeschadiging https://zelfbeschadiging.nl/wp-content/uploads/2016/07/Alternatieven-voor-zelfbeschadiging-1.pdf (bekeken op 16 aug 2018).
- Landelijke Stichting Zelfbeschadiging. Tips voor lotgenoten. – te verkrijgen op verzoek bij de hoofdauteur Nienke Kool.
- Landelijke Stichting Zelfbeschadiging. Zelfbeschadiging: wat kun jij doen om te helpen? https://www.zelfbeschadiging.nl/wat-kun-jij-doen-om-te-helpen/
- https://zelfbeschadiging.nl/wp-content/uploads/2017/01/Zelfbeschadiging-wat-kun-jij-doen-om-te-helpen-2.pdf (bekeken op 16 aug 2018)
- www.zelfbeschadiging.nl
