# Supplementary material for: Lesser-known types of violence: Helping nurses and midwives to signal and act
Source: Int J Nurs Stud Adv. 2022 Sep 17;4:100098. doi: 10.1016/j.ijnsa.2022.100098 (PMC11080451; doi:10.1016/j.ijnsa.2022.100098)
Supplement: Supplementary file 1 [file mmc1.zip › Factsheets English/(Ex-)partner violence - sources.pdf]

SOURCES (EX-)PARTNER VIOLENCE

ORGANISATIONS INVOLVED

The following organisations were involved in making this fact sheet:

- Movisie. For questions and/or remarks about the fact sheet, please email the main author: Wilma Schakenraad, w.schakenraad@movisie.nl
- Danielle van den Heuvel – Sterk Huis
- Suzanne Tan – Bureau Tangram
- Willemijn Krebbekx – Atria
- Karin van Rosmalen-Nooijens – Radboud UMC
- Marga Nicolaij – Veilig Thuis
- Mireille Bartelomij – Veilig Thuis

SOURCES

The following documents and other sources provide more information about the topic of this fact sheet:

- Daru, S., J. Mejdoubi, K. de Vaan en A. Visser (2016), Huiselijk geweld verklaard vanuit genderperspectief: Literatuurstudie. Amsterdam/Utrecht: Atria, Movisie en Regioplan.
- Janssen, H., Wentzel, W., & Vissers, B. (2015). Basisboek huiselijk geweld: signaleren, melden en aanpakken. 3e herz. druk. Bussum: Couthinho.
- Janssens, K., Visser, A., & Oosten, N. van (2017). RelatieWijs. Beoordelen en aanpakken van (ex-) partnergeweld. Utrecht: Movisie.
- Römkens, R., de Jong, T., & Harthoorn, H. (2014). Geweld tegen vrouwen. Europese onderzoeksgegevens in Nederlandse context. Amsterdam: Atria.
- R. Römkens, A. van den Brink en T. de Jong, (2018 Welk geweld telt? Opvattingen van Nederlanders over partnergeweld. Amsterdam: Atria, kennisinstituut voor emancipatie en vrouwengeschiedenis en Blijf Groep 2018.
- Steketee, M., R. Römkens, T. Pels, K. Lünnemann, E. Smits van Waesberghe, J. Mak, J. Mejdoubi & H. Harthoorn (2016). Preventie van intergenerationeel geweld Nederland en EU. Verkenning van wat werkt. Kennisinstituut voor emancipatie en vrouwengeschiedenis: Atria en Utrecht: Verweij-Jonker Instituut.
- Veen, H.C.J. van der, & Bogaerts, S. (2010). Huiselijk geweld in Nederland: overkoepelend synthese-rapport van het vangst-hervangst-, slachtoffer- en daderonderzoek 2007-2010. Den Haag, WODC.
- Verwijs, R., & Lünnemann, K. (2012). Partnergeweld. Achtergrond en risicofactoren. Utrecht: Verwey-Jonker Instituut.
- Signalenkaarten van Kadera, zie signalenkaart.nl/, These contain signals that can indicate domestic violence, both among victims and perpetrators. The signalling map is divided by age with risk factors in eight areas. One of the maps is aimed at adult offenders

This fact sheet was developed by Movisie in collaboration with various chain partners. september 2020
