# Supplementary material for: Lesser-known types of violence: Helping nurses and midwives to signal and act
Source: Int J Nurs Stud Adv. 2022 Sep 17;4:100098. doi: 10.1016/j.ijnsa.2022.100098 (PMC11080451; doi:10.1016/j.ijnsa.2022.100098)
Supplement: Supplementary file 1 [file mmc1.zip › Factsheets English/(Ex-)partner violence.pdf]

(EX-)PARTNER VIOLENCE

ALWAYS USE THE REPORTING CODE WHEN YOU ENCOUNTER A FORM OF (DOMESTIC) VIOLENCE, ABUSE, NEGLECT OR EXPLOITATION! ▶

This fact sheet is part of a series about *(domestic) violence, abuse, neglect, exploitation* and other types of harm that may be inflicted onto someone in a power-imbalanced relationship. Power-imbalanced relationships can exist with anyone, for example: an (ex-)partner, a child, a parent, a sibling, another family member, an informal or a professional carer, a friend, a flatmate or neighbour, a teacher, a colleague or supervisor, or just someone you know. These fact sheets describe different types of harm that can be inflicted in these relationships. They are meant as an add-on to the Dutch Reporting Code for these issues (English version here) and were developed for two reasons: 1) To provide professionals with an overview of all the types of harm that exist, to aid them in identifying both well-known and lesser-known types (see the Overview). 2) Signs/indicators may vary greatly by type of harm and certain types of harm require specific courses of action; the fact sheets help professionals with identifying the signs/indicators and risk factors of *each specific type* of harm and with acting appropriately when they do. Note: the general 5 steps in the Reporting Code are applicable to all types of harm in power-imbalanced relationships; the factsheets provide more guidance within these 5 steps – they are an add-on, not a replacement.

Below is a brief introduction to the topic of (ex-)partner violence, an overview of the signs/indicators and risk factors associated with this type of violence, and points of focus for when you encounter it.

WHAT IS (EX-)PARTNER VIOLENCE?

(Ex-)partner violence is any form of violence between partners or ex-partners. A (constant) threat of violence is also regarded as (ex-)partner violence. This includes all forms of violence; physical, emotional/psychological, sexual, financial or online, and stalking.

We can distinguish different categories of partner violence, including:

1. Common couple violence

This form of violence often arises from powerlessness, through loss of control. The perpetrator can be either partner. There can be a difference in the severity of the violence and the consequences differ per person. Consequences may be greater for women than for men (Daru et al., 2016).

2. Intimate terrorism

One-sided (threatening) serious violence, usually but not always committed by men. It is deliberate violence, partner terror, aimed at control over the partner.

POSSIBLE SIGNS/INDICATORS: HOW TO IDENTIFY IT

Signs/indicators that may indicate someone is a victim of (ex-) partner violence can be divided into:

- **physical signs**
  Repeated injuries: bruises, cuts, bites or head injuries, fractures (especially nose, bones, ribs), dislocations (especially jaw and shoulder), burns, loss of teeth, skull injuries and damage to the genitals.

- **psychosomatic signs**
  Depression, tremors, frequent headaches or stomach aches, fatigue, anxiety, sleep disorders, hyperventilation, heart palpitations.

FACTS AND FIGURES

According to a report by the FRA (European Union Agency for Fundamental Rights, 2014) that focused exclusively on female victims, 9% of Dutch women have experienced physical or sexual violence by their current partner and 25% by an ex-partner since age 15:

- One in five women has faced physical abuse by a partner or ex-partner.
- 11% of women have experienced sexual violence by a partner or ex-partner.
- In the past year, 3% of women have sometimes avoided their own homes for fear of violence.

(Ex-) partner violence makes up for more than 60% of domestic violence cases. Women (60%) are more likely to be victims of (ex-)partner violence than men (40%). The violence that men commit is more often of a structural nature than the violence that women commit. For both men and women, gender-specific assistance and care is possible and needed.

This fact sheet was developed by Movisie in collaboration with various chain partners. september 2020

(EX-)PARTNER VIOLENCE

- **behavioural signs**
  Difficulty making eye contact, always postponing appointments, a submissive attitude, vague requests for help, being anxious, women who are always accompanied by their partner, and/or cancelling last-minute consultations. on the other side of the spectrum: more than average use of care providers (GPs), more use of sleeping tablets and painkillers, or looking for an explanation for complaints more than average.

A complete overview of possible signs/indicators, including signs exhibited by children can be found here.

AT-RISK GROUPS

Partner violence can among all layers and groups within the population. Certain stress factors increase the risk of problems and violence occurring in the relationship, which is why in reality partner violence tends to be seen more often with couples who are dealing with stressors such as poverty, unemployment or other problems. This often involves a a combination of factors that result in (ex-)partner violence.

RISK FACTORS?

We can distinguish the following (groups of) risk factors:

1 Power discrepancy and dependence. Relational dependence, economic dependence, dependence because of residence status, care dependence, or a combination thereof.
2 Gender inequality.
3 Lack of defence mechanisms and social skills.
4 Isolation.
5 Problematic divorces.
6 Stress caused by a multitude of problems in the family, such as financial problems and debts, problems at work or problems due to lack of work, poor housing.
7 Alcohol and/or drug abuse.
8 Previous abuse (in earlier relationships or in the present relationship).
9 Behavioural problems at a young age.
10 Culturally determined tolerance towards violence.
11 Personal problems, including psychological or psychiatric problems and/or a mental disability.
12 Pregnancy (the correlation with partner violence is high).
13 Experienced violence in the past or witnessed partner violence in parents (girls are more likely to become victims and boys more likely to become perpetrators).

POINTS OF ATTENTION WHEN GOING THROUGH THE 5 STEPS IN THE REPORTING CODE

For any form of (domestic) violence, abuse, neglect or exploitation, professionals in the Netherlands are required to use the Reporting Code. For general reporting code guidelines (such as the 5 steps in this code) visit the link; these are not described in this fact sheet. We do describe here points of attention in going through the 5 steps that are specific to the topic of this fact sheet. These are:

If there is a suspicion of partner violence, always carry out the "Child Check". A separate fact sheet has been published on this subject.

"RELATIEWIJS" PROGRAMME

How do you know as a professional when behaviour in a relationship crosses a boundary? What do you do if there appears to be (ex-)partner violence? How do you make sure people can start talking about it? "RelatieWijs" is an aid in going through all steps of the Reporting Code. It helps in documenting signs/indicators of

MORE INFORMATION

See the Sources.

ADVICE/REPORTING

For advice, for reporting victims or perpetrators, and/or for referring someone to care (including shelters), call:

- Veilig Thuis ("Veilig Thuis" means "Safe at Home" in Dutch, it is the organization in the Netherlands for advice on, referrals to and reporting of any type of (domestic) violence, abuse, neglect or exploitation, or other types of harm in power-imbalanced relationships). Telephone: **0800 20 00**, free of charge and always open (24 hours per day, 7 days a week). It is possible to call anonymously and/or to call for advice or information only, without reporting someone.

In case of acute danger call the emergency services at the phone number **112**.

DUTCH TRANSLATION

See here.

This fact sheet was developed by Movisie in collaboration with various chain partners. september 2020

violence and assessing relational (boundary-crossing) behaviour based on objective criteria. These criteria can enable the partners to start talking about the identified behaviours: they make it possible to describe the behaviour in concrete terms and indicate when further research is needed. RelatieWijs uses seven criteria to determine whether relational behaviour is 'healthy' or boundary-crossing: mutual consent, voluntary action, equality, self-determination, appropriate to the context, self-respect and respect for the other.

This fact sheet was developed by Movisie in collaboration with various chain partners. september 2020
