# Supplementary material for: Lesser-known types of violence: Helping nurses and midwives to signal and act
Source: Int J Nurs Stud Adv. 2022 Sep 17;4:100098. doi: 10.1016/j.ijnsa.2022.100098 (PMC11080451; doi:10.1016/j.ijnsa.2022.100098)
Supplement: Supplementary file 1 [file mmc1.zip › Factsheets English/Abandoment - sources.pdf]

SOURCES ABANDONMENT

ORGANISATIONS INVOLVED

The following organisations were involved in making this fact sheet:

- The Landelijk Knooppunt Huwelijksdwang en Achterlating. For questions and/or remarks about the fact sheet, please email the main author: Diny Flierman, d.flierman@veiligthuishaaglanden.nl
- Augeo Foundation, Edith Geurts
- Bureau Tangram, Suzanne Tan
- CoMensha, Rik Viergever
- Fier - expertise en behandelcentrum op het terrein van geweld in afhankelijkheidsrelaties, Achille van Hees
- GGD GHOR Nederland, Annette Duenk en Sandra Hamming
- Landelijk Expertisecentrum Eergerelateerd Geweld, Korps nationale politie, Janine Janssen
- Leger des Heils Jeugdbescherming & Reclassering, Juul Polders
- Movisie, Oka Storms
- Sterk Huis, Diane de Winter
- Veilig Thuis, Sabina van der Meer
- Vereniging Vertrouwensartsen Kindermishandeling (VVAK)/ Veilig Thuis, Juliette Heetman
- Verwey-Jonker Instituut, Eliane Smits van Waesberghe

SOURCES

The following documents and other sources provide more information about the topic of this fact sheet:

Documents

- Bakker, H., Storms, O. (2015). De Meldcode bij (vermoedens van) eergerelateerd geweld. www.movisie.nl/publicatie/meldcode-vermoedens-eergerelateerd-geweld
- Bartels, E. A. C. (2005). Onderzoeksnotitie over migrantenvrouwen en kinderen die gedwongen zijn achtergelaten in landen van herkomst. (Voorstudie; No. 6). Den Haag: Advies Commissie Vreemdelingenzaken.
- Checklist EGG (eergerelateerd geweld).
- www.politie.nl/themas/eergerelateerd-geweld-voor-professionals.html
- Corringting, P. (2013). Going back 'home' Somali parents sending back their children from the Netherlands to Somalia. Master's thesis Social and Cultural Anthropology. Amsterdam: VU University.
- Herken de signalen en ga in gesprek. Tips voor professionals.
- www.huwelijksdwangenachterlating.nl/sites/www.huwelijksdwangenachterlating.nl/files/downloads/signaalkaart.pdf
- Janssen, J. (2017). Focus op eer. Een verkenning van eerzaken voor politieambtenaren en andere professionals. Den Haag: Boom criminologie. Onder meer voor relatie tussen eergerelateerd geweld en achterlating.
- Smits van Waesberghe, E., Sportel, I., Drost, E., Eijk, E. van, & Diepenbrock, E. (2014). Zo zijn we niet getrouwd. Een onderzoek naar omvang en aard van huwelijksdwang, achterlating en huwelijkse gevangenschap. Utrecht: Verweij-Jonker Instituut. www.verwey-jonker.nl/doc/vitaliteit/7414_Zo%20zijn%20we%20niet%20getrouwd_web.pdf

Websites

- Website of the Dutch Centre of forced marriage and abandonment
- www.politie.nl/themas/eergerelateerd-geweld.html
- www.nederlandwereldwijd.nl/hulp-bij-nood/huwelijksdwang

This fact sheet was developed by Landelijk Knooppunt Huwelijksdwang en Achterlating in collaboration with various chain partners. september 2020
