# Supplementary material for: Lesser-known types of violence: Helping nurses and midwives to signal and act
Source: Int J Nurs Stud Adv. 2022 Sep 17;4:100098. doi: 10.1016/j.ijnsa.2022.100098 (PMC11080451; doi:10.1016/j.ijnsa.2022.100098)
Supplement: Supplementary file 1 [file mmc1.zip › Factsheets English/Abandoment.pdf]

ABANDONMENT

ALWAYS USE THE REPORTING CODE WHEN YOU ENCOUNTER A FORM OF (DOMESTIC) VIOLENCE, ABUSE, NEGLECT OR EXPLOITATION! ▶

This fact sheet is part of a series about *(domestic) violence, abuse, neglect, exploitation* and other types of harm that may be inflicted onto someone in a power-imbalanced relationship. Power-imbalanced relationships can exist with anyone, for example: an (ex-)partner, a child, a parent, a sibling, another family member, an informal or a professional carer, a friend, a flatmate or neighbour, a teacher, a colleague or supervisor, or just someone you know. These fact sheets describe different types of harm that can be inflicted in these relationships. They are meant as an add-on to the Dutch Reporting Code for these issues (English version here) and were developed for two reasons: 1) To provide professionals with an overview of all the types of harm that exist, to aid them in identifying both well-known and lesser-known types (see the Overview). 2) Signs/indicators may vary greatly by type of harm and certain types of harm require specific courses of action; the fact sheets help professionals with identifying the signs/indicators and risk factors of *each specific type* of harm and with acting appropriately when they do. Note: the general 5 steps in the Reporting Code are applicable to all types of harm in power-imbalanced relationships; the factsheets provide more guidance within these 5 steps – they are an add-on, not a replacement.

Below is a brief introduction to this topic, an overview of the signs/indicators and risk factors associated with this type of harm, and points of attention for when you encounter it.

WHAT IS ABANDONMENT?

Abandonment entails that a child, young person, woman or man is send back or left behind against his or her will in another country, while the rest of the family returns to the Netherlands. There is an involuntary break with the social environment in the Netherlands. Abandonment occurs at the initiative of spouse, parent(s) or family. An attempt is made to prevent repatriation by taking away the victim's travel or residency documents. The victim is often in a dependent and/or isolated position abroad. The motives for abandoning a young person are often a fear of westerning or to prevent misconduct (e.g., bad friends, LGBTI+). Marital problems or issues of family honour are often the basis for the abandonment of women. Abandonment may be a way to get rid of a wife and make a new marriage possible.

POSSIBLE SIGNS/INDICATORS: HOW TO IDENTIFY IT

Prevention of abandonment requires that you are alert to warning signs and discuss them with the client. The signs are non-specific. Possible signs are:

- Not possessing one's own identity documents
- Suddenly going on holiday or earlier than planned
- Afraid of going on holiday, not returning from holiday / family visit

KEY FACTS

- The estimated number of victims is between 182 and 815 per year. This is more than the number of reports (178 per year).
- The majority of the victims are between 11 and 20 years old and adult women between 20 and 35 years old.
- Abandonment occurs in countries in North Africa (Morocco, Algeria, Egypt), West Africa (Guinea, Ghana, Nigeria), East Africa (Somalia, Kenya, Ethiopia, Sudan) and the Middle East (Iraq, Iran, Afghanistan, Turkey, Pakistan, India, Saudi Arabia, Syria).
- Educational level: both young people / women with a middle or high level of education, as well as those with little or no formal schooling, can become victims.

ADVICE/REPORTING

For advice, for reporting victims or perpetrators, and/or for referring someone to care (including shelters), call:

- Veilig Thuis ("Veilig Thuis" means "Safe at Home" in Dutch, it is the organization in the Netherlands for advice on, referrals to and reporting of any type of (domestic) violence, abuse, neglect or exploitation, or other types of harm in power-imbalanced relationships). Telephone: **0800 20 00**, free of charge and always open (24 hours per day, 7 days a week).

This fact sheet was developed by Landelijk Knooppunt Huwelijksdwang en Achterlating in collaboration with various chain partners. september 2020

ABANDONMENT

- Unexplained absence
- Behaviour change: withdrawn, shy, afraid, angry
- Puberty: growing up between cultures with conflicting values / norms
- Bad friends in the eyes of parents
- Conflicts between parents and child
- Running away from home
- Care provision does not sufficiently meet the demands of parents
- Ongoing investigations into the necessity of youth care

RISK FACTORS: WHO IS EXTRA VULNERABLE?

There is an increased risk of abandonment if a girl/woman or boy/man falls into one or more risk groups:

- Children, young people or married women with and without children from a closed community where traditional ideas prevail on the role, position, education and sexuality of women and men
- Young women with a partner-dependent residence permit (and their young children)
- Young people with mild mental disabilities or who are in special education

POINTS OF ATTENTION WHEN GOING THROUGH THE 5 STEPS IN THE REPORTING CODE

For any form of (domestic) violence, abuse, neglect or exploitation, professionals in the Netherlands are required to use the Reporting Code. For general reporting code guidelines (such as the 5 steps in this code) visit the link; these are not described in this fact sheet. We do describe here points of attention that are specific to the topic of this fact sheet. These are:

- Ask for advice and always consult an expert. Preventing and responding to abandonment requires specific expertise.
- Contact the police immediately if there is an acute threat to safety.
- Talk to the partner, parents/family only AFTER the situation and risks of such a conversation have been assessed with the help of an expert.
- When the victim is already abroad: do not talk to parents, partner or family for safety reasons

It is possible to call anonymously and/or to call for advice or information only, without reporting someone.

- The Landelijk Knooppunt Huwelijksdwang en Achterlating **070 34 54 319**

If you, or the person you know, is already abroad:

- If the victim is abroad, contact the Landelijk Knooppunt Huwelijksdwang en Achterlating directly.
- If you need help abroad, please contact the Dutch embassy.

Young people:

- Young people can chat anonymously; Eva and Zahir are national expertise and treatment centres.

Acute danger:

- In case of acute danger call the emergency services at the phone number **112**.

MORE INFORMATION

See the Sources.

DUTCH TRANSLATION

See here.

This fact sheet was developed by Landelijk Knooppunt Huwelijksdwang en Achterlating in collaboration with various chain partners.
