# Supplementary material for: Lesser-known types of violence: Helping nurses and midwives to signal and act
Source: Int J Nurs Stud Adv. 2022 Sep 17;4:100098. doi: 10.1016/j.ijnsa.2022.100098 (PMC11080451; doi:10.1016/j.ijnsa.2022.100098)
Supplement: Supplementary file 1 [file mmc1.zip › Factsheets English/Against migrants - sources.pdf]

SOURCES DOMESTIC VIOLENCE AGAINST/AMONG MIGRANTS IN VULNERABLE SITUATIONS

ORGANISATIONS INVOLVED

The following organisations were involved in making this fact sheet:

- Federatie Opvang (now Valente) and Stichting LOS. For questions and/or remarks about the fact sheet, please email the main author: Rian Ederveen, rian.ederveen@stichtinglos.nl

SOURCES

- The following documents and other sources provide more information about the topic of this fact sheet:
- Significant: Toegang tot de opvang van slachtoffers zonder eerdere verblijfsstatus, 3.11.17
- Ombudsman : Vrouwen in de knel (rapport 2017/075), 7.7.17
- KIS: Huiselijk geweld en veiligheid in asielopvangcentra
- Keygnaert, I. et al: Hidden Violence is a Silent Rape: Prevention of Sexual & Gender-Based Violence against Refugees & Asylum Seekers in Europe: a Participatory Approach Report. ICRH,Ugent, Ghent, 2008
- IND : Evaluatie gendergerelateerd vreemdelingenbeleid in Nederland, 2008
- WODC: Schattingen illegaal in Nederland verblijvende vreemdelingen 2012-2013, 2015

This fact sheet was developed by Federatie Opvang and Stichting LOS in collaboration with various chain partners.

september 2020
