# Supplementary material for: Lesser-known types of violence: Helping nurses and midwives to signal and act
Source: Int J Nurs Stud Adv. 2022 Sep 17;4:100098. doi: 10.1016/j.ijnsa.2022.100098 (PMC11080451; doi:10.1016/j.ijnsa.2022.100098)
Supplement: Supplementary file 1 [file mmc1.zip › Factsheets English/Against migrants.pdf]

DOMESTIC VIOLENCE AGAINST/AMONG MIGRANTS IN VULNERABLE SITUATIONS

This fact sheet is part of a series about *(domestic) violence, abuse, neglect, exploitation* and other types of harm that may be inflicted onto someone in a power-imbalanced relationship. Power-imbalanced relationships can exist with anyone, for example: an (ex-)partner, a child, a parent, a sibling, another family member, an informal or a professional carer, a friend, a flatmate or neighbour, a teacher, a colleague or supervisor, or just someone you know. These fact sheets describe different types of harm that can be inflicted in these relationships. They are meant as an add-on to the Dutch Reporting Code for these issues (English version here) and were developed for two reasons: 1) To provide professionals with an overview of all the types of harm that exist, to aid them in identifying both well-known and lesser-known types (see the Overview). 2) Signs/indicators may vary greatly by type of harm and certain types of harm require specific courses of action; the fact sheets help professionals with identifying the signs/indicators and risk factors of *each specific type* of harm and with acting appropriately when they do. Note: the general 5 steps in the Reporting Code are applicable to all types of harm in power-imbalanced relationships; the factsheets provide more guidance within these 5 steps – they are an add-on, not a replacement.

Below is a brief introduction to this topic, an overview of the signs/indicators and risk factors associated with this type of harm, and points of attention for when you encounter it.

ALWAYS USE THE REPORTING CODE WHEN YOU ENCOUNTER A FORM OF (DOMESTIC) VIOLENCE, ABUSE, NEGLECT OR EXPLOITATION! ▶

WHY A SEPARATE FACT SHEET ON MIGRANTS IN VULNERABLE SITUATIONS?

'Migrants' is the collective term for people who, for various reasons, have moved from abroad to the Netherlands to settle here temporarily or permanently. This fact sheet specifically deals with migrants **in vulnerable situations.** These groups have an increased risk of becoming victims of domestic violence and/or for them ending a relationship may have consequences for their residency status. For this reason, a separate factsheet was developed about these groups.

This fact sheet is not about victims of human trafficking, there is a separate fact sheet about that.

The specific characteristics of a number of groups of migrants in vulnerable situations are listed below – the groups that this fact sheet deals with.

FACTS AND FIGURES

The Dutch WODC (Research and Documentation Centre) estimates that in 2015 approximately 35,000 people resided in the Netherlands without a residence permit, of whom one third were women and one half were people with an asylum application that was rejected.

According to Significant (see Sources), the number of victims of domestic violence without a residence permit could be between 70 and 800 per year. Every year, some 30-40 victims of domestic violence apply for a residence permit on the basis of being a victim of domestic violence.

MORE INFORMATION

See the Sources and:

- IND brochure on the right of residence for victims of violence in power-imbalanced relationships
- Information on women's and men's shelters: www.opvang.nl, www.opvangatlas.nl, www.mannenopvang.nl
- Information about the rights of people without a residence permit: www.basicrights.nl, www.stichtinglos.nl or www.iLegalevrouw.nl
- Information on shelters for asylum seekers

This fact sheet was developed by Federatie Opvang and Stichting LOS in collaboration with various chain partners.

DOMESTIC VIOLENCE AGAINST/AMONG MIGRANTS IN VULNERABLE SITUATIONS

Category	Characteristics
Asylum seekers	Are awaiting a decision on application for a asylum residence permit and stay in an asylum seekers' centre (AZC). COA staff of these centres are trained in detecting domestic violence and they can also call in help from Veilig Thuis and women's shelters.
Status holders	Have received a residence permit and been allocated a residence or are still staying in an AZC pending allocation of a residence. Family members can travel with status holders for the purpose of family reunification. These family members are granted a derived residence permit.
Marriage migrants / Partner migrants	Living with the partner and having a residence permit that is dependent on that partner. Only after 5 years can they obtain an independent licence.
Europeans	Can be here as a partner migrant or be here to work.
Undocumented migrants	Undocumented migrants are migrants who entered the Netherlands illegally, migrants who stayed longer than their visa granted, rejected asylum seekers, or migrants who stayed after their right of residence was terminated on other grounds. They are largely invisible, very vulnerable and highly dependent on the person who shelters and feeds them. If the government comes into contact with them, they run the risk of being arrested and deported. That is why they are afraid of contact with formal authorities and often do not know their rights. As victims of domestic violence, they do have the right to protection, medical care, help and shelter under international treaties.

ADVICE/REPORTING

For advice, for reporting victims or perpetrators, and/or for referring someone to care (including shelters), call:

- Veilig Thuis ("Veilig Thuis" means "Safe at Home" in Dutch, it is the organization in the Netherlands for advice on, referrals to and reporting of any type of (domestic) violence, abuse, neglect or exploitation, or other types of harm in power-imbalanced relationships). Telephone: **0800 20 00**, free of charge and always open (24 hours per day, 7 days a week). It is possible to call anonymously and/or to call for advice or information only, without reporting someone.

In case of acute danger call the emergency services at the phone number **112**.

For advice about undocumented migrants, their rights and advice about available assistance, call:

- Stichting LOS **010 74 70 156**

DUTCH TRANSLATION

See here.

This fact sheet was developed by Federatie Opvang and Stichting LOS in collaboration with various chain partners.

DOMESTIC VIOLENCE AGAINST/AMONG MIGRANTS IN VULNERABLE SITUATIONS

DOMESTIC VIOLENCE AGAINST/AMONG MIGRANTS IN VULNERABLE SITUATIONS

Domestic violence is violence committed by someone from the domestic or family circle. It is about violence in a power-imbalanced relationship. The resulting dependence often makes it difficult for the victim to stop or escape from the violence. Migrants in vulnerable situations are often more dependent, see "Risk factors".

According to international law (Istanbul Convention and European Directive for victims), assistance and shelter for victims of domestic violence must be accessible, regardless of the residence status.

Domestic violence and subsequent separation can affect the right of residence:

Category	Characteristics
Status holders	Refugees and any family members who accompany them on their way to the Netherlands will retain their asylum status even after divorce.
Marriage migrants / Partner migrants	In the event of domestic violence, the dependent partner is granted a temporary permit. This is only extended if return would mean a risk of human rights violations in the country of origin.
Asylum seekers	If the asylum statement is linked to the other partner, the partner may no longer be able to obtain an asylum permit in the event of divorce. If separation in the country of origin leads to a risk of human rights violations, this may be an independent reason for an asylum permit.
Europeans	People from EU countries can stay in the Netherlands if they have their own means of existence. Contact with IND is important for Europeans who claim benefits and/or shelter.
Undocumented migrants	Undocumented migrants may be granted a permit as victims of domestic violence if their return would represent a risk of human rights violations in the country of origin.

This fact sheet was developed by Federatie Opvang and Stichting LOS in collaboration with various chain partners.

DOMESTIC VIOLENCE AGAINST/AMONG MIGRANTS IN VULNERABLE SITUATIONS

POSSIBLE SIGNS/INDICATORS: HOW TO IDENTIFY IT

In principle, the indicators of domestic violence for migrants in vulnerable situations are the same as for people with a Dutch background. However, it is particularly important for professionals working with migrants to pay attention to these signs because they are **more often hidden.** This is true especially for migrants who live in isolation, particularly undocumented migrants, who are afraid of institutions and who are not easily open about problems for cultural reasons.

RISK FACTORS: WHO IS EXTRA VULNERABLE?

Migrants in vulnerable situations may face an increased risk of domestic violence arising from various risk factors:

- Uncertainty about the rights of their residence status can make people even more dependent on partner or family and can make people reluctant to be open to authorities and other official agencies. This applies especially to people with no residency rights.
- Economic and social dependence can be high if one is not financially independent, has a low level of education and/or does not speak the language.
- Cultural aspects can play a role, for example the male-female relationships and/or reluctance to go to institutions with problems, unfamiliarity with institutions and/or shame about the situation.
- Unfamiliarity with the Dutch system of care and welfare.
- Migrants often have a more limited social network and therefore lack the protective factor that this provides.

POINTS OF ATTENTION WHEN GOING THROUGH THE 5 STEPS IN THE REPORTING CODE

For any form of (domestic) violence, abuse, neglect or exploitation, professionals in the Netherlands are required to use the Reporting Code. For general reporting code guidelines (such as the 5 steps in this code) visit the link; these are not described in this fact sheet. We do describe here points of attention in going through the 5 steps that are specific to the topic of this fact sheet. These are:

- Try to speak to the victim alone.
- Call in an interpreter if the victim has insufficient command of the Dutch language.
- Migrants with an uncertain right of residence and certainly undocumented migrants are often afraid of authorities and especially the police.
- Discuss carefully what the possibilities are to leave the situation of violence and what the possible consequences could be for the right of residence. For more information about these consequences see the Table about "Effects on right of residence".
- Often, after reporting a domestic violence situation, shelter needs to be arranged. It is important to start making arrangements for this immediately.

This fact sheet was developed by Federatie Opvang and Stichting LOS in collaboration with various chain partners.
