# Supplementary material for: Lesser-known types of violence: Helping nurses and midwives to signal and act
Source: Int J Nurs Stud Adv. 2022 Sep 17;4:100098. doi: 10.1016/j.ijnsa.2022.100098 (PMC11080451; doi:10.1016/j.ijnsa.2022.100098)
Supplement: Supplementary file 1 [file mmc1.zip › Factsheets English/Against people with disabilities - sources.pdf]

SOURCES

(SEXUAL) VIOLENCE AGAINST PEOPLE WITH INTELLECTUAL DISABILITIES

ORGANISATIONS INVOLVED

The following organisations were involved in making this fact sheet:

- Movisie – for questions and/or remarks about the fact sheet, please email the main author: Nico van Oosten, N.vanOosten@movisie.nl
- Bertine Spooren – GGD Amsterdam
- Sandra Hamming – GGD GHOR Nederland
- Hilair Balsters – Vilans
- Jolanda den Hartog – SIEN
- Marijke Lammers – Lammers Advies en Training
- Rianne van Beurden – Prisma
- Susan Dijkman – Veilig Thuis Kennemerland
- Wilma Schakenraad – Movisie

SOURCES

The following documents and other sources provide more information about the topic of this fact sheet:

- Berlo, W. van, Haas. S. de., Oosten, N. van, Dijk, L. van, Brants, L., Tonnon, S., & Storms, O. (2011). Beperkt Weerbaar - Een onderzoek naar seksueel geweld bij mensen met een lichamelijke, zintuiglijke of verstandelijke beperking, Utrecht: Rutgers WPF/MOVISIE.
- Casteel, C. Martin, S.L., Smith, J.B., Gurka, K.K. en Kupper, L.L. (2008). National study of physical and sexual assault among women with disabilities. Injury Prevention; vol. 14: 87-90
- FRA (2015). Violence against children with disabilities: easy read version.
- Heijden, I. van der (2014). What works to prevent violence against women with disabilities.
- Hughes, K., Bellis, M. A., Jones, L., Wood, S., Bates, G., Eckley, L., et al. (2012). Prevalence and risk of violence against adults with disabilities: a systematic review and meta-analysis of observation-al studies. Lancet, 379(9826), 1621-1629.
- Hughes, R. B., Lund, E. M., Gabrielli, J., Powers, L. E., & Curry, M. A. (2011). Prevalence of interper-sonal violence against community-living adults with disabilities: a literature review. Rehabilitation Psy-chology, 56(4), 302-319.
- Jones, L., Bellis, M. A., Wood, S., Hughes, K., McCoy, E., Eckley, L., et al. (2012). Prevalence and risk of violence against children with disabilities: a systematic review and meta-analysis of observa-tional studies. The Lancet, 380(9845), 899-907.
- Khemka, I., Hickson, L., Reynolds, G. (2005). Evaluation of a Decision-Making Curriculum Designed to Empower Women With Mental Retardation to Resist Abuse. American Journal On Mental Retardation, Volume 110, Number 3: 193–204, May 2005.
- Krnjacki, L., Emerson, E., Llewellyn, G., Kavanagh, A.M. (2016). Prevalence and risk of violence against people with and without disabilities: findings from an Australian population-based study. Australian and New Zealand Journal of Public Health, 40:16-21.
- Marsland, D., Oakes, P., & White, C. (2007). Abuse in care? The identification of early indicators of the abuse of people with learning disabilities in residential settings. The Journal of Adult Protection, 9(4), 6-20.
- Platt, L., Powers, L., Leotti, S., Hughes, R.B., Robinson, Whelen, S., Osborn, S. Ashkenazy, E., Beers, L., Lund, E. Nicolaidis, Ch., Partnering With People With Disabilities to Address Violence
- Consortium (2017). The Role of Gender in Violence Experienced by Adults With Developmental Disa-bilities. Journal of Interpersonal Violence, Vol. 32(1) 101–129.
- Plummer, S.B., Findley, P.A. (2012). Women With Disabilities' Experience With Physical and Sexual Abuse: Review of the Literature and Implications for the Field. Trauma Violence Abuse, 13(1) 15-29.
- Robinson-Whelen, S., Hughes, R.B., Gabrielli, J., Lund, E.M., Abramson, W., Swank, P.R. (2014)
- Sobsey, D. (2005). Violence & disability. In: W. M. Nehring (Ed.), Health promotion for persons with intellectual/developmental disabilities: The state of scientific evidence. Washington, DC: American Association on Mental Retardation.
- Strand, M.L., Benzein, E., Saveman, B. (2004). Violence in the care of adult persons with intellectual disabilities. Journal of Clinical Nursing, 13, 506–514.
- Trevellion, K., Oram, S., Feder, G., Howard, L.M. (2012). Experiences of Domestic Violence and Men-tal Disorders: A Systematic Review and Meta-Analysis. PLOS ONE, December, Volume 7 Issue 12.

This fact sheet was developed by Movisie in collaboration with various chain partners. september 2020
