# Supplementary material for: Lesser-known types of violence: Helping nurses and midwives to signal and act
Source: Int J Nurs Stud Adv. 2022 Sep 17;4:100098. doi: 10.1016/j.ijnsa.2022.100098 (PMC11080451; doi:10.1016/j.ijnsa.2022.100098)
Supplement: Supplementary file 1 [file mmc1.zip › Factsheets English/Against people with disabilities.pdf]

(SEXUAL) VIOLENCE AGAINST PEOPLE WITH INTELLECTUAL DISABILITIES

This fact sheet is part of a series about *(domestic) violence, abuse, neglect, exploitation* and other types of harm that may be inflicted onto someone in a power-imbalanced relationship. Power-imbalanced relationships can exist with anyone, for example: an (ex-)partner, a child, a parent, a sibling, another family member, an informal or a professional carer, a friend, a flatmate or neighbour, a teacher, a colleague or supervisor, or just someone you know. These fact sheets describe different types of harm that can be inflicted in these relationships. They are meant as an add-on to the Dutch Reporting Code for these issues (English version here) and were developed for two reasons: 1) To provide professionals with an overview of all the types of harm that exist, to aid them in identifying both well-known and lesser-known types (see the Overview). 2) Signs/indicators may vary greatly by type of harm and certain types of harm require specific courses of action; the fact sheets help professionals with identifying the signs/indicators and risk factors of *each specific type* of harm and with acting appropriately when they do. Note: the general 5 steps in the Reporting Code are applicable to all types of harm in power-imbalanced relationships; the factsheets provide more guidance within these 5 steps – they are an add-on, not a replacement.

Below is a brief introduction to this topic, an overview of the signs/indicators and risk factors associated with this type of harm, and points of attention for when you encounter it.

ALWAYS USE THE REPORTING CODE WHEN YOU ENCOUNTER A FORM OF (DOMESTIC) VIOLENCE, ABUSE, NEGLECT OR EXPLOITATION! ▶

WHAT FORMS OF VIOLENCE ARE WE TALKING ABOUT?

Like people without intellectual disabilities, people with intellectual disabilities may be subject to any type of (domestic) violence, abuse, neglect and/or exploitation, such as financial exploitation, physical abuse, psychological and emotional abuse, sexual abuse, sexual misconduct, neglect, violation of human and civil rights, and discrimination. They may experience this anywhere. The leading factor for whether or not the violence is reported to the police or Veilig Thuis ("Veilig Thuis" means "Safe at Home" in Dutch, it is the organization in the Netherlands for advice on, referrals to and reporting of any type of (domestic) violence, abuse, neglect or exploitation, or other types of harm in power-imbalanced relationships) is the relationship between the perpetrator and the victim, not the location (it does not matter whether it is committed inside the home or elsewhere). Examples of types of harm that people with mild intellectual disabilities may be subject to are: child abuse, partner violence, abuse by informal carers, elder abuse, child-parent abuse and honour-based violence. Young girls with a (light) mental disability are especially vulnerable to human trafficking and youth sex work.

ADVICE/REPORTING

For advice on this type of harm, reporting victims or perpetrators, or referring someone to care (including shelters), call:

- Veilig Thuis. Telephone: **0800 20 00**, free of charge and always open (24 hours per day, 7 days a week). It is possible to call anonymously and/or to call for advice or information only, without reporting someone.

In case of acute danger call the emergency services at the phone number **112**.

Sexual (or other) misconduct **by professional care providers** can be reported to:

- the 'Landelijk Meldpunt Zorg' of the 'Inspectie voor Gezondheidszorg en Jeugdzorg (IGJ)' **088 120 50 20**

DUTCH TRANSLATION

See here.

MORE INFORMATION

See the Sources.

This fact sheet was developed by Movisie in collaboration with various chain partners. september 2020

(SEXUAL) VIOLENCE AGAINST PEOPLE WITH INTELLECTUAL DISABILITIES

WHO IS THE TARGET GROUP?

A disability may be congenital or not (e.g. non-Congenital Brain Injury). In addition to their intellectual disability, people in this group may also suffer from other disabilities. In that case we speak of multiple disabilities. Finally, the intellectual disability or the combination of disabilities may vary in severity. In this fact sheet we limit ourselves to people with a congenital mental disability. Because of their limitations, these people are less resilient. They are often (strongly) dependent on care and support, especially in terms of social care. Because of their intellectual disability, they are less able to properly assess social situations (EQ), their ability to understand is less (IQ), and they often have more trouble expressing themselves verbally. Setting boundaries can therefore be difficult for them. People with intellectual disabilities are also often overestimated. Partly because their limitations are less visible and they themselves hide their limitations, and partly because they have no insight into their limitations.

FACTS AND FIGURES: HOW COMMON IS (SEXUAL) VIOLENCE AGAINST PEOPLE WITH INTELLECTUAL DISABILITIES?

The increased vulnerability of people with mental disabilities to various forms of violence is confirmed in a large number of domestic and foreign studies (see, among others, Platt et al. , 2017; Van der Heijden, 2014; Hughes et al., 2011; Van Berlo et al., 2011). Violence by acquaintances (including between people with intellectual disabilities) is most common (e.g. FRA, 2015).

- The UN suspects that children with disabilities face four times more violence than children without disabilities (FRA, 2015).
- A separate problem is witnessing violence between parents or other family members.
- According to foreign research, the prevalence of abuse among people with intellectual disabilities is 42% for boys and 39% for girls, 44% for adults men and 46% for adult women (Platt et al., 2017). An earlier review shows that people with intellectual disabilities are the most vulnerable group. This review shows that 26-90% of women and 29-86% of men with intellectual disabilities have at some point in their lives experienced some form of abuse (Hughes et al., 2011).
- Van Berlo et al. (2011)'s research into sexual misconduct among people with a disability is the most recent in our country in this field. It shows that when people are more specifically asked about different forms of sexual misconduct, the reported prevalence of such misconduct increases. The life-time prevalence for adult women with a (mild) mental disability was 72% and 44% for men in this study. It also concludes that 23% of women have ever been raped compared to 7% of men. Below the age of 16, the percentages for girls are 28% and for boys 19%.
- For bullying, figures that compare bullying prevalence between pupils with and without disabilities (see 'Kennisplein handicap sector') state that handicapped pupils are bullied five times more often: 10.8% against 2.2%. Children with disabilities are bullied 2 to 3 times as often as other children. Half of the children with a disability are afraid of their peers. 82% of children and young people (7-19 years) with a learning disorder have experienced bullying.

This fact sheet was developed by Movisie in collaboration with various chain partners.

(SEXUAL) VIOLENCE AGAINST PEOPLE WITH INTELLECTUAL DISABILITIES

POSSIBLE SIGNS/INDICATORS: HOW TO IDENTIFY IT

Signs with the victim

The most important signs of physical abuse are injuries, bruises spread over the body, and a combination of old and new bone fractures. Burns also occur. In people with intellectual disabilities, even sudden behavioural changes, such as withdrawal behaviour and aggression, are signs. For sexual misconduct, it can be a sign when the victim themselves start to engage in sexual misconduct and/or the use of sexual words that are not age-appropriate. Nevertheless, identifying violence is difficult among people with intellectual disabilities. They avoid making eye contact a little more because their limitations in general and in particular because of their limitations in communication and assessment of experiences, according to the research by Van Berlo et al. (2011). People with intellectual disabilities generally have less self-confidence, so this cannot be seen as a specific indicator of violence.

Signs in the offender

Signs or indicators that someone is a perpetrator of violence are the same as for perpetrators of violence against people without disabilities.

RISK FACTORS: WHO IS EXTRA VULNERABLE?

There are risk factors for violence at victim level (severity of the disability and deviant behaviour), perpetrator level (e.g. stress, parents with a slight mental disability) and - if the person with a mental disability stays in a care institution or receives support from an institution - at the organisation level of the care institution. For example, a lack of competent staff increases the risk of (sexual) misconduct among service users and also of family violence. Also, in such institutions, the violence committed is less often identified.

POINTS OF ATTENTION WHEN GOING THROUGH THE 5 STEPS IN THE REPORTING CODE

For any form of (domestic) violence, abuse, neglect or exploitation, professionals in the Netherlands are required to use the Reporting Code. For general reporting code guidelines (such as the 5 steps in this code) visit the link; these are not described in this fact sheet. We do describe here points of attention in going through the 5 steps that are specific to the topic of this fact sheet. These are:

- Special attention should be paid to the 'Child Check' when you are dealing with parents with a (mild) mental disability and/or low literacy.

This fact sheet was developed by Movisie in collaboration with various chain partners. september 2020
