# Supplementary material for: Lesser-known types of violence: Helping nurses and midwives to signal and act
Source: Int J Nurs Stud Adv. 2022 Sep 17;4:100098. doi: 10.1016/j.ijnsa.2022.100098 (PMC11080451; doi:10.1016/j.ijnsa.2022.100098)
Supplement: Supplementary file 1 [file mmc1.zip › Factsheets English/Bullying - sources.pdf]

SOURCES BULLYING

ORGANISATIONS INVOLVED

The following organisations were involved in making this fact sheet:

- TNO. For questions and/or remarks about the fact sheet, please email the main author: Minne Fekkes, minne.fekkes@tno.nl.
- Augeo, Edith Geurts
- Radboud umc, Karin van Rosmalen-Nooijens
- GGD GHOR, Sandra Hamming
- Veilig Thuis / VVAK, Juliette Heetman

SOURCES

The following documents and other sources provide more information about the topic of this fact sheet:

- www.pestweb.nl
- the JGZ guideline on Bullying www.ncj.nl/richtlijnen/alle-richtlijnen/richtlijn/pesten
- for interventions: www.uu.nl/sites/default/files/eindrapport-wat-werkt-tegen-pesten.pdf
- Vermande, M., van der Meulen, M. & Reijntjes, A. (Red.) *Pesten op school,* Boom Uitgevers Amsterdam.
