# Supplementary material for: Lesser-known types of violence: Helping nurses and midwives to signal and act
Source: Int J Nurs Stud Adv. 2022 Sep 17;4:100098. doi: 10.1016/j.ijnsa.2022.100098 (PMC11080451; doi:10.1016/j.ijnsa.2022.100098)
Supplement: Supplementary file 1 [file mmc1.zip › Factsheets English/Bullying.pdf]

BULLYING

ALWAYS USE THE REPORTING CODE WHEN YOU ENCOUNTER A FORM OF (DOMESTIC) VIOLENCE, ABUSE, NEGLECT OR EXPLOITATION! ▶

This fact sheet is part of a series about *(domestic) violence, abuse, neglect, exploitation* and other types of harm that may be inflicted onto someone in a power-imbalanced relationship. Power-imbalanced relationships can exist with anyone, for example: an (ex-)partner, a child, a parent, a sibling, another family member, an informal or a professional carer, a friend, a flatmate or neighbour, a teacher, a colleague or supervisor, or just someone you know. These fact sheets describe different types of harm that can be inflicted in these relationships. They are meant as an add-on to the Dutch Reporting Code for these issues (English version here) and were developed for two reasons: 1) To provide professionals with an overview of all the types of harm that exist, to aid them in identifying both well-known and lesser-known types (see the Overview). 2) Signs/indicators may vary greatly by type of harm and certain types of harm require specific courses of action; the fact sheets help professionals with identifying the signs/indicators and risk factors of *each specific type* of harm and with acting appropriately when they do. Note: the general 5 steps in the Reporting Code are applicable to all types of harm in power-imbalanced relationships; the factsheets provide more guidance within these 5 steps – they are an add-on, not a replacement.

Below is a brief introduction to this topic, an overview of the signs/indicators and risk factors associated with this type of harm, and points of attention for when you encounter it.

WHAT IS BULLYING?

Bullying is systematic aggressive behaviour in which one or more persons in a dominant position try to harm another person. Bullying can take the form of physical, verbal or psychological behaviour. Systematic exclusion is also included in the definition of bullying. Bullying often takes place in a group, for example in the classroom, or digitally, but can also take place in other places such as the sports club.

Bullying can lead to all kinds of negative consequences, including psychosomatic complaints, anxiety and depression, low self-confidence, loneliness, poor school performance and absenteeism. Some of these consequences can continue into adulthood. Besides the victims of bullying, perpetrators of bullying can also have problems in their development. The unadjusted behaviour and lack of empathy increases the chance of problematic behaviour in later life.

FACTS AND FIGURES

Every year, about 10% of primary-school pupils are victims of bullying. In secondary education this is about 8%.

Girls are bullied about as often as boys.

Homosexual, lesbian and transgender young people are twice as likely to be bullied and are also **much more likely to experience violent behaviour**. Various school programs are effective in reducing bullying.

MORE INFORMATION

See the Sources and:

- www.pestweb.nl
- the JGZ guideline Bullying
- *Pesten op school*. Vermande, M., van der Meulen, M. & Reijntjes, A. (Red.) Boom Uitgevers Amsterdam.
- Possible interventions

DUTCH TRANSLATION

See here.

This fact sheet was developed by TNO in collaboration with various chain partners.

september 2020

BULLYING

POSSIBLE SIGNS/INDICATORS: HOW TO IDENTIFY IT

The most important signs that may indicate that someone is being bullied are listed here below. Professionals can ask about these complaints and about someone's social functioning in general in order to start a conversation about bullying:

- Psychosomatic complaints such as abdominal pain, headache, bedwetting, eczema
- Anxious or depressed behaviour
- Withdrawn, quiet, sub-assertive behaviour
- Not enjoying school, not wanting to go to school
- Not having friends

RISK FACTORS: WHO IS EXTRA VULNERABLE?

The most important factors that can make children and young people more vulnerable to becoming a **victim** of bullying are:

- Being anxious, depressed, withdrawn or shy
- Being awkward physically or stuttering
- Being overweight, having ADHD or having autism
- Having few or no good friends
- Being LHBTQ
- Being non-average in terms of intelligence (gifted children or children with a mild intellectual disability)

In terms of **perpetrators**: children of parents with an aggressive/authoritarian parenting style are more likely to become perpetrators of bullying.

5-STEP PLAN FOR DEALING WITH BULLYING

When it is suspected that a child is being bullied, the child should be explicitly asked whether he or she is being bullied. If the child requires individual attention, an individual intervention can be implemented. Bullying is often a problem of the group and usually needs to be solved there, also in case of cyber-bullying. It is therefore important to involve parents and the school so that they take responsibility in solving the bullying problem. The Richtlijn voor Jeugdgezondheidszorg (the Youth Health Care Directive) recommends the following 5-step plan:

1. Estimation of needs of the child
2. Consultation with parents
3. Consultation with school, sports club or other organisation
4. Refer for individual help
5. Follow-up

This fact sheet was developed by TNO in collaboration with various chain partners. september 2020
