# Supplementary material for: Lesser-known types of violence: Helping nurses and midwives to signal and act
Source: Int J Nurs Stud Adv. 2022 Sep 17;4:100098. doi: 10.1016/j.ijnsa.2022.100098 (PMC11080451; doi:10.1016/j.ijnsa.2022.100098)
Supplement: Supplementary file 1 [file mmc1.zip › Factsheets English/Child Abuse.pdf]

# CHILD ABUSE

ALWAYS USE THE REPORTING CODE WHEN YOU ENCOUNTER A FORM OF (DOMESTIC) VIOLENCE, ABUSE, NEGLECT OR EXPLOITATION! ▶

This fact sheet is part of a series about *(domestic) violence, abuse, neglect, exploitation* and other types of harm that may be inflicted onto someone in a power-imbalanced relationship. Power-imbalanced relationships can exist with anyone, for example: an (ex-)partner, a child, a parent, a sibling, another family member, an informal or a professional carer, a friend, a flatmate or neighbour, a teacher, a colleague or supervisor, or just someone you know. These fact sheets describe different types of harm that can be inflicted in these relationships. They are meant as an add-on to the Dutch Reporting Code for these issues (English version here) and were developed for two reasons: 1) To provide professionals with an overview of all the types of harm that exist, to aid them in identifying both well-known and lesser-known types (see the Overview). 2) Signs/indicators may vary greatly by type of harm and certain types of harm require specific courses of action; the fact sheets help professionals with identifying the signs/indicators and risk factors of *each specific type* of harm and with acting appropriately when they do. Note: the general 5 steps in the Reporting Code are applicable to all types of harm in power-imbalanced relationships; the factsheets provide more guidance within these 5 steps – they are an add-on, not a replacement.

Below is a brief introduction to the topic of child abuse, an overview of the signs/indicators and risk factors associated with this type of violence, and points of focus for when you encounter it.

## WHAT IS CHILD ABUSE?

The Youth Law in the Netherlands defines child abuse as: 'Any form of threatening or violent interaction of a physical, psychological or sexual nature that is actively or passively imposed on the minor by the parents or other persons towards whom the minor is in a relationship of dependence or unfreedom, causing or threatening to cause serious mental or physical harm to the minor'.

## SHAPES

Child abuse takes various forms, such as physical abuse, physical neglect, psychological abuse, psychological neglect, sexual abuse (also online), and witnessing (partner) violence. Specific forms of physical abuse include shaken baby syndrom, pediatric condition falsification and female genital mutilation.

## ASSESSING SAFETY AND RISKS

Information about risks and protective factors is summarised in the various Child Abuse Guidelines that exist in the Netherlands.

A few points of attention:

- Risk factors increase the risk of child abuse.
- The more risk factors, the greater the risk of the occurrence or recurrence of child abuse.
- The main risk factors pertain to parental characteristics and the living conditions of the family.
- Partner violence is an important risk factor.

Various instruments are available to help to make a risk assessment. In practice, there sometimes appear to be misunderstandings about risk assessment. Read more about this in the publication 'Veiligheid en risico's inschatten: wat helpt'.

## FACTS AND FIGURES

- According to the National Abuse Prevalence Study (NPM), it is estimated that in **2017** between **90.000 and 127.000 children and young people aged 0 to 18** were exposed to some form of child abuse **in the Netherlands**. This means **3 percent** of all children.
- The most common forms of child abuse are emotional and physical neglect, with 36 and 24 percent of cases respectively.
- More figures from the National Prevalence Study can be found here.
- In the **Children's Abuse Survey 2016**, almost **25 percent of** secondary school students (grades 1-4) say that they have ever **been victims** of child abuse in life. This corresponds with data from research into adverse childhood experiences (ACEs) among pupils in grades 7 and 8 of primary education.

## ADVICE / REPORT

For advice, reporting and/or referral to reception and/or other help, call: Safe Home **0800 20 00**
In case of acute danger call **112**

## DUTCH TRANSLATION

See here.



This fact sheet was developed by Nederlands Jeugdinstituut (NJi) in collaboration with various chain partners and experts. september 2020

# CHILD ABUSE

## DIFFERENCE BETWEEN ACUTE AND STRUCTURAL UNSAFETY

- Acute unsafety includes immediate physical danger or the absence of basic care for dependent children.
- Structural unsafety refers to families or households where there are recurring or continuous unsafe events and situations. This structural unsafety (sometimes due to a lack of 'child signs') is not detected by many professionals.

## POSSIBLE SIGNS/INDICATORS: HOW TO IDENTIFY IT

Sometimes children do not show any signs of abuse and seem to function well, despite many problems with the parents. Nevertheless, there may be a situation of unsafety in the home. There are many overviews of signs of child abuse. Almost all signs are non-specific: not one characteristic of appearance, behaviour or development is specific to child abuse and all characteristics can also be a sign of other problems. Therefore, perhaps more important than knowledge about signs is a 'sensitivity' to them and to how to weigh them.

Professionals working with adults should carry out the "Child Check". This is part of the reporting code and is therefore obligatory. Professionals are responsible for following the reporting code when signls and facts support a suspicion of child abuse.

## POINTS OF ATTENTION WHEN GOING THROUGH THE 5 STEPS IN THE REPORTING CODE

The Dutch Reporting Code has changed as of 1 January 2019: Reporting suspicions of child abuse to Veilig Thuis ("Veilig Thuis" means "Safe at Home" in Dutch, it is the organization in the Netherlands for advice on, referrals to and reporting of any type of (domestic) violence, abuse, neglect or exploitation, or other types of harm in power-imbalanced relationships) is mandatory as of 1 January 2019 in all cases where there is a suspicion of acute and/or structural unsafety. Read more about working with the changed reporting code here.

## WANT TO KNOW MORE?

- Definition and forms of child abuse (Child abuse file, NJi)
- Physical child abuse (Child abuse file, NJi)
- Child abuse guidelines (Child abuse file, NJi)
- Identifying child abuse (Child abuse file, NJi)
- Overview card of signs/indicators of domestic violence and child abuse
- Assessing safety and risks: what helps? The sense and nonsense of risk assessment-instruments
- Learning from emergencies 2 - Safety of children in vulnerable families (Inspectie Justitie en Veiligheid)
- The child check (Augeo)
- Risk factors, protective factors and signals - Recommendations (Directive: Child abuse, NCJ 2016)
- The assessment framework in the Report Code on Domestic Violence and Child Abuse (Dutch government, 2017)
- Veilig Thuis (Dutch Government)



This fact sheet was developed by Nederlands Jeugdinstituut (NJi) in collaboration with various chain partners and experts. september 2020
