# Supplementary material for: Lesser-known types of violence: Helping nurses and midwives to signal and act
Source: Int J Nurs Stud Adv. 2022 Sep 17;4:100098. doi: 10.1016/j.ijnsa.2022.100098 (PMC11080451; doi:10.1016/j.ijnsa.2022.100098)
Supplement: Supplementary file 1 [file mmc1.zip › Factsheets English/Children in divorces - sources.pdf]

SOURCES CHILDREN IN A CONFLICT SEPARATION

ADDITIONAL INFORMATION

Different definitions of a 'high-conflict separation' are in use. We use the following definition:
There is **a high-conflict separation** when communication between parents about parenting and visitation rights is conflictual, as a result of which the children cannot maintain relaxed contact with one or both parents for a long time, experience suffering, pressure or parentification, and/or the child's development is threatened. It happens regularly that parents deny that there is a high-conflict separation.

High-conflict separations may be accompanied by:

- **Psychological abuse**
  For example: Parents do things that are mentally and/or emotionally offensive to the other parent as witnessed by the children (swearing, threatening, manipulating, disqualifying, humiliating, belittling or bullying); parents take a negative view of the other parent in presence of the children; children may not maintain contact with the other parent (parental rejection)
- **Pedagogical neglect**
  For example: There is uncertainty and disagreement about the way in which parents wish to care for and raise their children. Parents do not agree on the assistance that is necessary to eliminate insecurity for the child or to support the development of the child.
- **Witness to domestic violence**
  For example: Children witness conflicts, which may be accompanied by physical violence, between parents. There are many different ways of exposure: ranging from direct exposure (direct witnessing through seeing or hearing violence) to indirect exposure (seeing and experiencing the consequences of violence, such as a blue eye or the tension in the home).

ORGANISATIONS INVOLVED

The following organisations were involved in making this fact sheet:

- Radboudumc, afdeling eerstelijnsgeneeskunde, gender in transmural care. For questions and/or remarks about the fact sheet, please email the main author: Karin van Rosmalen-Nooijens, Karin.vanRosmalen-Nooijens@radboudumc.nl
- HMC Westeinde, Hesther Diderich
- TNO, Fieke Pannebakker
- GGD GHOR Nederland, Sandra Hamming
- Augeo, Marga Haagmans
- Sterk Huis, Cindy de Rijke

Sources

The following documents and other sources provide more information about the topic of this fact sheet:

- www.nji.nl/Mogelijke-effecten-van-echtscheiding-op-het-kind
- www.nji.nl/Scheiding-Praktijk-Erkende-interventies
- www.nji.nl/Scheiding-Praktijk-Wat-werkt
- www.nji.nl/wegwijzer-kind-en-scheiding
- www.rijksoverheid.nl/onderwerpen/scheiden/vraag-en-antwoord/kind-bij-vechtscheiding
- www.rijksoverheid.nl/documenten/rapporten/2018/02/22/rapport-scheiden...en-de-kinderen-dan
- hetlock.nl/wp-content/uploads/2017/03/Vechtscheidingen-Belevingen-en-ervaringen-van-ouders-en-kinderen-en-veranderingen-na-Kinderen-uit-de-knel.pdf
- www.kinderbescherming.nl/themas/g/gezag-en-omgang/innovaties-voor-behandeling-conflictscheidingen
- www.dekinderombudsman.nl/ul/cms/fck-uploaded/KOM003.2014Kinderombudsmanadviesrapportvechtscheidingen.pdf
- richtlijnenjeugdhulp.nl/scheiding/
- www.villapinedo.nl/
- vooreenveiligthuis.nl/ik-maak-me-zorgen-om-iemand/ik-ben-ouder-dan-18-jaar/ik-maak-me-zorgen-om-een-kind-in-een-vechtscheiding/
- kindbehartiger.nl
- www.tno.nl/atlas

This fact sheet was developed by Radboudumc, afdeling eerstelijnsgeneeskunde, gender in transmural care in collaboration with various chain partners. september 2020
