# Supplementary material for: Lesser-known types of violence: Helping nurses and midwives to signal and act
Source: Int J Nurs Stud Adv. 2022 Sep 17;4:100098. doi: 10.1016/j.ijnsa.2022.100098 (PMC11080451; doi:10.1016/j.ijnsa.2022.100098)
Supplement: Supplementary file 1 [file mmc1.zip › Factsheets English/Children in divorces.pdf]

CHILDREN IN A HIGH-CONFLICT DIVORCE

ALWAYS USE THE REPORTING CODE WHEN YOU ENCOUNTER A FORM OF (DOMESTIC) VIOLENCE, ABUSE, NEGLECT OR EXPLOITATION! ▶

This fact sheet is part of a series about *(domestic) violence, abuse, neglect, exploitation* and other types of harm that may be inflicted onto someone in a power-imbalanced relationship. Power-imbalanced relationships can exist with anyone, for example: an (ex-)partner, a child, a parent, a sibling, another family member, an informal or a professional carer, a friend, a flatmate or neighbour, a teacher, a colleague or supervisor, or just someone you know. These fact sheets describe different types of harm that can be inflicted in these relationships. They are meant as an add-on to the Dutch Reporting Code for these issues (English version here) and were developed for two reasons: 1) To provide professionals with an overview of all the types of harm that exist, to aid them in identifying both well-known and lesser-known types (see the Overview). 2) Signs/indicators may vary greatly by type of harm and certain types of harm require specific courses of action; the fact sheets help professionals with identifying the signs/indicators and risk factors of *each specific type* of harm and with acting appropriately when they do. Note: the general 5 steps in the Reporting Code are applicable to all types of harm in power-imbalanced relationships; the factsheets provide more guidance within these 5 steps – they are an add-on, not a replacement.

Below is a brief introduction to this topic, an overview of the signs/indicators and risk factors associated with this type of harm, and points of attention for when you encounter it

WHAT IS A HIGH-CONFLICT DIVORCE?

We speak of a high-conflict divorce when children are hampered by violent and complex conflicts between parents, and have become the focus of the struggle between parents. In this regard, the type of contract or bond that existed between the parents does not matter (see the Sources for a more detailed definition).

Every separation puts the family system under pressure. Parents also suffer from a high-conflict divorce: they may experience feelings of shame and guilt, and feel like they are failing as parents. Parents no longer take up their parental position. In addition, parents in separations are more vulnerable to the development of psychological and addiction problems, to which their children are exposed.

Consequences of a high-conflict divorce for children can include:

- Psychological complaints, such as anxiety, depression and post-traumatic stress.
- Emotional neglect by one or both parents (resulting in loneliness).
- Loyalty conflicts: parents make opposite appeals to the child. Children have the feeling that they cannot/must not be loyal to both parents, which leads to internal conflicts.

FACTS AND FIGURES

- A high-conflict divorce is also called a complex separation or a "fight-separation" ("vechtscheiding") in Dutch.
- In 2017 there were 18,178 separations involving children under the age of 18 years in the Netherlands. A separation always affects a child, the consequences of which vary according to age.
- Every year about 3500 children are involved in a high-conflict divorce in the Netherlands.
- About half of these separations relate to unmarried partners, which makes it more difficult to identify this group (but not less relevant! For the involved children this makes no difference).
- In 2014, the Children's Ombudsman of the Netherlands estimated that at that time approximately 16,000 children in the Netherlands were suffering from the effects of a high-conflict divorce.
- About 50% of the children in a high-conflict divorce develop post-traumatic stress symptoms. About a third of the children in a high-conflict divorce continue to have complaints.

This fact sheet was developed by Radboudumc, afdeling eerstelijnsgeneeskunde, gender in transmural care in collaboration with various chain partners.

september 2020

CHILDREN IN A HIGH-CONFLICT DIVORCE

- School and development problems:
  - internalising behaviour problems, e.g. reclusive behaviour
  - externalising behaviour problems, e.g. difficulty with authority, problems in dealing with peers, deteriorating school performance and (sexual) risk behaviour
- Intergenerational transmission, which means that children themselves also end up in a problematic relationship.
- Parentification, where the child feels (or is made) responsible for the well-being of the parents.

POSSIBLE SIGNS/INDICATORS: HOW TO IDENTIFY IT

Children in a high-conflict divorce are always victims. The signs in children that may lead you to identify that the child is a victim of a high-conflict divorce are comparable to the signs for child abuse and domestic violence. Signs often only become visible if there are already consequences due to the separation, as described above. Therefore, when there is a separation and children are involved → always be alert.

A specific and latent sign of a high-conflict divorce is parental rejection or parental alienation, in which the child completely rejects one of the parents and, as it were, takes sides.

RISK FACTORS: WHO IS EXTRA VULNERABLE?

Every child with divorcing parents has a chance to end up in a high-conflict divorce and develop problems as a result. Nevertheless, there are some factors that may increase the risks of developing problems in children whose parents are in a high-conflict divorce:

- Pre-existing and/or other violence and child abuse.
- Addiction or psychological or psychiatric problems in (one of) the divorcing parents.
- Psychological violence in the family, in particular psychological warfare between the parents; serious and prolonged parental conflicts.
- If the child has a bad relationship with (one of) the parent(s) or step-parent(s).
- Changes such as relocation, school change and new partners.
- Financial problems (generally decline in SES and in particular when there is poverty)

POINTS OF ATTENTION WHEN GOING THROUGH THE 5 STEPS IN THE REPORTING CODE

For any form of (domestic) violence, abuse, neglect or exploitation, professionals in the Netherlands are required to use the Reporting Code. For general reporting code guidelines (such as the 5 steps in this code) visit the link; these are not described in this fact sheet. We do describe here points of attention in going through the 5 steps that are specific to the topic of this fact sheet. These are:

There are many different interests involved in high-conflict divorces. Parents often underestimate the impact on children.

- Talk to the parents. For example, you can tell the parents what you see in the child or what you see them do.
- Talk to the child. Children process the separation better if they have people around them who make them feel they are not alone and explain to them what is going on at their own level. This also includes the use of a Child Representative.

MORE INFORMATION

See the Sources. Also read the Fact sheet about the Child Check for related information. Additional information:

- www.nji.nl
- www.richtlijnenjeugdhulp.nl
- www.vooreenveiligthuis.nl

ADVICE/REPORTING

For advice, for reporting victims or perpetrators, and/or for referring someone to care (including shelters), call:

- Veilig Thuis ("Veilig Thuis" means "Safe at Home" in Dutch, it is the organization in the Netherlands for advice on, referrals to and reporting of any type of (domestic) violence, abuse, neglect or exploitation, or other types of harm in power-imbalanced relationships). Telephone: **0800 20 00**, free of charge and always open (24 hours per day, 7 days a week). It is possible to call anonymously and/or to call for advice or information only, without reporting someone.

In case of acute danger call the emergency services at the phone number **112**.

DUTCH TRANSLATION

See here.

This fact sheet was developed by Radboudumc, afdeling eerstelijnsgeneeskunde, gender in transmural care in collaboration with various chain partners.

september 2020

CHILDREN IN A HIGH-CONFLICT DIVORCE

- A high-conflict divorce is a form of child abuse. Therefore, consult with Veilig Thuis for advice (see Advice/reporting).
- Both parents with parental authority are entitled to information about their child up to 16 years of age, but this is not a right to information about the other parent. As a counsellor, it is extra important to remain neutral and to keep information clear but concise.
- Social workers can keep an eye on the interests of both parents and children. The best results are achieved by a cooperation between the legal and the social workers. Then, attention is paid to the welfare of both parents and children, and agreement is also reached on the legal conflict points with mediators, lawyers and judges.
- There are several recognised interventions that may help in separations. It is also good to take note of what works. For parents, easily accessible information can be useful, for example with the new Separation ATLAS. Children can also be supported preventively with group programmes, for example.

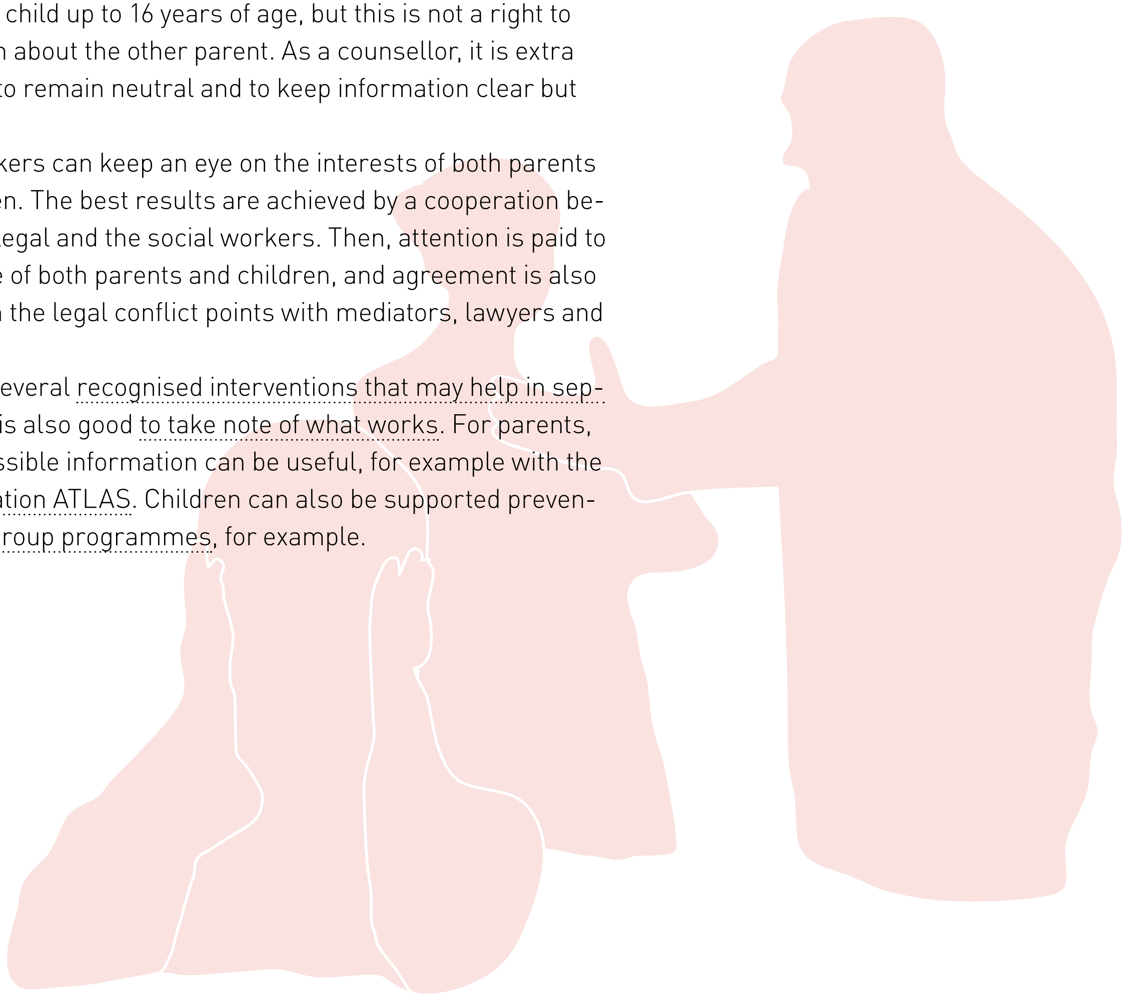

This fact sheet was developed by Radboudumc, afdeling eerstelijnsgeneeskunde, gender in transmural care in collaboration with various chain partners.
