# Supplementary material for: Lesser-known types of violence: Helping nurses and midwives to signal and act
Source: Int J Nurs Stud Adv. 2022 Sep 17;4:100098. doi: 10.1016/j.ijnsa.2022.100098 (PMC11080451; doi:10.1016/j.ijnsa.2022.100098)
Supplement: Supplementary file 1 [file mmc1.zip › Factsheets English/Elder abuse and abuse by carers - sources.pdf]

SOURCES ELDER ABUSE AND ABUSE BY INFORMAL CARERS

ORGANISATIONS INVOLVED

The following organisations were involved in making this fact sheet:

- Nederlandse Vereniging voor Klinische Geriatrie (NVKG) | Dutch Geriatrics Society NVKG (lead author of the factsheet on behalf of the NVKG: Ms Drs. M.E. van Houten, chairwoman of the Dutch guideline on suspected elder abuse in specialist healthcare services)
- Augeo
- GGD GHOR Nederland
- Movisie
- Veilig thuis
- Verenso

For questions and/or remarks about the fact sheet, please email info@nvkg.nl.

SOURCES

The following documents and other sources provide more information about the topic of this fact sheet:

- Actieplan 'Ouderen in veilige handen'. Brief aan de Tweede Kamer van 11 maart 2011, Kamerstukken II 2010/11, 29389, 30
- Comijs HC, Pot AM, Smit JH, et al. Elder abuse in the community: prevalence and consequences. J Am Geriatr Soc. 1998;46(7):885-8.PubMed PMID:9670877.
- Comijs, HC 1999, 'Elder mistreatment: prevalence, risk indicators and consequences', PhD, Vrije Universiteit Amsterdam.
- Gezondheidsmonitor Volwassenen en ouderen, 2016: bronnen.zorggegevens.nl/Bron?naam=Gezondheidsmonitor-Volwassenen-en-Ouderen%2C-GGD%E2%80%99en%2C-CBS-en-RIVM
- www.rijksoverheid.nl/binaries/rijksoverheid/documenten/rapporten/2015/06/15/ontspoorde-mantelzorg/ontspoorde-mantelzorg.pdf
- www.movisie.nl/sites/movisie.nl/files/publication-attachment/Factsheet%20Ouderenmishandeling%20III%20Financieel%20misbruik%20%5BMOV-287580-0.2%5D.pdf
- www.movisie.nl/sites/movisie.nl/files/publication-attachment/Factsheet%20ontspoorde%20mantelzorg%20%5BMOV-695455-1.1%5D.pdf
- www.movisie.nl/sites/movisie.nl/files/publication-attachment/Signalenkaart-Ontspoorde-Mantelzorg%20%5BMOV-458810-1.1%5D.pdf
- www.movisie.nl/sites/movisie.nl/files/publication-attachment/Factsheet-Ouderenmishandeling-Algemeen%20%5BMOV-225838-0.7%5D.pdf
- Dong X, Simon MA. Elder abuse as a risk factor for hospitalization in older persons. JAMA Intern Med. 2013;173(10):911-7. doi: 10.1001/jamainternmed.2013.238. PubMed PMID: 23567991.
- Dong XQ. Elder Abuse: Systematic Review and Implications for Practice. J Am Geriatr Soc. 2015;63(6):1214-38. doi: 10.1111/jgs.13454. Epub 2015 Jun 11. Review. PubMed PMID: 26096395.
- Handleiding ouderenmishandeling mei 2017, Samen Veilig Midden-Nederland, Utrecht
- Johannesen M, LoGiudice D. Elder abuse: a systematic review of risk factors in community-dwelling elders. Age Ageing. 2013;42(3):292-8. doi:10.1093/ageing/afs195. Epub 2013 Jan 22. Review. PubMed PMID: 23343837.
- McCausland B, Knight L, Page L, et al. A systematic review of the prevalence and odds of domestic abuse victimization among people with dementia. Int Rev Psychiatry. 2016;28(5):475-484. Epub 2016 Aug 26. PubMed PMID:27564566.
- Naughton C, J. Drennan, M.P. Treacy, A. Lafferty, I. Lyons, A. Phelan, S. Quin, O'Loughlin, A., Delaney, L. (2010) Abuse and Neglect of Older People in Ireland.
- Publicatie ouderen Veilig thuis, een beschrijving van de nieuwe werkwijze rondom ouderenmishandeling. Veilig thuis Utrecht. 2015,
- Prevalentieonderzoek naar aard en omvang van ouderenmishandeling, Regioplan, 2018
- Pillemer K, Burnes D, Riffin C, et al. Elder Abuse: Global Situation, Risk Factors, and Prevention Strategies. Gerontologist. 2016;56Suppl2:S194-205. doi: 10.1093/geront/gnw004. Review. PubMed PMID: 26994260; PubMed Central PMCID: PMC5291158.
- SCP-rapport Ouderenmishandeling in Nederland, 2015
- Yon Y, Mikton CR, Gassoumis ZD, Wilber KH. Elder abuse prevalence in community settings: a systematic review and meta-analysis. Lancet Glob Health. 2017 Feb;5(2):e147-e156. doi: 10.1016/S2214-109X(17)30006-2. Review. PubMed PMID: 28104184.

This fact sheet was developed by Nederlandse Vereniging voor Klinische Geriatrie (NVKG) in collaboration with various chain partners.
