# Supplementary material for: Lesser-known types of violence: Helping nurses and midwives to signal and act
Source: Int J Nurs Stud Adv. 2022 Sep 17;4:100098. doi: 10.1016/j.ijnsa.2022.100098 (PMC11080451; doi:10.1016/j.ijnsa.2022.100098)
Supplement: Supplementary file 1 [file mmc1.zip › Factsheets English/Elder abuse and abuse by carers.pdf]

ELDER ABUSE AND ABUSE BY INFORMAL CARERS

ALWAYS USE THE REPORTING CODE WHEN YOU ENCOUNTER A FORM OF (DOMESTIC) VIOLENCE, ABUSE, NEGLECT OR EXPLOITATION! ▶

This fact sheet is part of a series about *(domestic) violence, abuse, neglect, exploitation* and other types of harm that may be inflicted onto someone in a power-imbalanced relationship. Power-imbalanced relationships can exist with anyone, for example: an (ex-)partner, a child, a parent, a sibling, another family member, an informal or a professional carer, a friend, a flatmate or neighbour, a teacher, a colleague or supervisor, or just someone you know. These fact sheets describe different types of harm that can be inflicted in these relationships. They are meant as an add-on to the Dutch Reporting Code for these issues (English version here) and were developed for two reasons: 1) To provide professionals with an overview of all the types of harm that exist, to aid them in identifying both well-known and lesser-known types (see the Overview). 2) Signs/indicators may vary greatly by type of harm and certain types of harm require specific courses of action; the fact sheets help professionals with identifying the signs/indicators and risk factors of *each specific type* of harm and with acting appropriately when they do. Note: the general 5 steps in the Reporting Code are applicable to all types of harm in power-imbalanced relationships; the factsheets provide more guidance within these 5 steps – they are an add-on, not a replacement.

Below is a brief introduction to this topic, an overview of the signs/indicators and risk factors associated with this type of harm, and points of attention for when you encounter it.

WHAT IS ELDER ABUSE?

Elder abuse occurs when any person who has a recurring personal or professional relationship with the older person (aged 65 or over) acts or neglects to act in such a manner that it results in physical and/or psychological and/or material harm to the older person, while there is some form of partial or total dependency on the part of the older person (a power imbalance).

In the event of abuse by informal carers, the informal carer transgresses the boundaries of good care because of stress, powerlessness, incompetence or ignorance. A characteristic of abuse by informal carers is often the lack of intent.

FACTS AND FIGURES

- Gezondheidsmonitor 2016
  This monitor (spanning 12 months) shows that psychological abuse is the most common form of elder abuse and affects 4.0% of the elderly aged 65 and over in the Netherlands, followed by financial abuse (1.1%) and neglect (0.6%).
- Regioplan 2018
  This interview study (conducted in Rotterdam, Tilburg and Boxtel) concludes that 1 in 20 elderly people aged 65 and over living at home ever experience elder abuse at one point in their lives and 1 in 50 will experience elder abuse on an annual basis. In both cases, the most commonly reported form appears to be financial abuse, followed by psychological and physical abuse.

ADVICE/REPORTING

For advice, for reporting victims or perpetrators, and/or for referring someone to care (including shelters), call:

- Veilig Thuis ("Veilig Thuis" means "Safe at Home" in Dutch, it is the organization in the Netherlands for advice on, referrals to and reporting of any type of (domestic) violence, abuse, neglect or exploitation, or other types of harm in power-imbalanced relationships).

This fact sheet was developed by Nederlandse Vereniging voor Klinische Geriatrie (NVKG) in collaboration with various chain partners.

september 2020

ELDER ABUSE AND ABUSE BY INFORMAL CARERS

FORMS AND POSSIBLE SIGNS/INDICATORS OF ELDER ABUSE

Forms	Possible signs/indicators: how to identify it
Physical abuse (e.g. hitting, pushing, kicking, threatening or injuring)	unexplained subcutaneous bruising, fractures, injuries of different ages, injuries do not fit with reported circumstancesa
Psychological abuse (including repeated insults and belittling, restrictions on movement, denial of visits, withholding mail)	gedragsveranderingen, depressieve- en angstklachten
Financial abuse (e.g. changing the will, selling goods without permission, unauthorized use of someone's debit or credit card, abuse of personal care budgets)	lack of standard (medical) facilities, disappearance of belongings, sudden debts
Neglect (e.g. withholding nutrition, physical care or access to medical care, affective neglect)	untreated bedsores, malnutrition, poor physical hygiene, blemishes, inadequate supervision of older people
Sexual abuse (including verbal harassment, unwanted sexual acts with or in the presence of the older person)	unexplained subcutaneous bruising in genital area, bloodstains in underwear, unexplained sexually transmitted diseases

Telephone: **0800 20 00,** free of charge and always open (24 hours per day, 7 days a week). It is possible to call anonymously and/or to call for advice or information only, without reporting someone.

In case ofcute danger call the emergency services at the phone number 112.

MORE INFORMATION

See the Sources.

DUTCH TRANSLATION

See here.

This fact sheet was developed by Nederlandse Vereniging voor Klinische Geriatrie (NVKG) in collaboration with various chain partners.

ELDER ABUSE AND ABUSE BY INFORMAL CARERS

POSSIBLE SIGNS/INDICATORS OF ABUSE BY INFORMAL CARERS

- The carer being overburdened and frustrated, e.g. manifesting itself in boundary-transgressing behaviour towards the elderly or others
- Compassion fatigue on the part of the carer
- Refusing help or care for the elderly person, isolating the elderly person

RISK FACTORS: WHO IS EXTRA VULNERABLE?

On the part of victims:

- presence of cognitive disorders
- psychological / psychiatric disorders
- poor physical health
- functional limitations and dependency on care
- limited social network or loneliness
- lower socio-economic status

On the part of perpetrators:

- psychological / psychiatric disorders
- alcohol and substance use
- being overburdened in the informal care situation
- violence, abuse, neglect or exploitation in family history

POINTS OF ATTENTION WHEN GOING THROUGH THE 5 STEPS IN THE REPORTING CODE

For any form of (domestic) violence, abuse, neglect or exploitation, professionals in the Netherlands are required to use the Reporting Code. For general reporting code guidelines (such as the 5 steps in this code) visit the link; these are not described in this fact sheet. We do describe here points of attention in going through the 5 steps that are specific to the topic of this fact sheet. These are:

- Shame / taboo with victim and perpetrator may play a role

This fact sheet was developed by Nederlandse Vereniging voor Klinische Geriatrie (NVKG) in collaboration with various chain partners.

september 2020
