# Supplementary material for: Lesser-known types of violence: Helping nurses and midwives to signal and act
Source: Int J Nurs Stud Adv. 2022 Sep 17;4:100098. doi: 10.1016/j.ijnsa.2022.100098 (PMC11080451; doi:10.1016/j.ijnsa.2022.100098)
Supplement: Supplementary file 1 [file mmc1.zip › Factsheets English/Female genital mutilation - sources.pdf]

SOURCES FEMALE GENITAL MUTILATION

NOTES

- Consult FSAN for collaboration with trained FGM ambassadors.
- In case of health problems after FGM, you can refer a girl or woman to one of the consultation hours where specialised professionals work. Go to this website for an overview: www.pharos.nl/nl/kenniscentrum/meisjesbesnijdenis/focal-point-meisjesbesnijdenis/spreekuren

ORGANISATIONS INVOLVED

The following organisations were involved in making this fact sheet:

- Landelijk Expertisecentrum Gezondheidsverschillen Pharos. For questions and/or remarks about the fact sheet, please email the main authors: Yodit Jacob, Y.Jacob@pharos.nl and Diana Geraci, D.Geraci@pharos.nl
- Knooppunt huwelijksdwang en achterlating, Diny Flierman
- Radboud umc, Karin van Rosmalen-Nooijens
- Fier - expertise en behandelcentrum op het terrein van geweld in afhankelijkheidsrelaties, Anke van Dijke
- Bureau Tangram, Suzanne Tan
- GGD GHOR, Annette Duenk en Sandra Hamming
- LEC EGG, Jeanine Janssen
- Veilig Thuis, Juliette Heetman

SOURCES

The following documents and other sources provide more information about the topic of this fact sheet:

- Factsheet Vrouwelijke Genitale Verminking. PHAROS, Utrecht, 2016. www.pharos.nl/documents/doc/factsheet_vgv.pdf
- Factsheet Vrouwelijke Genitale Verminking en de Nederlandse ketenaanpak. PHAROS, Utrecht, 2016.
- www.pharos.nl/documents/doc/factsheet-vgv-nederlandse_ketenaanpak.pdf
- Focal point meisjesbesnijdenis. PHAROS, Utrecht 2017 www.pharos.nl/documents/doc/webshop/vgvfolder-2017.pdf
- Handelingsprotocol Vrouwelijke Genitale Verminking bij minderjarigen: Uitleg en handvatten bij aanpak VGV voor Veilig Thuis, Raad voor de Kinderbescherming en Politie. PHAROS, Utrecht, 2016. www.pharos.nl/documents/doc/pharos-handelingsprotocol_vrouwelijke_genitale_verminking_bij_minderjarigen.pdf
- Factsheet: De Meldcode bij (vermoedens van) eergerelateerd geweld. Hilde Bakker en Oka Storms. MOVISIE, Utrecht, 2014. Bijlage 1 : Handelen bij (vermoedens van) meisjesbesnijdenis. P14 en volgende. www.huiselijkgeweld.nl/doc/publicaties/Meldcode_bij_vermoedens_van_eergerelateerd_geweld.pdf
- Richtlijn: Kindermishandeling (2016), thema 13 Vrouwelijke Genitale Verminking (VGV). NCJ. www.ncj.nl/richtlijnen/alle-richtlijnen/richtlijn/?richtlijn=12&rlpag=1643
- Richtlijn: Checklist Eer gerelateerd geweld www.politie.nl/themas/eergerelateerd-geweld-voor-professionals.html
- Vrouwelijke genitale verminking in Nederland, omvang, risico's en determinanten. www.pharos.nl/documents/doc/webshop/vrouwelijkegenitaleverminkinginnederland.pdf
- Vrouwelijke Genitale Verminking Omvang en risico in Nederland. www.pharos.nl/kennisbank/vrouwelijke-genitale-verminking-omvang-en-risico-in-nederland/
- NVOG, AJN, KAMG, KNMG, KNOV, LHV, NHG, NVK, NVPC, NVU, NVVS, VVAK, Pharos (2010). Leidraad. Medische zorg voor vrouwen en meisjes met vrouwelijke genitale verminking (VGV), 2019. NVOG. www.pharos.nl/wp-content/uploads/2018/10/Leidraad-Medische-zorg-voor-vrouwen-en-meisjes-met-vrouwelijke-genitale-verminking.pdf
- Pijpers, F.I.M., M. Exterkate en M. de Jager (2010) Standpunt Preventie van Vrouwelijke Genitale Verminking (VGV) door de Jeugdgezondheidszorg. Centrum Jeugdgezondheid (RIVM).
- www.pharos.nl/wp-content/uploads/2020/07/The-prevalence-and-risk-of-Female-Genital-Mutilation-among-migrant-women-and-girls-in-the-Netherlands_journal.pone_.0230919.pdf

This fact sheet was developed by Pharos in collaboration with various chain partners in the fight against female genital mutilation.   september 2020
