# Supplementary material for: Lesser-known types of violence: Helping nurses and midwives to signal and act
Source: Int J Nurs Stud Adv. 2022 Sep 17;4:100098. doi: 10.1016/j.ijnsa.2022.100098 (PMC11080451; doi:10.1016/j.ijnsa.2022.100098)
Supplement: Supplementary file 1 [file mmc1.zip › Factsheets English/Female genital mutilation.pdf]

FEMALE GENITAL MUTILATION

ALWAYS USE THE REPORTING CODE WHEN YOU ENCOUNTER A FORM OF (DOMESTIC) VIOLENCE, ABUSE, NEGLECT OR EXPLOITATION! ▶

This fact sheet is part of a series about *(domestic) violence, abuse, neglect, exploitation* and other types of harm that may be inflicted onto someone in a power-imbalanced relationship. Power-imbalanced relationships can exist with anyone, for example: an (ex-)partner, a child, a parent, a sibling, another family member, an informal or a professional carer, a friend, a flatmate or neighbour, a teacher, a colleague or supervisor, or just someone you know. These fact sheets describe different types of harm that can be inflicted in these relationships. They are meant as an add-on to the Dutch Reporting Code for these issues (English version here) and were developed for two reasons: 1) To provide professionals with an overview of all the types of harm that exist, to aid them in identifying both well-known and lesser-known types (see the Overview). 2) Signs/indicators may vary greatly by type of harm and certain types of harm require specific courses of action; the fact sheets help professionals with identifying the signs/indicators and risk factors of *each specific type* of harm and with acting appropriately when they do. Note: the general 5 steps in the Reporting Code are applicable to all types of harm in power-imbalanced relationships; the factsheets provide more guidance within these 5 steps – they are an add-on, not a replacement.

Below is a brief introduction to the topic of female genital mutilation, an overview of the signs/indicators and risk factors associated with this type of harm, and points of attention for when you encounter it.

WHAT IS FEMALE GENITAL MUTILATION?

Female genital mutilation (FGM) is a practice involving the partial or total removal of the external female genital organs while there is no medical necessity. There are various ways in which FGM can be performed. The specific method used depends on the local traditions in the country of origin, the wishes of the parent(s), and the cutter, the person performing the practice. In the Netherlands, FGM is prevalent among certain groups of migrants. Most women and girls have been circumcised in their country of origin but now live in the Netherlands (see Facts and figures). The World Health Organization (WHO) distinguishes four types of FGM. For more information about the different types, their consequences and complaints, see the brochure "Focalpoint meisjesbesnijdenis". FGM is punishable in the Netherlands (as in many other countries) and is a form of child abuse.

POSSIBLE SIGNS/INDICATORS: HOW TO IDENTIFY IT

Signs that may indicate *a recently performed* FGM are:

- The girl has been sick during the holidays.
- The girl has been absent from school.
- The girl looks tired/exhausted.
- She goes to the toilet for long periods of time.
- She complains about abdominal pain.
- She cannot concentrate well.

FACTS AND FIGURES

Prevalence of FGM in the Netherlands

On 1 January 2018, in the Netherlands, there are an estimated 41,000 women who have undergone FGM. This represents 43% of all 95,000 migrants living in the Netherlands who are from countries where FGM is a cultural custom. Most of these women (82%) come from Somalia, Egypt, Ethiopia, Eritrea, Sudan or Iraq. For more information about other countries where FGM is prevalent, see the FGM prevalence world map by Pharos.

Risk of FGM in the Netherlands

In the Netherlands there are 38,000 girls aged 0-19 with at least one parent from a country where FGM is prevalent. Of these girls, 4200 run the risk of being circumcised in the next 20 years. This risk is especially high if their parents choose to continue the traditions of their country of origin and if preventive measures do not reach them. The risk is influenced by the length of the period of residence in the Netherlands, social pressure, knowledge about legislation, knowledge of the (health) risks of FGM, and people's attitude towards FGM.

This fact sheet was developed by Pharos in collaboration with various chain partners in the fight against female genital mutilation.

FEMALE GENITAL MUTILATION

- She is quiet and withdrawn.
- She reacts closed or is distant.
- She avoids health checks.
- She cannot do any physical exercise for a period of time.
- She has difficulty walking.

RISK FACTORS: WHO IS EXTRA VULNERABLE?

Signs that may indicate that FGM *could happen*:

- There are rumours circulating about a girl undergoing FGM.
- A holiday abroad is planned, to the country of origin.
- There are family members or relatives who have undergone FGM.
- People from high-risk countries who have only been in the Netherlands for a brief period of time and have limited knowledge about Dutch legislation on FGM.
- The family experiences pressure from relatives and/or the community to carry out FGM.
- The girl carefully speaks about the a planned FGM.

POINTS OF ATTENTION WHEN GOING THROUGH THE 5 STEPS IN THE REPORTING CODE

For any form of (domestic) violence, abuse, neglect or exploitation, professionals in the Netherlands are required to use the Reporting Code. For general reporting code guidelines (such as the 5 steps in this code) visit the link; these are not described in this fact sheet. Here, we do describe points of attention in going through the 5 steps that are specific to the topic of this fact sheet.

For a discussion with the person(s) suspected of involvement in a potential FGM-case, the following points of attention are essential:

- Show understanding for the person, the culture and the situation in a safe atmosphere.
- Invest time and effort to build a relationship of trust with the person(s) involved. Consider the possibility of using a professionally trained FGM ambassador. Consult FSAN for more information on working with trained FGM ambassadors.
- Provide clarity and explanation about the consequences and everyone's role and responsibilities in the process.
- Because FGM is a form of child abuse, the Reporting Code (English version here) applies. If you suspect a girl has recently undergone FGM or is at risk of undergoing FGM, you have to report this to Veilig Thuis (see Advice/reporting). At Veilig Thuis there are people specially trained on the topic of FGM, commonly the physicians.
- Be aware of your own views and feelings during a conversation and discuss these, when appropriate.
- In case of health problems after FGM, you can refer a girl or woman to one of the consultation hours where nurses and physicians work who are specialised in FGM.

MORE INFORMATION

See the Sources and the fact sheet on honour-based violence. The following 2 documents are particularly useful:

- Appendix 1 in the reporting code for honour-based violence
- The protocol for dealing with FGM among people <18 years of age

ADVICE/REPORTING

For advice, for reporting victims or perpetrators, and/or for referring someone to care (including shelters), call:

- Veilig Thuis ("Veilig Thuis" means "Safe at Home" in Dutch, it is the organization in the Netherlands for advice on, referrals to and reporting of any type of (domestic) violence, abuse, neglect or exploitation, or other types of harm in power-imbalanced relationships). Telephone: **0800 20 00**, free of charge and always open (24 hours per day, 7 days a week). It is possible to call anonymously and/or to call for advice or information only, without reporting someone.
- Pharos Focal Point VGV (advice only)

In case of acute danger call the emergency services at the phone number **112**.

DUTCH TRANSLATION

See here.

This fact sheet was developed by Pharos in collaboration with various chain partners in the fight against female genital mutilation. september 2020
