# Supplementary material for: Lesser-known types of violence: Helping nurses and midwives to signal and act
Source: Int J Nurs Stud Adv. 2022 Sep 17;4:100098. doi: 10.1016/j.ijnsa.2022.100098 (PMC11080451; doi:10.1016/j.ijnsa.2022.100098)
Supplement: Supplementary file 1 [file mmc1.zip › Factsheets English/Financial abuse - sources.pdf]

SOURCES FINANCIAL ABUSE

ORGANISATIONS INVOLVED

The following organisations were involved in making this fact sheet:

- Movisie. For questions and/or remarks about the fact sheet, please email the main author: Nico van Oosten, N.vanOosten@movisie.nl
- Veilig Thuis. Marianne van der Krans, Cathelijne Berkvens, Sigrid van den Boer, Anne-Marie Raat, Laura van der Voorn en Gerda Rosman

SOURCES

The following documents and other sources provide more information about the topic of this fact sheet:

- www.volksgezondheidenzorg.info/onderwerp/sociale-omgeving/ouderenmishandeling#node-financieel-benadeeld-de-thuissituatie-ggd-regio (2016)
- Bakker, L., Witkamp, B., Timmermans, M., Janssen, J., Lindenberg, J. (2018). Aard en omvang ouderenmishandeling. Amsterdam: Regioplan, Avans Hogeschool, Leyden Academy on Vitality and Ageing. www.regioplan.nl/publicaties/slug/type/rapporten/slug/aard_en_omvang_ouderenmishandeling
- Checklist voor ouderen ten behoeve van het voorkomen vanfinanciële uitbuiting. April 2016. Ministerie van Volksgezondheid, Welzijn en Sport in samenwerkingmet de Brede Alliantie 'Veilig financieel ouder worden'. www.aanpak-ouderenmishandeling.nl/doc/Checklist-ouder-voorkomen-van-financiele-uitbuiting.pdf
