# Supplementary material for: Lesser-known types of violence: Helping nurses and midwives to signal and act
Source: Int J Nurs Stud Adv. 2022 Sep 17;4:100098. doi: 10.1016/j.ijnsa.2022.100098 (PMC11080451; doi:10.1016/j.ijnsa.2022.100098)
Supplement: Supplementary file 1 [file mmc1.zip › Factsheets English/Financial abuse.pdf]

FINANCIAL ABUSE

ALWAYS USE THE REPORTING CODE WHEN YOU ENCOUNTER A FORM OF (DOMESTIC) VIOLENCE, ABUSE, NEGLECT OR EXPLOITATION! ▶

This fact sheet is part of a series about *(domestic) violence, abuse, neglect, exploitation* and other types of harm that may be inflicted onto someone in a power-imbalanced relationship. Power-imbalanced relationships can exist with anyone, for example: an (ex-)partner, a child, a parent, a sibling, another family member, an informal or a professional carer, a friend, a flatmate or neighbour, a teacher, a colleague or supervisor, or just someone you know. These fact sheets describe different types of harm that can be inflicted in these relationships. They are meant as an add-on to the Dutch Reporting Code for these issues (English version here) and were developed for two reasons: 1) To provide professionals with an overview of all the types of harm that exist, to aid them in identifying both well-known and lesser-known types (see the Overview). 2) Signs/indicators may vary greatly by type of harm and certain types of harm require specific courses of action; the fact sheets help professionals with identifying the signs/indicators and risk factors of *each specific type* of harm and with acting appropriately when they do. Note: the general 5 steps in the Reporting Code are applicable to all types of harm in power-imbalanced relationships; the factsheets provide more guidance within these 5 steps – they are an add-on, not a replacement.

Below is a brief introduction to this topic, an overview of the signs/indicators and risk factors associated with this type of harm, and points of attention for when you encounter it.

WHAT IS FINANCIAL ABUSE?

Financial abuse is the unwanted and unauthorised use of money and/or goods and/or other possessions of another person by someone in the household. The gravity varies betweenthe victim not realising what's happening, to a criminal offence. This fact sheet is specifically about financial abuse that victimises the elderly.

Examples of financial abuse include: shopping for oneself with someone else's debit card, transferring sums of money to one's own account, theft of - valuable and precious - property, debit card fraud, forced changes in someone's will or mortgage, sale of property, abuse of authorisations, purchases in the name of the victim, keeping someone on a 'short financial leash', i.e. limiting someone's financial freedom.

WHO ARE THE PERPETRATORS?

The perpetrators can be family members, such as (ex-)partners and (grand)children, but also house friends. In addition, they can also be persons the victim is dependent of, such as professional care workers or volunteers.

FACTS AND FIGURES

The 2016 Health Monitor i shows that nationwide, 1.1% of surveyed elderly people report to have experienced financial abuse. There are currently 3.1 million elderly people in the Netherlands, so this computes to 34,100 elderly people. Regional numbers ranged from 0.7 - 2.0%.

In a prevalence study on elder abuse by Regioplan ii one in twenty elderly people experience some form of elder abuse from the age of 65 years onwards. The most commonly reported form of elder abuse by elderly people themselves is financial abuse (3% of all elderly people). When looking at financial abuse *in the past* year, the study found an incidence of 0.9%.

The results of the studies are very similar. Both studies use the same definition for financial abuse and the same age limit of 65 years.

MORE INFORMATION

See the Sources.

This fact sheet was developed by Movisie in collaboration with various chain partners. september 2020

FINANCIAL ABUSE

POSSIBLE SIGNS/INDICATORS:

The signs/indications for financial abuse are as follows:

- Disappearance of money, goods or valuables
- Unexplained cash withdrawals or charges
- Sudden lack of money
- Delays on payments: rent, energy, bills
- Disconnected from gas and electricity
- Increasing number of creditors
- Letters from collection agencies
- Sudden requests for changes of wills in the presence of a family member (the suspected perpetrator)
- Avoiding or refusing to provide information about financial circumstances by an elder or caregiver (the suspected perpetrator)
- Resistance and/or verbal abuse (by the suspected perpetrator) when suspicions are discussed
- Verbal violence or threats (by the suspected perpetrator) against the victim when he or she resists
- Addiction or financial problems with the suspected perpetrator
- Neglected environment and/or appearance with the victim
- Absence of food in the house, signs of malnutrition

N.B. These last two signals can also indicate other problems, like mental decline and a poor social network and/or self-neglect (when someone is no longer able to take care of him-/herself).

RISK FACTORS: WHO IS EXTRA VULNERABLE?

The most important risk factors for financial abuse are the degree of (care) dependency, the relationship that exists between the perpetrator and the victim and the degree of loyalty there is in that relationship, and the circumstances of the perpetrator, such as debts, psychological problems and addiction. Another factor is that elderly people can be too trusting, and have not properly arranged their financial affairs before their health deteriorates to such an extent that they are no longer able to take care of their finances themselves. There is a checklist iii that elderly people can fill in themselves in this regard. On the basis of this checklist, they can decide, potentially in consultation with their relatives, the bank or a notary, whether they want or need to make further arrangements in order to reduce the risk of financial abuse in the future.

The risk factors can broadly be categorized into:

- **Ignorance**: No knowledge and skills of and in digital banking, and consequently leaving financial and administrative matters to others; too little up-to-date knowledge of increasingly complex regulations.
- **Loyalty and family ties:** Through loyalty, family ties, shame, guilt, insecurity (am I right in seeing this?) and/or fear of diminished contact the elderly person does not discuss his/her concerns and signs of abuse with a professional or volunteer. There are harmonious and high-conflict families. Financial abuse partly depends on how family members interact with and relate to one another. This has to do with loyalty and attachment. Parents can allow their children to exploit them for a longer period of time because of feelings of guilt about having failed their children in the past (e.g., because of illness).
  There are children who believe that they have not been given what they are due, that they have been neglected, or abused, by their parents. They may take revenge by financially damaging their parent(s).
  Family disputes can also arise because of claims that children believe they have. With family feuds it often happens that one of the children starts taking up a central role and excludes the other family members. This carries the risk of financial abuse of the parent(s).

ADVICE/REPORTING

For advice, for reporting victims or perpetrators, and/or for referring someone to care (including shelters), call:

- Veilig Thuis ("Veilig Thuis" means "Safe at Home" in Dutch, it is the organization in the Netherlands for advice on, referrals to and reporting of any type of (domestic) violence, abuse, neglect or exploitation, or other types of harm in power-imbalanced relationships). Telephone: **0800 20 00**, free of charge and always open (24 hours per day, 7 days a week). It is possible to call anonymously and/or to call for advice or information only, without reporting someone.

In case of acute danger call the emergency services at the phone number **112**.

DUTCH TRANSLATION

See here.

This fact sheet was developed by Movisie in collaboration with various chain partners.

FINANCIAL ABUSE

- **Physical limitations:** When an elderly person has limitations in terms of hearing, vision and/or mobility, they can run an increased risk because they are not (or no longer) able to (properly) look after their own financial interests.
- **Social limitations:** People with a small social network and few contacts outside the home are at increased risk of becoming victims of financial abuse. The desire for contact sometimes results in a lack of critical assessment about the person with whom the contact is made. This carries the risk that they will hire or engage with an unreliable person, and that this person will abuse their dependency and needs.
- **Psychological problems:** Elderly people are often still mourning the loss of a partner and independence, with depressive feelings influencing their energy to manage their finances properly. In addition, a risk factor is a person's mental decline.
- **Cognitive limitations:** The elderly are more likely to experience financial abuse because they no longer have an overview and lack the skills to control their own finances.

POINTS OF ATTENTION WHEN GOING THROUGH THE 5 STEPS IN THE REPORTING CODE

For any form of (domestic) violence, abuse, neglect or exploitation, professionals in the Netherlands are required to use the Reporting Code. For general reporting code guidelines (such as the 5 steps in this code) visit the link; these are not described in this fact sheet. We do list points of attention in going through the 5 steps that are specific to the topic of this fact sheet. These are:

- Banks and notaries already play an important role in preventing financial abuse.
- Assigning an administrator/custodian and/or reporting the abuse to Veilig Thuis or the police are (extreme) options to stop financial abuse.
- When it comes to theft by fellow clients in an intramural institution, the mental state of the perpetrator should always be taken into account. Psychogeriatric problems, such as dementia, can underlie the behaviour. Objects often reappear.
- This fact sheet has focused on financial abuse among the elderly, however, financial abuse can also occur among other groups of vulnerable citizens such as people with intellectual disabilities or other people who have a strongly power-imbalanced relationship with the perpetrator.

Financial exploitation can also be committed by a professional or by a volunteer. Both fall under the Dutch Quality, Complaints and Disputes in Health Care Act (Wkkgz) and serious incidents must be reported to the Health Care and Youth Care Inspectorate (IGJ).

This fact sheet was developed by Movisie in collaboration with various chain partners. september 2020
