# Supplementary material for: Lesser-known types of violence: Helping nurses and midwives to signal and act
Source: Int J Nurs Stud Adv. 2022 Sep 17;4:100098. doi: 10.1016/j.ijnsa.2022.100098 (PMC11080451; doi:10.1016/j.ijnsa.2022.100098)
Supplement: Supplementary file 1 [file mmc1.zip › Factsheets English/Forced marriage - sources.pdf]

SOURCES FORCED MARRIAGE

ORGANISATIONS INVOLVED

The following organisations were involved in making this fact sheet:

- The Landelijk Knooppunt Huwelijksdwang en Achterlating. For questions and/or remarks about the fact sheet, please email the main author: Diny Flierman, d.flierman@veiligthuishaaglanden.nl
- Augeo Foundation, Edith Geurts
- Bureau Tangram, Suzanne Tan
- CoMensha, Rik Viergever
- Fier - expertise and treatment centre in the field of violence in power-imbalanced relationships, Achille van Hees
- GGD GHOR Netherlands, Annette Duenk and Sandra Hamming
- Landelijk Expertisecentrum Eergerelateerd Geweld, Korps nationale politie, Janine Janssen
- Leger des Heils Jeugdbescherming & Reclassering, Juul Polders
- Movie, Oka Storms
- Sterk Huis, Diane de Winter
- Veilig Thuis, Sabina van der Meer
- Vereniging Vertrouwensartsen Kindermishandeling (VVAK)/ Veilig Thuis, Juliette Heetman
- Verwey-Jonker Instituut, Eliane Smits van Waesberghe

SOURCES

The following documents and other sources provide more information about the topic of this fact sheet:

Documents

- Bakker, H. & Noor, S. (2015). Factsheet huwelijksdwang. Kennisplatform Integratie en Samenleving. www.kis.nl/publicatie/fact sheet-huwelijksdwang
- Bakker, H., Storms, O. (2015). De Meldcode bij (vermoedens van) eergerelateerd geweld. www.movisie.nl/publicatie/meldcode-vermoedens-eergerelateerd-geweld
- Checklist EGG (eergerelateerd geweld). www.politie.nl/themas/eergerelateerd-geweld-voor-professionals.html
- Herken de signalen en ga in gesprek. Tips voor professionals. www.huwelijksdwangenachterlating.nl/sites/www.huwelijksdwangenachterlating.nl/files/downloads/signaalkaart.pdf
- Janssen, J. (2017). Focus op eer. Een verkenning van eerzaken voor politieambtenaren en andere professionals. Den Haag: Boom criminologie. Onder meer voor relatie tussen eergerelateerd geweld en huwelijksdwang.
- Ministerie Sociale Zaken en Werkgelegenheid (2017). Handreiking kindhuwelijken en informele huwelijken. www.rijksoverheid.nl/documenten/publicaties/2017/06/30/handreiking-kindhuwelijke-en-informele-huwelijken
- Smits van Waesberghe, E., Sportel, I., Drost, E., Eijk, E. van, & Diepenbrock, E. (2014). Zo zijn we niet getrouwd. Een onderzoek naar omvang en aard van huwelijksdwang, achterlating en huwelijkse gevangenschap. Utrecht: Verwey-Jonker Instituut. www.verwey-jonker.nl/doc/vitaliteit/7414_Zo%20zijn%20we%20niet%20getrouwd_web.pdf
- Warning signs of victim of forced marriage. westyorkscb.proceduresonline.com/pdfs/warning_signs_diagram.pdf

Websites

- Website Landelijk Knooppunt Huwelijksdwang en Achterlating: www.huwelijksdwangenchterlating.nl
- www.rijksoverheid.nl/onderwerpen/huwelijksdwang/huwelijksdwang-voorkomen
- www.huiselijkgeweld.nl/dossiers/huwelijksdwang
- www.fier.nl/kennis-en-expertise/eergerelateerd-geweld/huwelijksdwang
- www.augeo.nl/Huwelijksdwang
- www.augeo.nl/thema/huwelijksdwang/leren-over-huwelijksdwang
- www.politie.nl/themas/eergerelateerd-geweld.html
- www.nederlandwereldwijd.nl/hulp-bij-nood/huwelijksdwang

This fact sheet was developed by Landelijk Knooppunt Huwelijksdwang en Achterlating in collaboration with various chain partners. september 2020
