# Supplementary material for: Lesser-known types of violence: Helping nurses and midwives to signal and act
Source: Int J Nurs Stud Adv. 2022 Sep 17;4:100098. doi: 10.1016/j.ijnsa.2022.100098 (PMC11080451; doi:10.1016/j.ijnsa.2022.100098)
Supplement: Supplementary file 1 [file mmc1.zip › Factsheets English/Forced marriage.pdf]

FORCED MARRIAGE

ALWAYS USE THE REPORTING CODE WHEN YOU ENCOUNTER A FORM OF (DOMESTIC) VIOLENCE, ABUSE, NEGLECT OR EXPLOITATION! ▶

This fact sheet is part of a series about *(domestic) violence, abuse, neglect, exploitation* and other types of harm that may be inflicted onto someone in a power-imbalanced relationship. Power-imbalanced relationships can exist with anyone, for example: an (ex-)partner, a child, a parent, a sibling, another family member, an informal or a professional carer, a friend, a flatmate or neighbour, a teacher, a colleague or supervisor, or just someone you know. These fact sheets describe different types of harm that can be inflicted in these relationships. They are meant as an add-on to the Dutch Reporting Code for these issues (English version here) and were developed for two reasons: 1) To provide professionals with an overview of all the types of harm that exist, to aid them in identifying both well-known and lesser-known types (see the Overview). 2) Signs/indicators may vary greatly by type of harm and certain types of harm require specific courses of action; the fact sheets help professionals with identifying the signs/indicators and risk factors of *each specific type* of harm and with acting appropriately when they do. Note: the general 5 steps in the Reporting Code are applicable to all types of harm in power-imbalanced relationships; the factsheets provide more guidance within these 5 steps – they are an add-on, not a replacement.

Below is a brief introduction to this topic, an overview of the signs/indicators and risk factors associated with this type of harm, and points of attention for when you encounter it.

WHAT IS FORCED MARRIAGE?

A forced marriage is a marriage where the consent of the bride, groom or both is obtained through coercion by someone's parents, family or community. One or both spouses have no control over and do not consent to the marriage. There is no free partner choice or they must marry before a certain age. The marriage can be formal (legal) or informal. Family members or the community may exert pressure, for instance, emotional pressure or physical abuse. This pressure can be subtle to very compelling. Forced marriage is a form of domestic violence, can be honour-based, illegal and a criminal offence.

POSSIBLE SIGNS/INDICATORS: HOW TO IDENTIFY IT

Forced marriages are usually hidden from view. Sometimes victims show slight resistance, but out of fear, shame or loyalty to the family, they usually comply with the marital choice of the parents or community. These signs can be an indication of forced marriage:

- Behaviour change: withdrawn, afraid, angry, aggressive
- Spending less time with friends
- Does not answer phone or messages
- Suddenly different clothing
- Signs of abuse or violence
- Self-injury, suicide attempts, eating disorder
- Early or unwanted pregnancy

KEY FACTS

- The number of victims of forced marriage in the Netherlands is estimated to be between 338 and 957 per year. That is more than the number of reports of 181 per year.
- Forced marriage occurs especially among young people between 16 and 25.
- Forced marriage occurs in different ethnic and religious communities: Afghanistan, Bosnia, Bulgaria, Egypt, India, Indonesia, Iraq, Iran, Kurdish, Morocco, Pakistan, Poland, Somalia, Turkey, Suriname/Hindustani and among Sikhs and Roma
- Forced marriage takes place within family traditions, for interests' sake or solving a problem

ADVICE/REPORTING

For advice, for reporting victims or perpetrators, and/or for referring someone to care (including shelters), call:

- Veilig Thuis ("Veilig Thuis" means "Safe at Home" in Dutch, it is the organization in the Netherlands for advice on, referrals to and reporting of any type of (domestic) violence, abuse, neglect or exploitation, or other types of harm in power-imbalanced relationships). Telephone: **0800 20 00**, free of charge and always open (24 hours per day, 7 days a week).

This fact sheet was developed by Landelijk Knooppunt Huwelijksdwang en Achterlating in collaboration with various chain partners. september 2020

Warning signs at school:

- Frequent or long-term absence
- Request long family visits abroad
- Unexpected trip abroad
- Is being controlled Suddenly drop out of school
- No extracurricular activities
- Poor school performance

Warning signs at work:

- Not allowed to work or not flexible
- Frequent or long-term absence
- Poor performance
- Limited career opportunities
- Financial control
- Forced to hand over salary
- Is picked up at work

RISK FACTORS: WHO IS EXTRA VULNERABLE?

Forced marriage is more likely to occur to:

- Young women (more often) and men from closed communities where traditional ideas prevail on the role, position and sexuality of women and men.
- Migration background, honour culture, orthodox religious or high socio-economic status
- Young women and men in a highly dependent position, without a network outside the family or own income
- Persons with a dependent residence permit (see also the fact sheet about vulnerable migrants)

POINTS OF ATTENTION WHEN GOING THROUGH THE 5 STEPS IN THE REPORTING CODE

For any form of (domestic) violence, abuse, neglect or exploitation, professionals in the Netherlands are required to use the Reporting Code. For general reporting code guidelines (such as the 5 steps in this code) visit the link; these are not described in this fact sheet. We do describe here points of attention that are specific to the topic of this fact sheet. These are:

- Ask for advice and always consult an expert (see "Advice/report" below). The collective nature of marriage coercion requires specific expertise.
- Contact the police immediately if there is an acute threat to security.
- Talk to the partner, parents/family only AFTER the situation and risks of such a conversation have been assessed with the help of an expert.
- When the victim is already abroad: do not talk to parents, partner or family for safety reasons.

MORE INFORMATION

See the Sources.

It is possible to call anonymously and/or to call for advice or information only, without reporting someone.

- The Landelijk Knooppunt Huwelijksdwang en Achterlating 070 34 54 319

IF YOU, OR THE PERSON YOU KNOW, IS ALREADY ABROAD:

- If the victim is abroad, contact the Landelijk Knooppunt Huwelijksdwang en Achterlating directly.
- If you need help abroad, please contact the Dutch embassy.

YOUNG PEOPLE:

- Young people can chat anonymously; Eva and Zahir are national expertise and treatment centres.

ACUTE DANGER:

- In case of acute danger call the emergency services at the phone number **112**.

DUTCH TRANSLATION

See here.

This fact sheet was developed by Landelijk Knooppunt Huwelijksdwang en Achterlating in collaboration with various chain partners. september 2020
