# Supplementary material for: Lesser-known types of violence: Helping nurses and midwives to signal and act
Source: Int J Nurs Stud Adv. 2022 Sep 17;4:100098. doi: 10.1016/j.ijnsa.2022.100098 (PMC11080451; doi:10.1016/j.ijnsa.2022.100098)
Supplement: Supplementary file 1 [file mmc1.zip › Factsheets English/Hidden women - sources.pdf]

SOURCES HIDDEN WOMEN

ORGANISATIONS INVOLVED

The following organisations were involved in making this fact sheet:

- Verwey-Jonker Institute. For questions and/or remarks about the fact sheet, please email the main authors: Lisanne Drost and Eliane Smits van Waesberghe, at secr@verwey-jonker.nl
- Sterk Huis, Diane de Winter
- Bureau Tangram, Suzanne Tan
- Politie, Janine Janssen
- Movisie, Oka Storms / Wilma Schakenraad
- Landelijk Knooppunt Huwelijksdwang en Achterlating, Diny Flierman
- Veilig Thuis, Juliette Heetman
- GGD GHOR, Sandra Hamming

SOURCES

The following documents and other sources provide more information about the topic of this fact sheet:

- Drost, L., Smits van Waesberghe, E., Los, V. (2015). Opgesloten in eigen huis. Een onderzoek naar aard en omvang van verborgen vrouwen in Den Haag. Utrecht: Verwey-Jonker Instituut. www.verwey-jonker.nl/doc/2015/115002-verborgen-vrouwen-denhaag.pdf
- Drost, L., Goderie, M., Flikweert, M., & Tan, S. (2012). Leven in gedwongen isolement: Een verkennend onderzoek naar verborgen vrouwen in Amsterdam. Utrecht: Verwey-Jonker Instituut. www.verwey-jonker.nl/doc/vitaliteit/Verborgen_vrouwen_7249_web.pdf
- Informatieblad verborgen vrouwen gemeente Rotterdam: www.huiselijkgeweld.nl/doc/Informatieblad%20verborgen%20vrouwen%202016.pdf
- J. Janssen, Focus op eer. Een verkenning van eerzaken voor politieambtenaren en andere professionals, Den Haag: Boom Criminologie, 2017.
- Musa, S., Diepenbrock, E. (2013). Verborgen vrouwen: een vergeten groep. Een verkennend onderzoek naar aard, omvang en aanpak van de problematiek van verborgen vrouwen in de deelgemeente Delfshaven (Rotterdam). S.l.: Stichting Femmes For Freedom.
- Nieuwsbrief gemeente Rotterdam verborgen vrouwen, juli 2017 www.rotterdam.nl/wonen-leven/schadelijke-praktijken/NB2-verborgen-vrouwen.pdf
- www.politie.nl/themas/eergerelateerd-geweld-voor-professionals.html

This fact sheet was developed by Verwey-Jonker Instituut / Kennisplatform Integratie en Samenleving in collaboration with various chain partners.

september 2020
