# Supplementary material for: Lesser-known types of violence: Helping nurses and midwives to signal and act
Source: Int J Nurs Stud Adv. 2022 Sep 17;4:100098. doi: 10.1016/j.ijnsa.2022.100098 (PMC11080451; doi:10.1016/j.ijnsa.2022.100098)
Supplement: Supplementary file 1 [file mmc1.zip › Factsheets English/Hidden women.pdf]

HIDDEN WOMEN

ALWAYS USE THE REPORTING CODE WHEN YOU ENCOUNTER A FORM OF (DOMESTIC) VIOLENCE, ABUSE, NEGLECT OR EXPLOITATION! ▶

This fact sheet is part of a series about *(domestic) violence, abuse, neglect, exploitation* and other types of harm that may be inflicted onto someone in a power-imbalanced relationship. Power-imbalanced relationships can exist with anyone, for example: an (ex-)partner, a child, a parent, a sibling, another family member, an informal or a professional carer, a friend, a flatmate or neighbour, a teacher, a colleague or supervisor, or just someone you know. These fact sheets describe different types of harm that can be inflicted in these relationships. They are meant as an add-on to the Dutch Reporting Code for these issues (English version here) and were developed for two reasons: 1) To provide professionals with an overview of all the types of harm that exist, to aid them in identifying both well-known and lesser-known types (see the Overview). 2) Signs/indicators may vary greatly by type of harm and certain types of harm require specific courses of action; the fact sheets help professionals with identifying the signs/indicators and risk factors of *each specific type* of harm and with acting appropriately when they do. Note: the general 5 steps in the Reporting Code are applicable to all types of harm in power-imbalanced relationships; the factsheets provide more guidance within these 5 steps – they are an add-on, not a replacement.

Below is a brief introduction to this topic, an overview of the signs/indicators and risk factors associated with this type of harm, and points of attention for when you encounter it.

WHO/WHAT ARE HIDDEN WOMEN?

Hidden women are women who are forced by their partner, family and/or in-laws to live in isolation. This is often accompanied by psychological intimidation and/or (threat of) physical violence. These women are often locked up in their home against their own will and may have no or only very limited contact with others. In cases of contact outside the home, this often happens under the supervision and control of a partner, family and/or in-laws. Hidden women have little to no opportunity to participate in society, are not or hardly accessible for aid workers, and do not know how to access aid themselves (or only after a long time).

POSSIBLE SIGNS/INDICATORS: HOW TO IDENTIFY IT

- The woman does not or hardly gets out of the house.
- The woman does not show up for appointments.
- The woman is checked or accompanied by partner, family members or in-laws when she goes outside.
- The person who accompanies the woman always speaks on her behalf in conversations with professionals.
- Psychosomatic complaints.
- Domestic violence.
- The door of the house remains closed and windows are darkened.
- The woman looks anxious, timid and/or makes a lifeless impression.
- Neighbourhood children or classmates of children are never allowed to come and play.
- Children appear unkempt, anxious and/or avoiding.

FACTS AND FIGURES

Figures

It is estimated that there are a total of 600-900 hidden women living in the Netherlands in the big cities of Rotterdam, Amsterdam and The Hague alone. National figures are missing.

Facts

- Most of the hidden women come from Morocco and Turkey.
- Hidden women are women of all ages.
- The husband and his family often play an important role in keeping the woman hidden.
- The arrival or presence of children makes it even more difficult for women to break through their isolation.
- Hidden women are more likely to experience psychological harassment and physical violence.

MORE INFORMATION

See the Sources.

This fact sheet was developed by Verwey-Jonker Instituut / Kennisplatform Integratie en Samenleving in collaboration with various chain partners.

HIDDEN WOMEN

- The woman indicates that she is afraid of her husband or family.
- The woman does not want help, out of fear of more problems or violence.
- Forced residence with in-laws or family.

RISK GROUPS: AMONG WHICH GROUPS IS IT MORE PREVALENT?

- Forced residence with in-laws or family.
- Women from a closed community in which traditional ideas prevail about the role and position of women. Also communities where traditions and family honour play a major role.
- Women who migrate to the Netherlands for marriage.
- Women without a residency status or who are dependent on their partner for a residency permit.
- Women for whom a marriage is arranged with a man with a mental or physical disability.
- Women who marry a partner under pressure from the family (forced marriage).
- Women who are married to a partner with psychiatric problems, such as extreme suspicion, jealousy or paranoid behavior.

RISK FACTORS: WHO IS EXTRA VULNERABLE?

- Social pressure and control by partner, family, in-laws and/or community.
- Family honour.
- Low literacy, illiteracy and/or not being able to speak the language of the country where she lives.
- Financial dependence.
- Living in a house with the in-laws.
- Being new in a country and not knowing their way around.
- Being dependent on someone else (e.g. a partner) for their residency permit.

POINTS OF ATTENTION WHEN GOING THROUGH THE 5 STEPS IN THE REPORTING CODE

For any form of (domestic) violence, abuse, neglect or exploitation, professionals in the Netherlands are required to use the Reporting Code. For general reporting code guidelines (such as the 5 steps in this code) visit the link; these are not described in this fact sheet. We do describe here points of attention in going through the 5 steps that are specific to the topic of this fact sheet. These are:

- Professionals in primary care and education are the most important potential signers of hidden women, because these women hardly participate in society.
- Both the women themselves, and their potential children, should remain in view of social workers.
- If a hidden woman is in view of these professionals, then it is important for a professional to connect to the needs of the woman and any children: she determines the pace.
- A professional can make it explicit that men and women in the Netherlands have equal rights to participate in society (in accordance with the UN Convention on Women). Some hidden women do not know that forced isolation is punishable in the Netherlands and help is available.
- If the woman explicitly indicates that she wants to end her isolation, then, as a professional, make sure that the next safe steps are taken in consultation with Veilig Thuis ("Veilig Thuis" means "Safe at Home" in Dutch, it is the organization in the Netherlands for advice on, referrals to and reporting of any type of (domestic) violence, abuse, neglect or exploitation, or other types of harm in power-imbalanced relationships).

ADVICE/REPORTING

For advice on this type of harm, reporting victims or perpetrators, or referring someone to care (including shelters), call:

- Veilig Thuis. Telephone: **0800 20 00**, free of charge and always open (24 hours per day, 7 days a week). It is possible to call anonymously and/or to call for advice or information only, without reporting someone.

In case of acute danger call the emergency services at the phone number **112.**

DUTCH TRANSLATION

See here.

This fact sheet was developed by Verwey-Jonker Instituut / Kennisplatform Integratie en Samenleving in collaboration with various chain partners.
