# Supplementary material for: Lesser-known types of violence: Helping nurses and midwives to signal and act
Source: Int J Nurs Stud Adv. 2022 Sep 17;4:100098. doi: 10.1016/j.ijnsa.2022.100098 (PMC11080451; doi:10.1016/j.ijnsa.2022.100098)
Supplement: Supplementary file 1 [file mmc1.zip › Factsheets English/Honour-based violence - sources.pdf]

SOURCES HONOUR-BASED VIOLENCE

ORGANISATIONS INVOLVED

The following organisations were involved in making this fact sheet:

- Fier - expertise en behandelcentrum op het terrein van geweld in afhankelijkheidsrelaties. For questions and/ or remarks about the fact sheet, please email the main author: Gerda de Groot, GdeGroot@fier.nl
- Samen Veilig (Veilig Thuis regio Utrecht)
- Suzanne Tan, bureau TANGRAM
- Diny Flierman, Landelijk Knooppunt Huwelijksdwang en Achterlating
- Sandra Hamming, GGD GHOR
- Marga Haagmans, AUGEO
- Janine Janssen, LEC EGG

SOURCES

The following documents and other sources provide more information about the topic of this fact sheet:

Background

- Bakker, H., Storms, O. (2016). Factsheet De meldcode bij (vermoedens van) eergerelateerd geweld. Utrecht: Movisie.
- Brekelmans, I. & Groot, G. de (2014). De kleuren van Eva en Zahir. Amsterdam: SWP Uitgeverij.
- Dijke, A. van & Terpstra, L. (2010). De dochters van Zahir. Tussen traditie en wereldburgerschap. Amsterdam: SWP Uitgeverij.
- Ermers, R. (2018). Honor Related Violence. A new social psychological Perspective). Routledge, www.routledge.com
- Ferwerda, H.B & Leiden, I. van (2005). Eerwraak of eergerelateerd geweld? Naar een werkdefinitie. Arnhem: Advies- en Onderzoekgroep Beke.
- Janssen, J. & Sanberg, R. (2013) Uniformiteit in cijfers. Mogelijke eerzaken in 2010, 2011 en 2012. Den Haag: Landelijk Expertise Centrum Eer Gerelateerd Geweld.
- Janssen, J. (2013). De rol van religie bij het afbakenen, verklaren en aanpakken van eergerelateerd geweld. Tijdschrift voor Religie, Recht en Beleid (4)1: 5-15.
- Janssen, J. (2017). Focus op eer. Een verkenning van eerzaken voor politieambtenaren en andere professionals. Den Haag: Boom Criminologie.
- Vlamings, B., Geijn. R. van, Brekelmans, I. (2012). Methodiek Safe and Streetwise. Tilburg: Kompaan en De Bocht.
- Werson, S., Lamers, F., Pers, M. van der, & Dijke, A. van (2015). Fier en verder. Meiden over hun leven na de hulpverlening. Amsterdam: Uitgeverij SWP.

Fact sheets on specific forms of honour-based violence

- Female Genital Mutilation (FGM)
- Forced marriages
- Forced abandonment
- Forced isolation

National expertise and treatment centres

- www.fier.nl
- www.sterkhuis.nl

Internet

- www.huiselijkgeweld.nl/dossiers/eergerelateerdgeweld
- www.rijksoverheid.nl/onderwerpen/eergerelateerd-geweld
- www.huwelijksdwangenachterlating.nl
- www.movisie.nl/publicatie/eergerelateerd-geweld-seksuele-genderdiversiteit
- www.politie.nl/themas/eergerelateerd-geweld.html
- www.politie.nl/themas/eergerelateerd-geweld-voor-professionals.html
- www.eerenvrijheid.nl
- www.kis.nl/trefwoorden/eergerelateerd-geweld
- www.huiselijkgeweld.nl/nieuws/2018/270218_-eergerelateerd-geweld-is-geen-huiselijk-geweld-
- www.huiselijkgeweld.nl/doc/feiten/factsheet_EG_20p_aug%202011.pdf

Signs/indicators

- www.leceergerelateerdgeweld.nl/herkennen

Reporting and referral code

- www.meldcode.nu/eergerelateerd-geweld
