# Supplementary material for: Lesser-known types of violence: Helping nurses and midwives to signal and act
Source: Int J Nurs Stud Adv. 2022 Sep 17;4:100098. doi: 10.1016/j.ijnsa.2022.100098 (PMC11080451; doi:10.1016/j.ijnsa.2022.100098)
Supplement: Supplementary file 1 [file mmc1.zip › Factsheets English/Honour-based violence.pdf]

HONOUR-BASED VIOLENCE

This fact sheet is part of a series about *(domestic) violence, abuse, neglect, exploitation* and other types of harm that may be inflicted onto someone in a power-imbalanced relationship. Power-imbalanced relationships can exist with anyone, for example: an (ex-)partner, a child, a parent, a sibling, another family member, an informal or a professional carer, a friend, a flatmate or neighbour, a teacher, a colleague or supervisor, or just someone you know. These fact sheets describe different types of harm that can be inflicted in these relationships. They are meant as an add-on to the Dutch Reporting Code for these issues (English version here) and were developed for two reasons: 1) To provide professionals with an overview of all the types of harm that exist, to aid them in identifying both well-known and lesser-known types (see the Overview). 2) Signs/indicators may vary greatly by type of harm and certain types of harm require specific courses of action; the fact sheets help professionals with identifying the signs/indicators and risk factors of *each specific type* of harm and with acting appropriately when they do. Note: the general 5 steps in the Reporting Code are applicable to all types of harm in power-imbalanced relationships; the factsheets provide more guidance within these 5 steps – they are an add-on, not a replacement.

Below is a brief introduction to this topic, an overview of the signs/indicators and risk factors associated with this type of harm, and points of attention for when you encounter it.

HONOUR

Honour has to do with the reputation of integrity and reliability.

In some families and communities family honour plays an important role. Moral norms regarding sexuality determine the relationship between men and women. If these standards are used in one's family, then 'you are part of it'. When a person's reputation is not good owing to moral misconduct, he/she is considered immoral and therefore a bad person. As a result, the individual can be excluded. Immoral behaviour of a family member can also seriously damage the reputation of the integrity and reliability (family honour) of other family members with the risk of family exclusion by the community. The family will do everything in its power to prevent exclusion.

Individual choices thus have a direct influence on the position of family members and the position in the community. In these families the sense of shame of "undesirable" behaviour and the fear that this behaviour will be known to third parties are important factors.

ALWAYS USE THE REPORTING CODE WHEN YOU ENCOUNTER A FORM OF (DOMESTIC) VIOLENCE, ABUSE, NEGLECT OR EXPLOITATION! ▶

FACTS AND FIGURES

Every year the Dutch police see 2,500 - 3,000 offences in which family honour plays a (presumed) role. An average of 460 offences is referred to the 'Landelijk Expertisecentrum Eergerelateerd Geweld' because of their complexity. Of these, 7 to 17 end fatally (murder, manslaughter or suicide).

ADVICE/REPORTING

For advice, for reporting victims or perpetrators, and/or for referring someone to care (including shelters), call:

- Veilig Thuis ("Veilig Thuis" means "Safe at Home" in Dutch, it is the organization in the Netherlands for advice on, referrals to and reporting of any type of (domestic) violence, abuse, neglect or exploitation, or other types of harm in power-imbalanced relationships). Telephone: **0800 20 00**, free of charge and always open (24 hours per day, 7 days a week). It is possible to call anonymously and/or to call for advice or information only, without reporting someone.

In case of acute danger call the emergency services at the phone number **112**.

This fact sheet was developed by Fier in collaboration with various chain partners. september 2020

HONOUR-BASED VIOLENCE

HONOUR-BASED VIOLENCE

Honour-based violence is the umbrella term for forms of intimidation, coercion and psychological and physical violence committed from an honour-based motive. The purpose of the violence is to prevent a family member from behaving in a way that could harm the family honour. There are often orthodox views on the role of women and men, and on the sexuality and reproductive rights of women. The honour of the woman is linked to her sexuality. And the sexual honour of the woman is linked to the family honour. The violence always concerns behaviour that has become known to people in the environment of the family.
A commonly used definition: 'Honour-based violence includes any form of mental or physical violence, committed from a collective mentality in response to a (threat of) violation of the honour of a man or a woman and thus of his or her family, of which the outside world is aware or is at risk of becoming aware' (Working definition Ministry of Justice, Beke 2005).

Honour-based violence can take place both between an individual and the group and between different groups. If there is a risk of honour violations, these must be prevented by the family. When honour is violated, it must be restored by the family. The family is addressed by the community if it does not respond sufficiently to an infringement of the family honour. Girls and women have the responsibility to uphold the family honour; parents, men and boys to guard and if necessary restore it.

FORMS OF HONOUR-BASED VIOLENCE

Honour related violence has different manifestations and degrees of seriousness, for example threatening, great psychological pressure, restricting freedoms, controlling, isolating, healing rituals and incantations, (forced) abortion / abandonment of a child, physical violence, forced marriage, marital imprisonment, rejection, forced abandonment abroad, (female genital) mutilation, being forced to commit suicide, and honour killings.

It is important that a distinction is made between: honour-based violence; domestic violence, violence for the sake of the individual's honour; incapacity to educate; and child abuse. In the case of honour-based violence a motive of honour is explicitly present in a collective context; other interventions are appropriate in those situations.

POSSIBLE SIGNS/INDICATORS: HOW TO IDENTIFY IT

Signs/indicators are non-specific, i.e. there are no specific signals of honour-based violence.

Signs that *may* indicate honour-based violence are:
A fear of family violence, behavioural changes and or complaints such as depression, negative self-image, self-harm, eating problems and bad sleep, walking away from home, (structural) absenteeism from school and or daytime activities, reduced performance in school or at work, not looking forward to upcoming holidays, (suddenly) being met and/or brought by family members (surveillance), withholding medical care, breaking social contacts without clear reason, change of clothing, sudden announcement of engagement and / or marriage, gossip in the community, no longer having access to identity documents.

Shelter and help can be arranged in the region. For consultation / advice and specific shelter and treatment, call national one of the expertise centres: Sterk Huis at 013 54 33 073 or Fier **088 20 80 000** or www.chatmetfier.nl

For advice on marriage coercion and abandonment, call:

- Landelijk Knooppunt Huwelijksdwang en Achterlating (LKHA) at **070 34 54 319**

MORE INFORMATION

See the Sources and the 4 other fact sheets about in this series about specific forms of Honour-based violence:

- marriage coercion
- forced abandonment
- female genital mutilation
- hidden women

DUTCH TRANSLATION

See here.

This fact sheet was developed by Fier in collaboration with various chain partners. september 2020

HONOUR-BASED VIOLENCE

Please note: there are almost always feelings of guilt and shame and conflicts of loyalty.

RISK FACTORS: WHO IS EXTRA VULNERABLE?

- Women: a 'supposed' relationship, sex before marriage, a [supposed] extramarital relationship, unmarried pregnancy, refusing an arranged marriage, lesbian identity and/or relationship;
- Men: relationship/sex with a girl who is not allowed this by her family, relationship/sex with a married woman, refusing an arranged marriage, homosexual identity and/or relationship, refusing to carry out honour-based violence with regard to sister, niece or other family member;
- Bad contacts who, for example, threaten to place compromising images on the internet (sexting / digital blackmail);
- Sharp social control, gossip about (alleged) misconduct, misconduct is known in the area;
- (Threatening) honour-based violence is discussed with police / assistance (this can become known in the community);
- High interdependence within a family where family honour is important;
- Previous honour-based violence in the family;
- Strict / traditional moral standards with respect to moral family honour;
- Socio-economic problems, social isolation, multi-problem families.

POINTS OF ATTENTION WHEN GOING THROUGH THE 5 STEPS IN THE REPORTING CODE

For any form of (domestic) violence, abuse, neglect or exploitation, professionals in the Netherlands are required to use the Reporting Code. For general reporting code guidelines (such as the 5 steps in this code) visit the link; these are not described in this fact sheet. We do describe here points of attention in going through the 5 steps that are specific to the topic of this fact sheet. These are:

- Risk assessment and analysis always by police experts using a checklist for honour-based violence;
- It is very important not to involve or inform parents directly because of the risk of escalation and potential shame;
- There is a specific reporting code for honour-based violence;
- Via the police and Veilig Thuis a consultation, valuation and analysis can be performed by the 'Landelijk Expertisecentrum Eergerelateerd geweld'
- If in doubt: see at "Advice/reporting".

This fact sheet was developed by Fier in collaboration with various chain partners. september 2020
