# Supplementary material for: Lesser-known types of violence: Helping nurses and midwives to signal and act
Source: Int J Nurs Stud Adv. 2022 Sep 17;4:100098. doi: 10.1016/j.ijnsa.2022.100098 (PMC11080451; doi:10.1016/j.ijnsa.2022.100098)
Supplement: Supplementary file 1 [file mmc1.zip › Factsheets English/Line-crossing among young people.pdf]

LINE-CROSSING SEXUAL BEHAVIOUR AMONG YOUNG PEOPLE

ALWAYS USE THE REPORTING CODE WHEN YOU ENCOUNTER A FORM OF (DOMESTIC) VIOLENCE, ABUSE, NEGLECT OR EXPLOITATION! ▶

This fact sheet is part of a series about *(domestic) violence, abuse, neglect, exploitation* and other types of harm that may be inflicted onto someone in a power-imbalanced relationship. Power-imbalanced relationships can exist with anyone, for example: an (ex-)partner, a child, a parent, a sibling, another family member, an informal or a professional carer, a friend, a flatmate or neighbour, a teacher, a colleague or supervisor, or just someone you know. These fact sheets describe different types of harm that can be inflicted in these relationships. They are meant as an add-on to the Dutch Reporting Code for these issues (English version here) and were developed for two reasons: 1) To provide professionals with an overview of all the types of harm that exist, to aid them in identifying both well-known and lesser-known types (see the Overview). 2) Signs/indicators may vary greatly by type of harm and certain types of harm require specific courses of action; the fact sheets help professionals with identifying the signs/indicators and risk factors of *each specific type* of harm and with acting appropriately when they do. Note: the general 5 steps in the Reporting Code are applicable to all types of harm in power-imbalanced relationships; the factsheets provide more guidance within these 5 steps – they are an add-on, not a replacement.

Below is a brief introduction to this topic, an overview of the signs/indicators and risk factors associated with this type of harm, and points of attention for when you encounter it.

WHAT IS LINE-CROSSING SEXUAL BEHAVIOUR BETWEEN YOUNGSTERS?

Line- crossing sexual behaviour refers to behaviour or approaches that are sexual in nature and cross the boundaries of one or more of the people involved. This may or may not be physical (De Haas, 2012).

To determine whether or not sexual behaviour crosses someone's boundaries, we start from a number of fundamental criteria or social values, based on rights, legislation and regulation. The point of view here is that of the person responsible for the behaviour (Frans, De Wilde, Janssens, van Berlo & Storms, 2016): (1) mutual consent; (2) voluntary action; (3) equivalence; (4) appropriate to age or development or age; (5) appropriate to the context; (6) self-respect (see also the Sensoa Flagsystem© , Frans & Franck; 2010; 2014).

This fact sheet is concerned with sexual behaviours among children and adolescents. In the case of positive and acceptable sexual behaviour of children and adolescents, all criteria are met. When one or more of the criteria are not met, this is defined as sexual behaviour that crosses boundaries. We speak of sexual abuse for any form of sexual behaviour that crosses boundaries in a verbal or physical sense, whether intentional or unintentional, which clearly does not have mutual consent; and/or which is somehow enforced and/or where the victim is much younger or

NUMBERS

The study Seks onder je 25e by Rutgers (De Graaf et al. , 2017) shows that 2% of boys and 11% of girls are sometimes forced into sexual acts that they did not want to engage in.

MORE INFORMATION

See the Sources and these websites:

- www.act4respect.nl
- www.rutgers.nl
- www.atria.nl
- www.vlaggensysteem.nl

This fact sheet was developed by Movisie in collaboration with various chain partners. september 2020

in a dependent relationship (Frans et a. , 2016; Frans & Franck, 2014).

Hands-off and hands-on

When discussing line-crossing sexual behaviour, we distinguish between so-called hands-off and hands-on behaviour. Hands-off behaviour does not involve physical contact and involves, for example, unwanted and/or offensive sexual remarks or sexting (making and sending sexually-tinged messages or spicy photos and videos) or being forced to watch porn. In the case of hands-on behaviour, there is physical contact. This concerns sexual touching, intrusion, ranging from kissing (forced tongue kissing) to rape, the most serious form of line-crossing sexual behaviour, which is a form of sexual assault.

Young people experiment and sometimes cross boundaries

When young people's sexual boundaries are crossed it is often by peers.

A part of the developmental stage in young people's lives is (sexual) experimentation. Sometimes young people go beyond their own boundaries or those of another. They are sometimes insufficiently aware of their own limits and those of others, they do not know how to recognise these limits in someone else, or they find it difficult to say out loud what they like or dislike. Group pressure can also play a role; and/or there can be the influence of alcohol or other drugs.

(Professional) educators and parents therefore have the task of guiding young people in this, and to make young people aware of the importance of understanding sexual preferences and boundaries.

AT-RISK GROUPS: AMONG WHICH GROUPS IS IT MORE PREVALENT?

At-Risk groups for line-crossing sexual behaviour between young people:

- Girls are generally more at risk than boys.
- Lesbian, homosexual, bisexual or transgender youth.
- Young people with a low level of education
- Young people with a disability (physical or mental, also a mild mental disability).
- Young people staying in institutions.
- Young people with negative childhood experiences of physical abuse, emotional neglect and/or sexual abuse.

RISK FACTORS: WHO IS EXTRA VULNERABLE?

In addition to the risk groups mentioned above, there are also *individual and relational risk factors*. These include a lack of knowledge and skills among children and adolescents to assess sexual behaviour, a relationship with parents that is lacking in trust, and previous experiences with sexual behaviour that crossed boundaries (so-called revictimization). Young people with a low self-esteem, ADHD, or an autism spectrum disorder that results in insufficient awareness of what another person is experiencing are also at greater risk.

ADVICE/REPORTING

When the sexual violence is recent, contact:

- The Centrum Seksueel Geweld (CSG) (this name means "Centre for Sexual Violence" in Dutch) is the expertise centre of the Netherlands for victims of sexual violence in the acute phase (<7 days). At CSG, a team of doctors, nurses, police, psychologists, social workers and sexologists work together to provide specialist care to victims of assault and rape. Call 0800-0188.

When the sexual violence is less recent, contact:

- Veilig Thuis ("Veilig Thuis" means "Safe at Home" in Dutch, it is the organization in the Netherlands for advice on, referrals to and reporting of any type of (domestic) violence, abuse, neglect or exploitation, or other types of harm in power-imbalanced relationships). Telephone: **0800 20 00**, free of charge and always open (24 hours per day, 7 days a week). It is possible to call anonymously and/or to call for advice or information only, without reporting someone.

In case of acute danger call the emergency services at the phone number **112**.

DUTCH TRANSLATION

See here.

This fact sheet was developed by Movisie in collaboration with various chain partners. september 2020

LINE-CROSSING SEXUAL BEHAVIOUR AMONG YOUNG PEOPLE

Social norms and developments that increase the incidence of sexual behaviour that crosses boundaries include: traditional gender roles, gender stereotypes, double standards and changing realities. What also plays a role in this regard is that sexuality is more prevalent in the mass media today (Römkens, 2017) and that 'biological maturation' starts earlier in children/young people now than in the past (Storms & Doornink, 2016). Another social development of relevance is that young people today show more experimental behaviour and (ab)use alcohol and drugs more often.

GUIDELINES FOR PROFESSIONALS

The Sensoa Flagsystem© (Frans & Franck, 2010; 2014) offers professional educators tools to adequately assess sexual behaviour, to opening it up to discussing and to react appropriately. This is done on the basis of six criteria to determine whether the behaviour is healthy or crosses boundaries: consent, voluntary action, equivalence, age or development adequacy, context adequacy and self-respect. Specific attention is paid to gender and cultural aspects; as well as to children and young people with disabilities and/or trauma (Frans et al. , 2016).

What does the law say?

Sex with a minor (defined for this law as <16 years of age) is punishable in principle, even if there was mutual consent. However, between the ages of 12 and 16, the Public Prosecution Service asks the young person for his or her opinion, 'if possible' and in certain cases (art. 167a of the Code of Criminal Procedure). A 17-year-old boy who has sexual contact with a 15-year-old girl who has no objection to this, will most likely not be charged with an offence. Sex with a child under 12 is always punishable.

This fact sheet was developed by Movisie in collaboration with various chain partners.

september 2020
