# Supplementary material for: Lesser-known types of violence: Helping nurses and midwives to signal and act
Source: Int J Nurs Stud Adv. 2022 Sep 17;4:100098. doi: 10.1016/j.ijnsa.2022.100098 (PMC11080451; doi:10.1016/j.ijnsa.2022.100098)
Supplement: Supplementary file 1 [file mmc1.zip › Factsheets English/Male abuse - sources.pdf]

SOURCES MALE ABUSE

ORGANISATIONS INVOLVED

The following organisations were involved in making this fact sheet:

- Blijf Groep, Stichting Wende en Veilig Thuis. For questions and/or remarks about the fact sheet, please email the main authors: Claire Loeber, C.Loeber@blijfgroep.nl , Carla Scherpenhuijzen, c.scherpenhuijzen@perspektief.nl , Floor van Niekerk, en Ries Wilschut, RWilschut@samen-veilig.nl
- Robert Weinberg, Blijf groep

SOURCES

The following documents and other sources provide more information about the topic of this fact sheet:

- Nanhoe, A., *Pionieren in de mannenopvang, Een evaluatiestudie na 2½ jaar ervaring met de opvang en hulpverlening aan mannelijk slachtoffers van (dreiging van) geweld in afhankelijkheidsrelaties in Amsterdam, Rotterdam, Den Haag en Utrecht*, Rijksoverheid, Gemeente Amsterdam, Gemeente Rotterdam, Gemeente Den Haag, Gemeente Utrecht (Uitgevoerd door GGD Rotterdam-Rijnmond), 2011.
- Adrie Vermeulen, Necla Kilic, Bert Visser, Magda Vogelezang, *'Als man heb je al gauw de schijn tegen' Beschrijving expertise Pilot G4 Mannenopvang Huiselijk geweld, Eergerelateerd geweld & Mensenhandel*, 2014.
- Van Dijk, D., Hoekstra, L., & Nieuwenhout, Y. (2010). Als de nood aan de man is: Een verkennende studie naar de opvang van en hulpverlening aan mannelijke slachtoffers van (dreiging van) geweld in afhankelijkheidsrelaties. *Rotterdam: Sociale Zaken en Werkgelegenheid.*
- Janssen, J. H. L. J., & Sanberg, R. (2013). Als de nood aan de man komt. Slachtofferschap van mannen bij eergerelateerd geweld.
- Ligtenberg, D. (2018). Mijn Leven, geslagen man het taboe op mannenmishandeling. Libelle, 33, 74-78.
- Oosten, van, N, Visser, A., Hazebroek, L., Daru, s. (2015). Dossier: wat werkt bij partnergeweld. Utrecht: Movisie.
- Renzetti, C. M., & Miley, C. H. (2014). *Violence in gay and lesbian domestic partnerships*. Routledge.
- Römkens, R. (2010). Omstreden gelijkheid. *B. Den Haag, Huiselijk geweld,* 11-32.
- Schuyf, J. (2009). *Geweld tegen homoseksuele mannen en lesbische vrouwen.* Movisie.
- Tsui, V., Cheung, M., & Leung, P. (2010). Help-seeking among male victims of partner abuse: men's hard times. *Journal of community psychology,* 38(6), 769-780.
- Yanez, P. C. (2018). *A Treatment Model for Male Victims of Domestic Violence: A Support Group for Men with Abusive Partners* (Doctoral dissertation, The Chicago School of Professional Psychology).
- www.mannenmishandeling.nl
- www.huiselijkgeweld.nl
- signalenkaart.nl
