# Supplementary material for: Lesser-known types of violence: Helping nurses and midwives to signal and act
Source: Int J Nurs Stud Adv. 2022 Sep 17;4:100098. doi: 10.1016/j.ijnsa.2022.100098 (PMC11080451; doi:10.1016/j.ijnsa.2022.100098)
Supplement: Supplementary file 1 [file mmc1.zip › Factsheets English/Male abuse.pdf]

MALE ABUSE

ALWAYS USE THE REPORTING CODE WHEN YOU ENCOUNTER A FORM OF (DOMESTIC) VIOLENCE, ABUSE, NEGLECT OR EXPLOITATION! ▶

This fact sheet is part of a series about *(domestic) violence, abuse, neglect, exploitation* and other types of harm that may be inflicted onto someone in a power-imbalanced relationship. Power-imbalanced relationships can exist with anyone, for example: an (ex-)partner, a child, a parent, a sibling, another family member, an informal or a professional carer, a friend, a flatmate or neighbour, a teacher, a colleague or supervisor, or just someone you know. These fact sheets describe different types of harm that can be inflicted in these relationships. They are meant as an add-on to the Dutch Reporting Code for these issues (English version here) and were developed for two reasons: 1) To provide professionals with an overview of all the types of harm that exist, to aid them in identifying both well-known and lesser-known types (see the Overview). 2) Signs/indicators may vary greatly by type of harm and certain types of harm require specific courses of action; the fact sheets help professionals with identifying the signs/indicators and risk factors of *each specific type* of harm and with acting appropriately when they do. Note: the general 5 steps in the Reporting Code are applicable to all types of harm in power-imbalanced relationships; the factsheets provide more guidance within these 5 steps – they are an add-on, not a replacement.

Below is a brief introduction to this topic, an overview of the signs/indicators and risk factors associated with this type of harm, and points of attention for when you encounter it

WHAT IS MALE ABUSE?

Domestic violence against men – commonly referred to as male abuse ("mannenmishandeling") in the Netherlands – concerns any type of (domestic) violence, abuse, neglect, exploitation or other type of harm that may be inflicted onto men as part of a power-imbalanced relationship. This fact sheet focuses on (ex-) partner violence against men. See also the fact sheet on (ex-) partner violence in general. It is estimated that in 40% of the domestic violence cases, there is (also) male abuse. Approximately 80,000 men are severely abused in domestic settings every year.

(Ex-)partner violence aimed at men has many similarities with domestic violence as described in the fact sheet (ex)-partner violence. There are also differences, which must be taken into account in the identification and approach:

- **The perception of men:** Male abuse is not always seen as a problem by the men concerned, nor as a form of domestic violence. Furthermore, these men often feel that there are few, if any, fellow sufferers – that they are the only ones o experience it. Only 3% of men report domestic violence to the police.
- **Social images and norms:** Think of ideas like: "A man does not let himself be beaten (and certainly not by a woman)".

MORE INFORMATION

See the Sources and:

- www.mannenmishandeling.nl
- www.huiselijkgeweld.nl

ADVICE/REPORTING

For advice, for reporting victims or perpetrators, and/or for referring someone to care (including shelters), call:

- Veilig Thuis ("Veilig Thuis" means "Safe at Home" in Dutch, it is the organization in the Netherlands for advice on, referrals to and reporting of any type of (domestic) violence, abuse, neglect or exploitation, or other types of harm in power-imbalanced relationships). Telephone: **0800 20 00**, free of charge and always open (24 hours per day, 7 days a week). It is possible to call anonymously and/or to call for advice or information only, without reporting someone.

In case of acute danger call the emergency services at the phone number **112**.

In the Netherlands there are six shelter locations specifically for men (they have space for 40 men in total).

DUTCH TRANSLATION

See here.

This fact sheet was developed by Federatie Opvang and Stichting LOS in collaboration with various chain partners. september 2020

MALE ABUSE

- **The feeling of shame is very big:** Because of this, men who suffer violence or abuse are less likely to come forward, talk less about it and ask less for help. This shame is also nourished by the aforementioned social images and norms.

ATTENTION FOR THE SPECIFIC TARGET GROUP: LHBTI+

Domestic violence occurs in all relationships, whether heterosexual, homosexual, bisexual or other. Domestic violence under LHBTI+ is also a big taboo. British research shows that one in four homosexuals is a victim of partner violence. People often feel that violence in partner relationships between gays and lesbians is less severe or less serious, because both partners are more or less equal in strength and power ("the other can strike back or defend him-/herself"). Heterosexuals also often assume that homosexuals and lesbians will break off a relationship more quickly than heterosexuals if they are subject to domestic violence. Most heterosexuals do not see economic dependency and raising children together as core concepts of gay and lesbian relationships. Therefore, they assume there are less binding factors for homosexual or lesbian partners. The opposite is true. Research shows that homosexual men endure years of violence in their relationships precisely because they strive for a long-term stable love-relationship.

POSSIBLE SIGNS/INDICATORS: HOW TO IDENTIFY IT

The signs/indicators of male abuse are the same as the signs/indicators described in the fact sheet on (ex)-partner violence and there-mentioned lists of signs/indicators.

RISK FACTORS: WHO IS EXTRA VULNERABLE?

A number of factors can increase the risk of (recurrence of) domestic violence against men:

- **Demographic factors:** age, upbringing, personality, poverty and housing. For example, the risk of abuse is higher if the socio-economic status is lower.
- **Being a witness:** Men who have witnessed or been victims of domestic violence or child abuse in childhood are at increased risk of becoming victims again in adulthood.
- **Dynamic risk factors:** substance use, psychological problems, stress, relationship/family problems. Financial stress is one of the most common reasons for domestic partner violence. Migration stress is also one of the risk factors, as well as when a man comes to the Netherlands from abroad to get married.
- **Static risk factors:** this concerns the man's personal problems, such as a mild intellectual disability.
- **Conditional factors:** for example, social isolation or divorce problems.
- **Societal context:** The extent to which violence towards men is normalised and/or portrayed as less threatening from a social and/or media perspective.
- **Problems with the man's partner:** limited aggression control, addiction, personality problems, difficulty communicating or with conflict management, jealousy and/or a tendency to control.

This fact sheet was developed by Federatie Opvang and Stichting LOS in collaboration with various chain partners.

MALE ABUSE

POINTS OF ATTENTION WHEN GOING THROUGH THE 5 STEPS IN THE REPORTING CODE

For any form of (domestic) violence, abuse, neglect or exploitation, professionals in the Netherlands are required to use the Reporting Code. For general reporting code guidelines (such as the 5 steps in this code) visit the link; these are not described in this fact sheet. We do describe here points of attention in going through the 5 steps that are specific to the topic of this fact sheet. These are:

- **Gender-specific presentation:** In general it can be said that from a gender-specific perspective men have different ways of seeking help, presenting their problems and dealing with problems. There is also another experience of the problem. Men only seek help when they can no longer solve the problem themselves, they seek solutions themselves and try them out, they look for causes of problems outside themselves, they describe problems from an external spectator's point of view, they present problems in the context of work and they articulate problems in cases and facts.
- **Condemnation and societal norms:** Social and health workers too may (unconsciously, unintentionally) condemn or adhere to certain societal norms, which can hinder assistance provided.
- **Taboo:** Because of the taboo around this topic, men will not easily consider themselves to be a victim, for example.
- **Taking control:** Men who have been abused often benefit from retaining as much control as possible over their recovery process. Try to understand how men deal with their problems.

This fact sheet was developed by Federatie Opvang and Stichting LOS in collaboration with various chain partners.
