# Supplementary material for: Lesser-known types of violence: Helping nurses and midwives to signal and act
Source: Int J Nurs Stud Adv. 2022 Sep 17;4:100098. doi: 10.1016/j.ijnsa.2022.100098 (PMC11080451; doi:10.1016/j.ijnsa.2022.100098)
Supplement: Supplementary file 1 [file mmc1.zip › Factsheets English/Online sexual intimidation - sources.pdf]

SOURCES SEXUAL BEHAVIOUR BETWEEN YOUNG PEOPLE THAT CROSSES BOUNDARIES

NOTE

For all forms of domestic violence and child abuse, the Dutch Reporting Code (English version here) for these issues must be applied in the Netherlands by all groups of professionals named in the Reporting Code law. Sexual behaviour between young people that crosses boundaries does not fall under the definition of domestic violence or child abuse in the Netherlands and therefore, it is not legally required to use the reporting code when you encounter it as a professional. However, the reporting code may be used. Because we feel it is a useful guideline for professionals for this type of harm as well, and because it is important that professionals (e.g. general practitioners) can identify this type of harm and take the right steps, this factsheet was compiled.

ORGANISATIONS INVOLVED

The following organisations were involved in making this fact sheet:

- Movisie. For questions and/or remarks about the fact sheet, please email the main author: Wilma Schakenraad, w.schakenraad@movisie.nl
- Veilig Thuis
- Kennisinstituut voor Emancipatie en Vrouwengeschiedenis (Atria)

SOURCES

The following documents and other sources provide more information about the topic of this fact sheet:

Publications

- Berlo van, W. & Beek I. van (2015). Whitepaper Seksuele grensoverschrijding en seksueel geweld. Feiten en cijfers Utrecht: Rutgers en Movisie.
- Graaf, H. de, Borne, M. van den, Nikkelen, S., Twisk, D., & Meijer, S. (2017). Seks onder je 25e. Utrecht / Amsterdam: Rutgers / Soa Aids Nederland.
- Haas, S. de (2012). Seksueel grensoverschrijdend gedrag onder jongeren en volwassenen in Nederland. Tijdschrift voor Seksuologie, 36-2, p. 136-145.
- Höing, M., & Janssen, J. (2017). Seksueel grensoverschrijdend gedrag. In: Höing, M., & Janssen, J., Boer, A., & Liebregts, M. (red.). Bespreekbaar maken van seksualiteit en intimiteit. Handboek voor professionals in zorg en welzijn. Bussum: Coutinho.
- Nationaal Rapporteur Mensenhandel en Seksueel Geweld tegen Kinderen (2014). Op goede grond. De aanpak van seksueel geweld tegen kinderen. Den Haag: Nationaal Rapporteur.
- Römkens, R. (2017). Factsheet Online seksuele intimidatie. Amsterdam: Atria. Zie: www.atria.nl/sites/atria/files/atoms/files/factsheet-cybergeweld-onlineversiedef.pdf
- Storms, O. & Doornink, N. (2016). Vlaggensysteem: Reageren op seksueel (grensoverschrijdend) gedrag van kinderen en jongeren. Effectieve sociale interventies en Effectieve interventies huiselijk en seksueel geweld. Utrecht: Movisie.

Websites

- seksonderje25e.nl (Rutgers)
- Kennisdossier seksuele grensoverschrijding van Rutgers: www.rutgers.nl/feiten-en-cijfers/kennisdossiers/kennisdossier-seksuele-grensoverschrijding
- www.seksueelgeweld.info Website for victims of sexual violence, and for those involved and referrers. See also the social map with an overview of the available help for victims and perpetrators of sexual violence.
- www.vlaggensysteem.nl
- www.act4respect.nl
- www.atria.nl
