# Supplementary material for: Lesser-known types of violence: Helping nurses and midwives to signal and act
Source: Int J Nurs Stud Adv. 2022 Sep 17;4:100098. doi: 10.1016/j.ijnsa.2022.100098 (PMC11080451; doi:10.1016/j.ijnsa.2022.100098)
Supplement: Supplementary file 1 [file mmc1.zip › Factsheets English/Online sexual intimidation.pdf]

ONLINE SEXUAL INTIMIDATION

This fact sheet is part of a series about *(domestic) violence, abuse, neglect, exploitation* and other types of harm that may be inflicted onto someone in a power-imbalanced relationship. Power-imbalanced relationships can exist with anyone, for example: an (ex-)partner, a child, a parent, a sibling, another family member, an informal or a professional carer, a friend, a flatmate or neighbour, a teacher, a colleague or supervisor, or just someone you know. These fact sheets describe different types of harm that can be inflicted in these relationships. They are meant as an add-on to the Dutch Reporting Code for these issues (English version here) and were developed for two reasons: 1) To provide professionals with an overview of all the types of harm that exist, to aid them in identifying both well-known and lesser-known types (see the Overview). 2) Signs/indicators may vary greatly by type of harm and certain types of harm require specific courses of action; the fact sheets help professionals with identifying the signs/indicators and risk factors of *each specific type* of harm and with acting appropriately when they do. Note: the general 5 steps in the Reporting Code are applicable to all types of harm in power-imbalanced relationships; the factsheets provide more guidance within these 5 steps – they are an add-on, not a replacement.

Below is a brief introduction to this topic, an overview of the signs/indicators and risk factors associated with this type of harm, and points of attention for when you encounter it.

ALWAYS USE THE REPORTING CODE WHEN YOU ENCOUNTER A FORM OF (DOMESTIC) VIOLENCE, ABUSE, NEGLECT OR EXPLOITATION! ▶

WHAT IS ONLINE SEXUAL HARASSMENT?

The European Union Agency for Fundamental Rights (FRA) defines online sexual harassment as: receiving unwanted, offensive, sexually oriented e-mails or text messages or experiencing inappropriate sexual advances through social media or online chat rooms.[1] There is a continuum of behaviours that differ in nature and seriousness, with opinions varying about what is or is not socially or legally permissible.[2]

Forms of online sexual harassment are:

- Shame-sexting:[3] images of a sexual nature (often created by the person depicted) are shared with third parties via social media without the person's consent.[4]
- Grooming: the active approach and seduction of minors by adults via the Internet and social networking sites, chat rooms or webcams for the purpose of developing sexual contacts.
- Revenge pornography: shame-sexting with revenge as motive, often after a relationship ended.
- Sextortion: images of a sexual nature (often obtained under false pretences) are used to blackmail the person depicted
- Online distribution of sexual violence: recordings or images of forced sexual acts are distributed online to a wider audience
- Unsolicited sending or posting of messages of a sexual nature: this may involve gossip or bullying of a sexual nature.

FACTS AND FIGURES

Various studies carried out in the Netherlands show that:

- 14% of girls and 6% of boys aged 12-25 years had at least one experience with sexting that they found annoying[9].
- girls have received unwanted sexual advances on the internet about three times more often than boys [10]
- girls find sexually-tinged communication more annoying than boys[11]

This fact sheet was developed by Atria, Kennisinstituut voor Emancipatie en Vrouwengeschiedenis in collaboration with various chain partners.

ONLINE SEXUAL INTIMIDATION

POSSIBLE SIGNS/INDICATORS: HOW TO IDENTIFY IT

Victims regularly experience feelings of shame, so they often do not report an incident. Fear of how loved ones will respond makes identification difficult. Victims often show psychological problems such as anxiety and stress,[5] they withdraw and avoid social activities.[6] In addition, victims often experience a decrease in productivity at school/work and young people may be absent from school frequently.[7]

RISK FACTORS: WHO IS EXTRA VULNERABLE?

Young people and girls are at increased risk of sexual harassment online. International studies indicate that girls and women are targeted up to three times more often for sexual and unwanted advances than boys and men. In addition, various studies have shown that young people who run the risk of unwanted sexual advances 'offline' will also be more likely to be affected online.[8]

POINTS OF ATTENTION WHEN GOING THROUGH THE 5 STEPS IN THE REPORTING CODE

For any form of (domestic) violence, abuse, neglect or exploitation, professionals in the Netherlands are required to use the Reporting Code. For general reporting code guidelines (such as the 5 steps in this code) visit the link; these are not described in this fact sheet. We do describe here points of attention in going through the 5 steps that are specific to the topic of this fact sheet. These are:

- Preferably speak to someone alone
- Victims often do not know where to turn to: therefore, explain where they can get help.
- Victims may have to overcome shame and guilt.
- Victims are often unaware of their rights: explain these clearly
- For minors: discuss how parents can support. Follow the professional code when considering whether it can be kept secret from parents.
- For advice you can always contact Veilig Thuis.
- In some cases online sexual harassment is covered by the reporting code domestic violence and child abuse and in some cases not: contact Veilig Thuis for advice.

ADVICE/REPORTING

For advice, for reporting victims or perpetrators, and/or for referring someone to care (including shelters), call:

- Veilig Thuis ("Veilig Thuis" means "Safe at Home" in Dutch, it is the organization in the Netherlands for advice on, referrals to and reporting of any type of (domestic) violence, abuse, neglect or exploitation, or other types of harm in power-imbalanced relationships). Telephone: **0800 20 00**, free of charge and always open (24 hours per day, 7 days a week). It is possible to call anonymously and/or to call for advice or information only, without reporting someone.
- or see the websites under "More information"

In case of acute danger call the emergency services at the phone number **112**.

MORE INFORMATION

See the Sources, the online sexual harassment fact sheet by Atria and the following websites:

- Atria
- Centrum Seksueel Geweld
- Meldknop
- Politie

DUTCH VERSION

See here.

This fact sheet was developed by Atria, Kennisinstituut voor Emancipatie en Vrouwengeschiedenis in collaboration with various chain partners.

september 2020
