# Supplementary material for: Lesser-known types of violence: Helping nurses and midwives to signal and act
Source: Int J Nurs Stud Adv. 2022 Sep 17;4:100098. doi: 10.1016/j.ijnsa.2022.100098 (PMC11080451; doi:10.1016/j.ijnsa.2022.100098)
Supplement: Supplementary file 1 [file mmc1.zip › Factsheets English/Parent abuse - sources.pdf]

SOURCES PARENT ABUSE

ORGANISATIONS INVOLVED

The following organisations were involved in making this fact sheet:

- TNO (For questions and/or remarks about the fact sheet, please email the main author Remy Vink, remy.vink@tno.nl)
- MOVISIE
- GGD GHOR Nederland
- Augeo Foundation
- Veilig Thuis
- VVAK
- Het Lorentzhuis

SOURCES

The following documents and other sources provide more information about the topic of this fact sheet:

- Beckmann, L., Bergmann, M. C., Fischer, F. and Mosel, T. (2017) Risk and Protective Factors of Child-to-Parent Violence: A Comparison Between Physical and Verbal Aggression, Journal of Interpersonal Violence, first published online 12th December 2017
- Biehal, N. (2012). Parent abuse by young people on the edge of care: a child welfare perspective. Social policy & society, 11(2),251-263.
- Calvete E, Orue I, Gamez-Guadix M, Bushman BJ. (2015) Predictors of child-to-parent aggression: A 3-year longitudinal study. Dev Psychol. 2015 May;51(5):663-76. doi: 10.1037/a0039092. Epub 2015 Mar 30.
- Cottrell, B. & Monk, P. (2004). Adolescent-to-parent abuse: A qualitative overview of common themes. J.Fam. Issues, 25(8), 1072-1095.
- Eckstein, N.J. (2004). Emergent issues in families experiencing adolescent-to-parent abuse. Western Journal of Communication, 68(4), 365-388.
- Holt, A. (2013). Adolescent-to-Parent Abuse: Current Understandings in Research, Policy and Practice. Policy Press, Bristol University.
- Holt, A. (2015) Working with Adolescent Violence and Abuse Towards Parents: Approaches and Contexts for Intervention. London. Routledge.
- Ibabe, I. & Jaureguizar, J. (2010). Child-to-parent violence: Profile of abusive adolescents and their families. Journal of Criminal Justice, 38(4), 616-624.
- Kennair, N. & Mellor, D. (2007). Parent abuse: a review. Child Psychiatry Hum. Dev., 38(3), 203-219.
- Weinblatt, U. & Omer, H. (2008). Geweldloos verzet: een behandeling voor ouders van kinderen met ernstige gedragsproblemen. Gezinstherapie Wereldwijd, 19(4): 389-418.
- Pagani, L., Larocque, D., Vitaro, F., & Tremblay, R.E. (2003). Verbal and Physical Abuse Toward Mothers: The Role of Family Configuration, Environment, and Coping Strategies. Journal of Youth and Adolescence,32(3), 215-222.
- Routt, G., & Anderson L. (2011). Adolescent violence towards parents. Journal of Aggression, Maltreatment and Trauma, 20(1), 1-18.
- Ulman, A., & Straus, M.A. (2003). Violence by children against mothers in relation to violence between parents and corporal punishment by parents. J of Comparative Family Studies 34(1), 41-60.
- Vink, R.M., Goes, A., Doornink, N., Broerse, A., Pannebakker, F., Zwan, van der R., Schakenraad, W. (2014) Huiselijk geweld door kinderen en jongeren tegen hun ouders. Utrecht/Leiden. Movisie/TNO

This fact sheet was developed by TNO in collaboration with various chain partners. september 2020
