# Supplementary material for: Lesser-known types of violence: Helping nurses and midwives to signal and act
Source: Int J Nurs Stud Adv. 2022 Sep 17;4:100098. doi: 10.1016/j.ijnsa.2022.100098 (PMC11080451; doi:10.1016/j.ijnsa.2022.100098)
Supplement: Supplementary file 1 [file mmc1.zip › Factsheets English/Parent abuse.pdf]

PARENT ABUSE

This fact sheet is part of a series about *(domestic) violence, abuse, neglect, exploitation* and other types of harm that may be inflicted onto someone in a power-imbalanced relationship. Power-imbalanced relationships can exist with anyone, for example: an (ex-)partner, a child, a parent, a sibling, another family member, an informal or a professional carer, a friend, a flatmate or neighbour, a teacher, a colleague or supervisor, or just someone you know. These fact sheets describe different types of harm that can be inflicted in these relationships. They are meant as an add-on to the Dutch Reporting Code for these issues (English version here) and were developed for two reasons: 1) To provide professionals with an overview of all the types of harm that exist, to aid them in identifying both well-known and lesser-known types (see the Overview). 2) Signs/indicators may vary greatly by type of harm and certain types of harm require specific courses of action; the fact sheets help professionals with identifying the signs/indicators and risk factors of *each specific type* of harm and with acting appropriately when they do. Note: the general 5 steps in the Reporting Code are applicable to all types of harm in power-imbalanced relationships; the factsheets provide more guidance within these 5 steps – they are an add-on, not a replacement.

Below is a brief introduction to this topic, an overview of the signs/indicators and risk factors associated with this type of harm, and points of attention for when you encounter it.

ALWAYS USE THE REPORTING CODE WHEN YOU ENCOUNTER A FORM OF (DOMESTIC) VIOLENCE, ABUSE, NEGLECT OR EXPLOITATION! ▶

WHAT IS PARENT ABUSE?

Parent abuse (or "parental abuse" or "child-to-parent abuse/violence", but not to be confused with elder abuse) is: non-incidental violence in the family which is aimed at (one of) the parents, committed by a youth or young adult, who usually lives at home and is dependent on the parent. Parent abuse concerns repeated and serious violence (not the 'normal' puberty conflicts). Violence can be psychological, physical and sexual, with financial extortion and material damage.

POSSIBLE SIGNS/INDICATORS: HOW TO IDENTIFY IT

There is a lot of shame with parents (feelings of failure). Parents usually only ask for help at an advanced stage. Possible signs:

- Parents are afraid of their own child.
- Increase in verbal violence (often around 12-14 years) and escalation to serious threat and physical violence.
- Arson, (traces of) material damage.
- Severe outbursts of anger due to triggers (e.g. alcohol, drugs, internet and gaming addiction).
- Reversal of day-night rhythm and hardly any daytime activities.
- (Serious) behavioural problems, aggression and anger in connection with psychopathology.

FACTS AND FIGURES

- It is estimated that 10% of all incidents of family violence with police involvement concerns parents as victims.
- More than 80% of the perpetrators are boys/young men.
- In two thirds of cases, the biological mother is the victim.

ADVICE/REPORTING

For advice, for reporting victims or perpetrators, and/or for referring someone to care (including shelters), call:

- Veilig Thuis ("Veilig Thuis" means "Safe at Home" in Dutch, it is the organization in the Netherlands for advice on, referrals to and reporting of any type of (domestic) violence, abuse, neglect or exploitation, or other types of harm in power-imbalanced relationships). Telephone: **0800 20 00**, free of charge and always open (24 hours per day, 7 days a week). It is possible to call anonymously and/or to call for advice or information only, without reporting someone.

In case of acute danger call the emergency services at the phone number **112**.

DUTCH TRANSLATION

See here.

This fact sheet was developed by TNO in collaboration with various chain partners. september 2020

- Parent(s) feel the behavioural change of their child had a sudden onset ("he/she was not like this in the past").
- Co-occurrence of family violence such as sibling abuse.
- Violence also outside the family (e.g. dating violence).
- Negative communication and interaction patterns in the family.
- Male dominance as a social norm within the family.
- Problematic behaviour with peers, truancy and long term absenteeism, suspensions, bullying or being bullied.
- Debts, financial exploitation and extortion of parents.
- Restraining orders, report(s) to the police by parents or family.
- Parents isolate themselves and feelings of exhaustion.

RISK FACTORS: WHO IS EXTRA VULNERABLE?

Little is known about risk factors for parent abuse. The general picture from both practice and scientific literature is that parent abuse is mostly committed by boys, from 14/15 years of age, living at home. Violence is mostly aimed at single (divorced) mothers of all socio-economic backgrounds. Young people with psychiatric problems, especially when this only manifests itself in puberty, with behavioural problems, low frustration tolerance, low empathy and adaptability, seem to form a specific group of perpetrators.

A disrupted parent-child interaction is also a risk factor, for example because of: a very permissive upbringing without boundaries, lack of family cohesion or extreme protection, shift in authority relationships within the family (e.g. due to divorce), family violence, unpredictability of parents.

POINTS OF ATTENTION WHEN GOING THROUGH THE 5 STEPS IN THE REPORTING CODE

For any form of (domestic) violence, abuse, neglect or exploitation, professionals in the Netherlands are required to use the Reporting Code. For general reporting code guidelines (such as the 5 steps in this code) visit the link; these are not described in this fact sheet. We do describe here points of attention that are specific to the topic of this fact sheet. These are:

- Ask parents how they are doing and how they feel, listen, ask for details and take them seriously.
- When treating a young person with a mental health problem, check whether there is aggression and remember that parent abuse is a potential consequence of that situation.
- Also pay attention to the other children in the family (as victims or perpetrators).
- In order to stop serious violence it may be necessary to call in the police (resulting in an official report or a restraining order).
- Consult Veilig Thuis about the possibility of out-of-home placement of the young person.
- Consider the following programmes: parenting support, MST, Relational Family Therapy, MDFT, Non-Violent Resistance, or an Aggression Regulation programme.

MORE INFORMATION

See the Sources and:

- The website Holes in the wall
- The database of Effective interventions for family violence and sexual violence
- This report by TNO and Movisie about parent abuse

This fact sheet was developed by TNO in collaboration with various chain partners.
