# Supplementary material for: Lesser-known types of violence: Helping nurses and midwives to signal and act
Source: Int J Nurs Stud Adv. 2022 Sep 17;4:100098. doi: 10.1016/j.ijnsa.2022.100098 (PMC11080451; doi:10.1016/j.ijnsa.2022.100098)
Supplement: Supplementary file 1 [file mmc1.zip › Factsheets English/Protection of the unborn child - sources.pdf]

SOURCES VULNERABLE PREGNANT WOMEN AND PROTECTION OF THE UNBORN CHILD

ORGANISATIONS INVOLVED

The following organisations were involved in making this fact sheet:

- Veilig Thuis, Anne-Marie Raat
- TNO, Remy Vink, remy.vink@tno.nl
- Erasmus MC / Dutch Association for Obstetrics and Gynaecology (NVOG), Hans J. Duvekot

SOURCES

The following documents and other sources provide more information about the topic of this fact sheet:

websites

- Veilig Thuis
- www.voorzorg.info
- www.tienermoeders.nl
- www.nunietzwanger.nl
- www.RSJ.nl (advies prenatale kinderbescherming en de rol van de overheid, juni 2015)
- www.kinderbescherming.nl (Informatieblad Bescherming nog niet geboren kinderen en voorkomen kwetsbare zwangerschappen. Raad voor de Kinderbescherming, januari 2017)
- www.TNO.nl (De eerste duizend dagen)
- www.NVAVG.nl (Handreiking kinderwens en anticonceptie bij mensen met een verstandelijke beperking. Oktober 2016)
- www.fiom.nl en www.siriz.nl (hulp bij onbedoelde zwangerschap)
- www.lkpz.nl (Kenniscentrum Psychiatrie en zwangerschap)
- www.trimbos.nl
- ALPHA-NL
- R4U
- Mind2Care
- TNO Checklist Vroegsignalering
- Verloskundig SamenwerkingsVerband
- (Pre)SPARK
- GIZ
- SamenStarten
- Kindcheck
- Nji: figures about teenage mothers
- TNO about smoking
- TNO on alcohol consumption

publications

- Ahmadabadi, Z. e.a. (2018) Maternal intimate partner violence victimization and child maltreatment. Child Abuse & Neglect, 23-33
- Austin, A. e.a. (2018) Using time-to-event analysis to identify preconception and prenatal predictors of child protective services contact. Child Abuse & Neglect, 83-91
- Chen, H. e.a. (2018) Prenatal smoking and post partum depression: a meta- analysis. Journal of Psychosomatic Obstetrics & Gynecology, , 1-9
- Guterman, K., (2015) Unintended pregnancy as a predictor of child maltreatment. Child Abuse & Neglect, 160-169
- Hafekost, K. e.a. (2017) Maternal alcohol use disorder and subsequent child protection contact: A record-linkage population cohort study. Child Abuse & Neglect, 206-214
- Huizink, A.C. (2013) Prenatal cannabis exposure and infant outcomes: Overview of studies. Prog Neuro-Psychopharmacol Biol Psychiatry 2013
- Lambregtse-Van den Berg, M., Kamp, I. van, Wennink, H. (red.) Handboek psychiatrie en zwangerschap. ISBN 9789058982698. Utrecht. Uitgeverij De Tijdstroom (2015)
- Mejdoubi, J., Heijkant, S.C.C.M., van den, Struijf, E., Leerdam, J.M., van, HiraSing, R.A., Crijnen, A.M. (2013) Risicofactoren voor kindermishandeling bij jonge hoogrisicozwangeren: design van het VoorZorg onderzoek. Tijdschrift voor Jeugdgezondheidszorg, pp 26–31
- Prindle, P. e.a Prenatal substance exposure diagnosed at birth and infant involvement with child protective services. Child Abuse & Neglect 2018, 75-83
- Steegers, E. (2017) Sociale verloskunde: gelijke kansen op een gezonde start. Ned Tijdschr Geneesk;161: 1-4
- Taylor, J. & A. Lazenbatt (2014) Child Maltreatment and High Risk Families, Dunedin Academic Press, Edinburgh. ISBN 978-1-78046-031-4
- VWS, Actieprogramma Kansrijke Start, september 2018. Den Haag. Ministerie van Volksgezondheid, Welzijn en Sport
- Wall-Wieler, E. e.a. (2018) Predictors of having a first child taken into care at birth: a population based retrospective cohort study. Child Abuse & Neglect, 1-9
- Wewerinke, A., Honig, A., Heres, M.H.B., en Wennink, J.M.B. Psychiatrische stoornissen bij zwangeren en kraamvrouwen. Ned Tijdschr Geneeskd. 2006;150:294-8.

This fact sheet was developed by Nederlandse Vereniging voor Obstetrie en Gynaecologie (NVOG), Erasmus MC, TNO and Veilig Thuis in collaboration with various chain partners.

december 2018
