# Supplementary material for: Lesser-known types of violence: Helping nurses and midwives to signal and act
Source: Int J Nurs Stud Adv. 2022 Sep 17;4:100098. doi: 10.1016/j.ijnsa.2022.100098 (PMC11080451; doi:10.1016/j.ijnsa.2022.100098)
Supplement: Supplementary file 1 [file mmc1.zip › Factsheets English/Protection of the unborn child.pdf]

VULNERABLE PREGNANT WOMEN AND PROTECTION OF THE UNBORN CHILD

This fact sheet is part of a series about *(domestic) violence, abuse, neglect, exploitation* and other types of harm that may be inflicted onto someone in a power-imbalanced relationship. Power-imbalanced relationships can exist with anyone, for example: an (ex-)partner, a child, a parent, a sibling, another family member, an informal or a professional carer, a friend, a flatmate or neighbour, a teacher, a colleague or supervisor, or just someone you know. These fact sheets describe different types of harm that can be inflicted in these relationships. They are meant as an add-on to the Dutch Reporting Code for these issues (English version here) and were developed for two reasons: 1) To provide professionals with an overview of all the types of harm that exist, to aid them in identifying both well-known and lesser-known types (see the Overview). 2) Signs/indicators may vary greatly by type of harm and certain types of harm require specific courses of action; the fact sheets help professionals with identifying the signs/indicators and risk factors of *each specific type* of harm and with acting appropriately when they do. Note: the general 5 steps in the Reporting Code are applicable to all types of harm in power-imbalanced relationships; the factsheets provide more guidance within these 5 steps – they are an add-on, not a replacement.

Below is a brief introduction to the topic of vulnerable pregnant women and protection of the unborn child, an overview of the signs/indicators and risk factors associated with this type of harm, and points of attention for when you encounter it.

WHO ARE VULNERABLE PREGNANT WOMEN?

Vulnerable pregnant women have an increased risk of harm to themselves and to the unborn child, risk of poor pregnancy outcomes, and of child abuse and neglect. A bad start as a baby may affect people for the rest of their lives.

Vulnerabilities can often already be observed during the pregnancy and concern both the behaviour and lifestyle of the pregnant woman and that of the (ex-)partner or spouse.

Factors of vulnerability are for example: intimate partner violence, psychiatric diagnose or mental health issues, intellectual disability, substance abuse and substance use by the mother, young age (teenage pregnancies), or experiencing problems in several areas of life ('multi-problem' families; see Risk Factors below for a more detailed overview).

RISK FACTORS: WHO IS EXTRA VULNERABLE?

- Intimate partner violence prior to and/or during pregnancy.
- Experiences of expectant parent(s) with child abuse and neglect (including sexual abuse) in their own youth.
- Other children in the family are already under child protection or in foster care.
- Psychiatric problems, mental health issues, or (mild) intellectual disability in expectant parents.
- Depression and prior postnatal depression.
- Smoking, alcohol and drug use during pregnancy.
- Alcohol and drug abuse and other addiction in the partner / spouse.

FACTS AND FIGURES

- **Of all pregnant women in the Netherlands,** 8.9% sometimes **drink** alcohol and 0.8% sometimes **drink** more than four glasses in one day.
- Of all pregnant lower educated women, **22.1%** smoke daily throughout their pregnancy, **5.5%** of medium educated women and 0,9% high educated women do so.
- 12% of Dutch pregnant women experience **depressive disorder** during or after pregnancy
- Prevalence of teenage pregnancies including birth, in the Netherlands is approximately **1500 teenagers** (< 20 years) a year (0.9% of the total).

ADVICE/REPORTING

For advice, for reporting victims or perpetrators, and/or for referring someone to care (including shelters), call:

- Veilig Thuis ("Veilig Thuis" means "Safe at Home" in Dutch, it is the organization in the Netherlands for advice on, referrals to and reporting of any type of (domestic) violence, abuse, neglect or exploitation, or other types of harm in power-imbalanced relationships). Telephone: **0800 20 00**, free of charge and always open (24 hours per day, 7 days a week).

This fact sheet was developed by Nederlandse Vereniging voor Obstetrie en Gynaecologie (NVOG), Erasmus MC, TNO and Veilig Thuis in collaboration with various chain partners.

VULNERABLE PREGNANT WOMEN AND PROTECTION OF THE UNBORN CHILD

- Teenage pregnancies, unintentional pregnancies, low educational level and single parent (especially in co-occurrence: a group that is seen often in the prevention programme Voorzorg).
- Babies who are born prematurely or with a low birth weight are statistically more likely to be born into vulnerable families.
- Negative attributions and hostility towards the unborn child and/or of previous children.
- Late onset in maternity health care, non-compliance with therapy and advice, frequent cancellations of appointments or not appearing, insufficient self-care.
- Problems and stress in multiple areas of life: housing, financial problems and debts, unemployment, no insurance, crime and delinquency, family violence, no or non-helpful social support.

GUIDING DOCUMENTS FOR VARIOUS GROUPS OF PROFESSIONALS IN IDENTIFICATION/SIGNALLING

- **Midwifery/obstetricians:** interview and ALPHA-NL, R4U or Mind2Care.
- **Maternity carers:** use the TNO Early Warning Checklist during the maternity period.
- **Youth public health care:** screen during regular contact moments and/or based on (Pre)SPARK, GIZ, SamenStarten.
- **Everyone:** use the 'Child Check', discuss your concerns with the expectant parents, discuss your concerns in multidisciplinary consultations, consult with Veilig Thuis.

REFERRAL DURING PREGNANCY

Low threshold:

- Prenatal Home visits by a Youth Public Health Care nurse or by the 'Stevig Ouderschap' programme (prenatally)
- Social neighbourhood team (practical help in all areas of life, light parenting support, access to specialist help)
- Relationship therapy (via GP or social neighbourhood team)
- Other agreements made in the Maternity Health Care Collaboration (VSV)

Specific:

- The 'Voorzorg' programme
- (Youth) mental healthcare, addiction care, perpetrator programmes
- POP-clinic (Psychiatry, Obstetrics and Paediatrics): maternity health care in relation to psychiatry
- Forms of home visitation, various programmes for teenage mothers, parents with (mild) intellectual disabilities, parents with psychiatric problems.

POINTS OF ATTENTION WHEN GOING THROUGH THE 5 STEPS IN THE REPORTING CODE

For any form of (domestic) violence, abuse, neglect or exploitation, professionals in the Netherlands are required to use the Reporting Code. For general reporting code guidelines (such as the 5 steps in this code) visit the link; these are not described in this fact sheet. We do describe here points of attention in going through the 5 steps that are specific to the topic of this fact sheet.

- It is possible to call anonymously and/or to call for advice or information only, without reporting someone.

In case of acute danger call the emergency services at the phone number **112**.

DUTCH TRANSLATION

See here.

This fact sheet was developed by Nederlandse Vereniging voor Obstetrie en Gynaecologie (NVOG), Erasmus MC, TNO and Veilig Thuis in collaboration with various chain partners.

VULNERABLE PREGNANT WOMEN AND PROTECTION OF THE UNBORN CHILD

These are:

- Unborn children can be put under supervision by a social worker throughout pregnancy. Child protection can then give instructions such as assisting with obstetric check-ups and other forms of care.
- In the case of suicidality and addiction to hard drugs, forced admission to a care facility is possible on the basis of the BOPZ Act. In the event of incapacity in making decisions, mentorship or guardianship can be requested from the court.
- You can always ask advice at Veilig Thuis, anonymously if you wish , ask for the resident doctor. Acute and structural threat to the unborn child and not cooperating with care, are reasons to file a report to Veilig Thuis.

MORE INFORMATION

See the Sources and:

- www.fiom.nl
- www.tienermoeders.nl

This fact sheet was developed by Nederlandse Vereniging voor Obstetrie en Gynaecologie (NVOG), Erasmus MC, TNO and Veilig Thuis in collaboration with various chain partners.
