# Supplementary material for: Lesser-known types of violence: Helping nurses and midwives to signal and act
Source: Int J Nurs Stud Adv. 2022 Sep 17;4:100098. doi: 10.1016/j.ijnsa.2022.100098 (PMC11080451; doi:10.1016/j.ijnsa.2022.100098)
Supplement: Supplementary file 1 [file mmc1.zip › Factsheets English/Radicalisation - sources.pdf]

SOURCES RADICALISATION

ORGANISATIONS INVOLVED

The following organisations were involved in making this fact sheet:

- The Landelijk Steunpunt Extremisme (LSE) | National Support Centre for Extremism (LSE), also a member of the JeP Platform, was in charge of developing this fact sheet. For questions and/or remarks about the fact sheet, please email: info@hetlse.nl
- Stichting School en Veiligheid
- Veilig Thuis

SOURCES

The following documents and other sources provide more information about the topic of this fact sheet:

- An ideology is a package of ideas on worldviews and existential meaning which focuses on perceptions on being human and the organization of society, such as fascism, anarchism, nationalism, (neo)liberalism, socialism, Islamism, Christian democracy, and conservatism. An ideology is therefore always politically oriented, but is not always an antiliberal or violent movement.
- "Terrorism is the ideologically motivated act of violence against humans or social disruptive damage to public property with the aim of undermining and destabilising society, seriously terrorizing the population or influencing political decision-making. CT Strategy, NCTV, www.nctv.nl/binaries/CT-strategie%202016-2020_tcm31-80007.pdf.
- For an overview see: PlatformJEP, www.platformjep.nl/documenten/vragen-en-antwoorden/wat-zijn-de-definities-van-radicalisering-extremisme-en-polarisatie. NCTV: www.nctv.nl/organisatie/ct/terrorismebestrijding/extremisme/extremisme.aspx.
- For examples of radicalisation processes, see: Understanding Radicalisation: Review of Literature, Dzhekova et al., Center for the Study of Democracy, 2016, www.csd.bg/artShow.php?id=17560 . Trigger factors in the radicalisation process, Feddes et al. Expertise unit Social Stability and University of Amsterdam, 2015, www.socialestabiliteit.nl/professionals/documenten/publicaties/2015/10/13/triggerfactoren-in-het-radicaliseringsproces . Genesis of radicalisation, Wienke and Ramadan, NJI, 2011, www.nji.nl/nl/Producten-en-diensten/Publicaties/NJi-Publicaties/Polarisatie-en-radicalisering-bij-jongeren.html .
- For an overview of signals and factors, see: Trigger factors Radicalisation, www.socialestabiliteit.nl/professionals/triggerfactoren . And Recognition and interpretation, Wienke and Ramadan, NJI, 2011, www.nji.nl/nl/Download-NJi/Publicatie-NJi/Pol_Rad_Herkenning_duiding.pdf.
- Conversion to a new religion or other ideological identity can be experienced as polarising, but is not in itself a sign of radicalisation.
- For a discussion on this, see: NJI, www.nji.nl/nl/Kennis/Dossier/Radicalisering/Achtergrond/Ontwikkeling , and, www.nji.nl/nl/Download-NJi/Publicatie-NJi/Pol_Rad_Ontstaan_radicalisering.pdf .
- For examples, see: NJI, www.nji.nl/nl/Kennis/Dossier/De-rol-van-jeugdhulp-bij-het-tegengaan-van-radicalisering-van-jongeren.
- These figures mainly concern foreign fighters who joined Jihadist groups in Syria and Iraq as of 2013. Of the several hundred people who travelled to Syria and Iraq, a few dozen have now returned. These returnees may include people who, through their experience, have become disillusioned with the extremist ideology and network, and others who are still active. This fact sheet focuses primarily on identifying radicalisation among persons who have not yet committed any criminal offences, and in which radicalisation is considered in its full breadth and not only from the perspective of religious extremism such as Jihadism.

This fact sheet was developed by Landelijk Steunpunt Extremisme (LSE) in collaboration with various chain partners. september 2020
