# Supplementary material for: Lesser-known types of violence: Helping nurses and midwives to signal and act
Source: Int J Nurs Stud Adv. 2022 Sep 17;4:100098. doi: 10.1016/j.ijnsa.2022.100098 (PMC11080451; doi:10.1016/j.ijnsa.2022.100098)
Supplement: Supplementary file 1 [file mmc1.zip › Factsheets English/Radicalisation.pdf]

RADICALISATION

ALWAYS USE THE REPORTING CODE WHEN YOU ENCOUNTER A FORM OF (DOMESTIC) VIOLENCE, ABUSE, NEGLECT OR EXPLOITATION! ▶

This fact sheet is part of a series about *(domestic) violence, abuse, neglect, exploitation* and other types of harm that may be inflicted onto someone in a power-imbalanced relationship. Power-imbalanced relationships can exist with anyone, for example: an (ex-)partner, a child, a parent, a sibling, another family member, an informal or a professional carer, a friend, a flatmate or neighbour, a teacher, a colleague or supervisor, or just someone you know. These fact sheets describe different types of harm that can be inflicted in these relationships. They are meant as an add-on to the Dutch Reporting Code for these issues (English version here) and were developed for two reasons: 1) To provide professionals with an overview of all the types of harm that exist, to aid them in identifying both well-known and lesser-known types (see the Overview). 2) Signs/indicators may vary greatly by type of harm and certain types of harm require specific courses of action; the fact sheets help professionals with identifying the signs/indicators and risk factors of *each specific type* of harm and with acting appropriately when they do. Note: the general 5 steps in the Reporting Code are applicable to all types of harm in power-imbalanced relationships; the factsheets provide more guidance within these 5 steps – they are an add-on, not a replacement.

Below is a brief introduction to this topic, an overview of the signs/indicators and risk factors associated with this type of harm, and points of attention for when you encounter it.

WHAT IS RADICALISATION?

Since the mid-20th century, radicalisation and extremism have been studied extensively in relation to various forms of violent extremism. A central insight that has emerged from this research is that radicalisation is a difficult phenomenon to describe in a definite fashion. Violent forms may arise from any political or religious ideology. Some organise themselves into terrorist or paramilitary groups, others into small activist groups, and yet others become dangerous loners. In addition, not every form of radicalisation expresses itself through violence. Civil rights activists such as Martin Luther King were also considered radical. Certain ecological or religious lifestyles can be seen as radical or polarising, but are not necessarily illegal. What is considered radical is therefore very subjective. This fact sheet is limited to radicalisation that develops towards a form of (violent) extremism. It presents a general discussion of four main forms of extremism: religious extremism, left-wing extremism, right-wing extremism, and environmental extremism. Some examples of contemporary definitions of radicalisation and extremism are:

> "Radicalisation is an erratic process. A young person who is taken by radical ideas drifts away from democracy and grows towards violent extremism. A young person is inspired and starts to believe in an extremist narrative. He is going to take on more personal consequences and glorifies violence in order to achieve his ideals. Radicalisation is one way to become unhinged." (Stichting School & Veiligheid)

FACTS AND FIGURES

There are no direct figures on the number of radicalised persons within Dutch society, but there are figures on radicalised persons who have committed (violent) extremist crimes. There are, for example, several hundreds of people from the Netherlands, especially young people, who have travelled or tried to travel to conflict zones.[13] There are also multiple convictions for extremist activism such as threats, vandalism and arson. The percentage of people who commit criminal offences because of extremism is not high, but often these people come from extremist networks consisting of people who have not (yet) started (violent) extremist activities themselves. The number of people who have been radicalised in a worrying manner is therefore much higher than the number of people who have committed a known criminal offence.

MORE INFORMATION

See the Sources. In addition:

- PlatformJEP provides answers to the most important questions and provides an overview of different sources of information: www.platformjep.nl

This fact sheet was developed by Landelijk Steunpunt Extremisme (LSE) in collaboration with various chain partners.

RADICALISATION

"Extremism is the phenomenon wherein individuals or groups willingly cross the limits of the law in pursuit of their ideals. Extremism is different from activism. Activists can be vocal but without applying violence and abide by the limits of the law. Activism becomes extremism when criminal offences, such as violent threats and destruction of property, are deliberately committed." (NCTV)

There are several descriptions of radicalisation processes; some focus on psychological factors and others on sociological or economic factors. Because radicalisation is a capricious and dynamic phenomenon, especially among young people, these descriptions cannot be applied to all situations. Therefore, there are no checklists one can use to identify radicalisation.

POSSIBLE SIGNS/INDICATORS: HOW TO IDENTIFY IT

Because there is no checklist for radicalisation, it is difficult to indicate which signs indicate worrying behaviour. We often see various factors can influence the radicalisation process and the process not following a fixed pattern. There are all kinds of signs that in themselves are not indicative of a problem (so-called 'weak signs'), but that taken together can indicate that a situation is worrying:

- The person isolates himself from family and old friends, and socialises with a new network.
- The person visits worrying social media groups, websites or meetings.
- The person is intensively engaged in a new ideological or religious identity.[12]
- The person applies us-against-them-language, lecturing others, especially on political topics.
- Excessive truancy or quitting study, work, sports, or other hobbies.
- There has recently been a major event in the person's life, ranging from a move or a change of school or loss of work to death.

RISK FACTORS: WHO IS EXTRA VULNERABLE?

Because of the various forms of radicalisation there is no fixed profile of persons who are more likely to radicalise, but the risk of someone radicalising is increased when psychopathology is involved (psychosocial problems, mild intellectual disability, behavioural problems) and unstable living conditions (disrupted family/relationship, work and housing problems, drinking and/or drug use). Additionally, radicalisation mainly occurs among young people. A possible factor is brain development with regard to rational decision making which develops more slowly during the adolescent phase than other brain regions. This makes it difficult for young people between the ages of 14 and 23 to make informed choices, to think in abstract terms, and to understand the consequences of their choices.

STEPS TO FOLLOW WHEN YOU ENCOUNTER SIGNS OF RADICALISATION

Stay in contact and do not push away the person for his behaviour and statements. This may strengthen the behaviour and cause a person to (further) isolate himself. Engaging with non-radical persons is an important protecting factor against (further) radicalisation, and the present non-radical network also allows other persons and professionals to come into contact with the person. If there is radicalisation, several factors and approaches will have to be considered in order to reach the person and apply protective factors. Additionally, there are conversational techniques and interventions that can be used.

ADVICE

Employees of the Landelijk Steunpunt Extremisme (LSE) (the Dutch National Extremism Support Centre) are available to talk to you and discuss situations involving radicalisation. They provide advice on what you can do yourself and are able to indicate where professional help and possible reporting to the authorities are needed. If the situation does indeed turn out to be worrying, the case managers can help you. They are specialised in supporting and assisting people, families and professionals who are dealing with radicalisation. Web address: www.landelijksteunpuntextremisme.nl
Phone number: **088 - 20 80 080**.

DUTCH TRANSLATION

See here.

DEVELOPED BY

This fact sheet was developed by the Landelijk Steunpunt Extremisme (LSE) | National Support Centre for Extremism (LSE) in partnership with other organisations (see Organisations involved).

DOWNLOADS

This fact sheet can be accessed for free online and downloaded as a pdf, both in Dutch and in English.

This fact sheet was developed by Landelijk Steunpunt Extremisme (LSE) in collaboration with various chain partners.
