# Supplementary material for: Lesser-known types of violence: Helping nurses and midwives to signal and act
Source: Int J Nurs Stud Adv. 2022 Sep 17;4:100098. doi: 10.1016/j.ijnsa.2022.100098 (PMC11080451; doi:10.1016/j.ijnsa.2022.100098)
Supplement: Supplementary file 1 [file mmc1.zip › Factsheets English/Self-harm - sources.pdf]

SOURCES SELF-HARM

ORGANISATIONS INVOLVED

The following organisations were involved in making this fact sheet:

- Fivoor, forensic and intensive psychiatric care. For questions and/or remarks about the fact sheet, please email the main author: Nienke Kool, n.kool@fivoor.nl , nurse and researcher in the area of self-harm.
- Stichting Zelfbeschadiging, organisatie voor en door mensen die zichzelf beschadigen en hun omgeving, Dwayne Meijnckens, dwayne@zelfbeschadiging.nl / www.zelfbeschadiging.nl
- Veilig Thuis, Juliette Heetman, heetman@xs4all.nl
- Nadine Callens, nadine.callens1@telenet.be, author of the book "zelfverwonding bij jongeren, een gids voor leerkrachten, leerlingenbegeleiders, ouders en vrienden", uitgeverij Garant-Maklu.

SOURCES

The following documents and other sources provide more information about the topic of this fact sheet:

- Handreiking 113 zelfmoordpreventie voor de huisartsenpraktijk. www.113.nl/sites/default/files/113/preventie/Handreiking%20113%20zefmoordpreventie%20huisartsen.pdf
- Nienke Kool, 2011. Bejegening. – te verkrijgen op verzoek bij de hoofdauteur Nienke Kool.
- Landelijke Stichting Zelfbeschadiging. Alternatieven voor zelfbeschadiging. www.zelfbeschadiging.nl/alternatieven-voor-zelfbeschadiging zelfbeschadiging.nl/wp-content/uploads/2016/07/Alternatieven-voor-zelfbeschadiging-1.pdf (bekeken op 16 aug 2018).
- Landelijke Stichting Zelfbeschadiging. Tips voor lotgenoten. – te verkrijgen op verzoek bij de hoofdauteur Nienke Kool.
- Landelijke Stichting Zelfbeschadiging. Zelfbeschadiging: wat kun jij doen om te helpen? www.zelfbeschadiging.nl/wat-kun-jij-doen-om-te-helpen/
- zelfbeschadiging.nl/wp-content/uploads/2017/01/Zelfbeschadiging-wat-kun-jij-doen-om-te-helpen-2.pdf (bekeken op 16 aug 2018)
- www.zelfbeschadiging.nl
- www.sameninmijnschoenen.nl A website to provide easy access information about self-harm for young people who self-harm, next of kin and professionals.

This fact sheet was developed by Fivoor in collaboration with various chain partners. september 2020
