# Supplementary material for: Lesser-known types of violence: Helping nurses and midwives to signal and act
Source: Int J Nurs Stud Adv. 2022 Sep 17;4:100098. doi: 10.1016/j.ijnsa.2022.100098 (PMC11080451; doi:10.1016/j.ijnsa.2022.100098)
Supplement: Supplementary file 1 [file mmc1.zip › Factsheets English/Self-harm.pdf]

SELF-HARM

ALWAYS USE THE REPORTING CODE WHEN YOU ENCOUNTER A FORM OF (DOMESTIC) VIOLENCE, ABUSE, NEGLECT OR EXPLOITATION! ▶

This fact sheet is part of a series about *(domestic) violence, abuse, neglect, exploitation* and other types of harm that may be inflicted onto someone in a power-imbalanced relationship. Power-imbalanced relationships can exist with anyone, for example: an (ex-)partner, a child, a parent, a sibling, another family member, an informal or a professional carer, a friend, a flatmate or neighbour, a teacher, a colleague or supervisor, or just someone you know. These fact sheets describe different types of harm that can be inflicted in these relationships. They are meant as an add-on to the Dutch Reporting Code for these issues (English version here) and were developed for two reasons: 1) To provide professionals with an overview of all the types of harm that exist, to aid them in identifying both well-known and lesser-known types (see the Overview). 2) Signs/indicators may vary greatly by type of harm and certain types of harm require specific courses of action; the fact sheets help professionals with identifying the signs/indicators and risk factors of *each specific type* of harm and with acting appropriately when they do. Note: the general 5 steps in the Reporting Code are applicable to all types of harm in power-imbalanced relationships; the factsheets provide more guidance within these 5 steps – they are an add-on, not a replacement.

Below is a brief introduction to this topic, an overview of the signs/indicators and risk factors associated with this type of harm, and points of attention for when you encounter it.

WHAT IS SELF-HARM?

Self-harm is the self-inflicted physical injury or damage in a repeating pattern, without conscious suicidal intent. It is often an expression of intense, overwhelming emotions or feelings of emptiness that are hard to bear for those who damage themselves. Usually it has little to do with suicidality; self-harm is a survival strategy for people when they lack better alternatives.

POSSIBLE SIGNS/INDICATORS: HOW TO IDENTIFY IT

It is often not visible that someone is harming him- or herself. Specific signs are scars of (e.g.) cuts and burns, wearing covering clothing (e.g. long sleeves in summer) and not participating in activities that require a person to change clothes, such as sports and swimming. There may also be general signs that someone is not doing well, such as withdrawal, emotional outbursts and a deterioration in concentration and performance.

FACTS AND FIGURES

- The prevalence of self-harm among the adult population is 5.5%, among adolescents it is 15% and among youths it is 4%.
- Women are 1.5 times more likely than men to self-harm.
- The age of onset is usually between 12 and 14 years; the peak is in adolescence.
- The most common methods are cutting, scratching and burning.
- There are no figures on the course or prognosis of self-harming behaviour. In practice, the majority of people stop self-harming before the age of 30.

ADVICE AND HELP

For more information and advice on self-harm, please contact:

- Your general practitioner for basic care and referral
- the Stichting Zelfbeschadiging (the "Self-harm Foundation") for getting in touch with people with lived experience
- Fivoor (info@fivoor.nl, 088 02 82 220, ask for Nienke Kool) for professional and specific questions

Usually, self-harm is not a matter for Veilig Thuis ("Veilig Thuis" means "Safe at Home" in Dutch, it is the organization in the Netherlands for advice on, referrals to and reporting of any type

This fact sheet was developed by Fivoor in collaboration with various chain partners. september 2020

SELF-HARM

of (domestic) violence, abuse, neglect or exploitation, or other types of harm in power-imbalanced relationships) and the Reporting Code. However, in the following situations it is important to contact Veilig Thuis at **0800 20 00**:

- The cause of self-harm is related to an unsafe domestic situation/domestic violence and/or child abuse. It is quite possible that this will only become clear after a few conversations, because a trusting relationship needs to be established before people speak about these issues. If it is clear that there is an unsafe home, the reporting code must be followed.
- There may be doubts as to whether the injuries were self-made or made by someone else. If you have any doubts about this, discuss this with the person in question. If there is violence by others, contact Veilig Thuis for advice or reporting. The following signals may indicate violence by others:
  - when someone's story does not match their injuries,
  - injuries have been inflicted in places that are difficult to access for the person in question.
- If the person who self-harms is an adult, it is important to check whether they have children. In that case, consider a consultation with Veilig Thuis.

In case of acute danger call the emergency services at the phone number **112**.

RISK FACTORS: WHO IS EXTRA VULNERABLE?

There is no single reason why people harm themselves. Examples of risk factors that *can* make people vulnerable to self-harming behaviour are:

- Early childhood trauma (child abuse, whatever form it takes).
- The lack of support in difficult phases of life.
- Perfectionism and impulsivity.
- Being bullied.
- Being unable to express feelings.
- Slight intellectual disability.
- All kinds of psychiatric disorders such as psychotic, mood and personality disorders, addiction, eating disorder, post-traumatic stress disorder and autism

.

COURSE OF ACTION: WHAT TO DO WHEN THERE ARE (SUSPICIONS OF) SELF-HARM?

Someone who harms himself is often ashamed and anxious about being open towards others. Therefore, in the first contact moment with someone who harms him- or herself, it is essential that there is an open, empathic, non-judgmental attitude, with understanding of the underlying suffering. The contact moment should be as equal as possible and be aimed at building a collaboration.

In addition:

- Discuss (a suspicion of) self-harm directly with the person who is harming him- or herself.
- If someone has just harmed themselves, check together if (medical) care is needed for the wound(s) and agree who will provide that care.
- Usually there is no suicidality, but ask about it. This can also be done by directly asking: do you want to die? If this is the case, see also the "Handreiking 113 zelfmoordpreventie".
- Search together for meaning and follow-up steps, sometimes this can entail contact with people with lived experience (Stichting Zelfbeschadiging) and/or a few coaching conversations, but it may also entail a long therapeutic process. If referral is needed, supervise this process and stay involved in the background until someone has landed well (a warm transfer).
- Keep an eye for the impact on family and loved ones, if they are in the picture. Support them too.

MORE INFORMATION

See the Sources.
