# Supplementary material for: Lesser-known types of violence: Helping nurses and midwives to signal and act
Source: Int J Nurs Stud Adv. 2022 Sep 17;4:100098. doi: 10.1016/j.ijnsa.2022.100098 (PMC11080451; doi:10.1016/j.ijnsa.2022.100098)
Supplement: Supplementary file 1 [file mmc1.zip › Factsheets English/Sexual violence by strangers - sources.pdf]

SOURCES SEXUAL VIOLENCE AGAINST ADULTS BY STRANGERS

NOTE

For all forms of domestic violence and child abuse, the Dutch Reporting Code (English version here) for these issues **must** be applied in the Netherlands by all groups of professionals named in the Reporting Code law. Sexual violence against adults by strangers does not fall under the definition of domestic violence or child abuse in the Netherlands and therefore, it is not legally required to use the reporting code when you encounter it as a professional. However, the reporting code **may** be used. Because we feel it is a useful guideline for professionals for this type of harm as well, and because it is important that professionals (e.g. general practitioners) can identify this type of harm and take the right steps, this factsheet was compiled.

ORGANISATIONS INVOLVED

The following organisations were involved in making this fact sheet:

- Movisie. For questions and/or remarks about the fact sheets, please email the lead author: Wilma Schakenraad, w.schakenraad@movisie.nl
- Veilig Thuis
- Atria, kennisinstituut voor emancipatie en vrouwengeschiedenis

SOURCES

The following documents and other sources provide more information about the topic of this fact sheet:

Websites

- Kennisdossier seksuele grensoverschrijding van Rutgers: www.rutgers.nl/feiten-en-cijfers/kennisdossiers/kennisdossier-seksuele-grensoverschrijding
- www.seksueelgeweld.info . Website voor slachtoffers van seksueel geweld, en voor betrokkenen en verwijzers. Zie hierop ook de sociale kaart met hulpaanbod in Nederland voor slachtoffers en plegers van seksueel geweld.
- Dossier seksueel geweld op www.huiselijkgeweld.nl/dossiers/seksueel-geweld
- www.act4respect.nl (website wordt eind 2018 gelanceerd).

Publications

- Berlo van, W. & Beek I. van (2015). Whitepaper Seksuele grensoverschrijding en seksueel geweld. Feiten en cijfers Utrecht: Rutgers en Movisie.
- Bicanic, I., Jongh, A., de, Lagro-Janssen, T. & Leusink, P. (2016). Centrum seksueel geweld voor acute slachtoffers. Huisarts & Wetenschap. 59 (6), 265-267.
- Centraal Bureau voor de Statistiek (2012). Integrale Veiligheidsmonitor 2011. Landelijke rapportage. Den Haag: Centraal Bureau voor de Statistiek.
- European Union Agency for Fundamental Rights (FRA) (2014). Violence against women: an EU-wide survey. Luxemburg: publications Office of the European Union.
- Graaf, H. de , & Wijsen, C. (red.) (2017). Seksuele gezondheid in Nederland. Utrecht: Rutgers i.s.m. RIVM. Zie: www.rutgers.nl/sites/rutgersnl/files/PDF-Onderzoek/Seksuele_Gezondheid_in_NL_2017_23012018.pdf
- Haas, S. de (2012). Seksueel grensoverschrijdend gedrag onder jongeren en volwassenen in Nederland. In Tijdschrift voor Seksuologie, 36(2), 136-145.
- Haas, S. de (2014). Seksueel geweld en seksuele grensoverschrijding. In H. de Graaf, B. Bakker & C. Wijsen, Een wereld van verschil. Seksuele gezondheid van LHBT's in Nederland 2013. Utrecht: Rutgers WPF. Kühl, M., Schakenraad, W., & Beek, I. van (2017). Werken met volwassen slachtoffers van seksueel geweld. In: Höing, M., & Janssen, J., Boer, A., & Liebregts, M. (red.). Bespreekbaar maken van seksualiteit en intimiteit. Handboek voor professionals in zorg en welzijn. Bussum: Coutinho.
- Nationaal Rapporteur Mensenhandel en Seksueel Geweld tegen Kinderen (2014). Op goede grond. De aanpak van seksueel geweld tegen kinderen. Den Haag: Nationaal Rapporteur.
- Römkens, R., Jong, T. de en Harthoorn, H. (2014). Geweld tegen vrouwen. Europese onderzoeksgegevens in de Nederlandse context. Amsterdam: Atria.
- Rutgers WPF (2013). Wat maakt het verschil? Diversiteit in de seksuele gezondheid van LHBT's, een verkenning. Utrecht: Rutgers WPF
