# Supplementary material for: Lesser-known types of violence: Helping nurses and midwives to signal and act
Source: Int J Nurs Stud Adv. 2022 Sep 17;4:100098. doi: 10.1016/j.ijnsa.2022.100098 (PMC11080451; doi:10.1016/j.ijnsa.2022.100098)
Supplement: Supplementary file 1 [file mmc1.zip › Factsheets English/Sexual violence by strangers.pdf]

SEXUAL VIOLENCE AGAINST ADULTS BY STRANGERS

ALWAYS USE THE REPORTING CODE WHEN YOU ENCOUNTER A FORM OF (DOMESTIC) VIOLENCE, ABUSE, NEGLECT OR EXPLOITATION! ▶

This fact sheet is part of a series about *(domestic) violence, abuse, neglect, exploitation* and other types of harm that may be inflicted onto someone in a power-imbalanced relationship. Power-imbalanced relationships can exist with anyone, for example: an (ex-) partner, a child, a parent, a sibling, another family member, an informal or a professional carer, a friend, a flatmate or neighbour, a teacher, a colleague or supervisor, or just someone you know. These fact sheets describe different types of harm that can be inflicted in these relationships. They are meant as an add-on to the Dutch Reporting Code for these issues (English version here) and were developed for two reasons: 1) To provide professionals with an overview of all the types of harm that exist, to aid them in identifying both well-known and lesser-known types (see the Overview). 2) Signs/indicators may vary greatly by type of harm and certain types of harm require specific courses of action; the fact sheets help professionals with identifying the signs/indicators and risk factors of *each specific type* of harm and with acting appropriately when they do. Note: the general 5 steps in the Reporting Code are applicable to all types of harm in power-imbalanced relationships; the factsheets provide more guidance within these 5 steps – they are an add-on, not a replacement.

Below is a brief introduction to this topic, an overview of the signs/indicators and risk factors associated with this type of harm, and points of attention for when you encounter it.

WHAT IS SEXUAL VIOLENCE?

Sexual violence is defined by law as a form of assault and rape, i.e. penetration (rape) or other sexual acts (assault) in which violence is used, a threat of violence, or abuse of a vulnerable situation or condition that made it impossible for someone to refuse (e.g. substance use) (Van Beek & Van Berlo, 2015).*

In the Istanbul Convention the following definition is used: The vaginal, anal or oral penetration of another person's body with a body part or object without mutual consent. This means that the sexual act is performed without the consent of the other person.

WHY IS IT DIFFICULT TO RECOGNISE SIGNS OF SEXUAL VIOLENCE?

It is difficult to recognise signs of sexual violence because most signs are non-specific: signs such as unusual behaviour, but also behaviour that is too "quiet" or too "active", do not indicate one cause specifically. However, the combination of signs can reinforce a suspicion. Victims usually do not disclose sexual violence, not even to their GP.

RISK FACTORS: WHO IS EXTRA VULNERABLE?

Sexual violence (which in this fact sheet is about sexual violence by strangers, so such violence by intimate partners or other people known to the victim lies outside its scope) can affect anyone, but there are groups that run a greater risk. This applies to women and lesbians, homosexuals, bisexuals, transgender persons and intersex persons (LHBTI), but also to people with negative childhood experiences. Previous experiences with sexual violence are a risk factor: almost a quarter of men and almost half

FACTS AND FIGURES

Reporting and declaration

Research by the Centraal Bureau voor de Statistiek (CBS) shows that the vast majority of sexual offences are not reported to the police (9% in 2011). If the perpetrator is an unknown person, the victim waits on average 10 days to report; if the perpetrator is a known person, however, it takes an average of 8 months (National Rapporteur on Trafficking in Human Beings and Sexual Violence against Children, 2014).

Relationship with the perpetrator

About 30% of the perpetrators of sexual violence in women are their own partner or ex-partner, about 15% of the perpetrators are met during nightlife. Approximately 19% of the female victims experience sexual violence by unknown perpetrators. Men are mainly victims of sexual violence where a friend is the perpetrator, not necessarily a (former) intimate relationship (28%). Partners or ex-partners are the perpetrators of sexual violence against men in 18.5% of cases and 17.4% of the male victims are violated by unknown perpetrators (De Haas, 2012).

LGBTI's experience relatively high levels of sexual violence (Act4Respect). One in five gay and bisexual men have experienced a form of sexual violence

* This fact sheet follows the terminology of the Whitepaper on sexual harassment and sexual violence of Movisie and Rutgers (2015). This differs from the terminology used by Veilig Thuis.

of women who have experienced sexual violence experience this again later in life (revictimisation). In addition, social norms (such as socially accepted gender-stereotypical views, broadly supported double standards, general negative views about women and girls, and traditional views on male and female roles) can increase the risk of sexual violence (Act4Respect). Finally, drug use is a risk factor: for 31% of women and 23% of men, alcohol or drugs were used by the victim and/or the perpetrator before or during the event of sexual violence (De Graaf & Wijsen, 2017).

ASSISTANCE

In the event of a recent assault or rape victims are advised to report to the Centrum Seksueel Geweld (CSG) | the Sexual Violence Centre via 0800-0188 (preferably within the first seven days after the assault occurred). The CSG offers medical, forensic and psychological support after sexual violence in 16 regions A team of doctors, police, and emergency workers collaborate in the CSGs. This makes it an integrated care system; the victim preferably only has to come to one location. The victim receives medical assistance, a forensic analysis can be performed, a report can be made to the police (only if the victim wants to do this) and, if needed, psychological assistance is offered after a period of watchful waiting (monitoring the recovery process). Because of this integrated approach, the victim does not have to recount the violence more often than necessary (Bicanic, Engelhard & Sijbrandij, 2014).

If the sexual violence did not occur *recently*, the victim can contact the police or Slachtofferhulp Nederland (the Dutch Support organization for victims of violence). The Verbreek de Stilte helpline is part of the Dutch Victim Support Service and can be reached by telephone or chat. Here, social care professionals offer victims a listening ear; they also look for suitable help. Victim Support also offers legal, practical and emotional support; the assistance is free and always nearby.

Victims can also contact Veilig Thuis.

at some point in their lives, as compared to 6% of heterosexual men. Lesbian women are also relatively often victims of sexual violence: 37% of lesbian women have experiences with a form of sexual violence (De Haas, 2014; Rutgers WPF, 2013).

OTHER FIGURES

- Almost three quarters (73%) of all Dutch women have been sexually intimidated at some point in their lives;
- One in ten women have been raped at some point in their lives (FRA, 2014);
- 11% of all Dutch women have experienced sexual violence by a (former) partner;
- 12% of all Dutch women have been a victim of sexual violence by someone other than their partner.

MORE INFORMATION

See the Sources.

Separate fact sheets have been developed on other sexual violence topics:

- violence in the (ex-)partner relationship (including sexual violence)
- sexual behaviour that crosses boundaries between young people
- boundary-crossing behaviour against people with a disability
- online sexual harassment

ADVICE/REPORTING

When the sexual violence occurred recently, contact:

- The Centrum Seksueel Geweld (CSG) | the Sexual Violence Centre is the expertise centre of the Netherlands for victims of sexual violence in the acute phase (<7 days). At CSG, a team of doctors, nurses, police, psychologists, social workers and sexologists work together to provide specialist care to victims of assault and rape. Call **0800-0188**.

When the sexual violence occurred less recently, contact:

- Slachtofferhulp Nederland: telephone **0900-0101**
- Veilig Thuis ("Veilig Thuis" means "Safe at Home" in Dutch, it is the organization in the Netherlands for advice on, referrals to and reporting of any type of (domestic) violence, abuse, neglect or exploitation, or other types of harm in power-imbalanced relationships). Telephone: **0800 20 00**, free of charge and always open (24 hours per day, 7 days a week). It is possible to call anonymously and/or to call for advice or information only, without reporting someone.

In case of acute danger call the emergency services at the phone number **112**.

DUTCH TRANSLATION

See here.

This fact sheet was developed by Movisie in collaboration with various chain partners in the fight against sexual violence. september 2020
