# Supplementary material for: Lesser-known types of violence: Helping nurses and midwives to signal and act
Source: Int J Nurs Stud Adv. 2022 Sep 17;4:100098. doi: 10.1016/j.ijnsa.2022.100098 (PMC11080451; doi:10.1016/j.ijnsa.2022.100098)
Supplement: Supplementary file 1 [file mmc1.zip › Factsheets English/Stalking - sources.pdf]

SOURCES STALKING

NOTE

For all forms of domestic violence and child abuse, the Dutch Reporting Code (English version here) for these issues **must** be applied in the Netherlands by all groups of professionals named in the Reporting Code law. Stalking does not fall under the definition of domestic violence or child abuse in the Netherlands and therefore, it is not legally required to use the reporting code when you encounter it as a professional. However, the reporting code **may** be used. Because we feel it is a useful guideline for professionals for this type of harm as well, and because it is important that professionals (e.g. general practitioners) can identify this type of harm and take the right steps, this factsheet was compiled.

ORGANISATIONS INVOLVED

The following organisations were involved in making this fact sheet:

- Slachtofferhulp Nederland. For questions and/or remarks about the fact sheet, please email the main author: Franck Wagemakers, f.wagemakers@slachtofferhulp.nl
- AROSA: Magda Vogelezang
- Nationale politie: Cleo Brandt en Berdien Zuurveen
- Veilig Thuis: Inge Sauvé

SOURCES

The following documents and other sources provide more information about the topic of this fact sheet:

- www.slachtofferhulp.nl/gebeurtenissen/stalking
- www.movisie.nl/publicatie/u-wordt-gestalkt "Als u wordt gestalkt", Movisie
- www.slachtofferhulp.nl/gebeurtenissen/stalking/bewijs-verzamelen-gestalkt
- www.slachtofferhulp.nl/gebeurtenissen/stalking/rechten/#bekijk-uw-rechten

This fact sheet was developed by Slachtofferhulp Nederland in collaboration with various chain partners.

september 2020
