# Supplementary material for: Lesser-known types of violence: Helping nurses and midwives to signal and act
Source: Int J Nurs Stud Adv. 2022 Sep 17;4:100098. doi: 10.1016/j.ijnsa.2022.100098 (PMC11080451; doi:10.1016/j.ijnsa.2022.100098)
Supplement: Supplementary file 1 [file mmc1.zip › Factsheets English/Stalking.pdf]

STALKING

This fact sheet is part of a series about *(domestic) violence, abuse, neglect, exploitation* and other types of harm that may be inflicted onto someone in a power-imbalanced relationship. Power-imbalanced relationships can exist with anyone, for example: an (ex-)partner, a child, a parent, a sibling, another family member, an informal or a professional carer, a friend, a flatmate or neighbour, a teacher, a colleague or supervisor, or just someone you know. These fact sheets describe different types of harm that can be inflicted in these relationships. They are meant as an add-on to the Dutch Reporting Code for these issues (English version here) and were developed for two reasons: 1) To provide professionals with an overview of all the types of harm that exist, to aid them in identifying both well-known and lesser-known types (see the Overview). 2) Signs/indicators may vary greatly by type of harm and certain types of harm require specific courses of action; the fact sheets help professionals with identifying the signs/indicators and risk factors of *each specific type* of harm and with acting appropriately when they do. Note: the general 5 steps in the Reporting Code are applicable to all types of harm in power-imbalanced relationships; the factsheets provide more guidance within these 5 steps – they are an add-on, not a replacement.

Below is a brief introduction to this topic, an overview of the signs/indicators and risk factors associated with this type of harm, and points of attention for when you encounter it.

ALWAYS USE THE REPORTING CODE WHEN YOU ENCOUNTER A FORM OF (DOMESTIC) VIOLENCE, ABUSE, NEGLECT OR EXPLOITATION! ▶

WHAT IS STALKING?

Stalking (also known as stalking) is a deliberate and systematic harassment of someone, as a result of which that person no longer feels safe. It is often an invisible and difficult to understand terror; an accumulation of behaviour that is not always punishable in itself. Examples of stalking are chasing, threatening with violence, making unwanted phone calls and sending messages or ordering things in the name of someone else. Stalking can take place at home, elsewhere and/or online. Stalking is punishable in the Netherlands.[1]

Stalking is by definition a form of violence because it is a major violation of the perception of security in and on the lives of victims.

A stalker may have (had) an (intimate) relationship with the victim, but this is not necessary.

POSSIBLE SIGNS/INDICATORS: HOW TO IDENTIFY IT

The privacy of the victim is constantly and intentionally infringed, for example, with the aim of forcing the victim to react (for example, or to restore the relationship), frighten or hinder something.[2] Stalking concerns a pattern of behaviour in which every incident does not have to be very violent and/or punishable, but the combination of all those incidents is. For service providers it is important to realise that stalking has consequences for the feeling of safety and well-being of the victim. There is tension and stress. The victim is hindered in his daily functioning.

MORE INFORMATION

See the Sources and the publication "If you are stalked".

ADVICE/REPORTING

For advice, for reporting victims or perpetrators, and/or for referring someone to care (including shelters), call:

- Veilig Thuis ("Veilig Thuis" means "Safe at Home" in Dutch, it is the organization in the Netherlands for advice on, referrals to and reporting of any type of (domestic) violence, abuse, neglect or exploitation, or other types of harm in power-imbalanced relationships). Telephone: **0800 20 00**, free of charge and always open (24 hours per day, 7 days a week). It is possible to call anonymously and/or to call for advice or information only, without reporting someone.

In case of acute danger call the emergency services at the phone number **112.**

This fact sheet was developed by Slachtofferhulp Nederland in collaboration with various chain partners. september 2020

To estimate the risk that the person being stalked runs, several questions can be helpful (see p4 and 5 of the publication "If you are stalked").

POINTS OF ATTENTION WHEN GOING THROUGH THE 5 STEPS IN THE REPORTING CODE

For any form of (domestic) violence, abuse, neglect or exploitation, professionals in the Netherlands are required to use the Reporting Code. For general reporting code guidelines (such as the 5 steps in this code) visit the link; these are not described in this fact sheet. We do describe here points of attention in going through the 5 steps that are specific to the topic of this fact sheet. These are:

- An important rule of thumb: take the victim's feelings seriously: if they are really afraid, then there is actually a risk of danger/ threat.
- There are a number of rules of thumb for victims themselves in what they can do when they are stalked: see p7 and 8 of the publication "If you are stalked".
- It is important to gather evidence.3
- Tune in with the Police. In an acute dangerous or threatening situation, call the police immediately.
- Victims of stalking are entitled to protection. For example, the public prosecutor or judge can impose an area and/or contact ban on the stalker. Read: more about rights in case of stalking.4
- Involve neighbours, family members, friends and colleagues who can watch out and support.

If necessary, a mobile alarm system (AWARE) or a placement within the shelter can be realized.

- Slachtofferhulp Nederland (this name means "Victim Support in the Netherlands" in Dutch), telephone 0900 01 01, offers practical support but can also refer to help with processing or groups of fellow sufferers.

DUTCH TRANSLATION

See here.

This fact sheet was developed by Slachtofferhulp Nederland in collaboration with various chain partners. september 2020
