# Supplementary material for: Lesser-known types of violence: Helping nurses and midwives to signal and act
Source: Int J Nurs Stud Adv. 2022 Sep 17;4:100098. doi: 10.1016/j.ijnsa.2022.100098 (PMC11080451; doi:10.1016/j.ijnsa.2022.100098)
Supplement: Supplementary file 1 [file mmc1.zip › Factsheets English/The Child Check - sources.pdf]

SOURCES THE CHILD CHECK

ORGANISATIONS INVOLVED

The following organisations were involved in making this fact sheet:

- De Kindcheck. For questions and/or remarks about the fact sheet, please email the main author: Hester Diderich, h.diderich@kindcheck-ggz.nl
- Veilig Thuis, Wanda Lansbergen
- Radboud umc, Karin van Rosmalen-Nooijens
- SIEN, voor mensen met een verstandelijke beperking, Jolanda den Hartog
- Augeo

SOURCES

The following documents and other sources provide more information about the topic of this fact sheet:

- Diderich et al., 2013
- Augeo. De Kindcheck.
- Augeo magazine. De Kindcheck voor medici: signalen van ouders

This fact sheet was developed by De Kindcheck in collaboration with various chain partners. september 2020
