# Supplementary material for: Lesser-known types of violence: Helping nurses and midwives to signal and act
Source: Int J Nurs Stud Adv. 2022 Sep 17;4:100098. doi: 10.1016/j.ijnsa.2022.100098 (PMC11080451; doi:10.1016/j.ijnsa.2022.100098)
Supplement: Supplementary file 1 [file mmc1.zip › Factsheets English/The Child Check.pdf]

THE CHILD CHECK

SIGNALLING CHILD ABUSE BASED ON PARENTAL SIGNALS

ALWAYS USE THE REPORTING CODE WHEN YOU ENCOUNTER A FORM OF (DOMESTIC) VIOLENCE, ABUSE, NEGLECT OR EXPLOITATION! ▶

This fact sheet is part of a series about *(domestic) violence, abuse, neglect, exploitation* and other types of harm that may be inflicted onto someone in a power-imbalanced relationship. Power-imbalanced relationships can exist with anyone, for example: an (ex-)partner, a child, a parent, a sibling, another family member, an informal or a professional carer, a friend, a flatmate or neighbour, a teacher, a colleague or supervisor, or just someone you know. These fact sheets describe different types of harm that can be inflicted in these relationships. They are meant as an add-on to the Dutch Reporting Code for these issues (English version here) and were developed for two reasons: 1) To provide professionals with an overview of all the types of harm that exist, to aid them in identifying both well-known and lesser-known types (see the Overview). 2) Signs/indicators may vary greatly by type of harm and certain types of harm require specific courses of action; the fact sheets help professionals with identifying the signs/indicators and risk factors of *each specific type* of harm and with acting appropriately when they do. Note: the general 5 steps in the Reporting Code are applicable to all types of harm in power-imbalanced relationships; the factsheets provide more guidance within these 5 steps – they are an add-on, not a replacement.

Below is a brief introduction to this topic, an overview of the signs/indicators and risk factors associated with this type of harm, and points of attention for when you encounter it.

WHAT IS THE CHILD CHECK?

The 'Child Check' is part of the Dutch domestic violence and child abuse Reporting Code (English version here). The aim of the Child Check is to identify more children at serious risk of abuse or neglect.

The Child Check is especially meant for people who work with adult clients/patients, such as (family) doctors, nurses, social workers, psychiatrists and psychologists. The Child Check means that in your contacts with adult clients/patients you check whether there are children involved and assess whether they are safe.

SIGNS/INDICATORS: WHICH PARENTAL SIGNS/ INDICATORS TO LOOK OUT FOR

For example, you may perform the Child Check for adult clients/ patients:

- with serious psychological problems
- following a suicide attempt
- with an addiction to drugs and/or alcohol
- who are victims or perpetrators of domestic violence
- who are very aggressive and/or dangerous to firearms
- who show signs of severe personal neglect and/or have no permanent residence or domicile
- who are mentally disabled

FACTS AND FIGURES

Research on the Child Check shows that in 91% of the cases when the Child Check gives a positive result, there is a form of child abuse. Three quarters of these children were not yet known to Veilig Thuis (Diderich et al., 2013).
By identifying child abuse also based on **parental signals** instead of only on **child signals,** help and support can be organized for a family at an earlier stage. Since 2013, the Child Check is a mandatory part of the Dutch Reporting Code.
Children of parents with a mental disorder and/or an addiction carry an increased risk of neglect because parents, as a result of personal problems, are insufficiently able to provide needed care for their child (basic care, emotional and affective support).

MORE INFORMATION

See the Sources and:

- Augeo. The Child Check.
- Augeo magazine. The Child Check for doctors: signals from parents

This fact sheet was developed by De Kindcheck in collaboration with various chain partners. september 2020

It is also important to perform the Child Check when an adult is being held by the police/judiciary.

POINTS OF ATTENTION WHEN GOING THROUGH THE 5 STEPS IN THE REPORTING CODE

For any form of (domestic) violence, abuse, neglect or exploitation, professionals in the Netherlands are required to use the Reporting Code. For general reporting code guidelines (such as the 5 steps in this code) visit the link; these are not described in this fact sheet. We do describe here points of attention in going through the 5 steps that are specific to the topic of this fact sheet. These are:

- The Child Check should be performed as part of step 1 in the Reporting Code when mapping *signs/indicators*.
- If you have any doubts about the safety of children who are part of the adult's e.g. family, for example because the adult who you are in contact with has a limited social network, or because there is no other parent – then go through the steps of the Reporting Code (English version here). It is important to firstly denote the reasons why you doubt the safety of the children, based on which signs/indicators.
- When the Child Check turns out positive (i.e. concerns about the safety of the children persist after step 4 in the Reporting Code), ALWAYS report to Veilig Thuis. Then, in consultation with Veilig Thuis, you can discuss how you can best organize safety for the children.
- **Important**: To ensure that the Child Check is always performed when there are concerns about adult clients/patients, it is important to include standard Child Check questions in the clients/patients file. This way, the Child Check cannot be forgotten and colleagues can retrace steps taken earlier.

ADVICE/REPORTING

For advice, for reporting victims or perpetrators, and/or for referring someone to care (including shelters), call:

- Veilig Thuis ("Veilig Thuis" means "Safe at Home" in Dutch, it is the organization in the Netherlands for advice on, referrals to and reporting of any type of (domestic) violence, abuse, neglect or exploitation, or other types of harm in power-imbalanced relationships). Telephone: **0800 20 00**, free of charge and always open (24 hours per day, 7 days a week). It is possible to call anonymously and/or to call for advice or information only, without reporting someone.

In case of acute danger call the emergency services at the phone number **112**.

DUTCH TRANSLATION

See here.
