# Supplementary material for: Lesser-known types of violence: Helping nurses and midwives to signal and act
Source: Int J Nurs Stud Adv. 2022 Sep 17;4:100098. doi: 10.1016/j.ijnsa.2022.100098 (PMC11080451; doi:10.1016/j.ijnsa.2022.100098)
Supplement: Supplementary file 1 [file mmc1.zip › Factsheets English/Trafficking in human beings.pdf]

TRAFFICKING IN HUMAN BEINGS

This fact sheet is part of a series about *(domestic) violence, abuse, neglect, exploitation* and other types of harm that may be inflicted onto someone in a power-imbalanced relationship. Power-imbalanced relationships can exist with anyone, for example: an (ex-)partner, a child, a parent, a sibling, another family member, an informal or a professional carer, a friend, a flatmate or neighbour, a teacher, a colleague or supervisor, or just someone you know. These fact sheets describe different types of harm that can be inflicted in these relationships. They are meant as an add-on to the Dutch Reporting Code for these issues (English version here) and were developed for two reasons: 1) To provide professionals with an overview of all the types of harm that exist, to aid them in identifying both well-known and lesser-known types (see the Overview). 2) Signs/indicators may vary greatly by type of harm and certain types of harm require specific courses of action; the fact sheets help professionals with identifying the signs/indicators and risk factors of *each specific type* of harm and with acting appropriately when they do. Note: the general 5 steps in the Reporting Code are applicable to all types of harm in power-imbalanced relationships; the factsheets provide more guidance within these 5 steps – they are an add-on, not a replacement.

Below is a brief introduction to this topic, an overview of the signs/indicators and risk factors associated with this type of harm, and points of attention for when you encounter it.

ALWAYS USE THE REPORTING CODE WHEN YOU ENCOUNTER A FORM OF (DOMESTIC) VIOLENCE, ABUSE, NEGLECT OR EXPLOITATION! ▶

WHAT IS TRAFFICKING IN HUMAN BEINGS?

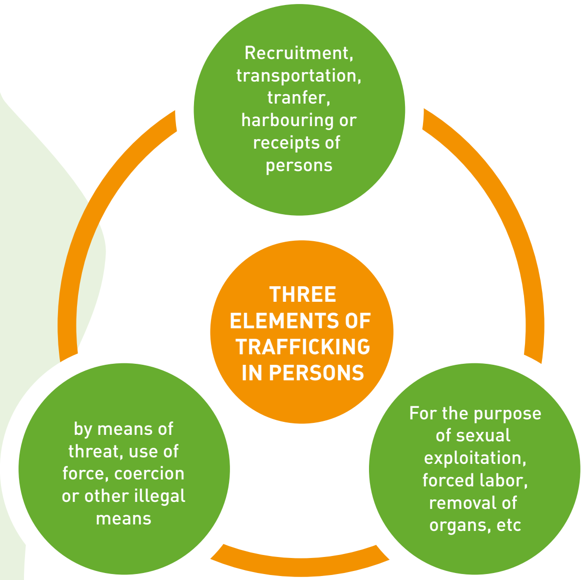

Figure 1. The three elements of Trafficking in Persons. from: www.sb.gov.hk/eng/special/bound/iimm.htm

FACTS AND FIGURES

- The number of victims of human trafficking in the Netherlands per year is estimated to be 6,250.
- This is 6 times more than the number of reported victims; human trafficking is often a hidden problem.
- 56% of all trafficking in human beings is **domestic** (meaning it happens to Dutch nationals within Dutch borders); the vast majority of this group is trafficked for the purpose of sexual exploitation
- About half of all domestic victims are minors
- Foreign victims often come from Romania, Poland, Hungary, Bulgaria and Nigeria.
- There are ICD-10 codes for trafficking in human beings
- Foreign victims of trafficking in human beings always have the right to a temporary residence permit
- There are shelters for foreign and domestic victims
- For information on organisations who work on this topic see www.wegwijzermensenhandel.nl

This fact sheet was developed by the Dutch national coordination centre against human trafficking (CoMensha) in collaboration with various chain partners.

TRAFFICKING IN HUMAN BEINGS

In the Netherlands, human trafficking (or 'trafficking in human beings' or 'trafficking in person') is understood to mean:
- **what:** recruiting (for work), transporting or housing someone,
- **how:** using coercion, violence, deception or abuse of a vulnerable position (such as with children or people with a mental disability),
- **the goal:** to reduce or not pay out someone's income (*exploitation*).

This takes place in the sex industry, as well as in many other labour sectors (such as agriculture, construction, domestic work, forced begging or crime (e.g. forced cannabis cultivation or drug dealing). Organ trafficking and forced surrogacy are also forms of trafficking in human beings. Human trafficking can take place across borders or within the Netherlands ('domestic human trafficking'). Domestic human traffickers for the purpose of sexual exploitation are often called 'loverboys' in the Netherlands (although we believe this term to be too euphemistic).

POSSIBLE SIGNS/INDICATORS: HOW TO IDENTIFY IT

All forms
- there is usually a companion with the person
- vague explanations for injuries
- threats (to the victim him- or herself or to relatives, e.g. younger sisters or parents)
- work related health problems (e.g. accidents at work in construction and multiple pregnancies, abortions, STIs or vaginal and genital complaints in sex work)
- psychosomatic health problems (psychological complaints that express themselves physically)
- drug/alcohol addiction
- tattoos of the human trafficker (such as the name or an icon)

Trafficking in human beings WITHIN borders (origin and destination country are the same)
- increasing isolation from family/friends + dependency on others
- suddenly having a lot of money / other clothing
- behavioural changes
- extortion (e.g. with movies)
- deteriorating school or work performance

TRAFFICKING IN HUMAN BEINGS ACROSS BORDERS:
- living in the workplace
- passport not in own possession
- did not arrange one's own travel
- work address not known
- accrued 'debts' (not really debts, but the traffickers say they have debts for the travel arrangements and possibly other matters)
- being undocumented

RISK FACTORS: WHO IS EXTRA VULNERABLE?
Previous victims of (sexual) violence, persons with psychological complaints (such as mild intellectual disabilities), originating from multi-problem or broken families, orphans, low self-esteem, homelessness, history of violence or trauma / youth support past, refugees / undocumented people, poverty, being easy to influence, age 12-24 years, LHBTI+ (for boys).

ADVICE/REPORTING
For advice, for reporting victims or perpetrators, and/or for referring someone to care (including shelters), call:
- Veilig Thuis ("Veilig Thuis" means "Safe at Home" in Dutch, it is the organization in the Netherlands for advice on, referrals to and reporting of any type of (domestic) violence, abuse, neglect or exploitation, or other types of harm in power-imbalanced relationships). Telephone: 0800 20 00, free of charge and always open (24 hours per day, 7 days a week). It is possible to call anonymously and/or to call for advice or information only, without reporting someone.
- the Dutch national coordination centre against human trafficking (CoMensha), telephone 033 44 81 186
- a domestic trafficking hotline

In case of acute danger call the emergency services at the phone number 112.

MORE INFORMATION
See the Sources.

DUTCH TRANSLATION
See here.

This fact sheet was developed by the Dutch national coordination centre against human trafficking (CoMensha) in collaboration with various chain partners.

POINTS OF ATTENTION WHEN GOING THROUGH THE 5 STEPS IN THE REPORTING CODE

For any form of (domestic) violence, abuse, neglect or exploitation, professionals in the Netherlands are required to use the Reporting Code. For general reporting code guidelines (such as the 5 steps in this code) visit the link; these are not described in this fact sheet. We do describe here points of attention that are specific to the topic of this fact sheet. These are:

- Human traffickers are often family members/friends/partners/acquaintances (in 68% of all cases of human trafficking in the Netherlands!).
- Speak to someone alone! If you do not speak the same language, engage an external interpreter
- Men also become victims of human trafficking, including of sexual exploitation.
- Pay extra attention to your own safety, that of the victim and his/her family
- People do not always see themselves as 'being trafficked', even when according to our standards and values they are. Regularly, given the context of their origin country, this point of view is understandable (which does not mean you should not act). At other times, people are not able (or refuse) to see the nature of their situation because of the influences of their trafficker. On top of this, even when you think someone is a 'victim', people rarely conceptualize themselves as such, so be hesitant in using this label.
- Human trafficking and human smuggling are not the same!
- Foreign people who have been trafficked often have little knowledge of their rights and little confidence in the police

This fact sheet was developed by the Dutch national coordination centre against human trafficking (CoMensha) in collaboration with various chain partners.
