# Supplementary material for: Lesser-known types of violence: Helping nurses and midwives to signal and act
Source: Int J Nurs Stud Adv. 2022 Sep 17;4:100098. doi: 10.1016/j.ijnsa.2022.100098 (PMC11080451; doi:10.1016/j.ijnsa.2022.100098)
Supplement: Supplementary file 1 [file mmc1.zip › Overview + all factsheets - English.pdf]

SPECIFIC TYPES AND TARGET POPULATIONS OF (DOMESTIC) VIOLENCE, ABUSE, NEGLECT, EXPLOITATION

in power-imbalanced relationships

Fact sheets and websites for professionals who work with the Dutch Reporting Code

AGAINST PARTNER
- (EX-)PARTNER VIOLENCE

AGAINST CHILD
- CHILD ABUSE
- PROTECTION OF THE UNBORN CHILD
- SHAKEN BABY SYNDROOM
- FEMALE GENITAL MUTILATION
- PEDIATRIC CONDITION FALSIFICATION
- GIRLS <18 YEARS WHO SELL SEX
- BOYS <18 YEARS WHO SELL SEX
- THE CHILD CHECK
- CHILDREN IN A HIGH-CONFLICT SEPARATION

AGAINST ELDERLY
- ELDER ABUSE

AGAINST PARENT
- PARENT ABUSE

AGAINST ...
- MALE ABUSE
- VIOLENCE AGAINST/ AMONG MIGRANTS
- VIOLENCE AGAINST PEOPLE WITH INTELLECTUAL DISABILITIES
- ABUSE BY INFORMAL CARERS

>1 TARGET POPULATION
- HUMAN TRAFFICKING
- ONLINE SEXUAL INTIMIDATION
- STALKING
- HONOUR-BASED VIOLENCE
- HIDDEN WOMEN
- ABANDONMENT
- FORCED MARRIAGE

PERPETRATORS
- PERPETRATORS

OTHER
Other types of violence in power-imbalanced relationships, such as against a brother or sister, an adult housemate or an acquaintance

This overview is intended to help professionals respond to violence in power-imbalanced relationships. There are also other types of violence, for which it is also useful to have knowledge of signs and what to do when you see signs. Examples are:

- Bullying
- Self-harm
- Sexual behaviour between young people that crosses boundaries
- Sexual violence against adults by strangers
- Radicalization

TYPES OF VIOLENCE
With the types of (domestic) violence, abuse, neglect and exploitation listed here above, there are six broader types of violence that often play a role

- PHYSICAL
- EMOTIONAL
- SEXUAL
- NEGLECT
- FINANCIAL
- BEING A WITNESS

ALWAYS USE THE REPORTING CODE WHEN YOU ENCOUNTER A FORM OF (DOMESTIC) VIOLENCE, ABUSE, NEGLECT OR EXPLOITATION!

(EX-)PARTNER VIOLENCE

ALWAYS USE THE REPORTING CODE WHEN YOU ENCOUNTER A FORM OF (DOMESTIC) VIOLENCE, ABUSE, NEGLECT OR EXPLOITATION! ▶

This fact sheet is part of a series about *(domestic) violence, abuse, neglect, exploitation* and other types of harm that may be inflicted onto someone in a power-imbalanced relationship. Power-imbalanced relationships can exist with anyone, for example: an (ex-)partner, a child, a parent, a sibling, another family member, an informal or a professional carer, a friend, a flatmate or neighbour, a teacher, a colleague or supervisor, or just someone you know. These fact sheets describe different types of harm that can be inflicted in these relationships. They are meant as an add-on to the Dutch Reporting Code for these issues (English version here) and were developed for two reasons: 1) To provide professionals with an overview of all the types of harm that exist, to aid them in identifying both well-known and lesser-known types (see the Overview). 2) Signs/indicators may vary greatly by type of harm and certain types of harm require specific courses of action; the fact sheets help professionals with identifying the signs/indicators and risk factors of *each specific type* of harm and with acting appropriately when they do. Note: the general 5 steps in the Reporting Code are applicable to all types of harm in power-imbalanced relationships; the factsheets provide more guidance within these 5 steps – they are an add-on, not a replacement.

Below is a brief introduction to the topic of (ex-)partner violence, an overview of the signs/indicators and risk factors associated with this type of violence, and points of focus for when you encounter it.

WHAT IS (EX-)PARTNER VIOLENCE?

(Ex-)partner violence is any form of violence between partners or ex-partners. A (constant) threat of violence is also regarded as (ex-)partner violence. This includes all forms of violence; physical, emotional/psychological, sexual, financial or online, and stalking.

We can distinguish different categories of partner violence, including:

1. Common couple violence

This form of violence often arises from powerlessness, through loss of control. The perpetrator can be either partner. There can be a difference in the severity of the violence and the consequences differ per person. Consequences may be greater for women than for men (Daru et al., 2016).

2. Intimate terrorism

One-sided (threatening) serious violence, usually but not always committed by men. It is deliberate violence, partner terror, aimed at control over the partner.

POSSIBLE SIGNS/INDICATORS: HOW TO IDENTIFY IT

Signs/indicators that may indicate someone is a victim of (ex-) partner violence can be divided into:

- **physical signs**

  Repeated injuries: bruises, cuts, bites or head injuries, fractures (especially nose, bones, ribs), dislocations (especially jaw and shoulder), burns, loss of teeth, skull injuries and damage to the genitals.

- **psychosomatic signs**

  Depression, tremors, frequent headaches or stomach aches, fatigue, anxiety, sleep disorders, hyperventilation, heart palpitations.

FACTS AND FIGURES

According to a report by the FRA (European Union Agency for Fundamental Rights, 2014) that focused exclusively on female victims, 9% of Dutch women have experienced physical or sexual violence by their current partner and 25% by an ex-partner since age 15:

- One in five women has faced physical abuse by a partner or ex-partner.
- 11% of women have experienced sexual violence by a partner or ex-partner.
- In the past year, 3% of women have sometimes avoided their own homes for fear of violence.

(Ex-) partner violence makes up for more than 60% of domestic violence cases. Women (60%) are more likely to be victims of (ex-)partner violence than men (40%). The violence that men commit is more often of a structural nature than the violence that women commit. For both men and women, gender-specific assistance and care is possible and needed.

This fact sheet was developed by Movisie in collaboration with various chain partners. september 2020

(EX-)PARTNER VIOLENCE

- **behavioural signs**
  Difficulty making eye contact, always postponing appointments, a submissive attitude, vague requests for help, being anxious, women who are always accompanied by their partner, and/or cancelling last-minute consultations. on the other side of the spectrum: more than average use of care providers (GPs), more use of sleeping tablets and painkillers, or looking for an explanation for complaints more than average.

A complete overview of possible signs/indicators, including signs exhibited by children can be found here.

AT-RISK GROUPS

Partner violence can among all layers and groups within the population. Certain stress factors increase the risk of problems and violence occurring in the relationship, which is why in reality partner violence tends to be seen more often with couples who are dealing with stressors such as poverty, unemployment or other problems. This often involves a a combination of factors that result in (ex-)partner violence.

RISK FACTORS?

We can distinguish the following (groups of) risk factors:

1. Power discrepancy and dependence. Relational dependence, economic dependence, dependence because of residence status, care dependence, or a combination thereof.
2. Gender inequality.
3. Lack of defence mechanisms and social skills.
4. Isolation.
5. Problematic divorces.
6. Stress caused by a multitude of problems in the family, such as financial problems and debts, problems at work or problems due to lack of work, poor housing.
7. Alcohol and/or drug abuse.
8. Previous abuse (in earlier relationships or in the present relationship).
9. Behavioural problems at a young age.
10. Culturally determined tolerance towards violence.
11. Personal problems, including psychological or psychiatric problems and/or a mental disability.
12. Pregnancy (the correlation with partner violence is high).
13. Experienced violence in the past or witnessed partner violence in parents (girls are more likely to become victims and boys more likely to become perpetrators).

POINTS OF ATTENTION WHEN GOING THROUGH THE 5 STEPS IN THE REPORTING CODE

For any form of (domestic) violence, abuse, neglect or exploitation, professionals in the Netherlands are required to use the Reporting Code. For general reporting code guidelines (such as the 5 steps in this code) visit the link; these are not described in this fact sheet. We do describe here points of attention in going through the 5 steps that are specific to the topic of this fact sheet. These are:

If there is a suspicion of partner violence, always carry out the "Child Check". A separate fact sheet has been published on this subject.

"RELATIEWIJS" PROGRAMME

How do you know as a professional when behaviour in a relationship crosses a boundary? What do you do if there appears to be (ex-)partner violence? How do you make sure people can start talking about it? "RelatieWijs" is an aid in going through all steps of the Reporting Code. It helps in documenting signs/indicators of

MORE INFORMATION

See the Sources.

ADVICE/REPORTING

For advice, for reporting victims or perpetrators, and/or for referring someone to care (including shelters), call:

- Veilig Thuis ("Veilig Thuis" means "Safe at Home" in Dutch, it is the organization in the Netherlands for advice on, referrals to and reporting of any type of (domestic) violence, abuse, neglect or exploitation, or other types of harm in power-imbalanced relationships). Telephone: **0800 20 00**, free of charge and always open (24 hours per day, 7 days a week). It is possible to call anonymously and/or to call for advice or information only, without reporting someone.

In case of acute danger call the emergency services at the phone number **112**.

DUTCH TRANSLATION

See here.

This fact sheet was developed by Movisie in collaboration with various chain partners. september 2020

violence and assessing relational (boundary-crossing) behaviour based on objective criteria. These criteria can enable the partners to start talking about the identified behaviours: they make it possible to describe the behaviour in concrete terms and indicate when further research is needed. RelatieWijs uses seven criteria to determine whether relational behaviour is 'healthy' or boundary-crossing: mutual consent, voluntary action, equality, self-determination, appropriate to the context, self-respect and respect for the other.

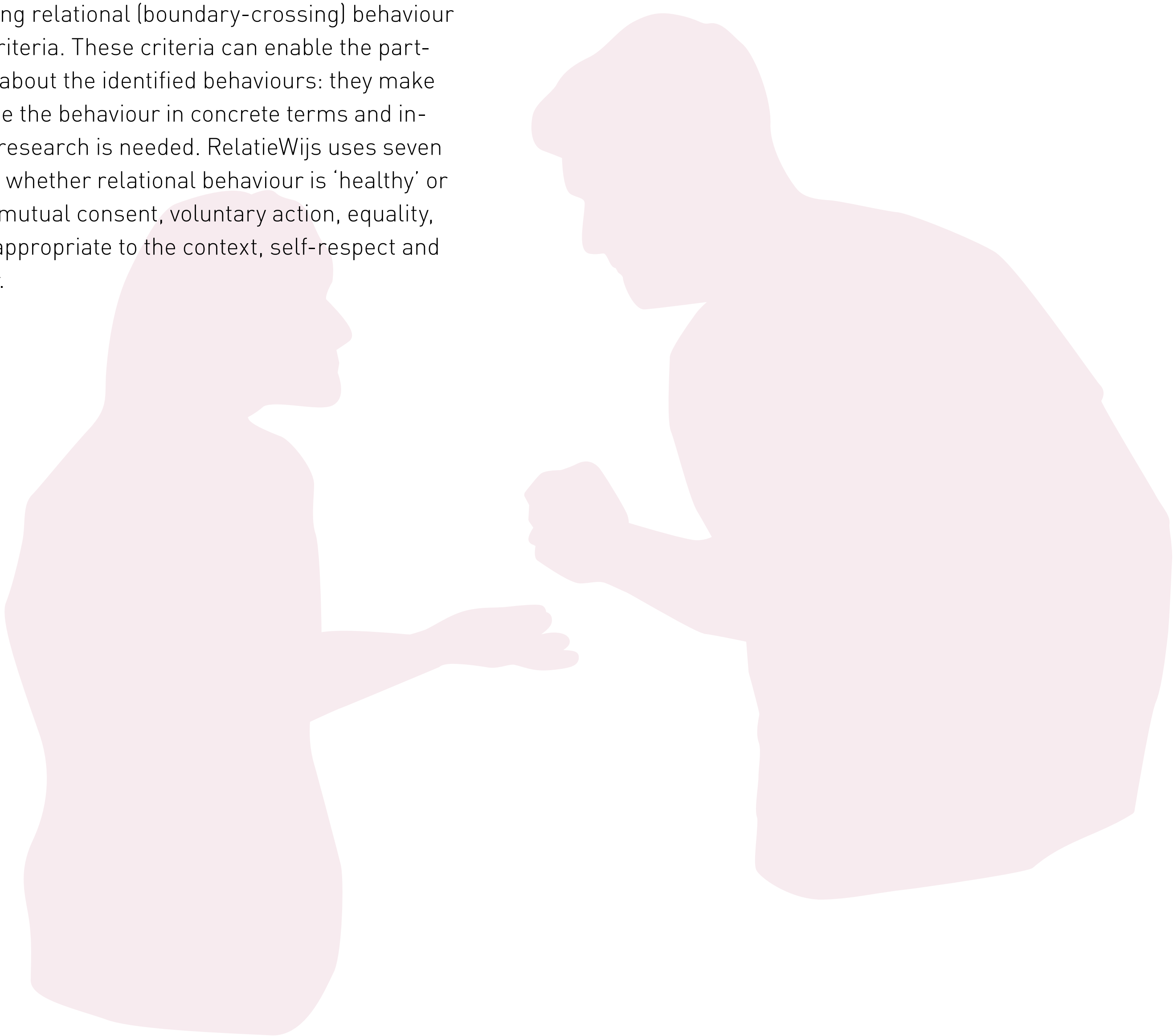

SOURCES (EX-)PARTNER VIOLENCE

ORGANISATIONS INVOLVED

The following organisations were involved in making this fact sheet:

- Movisie. For questions and/or remarks about the fact sheet, please email the main author: Wilma Schakenraad, w.schakenraad@movisie.nl
- Danielle van den Heuvel – Sterk Huis
- Suzanne Tan – Bureau Tangram
- Willemijn Krebbekx – Atria
- Karin van Rosmalen-Nooijens – Radboud UMC
- Marga Nicolaij – Veilig Thuis
- Mireille Bartelomij – Veilig Thuis

SOURCES

The following documents and other sources provide more information about the topic of this fact sheet:

- Daru, S., J. Mejdoubi, K. de Vaan en A. Visser (2016), Huiselijk geweld verklaard vanuit genderperspectief: Literatuurstudie. Amsterdam/Utrecht: Atria, Movisie en Regioplan.
- Janssen, H., Wentzel, W., & Vissers, B. (2015). Basisboek huiselijk geweld: signaleren, melden en aanpakken. 3e herz. druk. Bussum: Couthinho.
- Janssens, K., Visser, A., & Oosten, N. van (2017). RelatieWijs. Beoordelen en aanpakken van (ex-) partnergeweld. Utrecht: Movisie.
- Römkens, R., de Jong, T., & Harthoorn, H. (2014). Geweld tegen vrouwen. Europese onderzoeksgegevens in Nederlandse context. Amsterdam: Atria.
- R. Römkens, A. van den Brink en T. de Jong, (2018 Welk geweld telt? Opvattingen van Nederlanders over partnergeweld. Amsterdam: Atria, kennisinstituut voor emancipatie en vrouwengeschiedenis en Blijf Groep 2018.
- Steketee, M., R. Römkens, T. Pels, K. Lünnemann, E. Smits van Waesberghe, J. Mak, J. Mejdoubi & H. Harthoorn (2016). Preventie van intergenerationeel geweld Nederland en EU. Verkenning van wat werkt. Kennisinstituut voor emancipatie en vrouwengeschiedenis: Atria en Utrecht: Verweij-Jonker Instituut.
- Veen, H.C.J. van der, & Bogaerts, S. (2010). Huiselijk geweld in Nederland: overkoepelend synthese-rapport van het vangst-hervangst-, slachtoffer- en daderonderzoek 2007-2010. Den Haag, WODC.
- Verwijs, R., & Lünnemann, K. (2012). Partnergeweld. Achtergrond en risicofactoren. Utrecht: Verwey-Jonker Instituut.
- Signalenkaarten van Kadera, zie signalenkaart.nl/, These contain signals that can indicate domestic violence, both among victims and perpetrators. The signalling map is divided by age with risk factors in eight areas. One of the maps is aimed at adult offenders

CHILD ABUSE

ALWAYS USE THE REPORTING CODE WHEN YOU ENCOUNTER A FORM OF (DOMESTIC) VIOLENCE, ABUSE, NEGLECT OR EXPLOITATION! ▶

This fact sheet is part of a series about *(domestic) violence, abuse, neglect, exploitation* and other types of harm that may be inflicted onto someone in a power-imbalanced relationship. Power-imbalanced relationships can exist with anyone, for example: an (ex-)partner, a child, a parent, a sibling, another family member, an informal or a professional carer, a friend, a flatmate or neighbour, a teacher, a colleague or supervisor, or just someone you know. These fact sheets describe different types of harm that can be inflicted in these relationships. They are meant as an add-on to the Dutch Reporting Code for these issues (English version here) and were developed for two reasons: 1) To provide professionals with an overview of all the types of harm that exist, to aid them in identifying both well-known and lesser-known types (see the Overview). 2) Signs/indicators may vary greatly by type of harm and certain types of harm require specific courses of action; the fact sheets help professionals with identifying the signs/indicators and risk factors of *each specific type* of harm and with acting appropriately when they do. Note: the general 5 steps in the Reporting Code are applicable to all types of harm in power-imbalanced relationships; the factsheets provide more guidance within these 5 steps – they are an add-on, not a replacement.

Below is a brief introduction to the topic of child abuse, an overview of the signs/indicators and risk factors associated with this type of violence, and points of focus for when you encounter it.

WHAT IS CHILD ABUSE?

The Youth Law in the Netherlands defines child abuse as: 'Any form of threatening or violent interaction of a physical, psychological or sexual nature that is actively or passively imposed on the minor by the parents or other persons towards whom the minor is in a relationship of dependence or unfreedom, causing or threatening to cause serious mental or physical harm to the minor'.

SHAPES

Child abuse takes various forms, such as physical abuse, physical neglect, psychological abuse, psychological neglect, sexual abuse (also online), and witnessing (partner) violence. Specific forms of physical abuse include shaken baby syndrom, pediatric condition falsification and female genital mutilation.

ASSESSING SAFETY AND RISKS

Information about risks and protective factors is summarised in the various Child Abuse Guidelines that exist in the Netherlands.

A few points of attention:

- Risk factors increase the risk of child abuse.
- The more risk factors, the greater the risk of the occurrence or recurrence of child abuse.
- The main risk factors pertain to parental characteristics and the living conditions of the family.
- Partner violence is an important risk factor.

Various instruments are available to help to make a risk assessment. In practice, there sometimes appear to be misunderstandings about risk assessment. Read more about this in the publication 'Veiligheid en risico's inschatten: wat helpt'.

FACTS AND FIGURES

- According to the National Abuse Prevalence Study (NPM), it is estimated that in **2017** between **90.000 and 127.000 children and young people aged 0 to 18** were exposed to some form of child abuse **in the Netherlands**. This means **3 percent** of all children.
- The most common forms of child abuse are emotional and physical neglect, with 36 and 24 percent of cases respectively.
- More figures from the National Prevalence Study can be found here.
- In the **Children's Abuse Survey 2016,** almost **25 percent of** secondary school students (grades 1-4) say that they have ever **been victims** of child abuse in life. This corresponds with data from research into adverse childhood experiences (ACEs) among pupils in grades 7 and 8 of primary education.

ADVICE / REPORT

For advice, reporting and/or referral to reception and/or other help, call: Safe Home **0800 20 00**

In case of acute danger call **112**

DUTCH TRANSLATION

See here.

This fact sheet was developed by Nederlands Jeugdinstituut (NJi) in collaboration with various chain partners and experts.

CHILD ABUSE

DIFFERENCE BETWEEN ACUTE AND STRUCTURAL UNSAFETY

- **Acute unsafety** includes immediate physical danger or the absence of basic care for dependent children.
- **Structural unsafety refers** to families or households where there are recurring or continuous unsafe events and situations. This structural unsafety (sometimes due to a lack of 'child signs') is not detected by many professionals.

POSSIBLE SIGNS/INDICATORS: HOW TO IDENTIFY IT

Sometimes children do not show any signs of abuse and seem to function well, despite many problems with the parents. Nevertheless, there may be a situation of unsafety in the home. There are many overviews of signs of child abuse. Almost all signs are non-specific: not one characteristic of appearance, behaviour or development is specific to child abuse and all characteristics can also be a sign of other problems. Therefore, perhaps more important than knowledge about signs is a 'sensitivity' to them and to how to weigh them.

Professionals working with adults should carry out the "Child Check". This is part of the reporting code and is therefore obligatory. Professionals are responsible for following the reporting code when signls and facts support a suspicion of child abuse.

POINTS OF ATTENTION WHEN GOING THROUGH THE 5 STEPS IN THE REPORTING CODE

The Dutch Reporting Code has changed as of 1 January 2019: Reporting suspicions of child abuse to Veilig Thuis ("Veilig Thuis" means "Safe at Home" in Dutch, it is the organization in the Netherlands for advice on, referrals to and reporting of any type of (domestic) violence, abuse, neglect or exploitation, or other types of harm in power-imbalanced relationships) is mandatory as of 1 January 2019 in all cases where there is a suspicion of acute and/or structural unsafety. Read more about working with the changed reporting code here.

WANT TO KNOW MORE?

- Definition and forms of child abuse (Child abuse file, NJi)
- Physical child abuse (Child abuse file, NJi)
- Child abuse guidelines (Child abuse file, NJi)
- Identifying child abuse (Child abuse file, NJi)
- Overview card of signs/indicators of domestic violence and child abuse
- Assessing safety and risks: what helps? The sense and nonsense of risk assessment-instruments
- Learning from emergencies 2 - Safety of children in vulnerable families (Inspectie Justitie en Veiligheid)
- The child check (Augeo)
- Risk factors, protective factors and signals - Recommendations (Directive: Child abuse, NCJ 2016)
- The assessment framework in the Report Code on Domestic Violence and Child Abuse (Dutch government, 2017)
- Veilig Thuis (Dutch Government)

This fact sheet was developed by Nederlands Jeugdinstituut (NJi) in collaboration with various chain partners and experts.

VULNERABLE PREGNANT WOMEN AND PROTECTION OF THE UNBORN CHILD

This fact sheet is part of a series about *(domestic) violence, abuse, neglect, exploitation* and other types of harm that may be inflicted onto someone in a power-imbalanced relationship. Power-imbalanced relationships can exist with anyone, for example: an (ex-)partner, a child, a parent, a sibling, another family member, an informal or a professional carer, a friend, a flatmate or neighbour, a teacher, a colleague or supervisor, or just someone you know. These fact sheets describe different types of harm that can be inflicted in these relationships. They are meant as an add-on to the Dutch Reporting Code for these issues (English version here) and were developed for two reasons: 1) To provide professionals with an overview of all the types of harm that exist, to aid them in identifying both well-known and lesser-known types (see the Overview). 2) Signs/indicators may vary greatly by type of harm and certain types of harm require specific courses of action; the fact sheets help professionals with identifying the signs/indicators and risk factors of *each specific type* of harm and with acting appropriately when they do. Note: the general 5 steps in the Reporting Code are applicable to all types of harm in power-imbalanced relationships; the factsheets provide more guidance within these 5 steps – they are an add-on, not a replacement.

Below is a brief introduction to the topic of vulnerable pregnant women and protection of the unborn child, an overview of the signs/indicators and risk factors associated with this type of harm, and points of attention for when you encounter it.

WHO ARE VULNERABLE PREGNANT WOMEN?

Vulnerable pregnant women have an increased risk of harm to themselves and to the unborn child, risk of poor pregnancy outcomes, and of child abuse and neglect. A bad start as a baby may affect people for the rest of their lives.

Vulnerabilities can often already be observed during the pregnancy and concern both the behaviour and lifestyle of the pregnant woman and that of the (ex-)partner or spouse.

Factors of vulnerability are for example: intimate partner violence, psychiatric diagnose or mental health issues, intellectual disability, substance abuse and substance use by the mother, young age (teenage pregnancies), or experiencing problems in several areas of life ('multi-problem' families; see Risk Factors below for a more detailed overview).

RISK FACTORS: WHO IS EXTRA VULNERABLE?

- Intimate partner violence prior to and/or during pregnancy.
- Experiences of expectant parent(s) with child abuse and neglect (including sexual abuse) in their own youth.
- Other children in the family are already under child protection or in foster care.
- Psychiatric problems, mental health issues, or (mild) intellectual disability in expectant parents.
- Depression and prior postnatal depression.
- Smoking, alcohol and drug use during pregnancy.
- Alcohol and drug abuse and other addiction in the partner / spouse.

FACTS AND FIGURES

- Of all pregnant women in the **Netherlands,** 8.9% sometimes **drink** alcohol and 0.8% sometimes **drink** more than four glasses in one day.
- Of all pregnant lower educated women, **22.1%** smoke daily throughout their pregnancy, **5.5%** of medium educated women and 0,9% high educated women do so.
- 12% of Dutch pregnant women experience **depressive disorder** during or after pregnancy
- Prevalence of teenage pregnancies including birth, in the Netherlands is approximately **1500 teenagers** (< 20 years) a year (0.9% of the total).

ADVICE/REPORTING

For advice, for reporting victims or perpetrators, and/or for referring someone to care (including shelters), call:

- Veilig Thuis ("Veilig Thuis" means "Safe at Home" in Dutch, it is the organization in the Netherlands for advice on, referrals to and reporting of any type of (domestic) violence, abuse, neglect or exploitation, or other types of harm in power-imbalanced relationships). Telephone: **0800 20 00**, free of charge and always open (24 hours per day, 7 days a week).

This fact sheet was developed by Nederlandse Vereniging voor Obstetrie en Gynaecologie (NVOG), Erasmus MC, TNO and Veilig Thuis in collaboration with various chain partners.

VULNERABLE PREGNANT WOMEN AND PROTECTION OF THE UNBORN CHILD

- Teenage pregnancies, unintentional pregnancies, low educational level and single parent (especially in co-occurrence: a group that is seen often in the prevention programme Voorzorg).
- Babies who are born prematurely or with a low birth weight are statistically more likely to be born into vulnerable families.
- Negative attributions and hostility towards the unborn child and/or of previous children.
- Late onset in maternity health care, non-compliance with therapy and advice, frequent cancellations of appointments or not appearing, insufficient self-care.
- Problems and stress in multiple areas of life: housing, financial problems and debts, unemployment, no insurance, crime and delinquency, family violence, no or non-helpful social support.

GUIDING DOCUMENTS FOR VARIOUS GROUPS OF PROFESSIONALS IN IDENTIFICATION/SIGNALLING

- **Midwifery/obstetricians:** interview and ALPHA-NL, R4U or Mind2Care.
- **Maternity carers:** use the TNO Early Warning Checklist during the maternity period.
- **Youth public health care:** screen during regular contact moments and/or based on (Pre)SPARK, GIZ, SamenStarten.
- **Everyone:** use the 'Child Check', discuss your concerns with the expectant parents, discuss your concerns in multidisciplinary consultations, consult with Veilig Thuis.

REFERRAL DURING PREGNANCY

Low threshold:

- Prenatal Home visits by a Youth Public Health Care nurse or by the 'Stevig Ouderschap' programme (prenatally)
- Social neighbourhood team (practical help in all areas of life, light parenting support, access to specialist help)
- Relationship therapy (via GP or social neighbourhood team)
- Other agreements made in the Maternity Health Care Collaboration (VSV)

Specific:

- The 'Voorzorg' programme
- (Youth) mental healthcare, addiction care, perpetrator programmes
- POP-clinic (Psychiatry, Obstetrics and Paediatrics): maternity health care in relation to psychiatry
- Forms of home visitation, various programmes for teenage mothers, parents with (mild) intellectual disabilities, parents with psychiatric problems.

POINTS OF ATTENTION WHEN GOING THROUGH THE 5 STEPS IN THE REPORTING CODE

For any form of (domestic) violence, abuse, neglect or exploitation, professionals in the Netherlands are required to use the Reporting Code. For general reporting code guidelines (such as the 5 steps in this code) visit the link; these are not described in this fact sheet. We do describe here points of attention in going through the 5 steps that are specific to the topic of this fact sheet.

- It is possible to call anonymously and/or to call for advice or information only, without reporting someone.

In case of acute danger call the emergency services at the phone number **112**.

DUTCH TRANSLATION

See here.

This fact sheet was developed by Nederlandse Vereniging voor Obstetrie en Gynaecologie (NVOG), Erasmus MC, TNO and Veilig Thuis in collaboration with various chain partners.

VULNERABLE PREGNANT WOMEN AND PROTECTION OF THE UNBORN CHILD

These are:

- Unborn children can be put under supervision by a social worker throughout pregnancy. Child protection can then give instructions such as assisting with obstetric check-ups and other forms of care.
- In the case of suicidality and addiction to hard drugs, forced admission to a care facility is possible on the basis of the BOPZ Act. In the event of incapacity in making decisions, mentorship or guardianship can be requested from the court.
- You can always ask advice at Veilig Thuis, anonymously if you wish, ask for the resident doctor. Acute and structural threat to the unborn child and not cooperating with care, are reasons to file a report to Veilig Thuis.

MORE INFORMATION

See the Sources and:

- www.fiom.nl
- www.tienermoeders.nl

This fact sheet was developed by Nederlandse Vereniging voor Obstetrie en Gynaecologie (NVOG), Erasmus MC, TNO and Veilig Thuis in collaboration with various chain partners.

SOURCES VULNERABLE PREGNANT WOMEN AND PROTECTION OF THE UNBORN CHILD

ORGANISATIONS INVOLVED

The following organisations were involved in making this fact sheet:

- Veilig Thuis, Anne-Marie Raat
- TNO, Remy Vink, remy.vink@tno.nl
- Erasmus MC / Dutch Association for Obstetrics and Gynaecology (NVOG), Hans J. Duvekot

SOURCES

The following documents and other sources provide more information about the topic of this fact sheet:

websites

- Veilig Thuis
- www.voorzorg.info
- www.tienermoeders.nl
- www.nunietzwanger.nl
- www.RSJ.nl (advies prenatale kinderbescherming en de rol van de overheid, juni 2015)
- www.kinderbescherming.nl (Informatieblad Bescherming nog niet geboren kinderen en voorkomen kwetsbare zwangerschappen. Raad voor de Kinderbescherming, januari 2017)
- www.TNO.nl (De eerste duizend dagen)
- www.NVAVG.nl (Handreiking kinderwens en anticonceptie bij mensen met een verstandelijke beperking. Oktober 2016)
- www.fiom.nl en www.siriz.nl (hulp bij onbedoelde zwangerschap)
- www.lkpz.nl (Kenniscentrum Psychiatrie en zwangerschap)
- www.trimbos.nl
- ALPHA-NL
- R4U
- Mind2Care
- TNO Checklist Vroegsignalering
- Verloskundig SamenwerkingsVerband
- (Pre)SPARK
- GIZ
- SamenStarten
- Kindcheck
- Nji: figures about teenage mothers
- TNO about smoking
- TNO on alcohol consumption

publications

- Ahmadabadi, Z. e.a. (2018) Maternal intimate partner violence victimization and child maltreatment. Child Abuse & Neglect, 23-33
- Austin, A. e.a. (2018) Using time-to-event analysis to identify preconception and prenatal predictors of child protective services contact. Child Abuse & Neglect, 83-91
- Chen, H. e.a. (2018) Prenatal smoking and post partum depression: a meta- analysis. Journal of Psychosomatic Obstetrics & Gynecology, , 1-9
- Guterman, K., (2015) Unintended pregnancy as a predictor of child maltreatment. Child Abuse & Neglect, 160-169
- Hafekost, K. e.a. (2017) Maternal alcohol use disorder and subsequent child protection contact: A record-linkage population cohort study. Child Abuse & Neglect, 206-214
- Huizink, A.C. (2013) Prenatal cannabis exposure and infant outcomes: Overview of studies. Prog Neuro-Psychopharmacol Biol Psychiatry 2013
- Lambregtse-Van den Berg, M., Kamp, I. van, Wennink, H. (red.) Handboek psychiatrie en zwangerschap. ISBN 9789058982698. Utrecht. Uitgeverij De Tijdstroom (2015)
- Mejdoubi, J., Heijkant, S.C.C.M., van den, Struijf, E., Leerdam, J.M., van, HiraSing, R.A., Crijnen, A.M. (2013) Risicofactoren voor kindermishandeling bij jonge hoogrisicozwangeren: design van het VoorZorg onderzoek. Tijdschrift voor Jeugdgezondheidszorg, pp 26–31
- Prindle, P. e.a Prenatal substance exposure diagnosed at birth and infant involvement with child protective services. Child Abuse & Neglect 2018, 75-83
- Steegers, E. (2017) Sociale verloskunde: gelijke kansen op een gezonde start. Ned Tijdschr Geneesk;161: 1-4
- Taylor, J. & A. Lazenbatt (2014) Child Maltreatment and High Risk Families, Dunedin Academic Press, Edinburgh. ISBN 978-1-78046-031-4
- VWS, Actieprogramma Kansrijke Start, september 2018. Den Haag. Ministerie van Volksgezondheid, Welzijn en Sport
- Wall-Wieler, E. e.a. (2018) Predictors of having a first child taken into care at birth: a population based retrospective cohort study. Child Abuse & Neglect, 1-9
- Wewerinke, A., Honig, A., Heres, M.H.B., en Wennink, J.M.B. Psychiatrische stoornissen bij zwangeren en kraamvrouwen. Ned Tijdschr Geneeskd. 2006;150:294-8.

This fact sheet was developed by Nederlandse Vereniging voor Obstetrie en Gynaecologie (NVOG), Erasmus MC, TNO and Veilig Thuis in collaboration with various chain partners.

FEMALE GENITAL MUTILATION

ALWAYS USE THE REPORTING CODE WHEN YOU ENCOUNTER A FORM OF (DOMESTIC) VIOLENCE, ABUSE, NEGLECT OR EXPLOITATION! ▶

This fact sheet is part of a series about *(domestic) violence, abuse, neglect, exploitation* and other types of harm that may be inflicted onto someone in a power-imbalanced relationship. Power-imbalanced relationships can exist with anyone, for example: an (ex-)partner, a child, a parent, a sibling, another family member, an informal or a professional carer, a friend, a flatmate or neighbour, a teacher, a colleague or supervisor, or just someone you know. These fact sheets describe different types of harm that can be inflicted in these relationships. They are meant as an add-on to the Dutch Reporting Code for these issues (English version here) and were developed for two reasons: 1) To provide professionals with an overview of all the types of harm that exist, to aid them in identifying both well-known and lesser-known types (see the Overview). 2) Signs/indicators may vary greatly by type of harm and certain types of harm require specific courses of action; the fact sheets help professionals with identifying the signs/indicators and risk factors of *each specific type* of harm and with acting appropriately when they do. Note: the general 5 steps in the Reporting Code are applicable to all types of harm in power-imbalanced relationships; the factsheets provide more guidance within these 5 steps – they are an add-on, not a replacement.

Below is a brief introduction to the topic of female genital mutilation, an overview of the signs/indicators and risk factors associated with this type of harm, and points of attention for when you encounter it.

WHAT IS FEMALE GENITAL MUTILATION?

Female genital mutilation (FGM) is a practice involving the partial or total removal of the external female genital organs while there is no medical necessity. There are various ways in which FGM can be performed. The specific method used depends on the local traditions in the country of origin, the wishes of the parent(s), and the cutter, the person performing the practice. In the Netherlands, FGM is prevalent among certain groups of migrants. Most women and girls have been circumcised in their country of origin but now live in the Netherlands (see Facts and figures). The World Health Organization (WHO) distinguishes four types of FGM. For more information about the different types, their consequences and complaints, see the brochure "Focalpoint meisjesbesnijdenis". FGM is punishable in the Netherlands (as in many other countries) and is a form of child abuse.

POSSIBLE SIGNS/INDICATORS: HOW TO IDENTIFY IT

Signs that may indicate *a recently performed* FGM are:

- The girl has been sick during the holidays.
- The girl has been absent from school.
- The girl looks tired/exhausted.
- She goes to the toilet for long periods of time.
- She complains about abdominal pain.
- She cannot concentrate well.

FACTS AND FIGURES

Prevalence of FGM in the Netherlands

On 1 January 2018, in the Netherlands, there are an estimated 41,000 women who have undergone FGM. This represents 43% of all 95,000 migrants living in the Netherlands who are from countries where FGM is a cultural custom. Most of these women (82%) come from Somalia, Egypt, Ethiopia, Eritrea, Sudan or Iraq. For more information about other countries where FGM is prevalent, see the FGM prevalence world map by Pharos.

Risk of FGM in the Netherlands

In the Netherlands there are 38,000 girls aged 0-19 with at least one parent from a country where FGM is prevalent. Of these girls, 4200 run the risk of being circumcised in the next 20 years. This risk is especially high if their parents choose to continue the traditions of their country of origin and if preventive measures do not reach them. The risk is influenced by the length of the period of residence in the Netherlands, social pressure, knowledge about legislation, knowledge of the (health) risks of FGM, and people's attitude towards FGM.

This fact sheet was developed by Pharos in collaboration with various chain partners in the fight against female genital mutilation.

FEMALE GENITAL MUTILATION

- She is quiet and withdrawn.
- She reacts closed or is distant.
- She avoids health checks.
- She cannot do any physical exercise for a period of time.
- She has difficulty walking.

RISK FACTORS: WHO IS EXTRA VULNERABLE?

Signs that may indicate that FGM *could happen*:

- There are rumours circulating about a girl undergoing FGM.
- A holiday abroad is planned, to the country of origin.
- There are family members or relatives who have undergone FGM.
- People from high-risk countries who have only been in the Netherlands for a brief period of time and have limited knowledge about Dutch legislation on FGM.
- The family experiences pressure from relatives and/or the community to carry out FGM.
- The girl carefully speaks about the a planned FGM.

POINTS OF ATTENTION WHEN GOING THROUGH THE 5 STEPS IN THE REPORTING CODE

For any form of (domestic) violence, abuse, neglect or exploitation, professionals in the Netherlands are required to use the Reporting Code. For general reporting code guidelines (such as the 5 steps in this code) visit the link; these are not described in this fact sheet. Here, we do describe points of attention in going through the 5 steps that are specific to the topic of this fact sheet.

For a discussion with the person(s) suspected of involvement in a potential FGM-case, the following points of attention are essential:

- Show understanding for the person, the culture and the situation in a safe atmosphere.
- Invest time and effort to build a relationship of trust with the person(s) involved. Consider the possibility of using a professionally trained FGM ambassador. Consult FSAN for more information on working with trained FGM ambassadors.
- Provide clarity and explanation about the consequences and everyone's role and responsibilities in the process.
- Because FGM is a form of child abuse, the Reporting Code (English version here) applies. If you suspect a girl has recently undergone FGM or is at risk of undergoing FGM, you have to report this to Veilig Thuis (see Advice/reporting). At Veilig Thuis there are people specially trained on the topic of FGM, commonly the physicians.
- Be aware of your own views and feelings during a conversation and discuss these, when appropriate.
- In case of health problems after FGM, you can refer a girl or woman to one of the consultation hours where nurses and physicians work who are specialised in FGM.

MORE INFORMATION

See the Sources and the fact sheet on honour-based violence. The following 2 documents are particularly useful:

- Appendix 1 in the reporting code for honour-based violence
- The protocol for dealing with FGM among people <18 years of age

ADVICE/REPORTING

For advice, for reporting victims or perpetrators, and/or for referring someone to care (including shelters), call:

- Veilig Thuis ("Veilig Thuis" means "Safe at Home" in Dutch, it is the organization in the Netherlands for advice on, referrals to and reporting of any type of (domestic) violence, abuse, neglect or exploitation, or other types of harm in power-imbalanced relationships). Telephone: **0800 20 00**, free of charge and always open (24 hours per day, 7 days a week). It is possible to call anonymously and/or to call for advice or information only, without reporting someone.
- Pharos Focal Point VGV (advice only)

In case of acute danger call the emergency services at the phone number **112**.

DUTCH TRANSLATION

See here.

This fact sheet was developed by Pharos in collaboration with various chain partners in the fight against female genital mutilation.

SOURCES FEMALE GENITAL MUTILATION

NOTES

- Consult FSAN for collaboration with trained FGM ambassadors.
- In case of health problems after FGM, you can refer a girl or woman to one of the consultation hours where specialised professionals work. Go to this website for an overview: www.pharos.nl/nl/kenniscentrum/meisjes-besnijdenis/focal-point-meisjesbesnijdenis/spreekuren

ORGANISATIONS INVOLVED

The following organisations were involved in making this fact sheet:

- Landelijk Expertisecentrum Gezondheidsverschillen Pharos. For questions and/or remarks about the fact sheet, please email the main authors: Yodit Jacob, Y.Jacob@pharos.nl and Diana Geraci, D.Geraci@pharos.nl
- Knooppunt huwelijksdwang en achterlating, Diny Flierman
- Radboud umc, Karin van Rosmalen-Nooijens
- Fier - expertise en behandelcentrum op het terrein van geweld in afhankelijkheidsrelaties, Anke van Dijke
- Bureau Tangram, Suzanne Tan
- GGD GHOR, Annette Duenk en Sandra Hamming
- LEC EGG, Jeanine Janssen
- Veilig Thuis, Juliette Heetman

SOURCES

The following documents and other sources provide more information about the topic of this fact sheet:

- Factsheet Vrouwelijke Genitale Verminking. PHAROS, Utrecht, 2016. www.pharos.nl/documents/doc/factsheet_vgv.pdf
- Factsheet Vrouwelijke Genitale Verminking en de Nederlandse ketenaanpak. PHAROS, Utrecht, 2016.
- www.pharos.nl/documents/doc/factsheet-vgv-nederlandse_ketenaanpak.pdf
- Focal point meisjesbesnijdenis. PHAROS, Utrecht 2017 www.pharos.nl/documents/doc/webshop/vgvfolder-2017.pdf
- Handelingsprotocol Vrouwelijke Genitale Verminking bij minderjarigen: Uitleg en handvatten bij aanpak VGV voor Veilig Thuis, Raad voor de Kinderbescherming en Politie. PHAROS, Utrecht, 2016. www.pharos.nl/documents/doc/pharos-handelingsprotocol_vrouwelijke_genitale_verminking_bij_minderjarigen.pdf
- Factsheet: De Meldcode bij (vermoedens van) eergerelateerd geweld. Hilde Bakker en Oka Storms. MOVISIE, Utrecht, 2014. Bijlage 1 : Handelen bij (vermoedens van) meisjesbesnijdenis. P14 en volgende. www.huiselijkgeweld.nl/doc/publicaties/Meldcode_bij_vermoedens_van_eergerelateerd_geweld.pdf
- Richtlijn: Kindermishandeling (2016), thema 13 Vrouwelijke Genitale Verminking (VGV). NCJ. www.ncj.nl/richtlijnen/alle-richtlijnen/richtlijn/?richtlijn=12&rlpag=1643
- Richtlijn: Checklist Eer gerelateerd geweld www.politie.nl/themas/eergerelateerd-geweld-voor-professionals.html
- Vrouwelijke genitale verminking in Nederland, omvang, risico's en determinanten. www.pharos.nl/documents/doc/webshop/vrouwelijkegenitaleverminkinginnederland.pdf
- Vrouwelijke Genitale Verminking Omvang en risico in Nederland. www.pharos.nl/kennisbank/vrouwelijke-genitale-verminking-omvang-en-risico-in-nederland/
- NVOG, AJN, KAMG, KNMG, KNOV, LHV, NHG, NVK, NVPC, NVU, NVVS, VVAK, Pharos (2010). Leidraad. Medische zorg voor vrouwen en meisjes met vrouwelijke genitale verminking (VGV), 2019. NVOG. www.pharos.nl/wp-content/uploads/2018/10/Leidraad-Medische-zorg-voor-vrouwen-en-meisjes-met-vrouwelijke-genitale-verminking.pdf
- Pijpers, F.I.M., M. Exterkate en M. de Jager (2010) Standpunt Preventie van Vrouwelijke Genitale Verminking (VGV) door de Jeugdgezondheidszorg. Centrum Jeugdgezondheid (RIVM).
- www.pharos.nl/wp-content/uploads/2020/07/The-prevalence-and-risk-of-Female-Genital-Mutilation-among-migrant-women-and-girls-in-the-Netherlands_journal.pone_.0230919.pdf

GIRLS <18 YEARS OF AGE WHO SELL SEX

ALWAYS USE THE REPORTING CODE WHEN YOU ENCOUNTER A FORM OF (DOMESTIC) VIOLENCE, ABUSE, NEGLECT OR EXPLOITATION! ▶

This fact sheet is part of a series about *(domestic) violence, abuse, neglect, exploitation* and other types of harm that may be inflicted onto someone in a power-imbalanced relationship. Power-imbalanced relationships can exist with anyone, for example: an (ex-)partner, a child, a parent, a sibling, another family member, an informal or a professional carer, a friend, a flatmate or neighbour, a teacher, a colleague or supervisor, or just someone you know. These fact sheets describe different types of harm that can be inflicted in these relationships. They are meant as an add-on to the Dutch Reporting Code for these issues (English version here) and were developed for two reasons: 1) To provide professionals with an overview of all the types of harm that exist, to aid them in identifying both well-known and lesser-known types (see the Overview). 2) Signs/indicators may vary greatly by type of harm and certain types of harm require specific courses of action; the fact sheets help professionals with identifying the signs/indicators and risk factors of *each specific type* of harm and with acting appropriately when they do. Note: the general 5 steps in the Reporting Code are applicable to all types of harm in power-imbalanced relationships; the factsheets provide more guidance within these 5 steps – they are an add-on, not a replacement.

Below is a brief introduction to this topic, an overview of the signs/indicators and risk factors associated with this type of harm, and points of attention for when you encounter it.

DEFINITION AND WHY IS THERE A SPECIFIC FACT SHEET FOR GIRLS?

Different recommendations apply to boys <18 years of age who sell sex (see the boys' fact sheet) and girls <18 years who do so; therefore, it was decided to develop two separate fact sheets about these groups. Girls' <18 years who sell sex, also referred to as 'youth prostitution', 'youth sex work' or 'transactional sex among girls', is the performance of sexual acts by girls under the age of 18 years for money, goods or some other counter compensation (Movisie 2009). This includes acts referred to as 'sex work' as well as 'transactional sex'. When girls have paid sex, it does not *have* to be human trafficking (which usually involves a third person who benefits financially from the girl selling sex), but it *can* be. Sex with girls or boys under the age of 18 years may qualify as a sex offence depending on the circumstances (see *Facts and figures*) in the Netherlands. Paid sex with girls or boys under the age of 18 years is always a criminal offence.

POSSIBLE SIGNS/INDICATORS: HOW TO IDENTIFY IT

- **External and physical condition.** Looks overly groomed, suddenly looks challenging and sexy, changes in clothing and makeup.
- **Behaviour (general).** Suddenly has a lot of money/costly stuff, avoids talking about sex work or projects it on others, possibly drugs and/or alcohol abuse.

FACTS AND FIGURES

The law

In the Netherlands, buying sex services from people under the age of 18 years is illegal. A law is currently being drafted to increase this age to 21 years. Anyone who has sex with or buys from a minor runs the risk of being convicted. Sex with someone under the age of 16 is often punishable also without payment. It is the client's responsibility to check whether the person selling sex is 18 years old. Situations where minors sell sex do not always constitute trafficking in human beings, in which case there is usually a third person who facilitates the minor selling sex and who benefits financially from the transaction.

Size

Little is still known about the number of girls < 18 years who sell sex. Minors who sell sex often do so in secret. These young people are difficult to reach for example for social workers.

This fact sheet was developed by SHOP Den Haag in collaboration with various chain partners.

GIRLS <18 YEARS OF AGE WHO SELL SEX

- **Sexual behavioural signals.** A lot of knowledge in the field of sexuality, sudden change in behaviour, blurring of norms with regard to sexuality, having multiple sexual partners / contacts.
- **Behaviour and attitude towards others.** Demonstrates socially desirable behaviour (to avoid creating distrust), shows totally different behaviour in different places (e.g. at school or in the presence of friends), there are problems at home and at school.
- **Physical signals/health complaints.** Pain in genitals, bloody genitals, other vaginal and genital complaints, sexually transmitted diseases, cystitis and unwanted pregnancy.

For more information about these signs see this website.

RISK FACTORS: WHO IS EXTRA VULNERABLE TO START SELLING SEX <18 YEARS OF AGE?

Girls run an extra risk when dealing with:

- **Problems related to puberty:** conflicts with parents, wrestling with excessive protection, meeting wrong friends.
- **Great influenceability:** as a result of a mental disability, self-image.
- **(Serious) trauma:** as a result of neglect, abuse or (sexual) abuse.
- **Multi-problem situations:** problems in developing trusting relationships with others, evictions, problems at school, low self-image and/or poor social network.
- **Unaccompanied minor refugee:** lacking support from parents and family. (Source: Pharos)

POINTS OF ATTENTION WHEN GOING THROUGH THE 5 STEPS IN THE REPORTING CODE

- For any form of (domestic) violence, abuse, neglect or exploitation, professionals in the Netherlands are required to use the Reporting Code. For general reporting code guidelines (such as the 5 steps in this code) visit the link; these are not described in this fact sheet. We do describe here points of attention that are specific to the topic of this fact sheet. These are:
- Use the Ris-L. This helps with the identification and screening of possible human trafficking.
- Contact an expert in this field, within your organization or at other organizations (see Advice/reporting). Also contact an expert in the region.
- Enter the conversation openly and without judgment, be reliable.
- Make sure that the girl gets tested for cystitis, STIs and knows how to work safely.
- Record steps and/or concerns in your file on the girl (e.g. medical or educational).

MORE INFORMATION

See this roadmap and the Sources.

ADVICE/REPORTING

For advice, for reporting victims or perpetrators, and/or for referring someone to care (including shelters), call:

- Veilig Thuis ("Veilig Thuis" means "Safe at Home" in Dutch, it is the organization in the Netherlands for advice on, referrals to and reporting of any type of (domestic) violence, abuse, neglect or exploitation, or other types of harm in power-imbalanced relationships). Telephone: **0800 20 00**, free of charge and always open (24 hours per day, 7 days a week). It is possible to call anonymously and/or to call for advice or information only, without reporting someone.
- CoMensha 033 44 81 186
- a domestic trafficking hotline

In case of acute danger call the emergency services at the phone number **112**.

DUTCH TRANSLATION

See here.

This fact sheet was developed by SHOP Den Haag in collaboration with various chain partners. september 2020

SOURCES GIRLS <18 YEARS WHO SELL SEX

ORGANISATIONS INVOLVED

The following organisations were involved in making this fact sheet:

- SHOP Den Haag. For questions and/or remarks about the fact sheet, please email the main author: Nicole Harms, n.harms@shop-denhaag.nl
- CoMensha, het landelijk coördinatiecentrum tegen mensenhandel
- Expertisecentrum Mensenhandel en (jeugd)Prostitutie
- Augeo
- Movisie
- Bureau Nationaal Rapporteur Mensenhandel en Seksueel Geweld tegen Kinderen
- GGD GHOR Nederland
- GGD haaglanden
- Lumens
- Veilig Thuis

SOURCES

The following documents and other sources provide more information about the topic of this fact sheet:

- www.nji.nl/nl/Download-NJi/Hoe-signaleer-je-slachtoffers-Stappenplan-voor-professionals.pdf
- www.nji.nl/nl/Download-NJi/Publicatie-NJi/Actieplan-Hun-verleden-is-niet-hun-toekomst.pdf
- www.movisie.nl/publicatie/factsheet-meisjesprostitutie
- www.shop-jeugd-denhaag.nl/signalenlijst
- www.nji.nl/nl/Download-NJi/Hoe-signaleer-je-slachtoffers-Stappenplan-voor-professionals.pdf
- www.pharos.nl/nl/kenniscentrum/gezond-opgroeien/kindermishandeling/kennisdossier-kindermishandeling/exploitatie/jeugdprostitutie

BOYS <18 YEARS OF AGE WHO SELL SEX

ALWAYS USE THE REPORTING CODE WHEN YOU ENCOUNTER A FORM OF (DOMESTIC) VIOLENCE, ABUSE, NEGLECT OR EXPLOITATION! ▶

This fact sheet is part of a series about *(domestic) violence, abuse, neglect, exploitation* and other types of harm that may be inflicted onto someone in a power-imbalanced relationship. Power-imbalanced relationships can exist with anyone, for example: an (ex-)partner, a child, a parent, a sibling, another family member, an informal or a professional carer, a friend, a flatmate or neighbour, a teacher, a colleague or supervisor, or just someone you know. These fact sheets describe different types of harm that can be inflicted in these relationships. They are meant as an add-on to the Dutch Reporting Code for these issues (English version here) and were developed for two reasons: 1) To provide professionals with an overview of all the types of harm that exist, to aid them in identifying both well-known and lesser-known types (see the Overview). 2) Signs/indicators may vary greatly by type of harm and certain types of harm require specific courses of action; the fact sheets help professionals with identifying the signs/indicators and risk factors of *each specific type* of harm and with acting appropriately when they do. Note: the general 5 steps in the Reporting Code are applicable to all types of harm in power-imbalanced relationships; the factsheets provide more guidance within these 5 steps – they are an add-on, not a replacement.

Below is a brief introduction to this topic, an overview of the signs/indicators and risk factors associated with this type of harm, and points of attention for when you encounter it.

WHO/WHAT ARE BOYS <18 YEARS WHO SELL SEX?

Boys <18 years of age who sell sex is a form of youth prostitution. In the Netherlands, boy prostitution is understood to mean the provision of sexual services by men or boys in exchange for financial or material compensation. Prostitution is legal in the Netherlands. However, the following cases are punishable legally:

- When someone buys sex from someone who has not yet reached the age of 18 years.
- When someone forces another person (18- or 18+) to work as a prostitute.

There are different subgroups, and consequently different signs/indicators and recommendations, of boys <18 years of age who sell sex as compared to girls who do so. Therefore, two separate fact sheets were developed about these groups as part of this series (see also fact sheet on girls <18 years of age who sell sex).

POSSIBLE SIGNS/INDICATORS: HOW TO IDENTIFY IT

- Vague explanations for injuries
- Retreats/is closed off
- Is silent
- Isolation from family / friends + dependency on someone else
- Suddenly a lot of money / other clothes
- Extortion (e.g. with movies made of someone performing sexual acts)

FACTS AND FIGURES

- There are no precise figures on the number of boy prostitutes. It is estimated that 1500 boys "work" every day.
- Taboos (on homosexuality, on paid sex and on victimization of boys) make it more difficult for boys to ask for help.
- Male victims of exploitation are more likely to turn to perpetrators (from victimhood).
- Younger boys (14-16yrs) are asked more than older ones (18+).
- Not all boy prostitutes are homosexual.
- Boy prostitutes have a relatively higher chance of contracting STDs!

For organisations involved, see www.wegwijzermensenhandel.nl.

MORE INFORMATION

See the Sources and "Boy prostitution in view").

This fact sheet was developed by Expertisecentrum Mensenhandel en (Jeugd)Prostitutie in collaboration with various chain partners.

BOYS <18 YEARS OF AGE WHO SELL SEX

- Drives around, is picked up and brought by car (by new/unknown person)
- Psychosomatic complaints
- Demonstrates socially desirable behaviour
- Appears to have secrets
- Fiercely "anti homosexual" / suddenly very "overtly" homosexual
- Threats (from the victim himself or from family members)
- Drug/alcohol addiction: there is more often substance abuse than with girls <18 years who sell sex
- The boy has multiple mobile phones
- Gets a lot of phone calls (his whereabouts are checked by someone)
- Is present at locations that may be associated with prostitution
- Has anal complaints / other physical complaints that are vaguely explained
- Behavioural changes: has totally different behaviour in different places (school/friends/home)
- The boy's day and night rhythm is disturbed
- He has concentration problems
- Deteriorating school performance

RISK FACTORS: WHO IS EXTRA VULNERABLE?

Phase problems/puberty, identity issues, highly impressionable boys/mild mental disability, traumatised boys, boys from multi-problem families, boys with a non-western-migration background (honour-based violence), (undocumented) asylum seekers, boys who are young, and boys who live in two different worlds (see "Boy prostitution in view").

POINTS OF ATTENTION WHEN GOING THROUGH THE 5 STEPS IN THE REPORTING CODE

For any form of (domestic) violence, abuse, neglect or exploitation, professionals in the Netherlands are required to use the Reporting Code. For general reporting code guidelines (such as the 5 steps in this code) visit the link; these are not described in this fact sheet. We do describe here points of attention in going through the 5 steps that are specific to the topic of this fact sheet. These are:

- Youth prostitution is sometimes voluntary and sometimes not. When it is not, and someone is exploited/trafficked, remember that perpetrators (human traffickers) may be strangers, but are also often family members, friends or partners!
- Speak to someone alone!
- Pay extra attention to your own safety, that of the victim and his family.
- Non-Dutch boys often have little knowledge of their rights and of the reliability of the police.
- The boys – even when they are exploited – do not always see themselves as victims. Therefore, they need a special approach that is non-judgmental and non-stigmatizing.

ADVICE/REPORTING

For advice, for reporting victims or perpetrators, and/or for referring someone to care (including shelters), call:

- Veilig Thuis ("Veilig Thuis" means "Safe at Home" in Dutch, it is the organization in the Netherlands for advice on, referrals to and reporting of any type of (domestic) violence, abuse, neglect or exploitation, or other types of harm in power-imbalanced relationships). Telephone: **0800 20 00**, free of charge and always open (24 hours per day, 7 days a week). It is possible to call anonymously and/or to call for advice or information only, without reporting someone.
- CoMensha **033 44 81 186**
- a regional reporting point for domestic trafficking

In case of acute danger call the emergency services at the phone number **112**.

DUTCH TRANSLATION

See here.

This fact sheet was developed by Expertisecentrum Mensenhandel en (Jeugd)Prostitutie in collaboration with various chain partners.

THE CHILD CHECK

SIGNALLING CHILD ABUSE BASED ON PARENTAL SIGNALS

ALWAYS USE THE REPORTING CODE WHEN YOU ENCOUNTER A FORM OF (DOMESTIC) VIOLENCE, ABUSE, NEGLECT OR EXPLOITATION! ▶

This fact sheet is part of a series about *(domestic) violence, abuse, neglect, exploitation* and other types of harm that may be inflicted onto someone in a power-imbalanced relationship. Power-imbalanced relationships can exist with anyone, for example: an (ex-)partner, a child, a parent, a sibling, another family member, an informal or a professional carer, a friend, a flatmate or neighbour, a teacher, a colleague or supervisor, or just someone you know. These fact sheets describe different types of harm that can be inflicted in these relationships. They are meant as an add-on to the Dutch Reporting Code for these issues (English version here) and were developed for two reasons: 1) To provide professionals with an overview of all the types of harm that exist, to aid them in identifying both well-known and lesser-known types (see the Overview). 2) Signs/indicators may vary greatly by type of harm and certain types of harm require specific courses of action; the fact sheets help professionals with identifying the signs/indicators and risk factors of *each specific type* of harm and with acting appropriately when they do. Note: the general 5 steps in the Reporting Code are applicable to all types of harm in power-imbalanced relationships; the factsheets provide more guidance within these 5 steps – they are an add-on, not a replacement.

Below is a brief introduction to this topic, an overview of the signs/indicators and risk factors associated with this type of harm, and points of attention for when you encounter it.

WHAT IS THE CHILD CHECK?

The 'Child Check' is part of the Dutch domestic violence and child abuse Reporting Code (English version here). The aim of the Child Check is to identify more children at serious risk of abuse or neglect.

The Child Check is especially meant for people who work with adult clients/patients, such as (family) doctors, nurses, social workers, psychiatrists and psychologists. The Child Check means that in your contacts with adult clients/patients you check whether there are children involved and assess whether they are safe.

SIGNS/INDICATORS: WHICH PARENTAL SIGNS/ INDICATORS TO LOOK OUT FOR

For example, you may perform the Child Check for adult clients/ patients:

- with serious psychological problems
- following a suicide attempt
- with an addiction to drugs and/or alcohol
- who are victims or perpetrators of domestic violence
- who are very aggressive and/or dangerous to firearms
- who show signs of severe personal neglect and/or have no permanent residence or domicile
- who are mentally disabled

FACTS AND FIGURES

Research on the Child Check shows that in 91% of the cases when the Child Check gives a positive result, there is a form of child abuse. Three quarters of these children were not yet known to Veilig Thuis (Diderich et al., 2013).
By identifying child abuse also based on **parental signals** instead of only on **child signals,** help and support can be organized for a family at an earlier stage. Since 2013, the Child Check is a mandatory part of the Dutch Reporting Code.
Children of parents with a mental disorder and/or an addiction carry an increased risk of neglect because parents, as a result of personal problems, are insufficiently able to provide needed care for their child (basic care, emotional and affective support).

MORE INFORMATION

See the Sources and:

- Augeo. The Child Check.
- Augeo magazine. The Child Check for doctors: signals from parents

This fact sheet was developed by De Kindcheck in collaboration with various chain partners. september 2020

THE CHILD CHECK

SIGNALLING CHILD ABUSE BASED ON PARENTAL SIGNALS

It is also important to perform the Child Check when an adult is being held by the police/judiciary.

POINTS OF ATTENTION WHEN GOING THROUGH THE 5 STEPS IN THE REPORTING CODE

For any form of (domestic) violence, abuse, neglect or exploitation, professionals in the Netherlands are required to use the Reporting Code. For general reporting code guidelines (such as the 5 steps in this code) visit the link; these are not described in this fact sheet. We do describe here points of attention in going through the 5 steps that are specific to the topic of this fact sheet. These are:

- The Child Check should be performed as part of step 1 in the Reporting Code when mapping *signs/indicators*.
- If you have any doubts about the safety of children who are part of the adult's e.g. family, for example because the adult who you are in contact with has a limited social network, or because there is no other parent – then go through the steps of the Reporting Code (English version here). It is important to firstly denote the reasons why you doubt the safety of the children, based on which signs/indicators.
- When the Child Check turns out positive (i.e. concerns about the safety of the children persist after step 4 in the Reporting Code), ALWAYS report to Veilig Thuis. Then, in consultation with Veilig Thuis, you can discuss how you can best organize safety for the children.
- **Important**: To ensure that the Child Check is always performed when there are concerns about adult clients/patients, it is important to include standard Child Check questions in the clients/patients file. This way, the Child Check cannot be forgotten and colleagues can retrace steps taken earlier.

ADVICE/REPORTING

For advice, for reporting victims or perpetrators, and/or for referring someone to care (including shelters), call:

- Veilig Thuis ("Veilig Thuis" means "Safe at Home" in Dutch, it is the organization in the Netherlands for advice on, referrals to and reporting of any type of (domestic) violence, abuse, neglect or exploitation, or other types of harm in power-imbalanced relationships). Telephone: **0800 20 00**, free of charge and always open (24 hours per day, 7 days a week). It is possible to call anonymously and/or to call for advice or information only, without reporting someone.

In case of acute danger call the emergency services at the phone number **112**.

DUTCH TRANSLATION

See here.

This fact sheet was developed by De Kindcheck in collaboration with various chain partners. september 2020

SOURCES THE CHILD CHECK

ORGANISATIONS INVOLVED

The following organisations were involved in making this fact sheet:

- De Kindcheck. For questions and/or remarks about the fact sheet, please email the main author: Hester Diderich, h.diderich@kindcheck-ggz.nl
- Veilig Thuis, Wanda Lansbergen
- Radboud umc, Karin van Rosmalen-Nooijens
- SIEN, voor mensen met een verstandelijke beperking, Jolanda den Hartog
- Augeo

SOURCES

The following documents and other sources provide more information about the topic of this fact sheet:

- Diderich et al., 2013
- Augeo. De Kindcheck.
- Augeo magazine. De Kindcheck voor medici: signalen van ouders

CHILDREN IN A HIGH-CONFLICT DIVORCE

ALWAYS USE THE REPORTING CODE WHEN YOU ENCOUNTER A FORM OF (DOMESTIC) VIOLENCE, ABUSE, NEGLECT OR EXPLOITATION! ▶

This fact sheet is part of a series about *(domestic) violence, abuse, neglect, exploitation* and other types of harm that may be inflicted onto someone in a power-imbalanced relationship. Power-imbalanced relationships can exist with anyone, for example: an (ex-)partner, a child, a parent, a sibling, another family member, an informal or a professional carer, a friend, a flatmate or neighbour, a teacher, a colleague or supervisor, or just someone you know. These fact sheets describe different types of harm that can be inflicted in these relationships. They are meant as an add-on to the Dutch Reporting Code for these issues (English version here) and were developed for two reasons: 1) To provide professionals with an overview of all the types of harm that exist, to aid them in identifying both well-known and lesser-known types (see the Overview). 2) Signs/indicators may vary greatly by type of harm and certain types of harm require specific courses of action; the fact sheets help professionals with identifying the signs/indicators and risk factors of *each specific type* of harm and with acting appropriately when they do. Note: the general 5 steps in the Reporting Code are applicable to all types of harm in power-imbalanced relationships; the factsheets provide more guidance within these 5 steps – they are an add-on, not a replacement.

Below is a brief introduction to this topic, an overview of the signs/indicators and risk factors associated with this type of harm, and points of attention for when you encounter it

WHAT IS A HIGH-CONFLICT DIVORCE?

We speak of a high-conflict divorce when children are hampered by violent and complex conflicts between parents, and have become the focus of the struggle between parents. In this regard, the type of contract or bond that existed between the parents does not matter (see the Sources for a more detailed definition).

Every separation puts the family system under pressure. Parents also suffer from a high-conflict divorce: they may experience feelings of shame and guilt, and feel like they are failing as parents. Parents no longer take up their parental position. In addition, parents in separations are more vulnerable to the development of psychological and addiction problems, to which their children are exposed.

Consequences of a high-conflict divorce for children can include:

- Psychological complaints, such as anxiety, depression and post-traumatic stress.
- Emotional neglect by one or both parents (resulting in loneliness).
- Loyalty conflicts: parents make opposite appeals to the child. Children have the feeling that they cannot/must not be loyal to both parents, which leads to internal conflicts.

FACTS AND FIGURES

- A high-conflict divorce is also called a complex separation or a "fight-separation" ("vechtscheiding") in Dutch.
- In 2017 there were 18,178 separations involving children under the age of 18 years in the Netherlands. A separation always affects a child, the consequences of which vary according to age.
- Every year about 3500 children are involved in a high-conflict divorce in the Netherlands.
- About half of these separations relate to unmarried partners, which makes it more difficult to identify this group (but not less relevant! For the involved children this makes no difference).
- In 2014, the Children's Ombudsman of the Netherlands estimated that at that time approximately 16,000 children in the Netherlands were suffering from the effects of a high-conflict divorce.
- About 50% of the children in a high-conflict divorce develop post-traumatic stress symptoms. About a third of the children in a high-conflict divorce continue to have complaints.

This fact sheet was developed by Radboudumc, afdeling eerstelijnsgeneeskunde, gender in transmural care in collaboration with various chain partners. september 2020

CHILDREN IN A HIGH-CONFLICT DIVORCE

- School and development problems:
  - internalising behaviour problems, e.g. reclusive behaviour
  - externalising behaviour problems, e.g. difficulty with authority, problems in dealing with peers, deteriorating school performance and (sexual) risk behaviour
- Intergenerational transmission, which means that children themselves also end up in a problematic relationship.
- Parentification, where the child feels (or is made) responsible for the well-being of the parents.

POSSIBLE SIGNS/INDICATORS: HOW TO IDENTIFY IT

Children in a high-conflict divorce are always victims. The signs in children that may lead you to identify that the child is a victim of a high-conflict divorce are comparable to the signs for child abuse and domestic violence. Signs often only become visible if there are already consequences due to the separation, as described above. Therefore, when there is a separation and children are involved → always be alert.

A specific and latent sign of a high-conflict divorce is parental rejection or parental alienation, in which the child completely rejects one of the parents and, as it were, takes sides.

RISK FACTORS: WHO IS EXTRA VULNERABLE?

Every child with divorcing parents has a chance to end up in a high-conflict divorce and develop problems as a result. Nevertheless, there are some factors that may increase the risks of developing problems in children whose parents are in a high-conflict divorce:

- Pre-existing and/or other violence and child abuse.
- Addiction or psychological or psychiatric problems in (one of) the divorcing parents.
- Psychological violence in the family, in particular psychological warfare between the parents; serious and prolonged parental conflicts.
- If the child has a bad relationship with (one of) the parent(s) or step-parent(s).
- Changes such as relocation, school change and new partners.
- Financial problems (generally decline in SES and in particular when there is poverty)

POINTS OF ATTENTION WHEN GOING THROUGH THE 5 STEPS IN THE REPORTING CODE

For any form of (domestic) violence, abuse, neglect or exploitation, professionals in the Netherlands are required to use the Reporting Code. For general reporting code guidelines (such as the 5 steps in this code) visit the link; these are not described in this fact sheet. We do describe here points of attention in going through the 5 steps that are specific to the topic of this fact sheet. These are:

There are many different interests involved in high-conflict divorces. Parents often underestimate the impact on children.

- Talk to the parents. For example, you can tell the parents what you see in the child or what you see them do.
- Talk to the child. Children process the separation better if they have people around them who make them feel they are not alone and explain to them what is going on at their own level. This also includes the use of a Child Representative.

MORE INFORMATION

See the Sources. Also read the Fact sheet about the Child Check for related information. Additional information:

- www.nji.nl
- www.richtlijnenjeugdhulp.nl
- www.vooreenveiligthuis.nl

ADVICE/REPORTING

For advice, for reporting victims or perpetrators, and/or for referring someone to care (including shelters), call:

- Veilig Thuis ("Veilig Thuis" means "Safe at Home" in Dutch, it is the organization in the Netherlands for advice on, referrals to and reporting of any type of (domestic) violence, abuse, neglect or exploitation, or other types of harm in power-imbalanced relationships). Telephone: **0800 20 00**, free of charge and always open (24 hours per day, 7 days a week). It is possible to call anonymously and/or to call for advice or information only, without reporting someone.

In case of acute danger call the emergency services at the phone number **112**.

DUTCH TRANSLATION

See here.

This fact sheet was developed by Radboudumc, afdeling eerstelijnsgeneeskunde, gender in transmural care in collaboration with various chain partners. september 2020

CHILDREN IN A HIGH-CONFLICT DIVORCE

- A high-conflict divorce is a form of child abuse. Therefore, consult with Veilig Thuis for advice (see Advice/reporting).
- Both parents with parental authority are entitled to information about their child up to 16 years of age, but this is not a right to information about the other parent. As a counsellor, it is extra important to remain neutral and to keep information clear but concise.
- Social workers can keep an eye on the interests of both parents and children. The best results are achieved by a cooperation between the legal and the social workers. Then, attention is paid to the welfare of both parents and children, and agreement is also reached on the legal conflict points with mediators, lawyers and judges.
- There are several recognised interventions that may help in separations. It is also good to take note of what works. For parents, easily accessible information can be useful, for example with the new Separation ATLAS. Children can also be supported preventively with group programmes, for example.

This fact sheet was developed by Radboudumc, afdeling eerstelijnsgeneeskunde, gender in transmural care in collaboration with various chain partners.

SOURCES CHILDREN IN A CONFLICT SEPARATION

ADDITIONAL INFORMATION

Different definitions of a 'high-conflict separation' are in use. We use the following definition:
There is **a high-conflict separation** when communication between parents about parenting and visitation rights is conflictual, as a result of which the children cannot maintain relaxed contact with one or both parents for a long time, experience suffering, pressure or parentification, and/or the child's development is threatened. It happens regularly that parents deny that there is a high-conflict separation.

High-conflict separations may be accompanied by:

- **Psychological abuse**
  For example: Parents do things that are mentally and/or emotionally offensive to the other parent as witnessed by the children (swearing, threatening, manipulating, disqualifying, humiliating, belittling or bullying); parents take a negative view of the other parent in presence of the children; children may not maintain contact with the other parent (parental rejection)
- **Pedagogical neglect**
  For example: There is uncertainty and disagreement about the way in which parents wish to care for and raise their children. Parents do not agree on the assistance that is necessary to eliminate insecurity for the child or to support the development of the child.
- **Witness to domestic violence**
  For example: Children witness conflicts, which may be accompanied by physical violence, between parents. There are many different ways of exposure: ranging from direct exposure (direct witnessing through seeing or hearing violence) to indirect exposure (seeing and experiencing the consequences of violence, such as a blue eye or the tension in the home).

ORGANISATIONS INVOLVED

The following organisations were involved in making this fact sheet:

- Radboudumc, afdeling eerstelijnsgeneeskunde, gender in transmural care. For questions and/or remarks about the fact sheet, please email the main author: Karin van Rosmalen-Nooijens, Karin.vanRosmalen-Nooijens@radboudumc.nl
- HMC Westeinde, Hesther Diderich
- TNO, Fieke Pannebakker
- GGD GHOR Nederland, Sandra Hamming
- Augeo, Marga Haagmans
- Sterk Huis, Cindy de Rijke

Sources

The following documents and other sources provide more information about the topic of this fact sheet:

- www.nji.nl/Mogelijke-effecten-van-echtscheiding-op-het-kind
- www.nji.nl/Scheiding-Praktijk-Erkende-interventies
- www.nji.nl/Scheiding-Praktijk-Wat-werkt
- www.nji.nl/wegwijzer-kind-en-scheiding
- www.rijksoverheid.nl/onderwerpen/scheiden/vraag-en-antwoord/kind-bij-vechtscheiding
- www.rijksoverheid.nl/documenten/rapporten/2018/02/22/rapport-scheiden...en-de-kinderen-dan
- hetlock.nl/wp-content/uploads/2017/03/Vechtscheidingen-Belevingen-en-ervaringen-van-ouders-en-kinderen-en-veranderingen-na-Kinderen-uit-de-knel.pdf
- www.kinderbescherming.nl/themas/g/gezag-en-omgang/innovaties-voor-behandeling-conflictscheidingen
- www.dekinderombudsman.nl/ul/cms/fck-uploaded/KOM003.2014Kinderombudsmanadviesrapportvechtscheidingen.pdf
- richtlijnenjeugdhulp.nl/scheiding/
- www.villapinedo.nl/
- vooreenveiligthuis.nl/ik-maak-me-zorgen-om-iemand/ik-ben-ouder-dan-18-jaar/ik-maak-me-zorgen-om-een-kind-in-een-vechtscheiding/
- kindbehartiger.nl
- www.tno.nl/atlas

This fact sheet was developed by Radboudumc, afdeling eerstelijnsgeneeskunde, gender in transmural care in collaboration with various chain partners.

MALE ABUSE

ALWAYS USE THE REPORTING CODE WHEN YOU ENCOUNTER A FORM OF (DOMESTIC) VIOLENCE, ABUSE, NEGLECT OR EXPLOITATION! ▶

This fact sheet is part of a series about *(domestic) violence, abuse, neglect, exploitation* and other types of harm that may be inflicted onto someone in a power-imbalanced relationship. Power-imbalanced relationships can exist with anyone, for example: an (ex-)partner, a child, a parent, a sibling, another family member, an informal or a professional carer, a friend, a flatmate or neighbour, a teacher, a colleague or supervisor, or just someone you know. These fact sheets describe different types of harm that can be inflicted in these relationships. They are meant as an add-on to the Dutch Reporting Code for these issues (English version here) and were developed for two reasons: 1) To provide professionals with an overview of all the types of harm that exist, to aid them in identifying both well-known and lesser-known types (see the Overview). 2) Signs/indicators may vary greatly by type of harm and certain types of harm require specific courses of action; the fact sheets help professionals with identifying the signs/indicators and risk factors of *each specific type* of harm and with acting appropriately when they do. Note: the general 5 steps in the Reporting Code are applicable to all types of harm in power-imbalanced relationships; the factsheets provide more guidance within these 5 steps – they are an add-on, not a replacement.

Below is a brief introduction to this topic, an overview of the signs/indicators and risk factors associated with this type of harm, and points of attention for when you encounter it

WHAT IS MALE ABUSE?

Domestic violence against men – commonly referred to as male abuse ("mannenmishandeling") in the Netherlands – concerns any type of (domestic) violence, abuse, neglect, exploitation or other type of harm that may be inflicted onto men as part of a power-imbalanced relationship. This fact sheet focuses on (ex-)partner violence against men. See also the fact sheet on (ex-) partner violence in general. It is estimated that in 40% of the domestic violence cases, there is (also) male abuse. Approximately 80,000 men are severely abused in domestic settings every year.

(Ex-)partner violence aimed at men has many similarities with domestic violence as described in the fact sheet (ex)-partner violence. There are also differences, which must be taken into account in the identification and approach:

- **The perception of men:** Male abuse is not always seen as a problem by the men concerned, nor as a form of domestic violence. Furthermore, these men often feel that there are few, if any, fellow sufferers – that they are the only ones o experience it. Only 3% of men report domestic violence to the police.
- **Social images and norms:** Think of ideas like: "A man does not let himself be beaten (and certainly not by a woman)".

MORE INFORMATION

See the Sources and:

- www.mannenmishandeling.nl
- www.huiselijkgeweld.nl

ADVICE/REPORTING

For advice, for reporting victims or perpetrators, and/or for referring someone to care (including shelters), call:

- Veilig Thuis ("Veilig Thuis" means "Safe at Home" in Dutch, it is the organization in the Netherlands for advice on, referrals to and reporting of any type of (domestic) violence, abuse, neglect or exploitation, or other types of harm in power-imbalanced relationships). Telephone: **0800 20 00**, free of charge and always open (24 hours per day, 7 days a week). It is possible to call anonymously and/or to call for advice or information only, without reporting someone.

In case of acute danger call the emergency services at the phone number **112**.

In the Netherlands there are six shelter locations specifically for men (they have space for 40 men in total).

DUTCH TRANSLATION

See here.

This fact sheet was developed by Federatie Opvang and Stichting LOS in collaboration with various chain partners.

- **The feeling of shame is very big:** Because of this, men who suffer violence or abuse are less likely to come forward, talk less about it and ask less for help. This shame is also nourished by the aforementioned social images and norms.

ATTENTION FOR THE SPECIFIC TARGET GROUP: LHBTI+

Domestic violence occurs in all relationships, whether heterosexual, homosexual, bisexual or other. Domestic violence under LHBTI+ is also a big taboo. British research shows that one in four homosexuals is a victim of partner violence. People often feel that violence in partner relationships between gays and lesbians is less severe or less serious, because both partners are more or less equal in strength and power ("the other can strike back or defend him-/herself"). Heterosexuals also often assume that homosexuals and lesbians will break off a relationship more quickly than heterosexuals if they are subject to domestic violence. Most heterosexuals do not see economic dependency and raising children together as core concepts of gay and lesbian relationships. Therefore, they assume there are less binding factors for homosexual or lesbian partners. The opposite is true. Research shows that homosexual men endure years of violence in their relationships precisely because they strive for a long-term stable love-relationship.

POSSIBLE SIGNS/INDICATORS: HOW TO IDENTIFY IT

The signs/indicators of male abuse are the same as the signs/indicators described in the fact sheet on (ex)-partner violence and there-mentioned lists of signs/indicators.

RISK FACTORS: WHO IS EXTRA VULNERABLE?

A number of factors can increase the risk of (recurrence of) domestic violence against men:

- **Demographic factors:** age, upbringing, personality, poverty and housing. For example, the risk of abuse is higher if the socio-economic status is lower.
- **Being a witness:** Men who have witnessed or been victims of domestic violence or child abuse in childhood are at increased risk of becoming victims again in adulthood.
- **Dynamic risk factors:** substance use, psychological problems, stress, relationship/family problems. Financial stress is one of the most common reasons for domestic partner violence. Migration stress is also one of the risk factors, as well as when a man comes to the Netherlands from abroad to get married.
- **Static risk factors:** this concerns the man's personal problems, such as a mild intellectual disability.
- **Conditional factors:** for example, social isolation or divorce problems.
- **Societal context:** The extent to which violence towards men is normalised and/or portrayed as less threatening from a social and/or media perspective.
- **Problems with the man's partner:** limited aggression control, addiction, personality problems, difficulty communicating or with conflict management, jealousy and/or a tendency to control.

This fact sheet was developed by Federatie Opvang and Stichting LOS in collaboration with various chain partners.

MALE ABUSE

POINTS OF ATTENTION WHEN GOING THROUGH THE 5 STEPS IN THE REPORTING CODE

For any form of (domestic) violence, abuse, neglect or exploitation, professionals in the Netherlands are required to use the Reporting Code. For general reporting code guidelines (such as the 5 steps in this code) visit the link; these are not described in this fact sheet. We do describe here points of attention in going through the 5 steps that are specific to the topic of this fact sheet. These are:

- **Gender-specific presentation:** In general it can be said that from a gender-specific perspective men have different ways of seeking help, presenting their problems and dealing with problems. There is also another experience of the problem. Men only seek help when they can no longer solve the problem themselves, they seek solutions themselves and try them out, they look for causes of problems outside themselves, they describe problems from an external spectator's point of view, they present problems in the context of work and they articulate problems in cases and facts.
- **Condemnation and societal norms:** Social and health workers too may (unconsciously, unintentionally) condemn or adhere to certain societal norms, which can hinder assistance provided.
- **Taboo:** Because of the taboo around this topic, men will not easily consider themselves to be a victim, for example.
- **Taking control:** Men who have been abused often benefit from retaining as much control as possible over their recovery process. Try to understand how men deal with their problems.

This fact sheet was developed by Federatie Opvang and Stichting LOS in collaboration with various chain partners. september 2020

SOURCES MALE ABUSE

ORGANISATIONS INVOLVED

The following organisations were involved in making this fact sheet:

- Blijf Groep, Stichting Wende en Veilig Thuis. For questions and/or remarks about the fact sheet, please email the main authors: Claire Loeber, C.Loeber@blijfgroep.nl , Carla Scherpenhuijzen, c.scherpenhuijzen@perspektief.nl , Floor van Niekerk, en Ries Wilschut, RWilschut@samen-veilig.nl
- Robert Weinberg, Blijf groep

SOURCES

The following documents and other sources provide more information about the topic of this fact sheet:

- Nanhoe, A., *Pionieren in de mannenopvang, Een evaluatiestudie na 2½ jaar ervaring met de opvang en hulpverlening aan mannelijk slachtoffers van (dreiging van) geweld in afhankelijkheidsrelaties in Amsterdam, Rotterdam, Den Haag en Utrecht*, Rijksoverheid, Gemeente Amsterdam, Gemeente Rotterdam, Gemeente Den Haag, Gemeente Utrecht (Uitgevoerd door GGD Rotterdam-Rijnmond), 2011.
- Adrie Vermeulen, Necla Kilic, Bert Visser, Magda Vogelezang, *'Als man heb je al gauw de schijn tegen' Beschrijving expertise Pilot G4 Mannenopvang Huiselijk geweld, Eergerelateerd geweld & Mensenhandel*, 2014.
- Van Dijk, D., Hoekstra, L., & Nieuwenhout, Y. (2010). Als de nood aan de man is: Een verkennende studie naar de opvang van en hulpverlening aan mannelijke slachtoffers van (dreiging van) geweld in afhankelijkheidsrelaties. *Rotterdam: Sociale Zaken en Werkgelegenheid.*
- Janssen, J. H. L. J., & Sanberg, R. (2013). Als de nood aan de man komt. Slachtofferschap van mannen bij eergerelateerd geweld.
- Ligtenberg, D. (2018). Mijn Leven, geslagen man het taboe op mannenmishandeling. Libelle, 33, 74-78.
- Oosten, van, N, Visser, A., Hazebroek, L., Daru, s. (2015). Dossier: wat werkt bij partnergeweld. Utrecht: Movisie.
- Renzetti, C. M., & Miley, C. H. (2014). *Violence in gay and lesbian domestic partnerships*. Routledge.
- Römkens, R. (2010). Omstreden gelijkheid. *B. Den Haag, Huiselijk geweld*, 11-32.
- Schuyf, J. (2009). *Geweld tegen homoseksuele mannen en lesbische vrouwen.* Movisie.
- Tsui, V., Cheung, M., & Leung, P. (2010). Help-seeking among male victims of partner abuse: men's hard times. *Journal of community psychology*, 38(6), 769-780.
- Yanez, P. C. (2018). *A Treatment Model for Male Victims of Domestic Violence: A Support Group for Men with Abusive Partners* (Doctoral dissertation, The Chicago School of Professional Psychology).
- www.mannenmishandeling.nl
- www.huiselijkgeweld.nl
- signalenkaart.nl

DOMESTIC VIOLENCE AGAINST/AMONG MIGRANTS IN VULNERABLE SITUATIONS

This fact sheet is part of a series about *(domestic) violence, abuse, neglect, exploitation* and other types of harm that may be inflicted onto someone in a power-imbalanced relationship. Power-imbalanced relationships can exist with anyone, for example: an (ex-)partner, a child, a parent, a sibling, another family member, an informal or a professional carer, a friend, a flatmate or neighbour, a teacher, a colleague or supervisor, or just someone you know. These fact sheets describe different types of harm that can be inflicted in these relationships. They are meant as an add-on to the Dutch Reporting Code for these issues (English version here) and were developed for two reasons: 1) To provide professionals with an overview of all the types of harm that exist, to aid them in identifying both well-known and lesser-known types (see the Overview). 2) Signs/indicators may vary greatly by type of harm and certain types of harm require specific courses of action; the fact sheets help professionals with identifying the signs/indicators and risk factors of *each specific type* of harm and with acting appropriately when they do. Note: the general 5 steps in the Reporting Code are applicable to all types of harm in power-imbalanced relationships; the factsheets provide more guidance within these 5 steps – they are an add-on, not a replacement.

Below is a brief introduction to this topic, an overview of the signs/indicators and risk factors associated with this type of harm, and points of attention for when you encounter it.

ALWAYS USE THE REPORTING CODE WHEN YOU ENCOUNTER A FORM OF (DOMESTIC) VIOLENCE, ABUSE, NEGLECT OR EXPLOITATION! ▶

WHY A SEPARATE FACT SHEET ON MIGRANTS IN VULNERABLE SITUATIONS?

'Migrants' is the collective term for people who, for various reasons, have moved from abroad to the Netherlands to settle here temporarily or permanently. This fact sheet specifically deals with migrants **in vulnerable situations.** These groups have an increased risk of becoming victims of domestic violence and/or for them ending a relationship may have consequences for their residency status. For this reason, a separate factsheet was developed about these groups.

This fact sheet is not about victims of human trafficking, there is a separate fact sheet about that.

The specific characteristics of a number of groups of migrants in vulnerable situations are listed below – the groups that this fact sheet deals with.

FACTS AND FIGURES

The Dutch WODC (Research and Documentation Centre) estimates that in 2015 approximately 35,000 people resided in the Netherlands without a residence permit, of whom one third were women and one half were people with an asylum application that was rejected.

According to Significant (see Sources), the number of victims of domestic violence without a residence permit could be between 70 and 800 per year. Every year, some 30-40 victims of domestic violence apply for a residence permit on the basis of being a victim of domestic violence.

MORE INFORMATION

See the Sources and:

- IND brochure on the right of residence for victims of violence in power-imbalanced relationships
- Information on women's and men's shelters: www.opvang.nl, www.opvangatlas.nl, www.mannenopvang.nl
- Information about the rights of people without a residence permit: www.basicrights.nl, www.stichtinglos.nl or www.iLegalevrouw.nl
- Information on shelters for asylum seekers

This fact sheet was developed by Federatie Opvang and Stichting LOS in collaboration with various chain partners.

DOMESTIC VIOLENCE AGAINST/AMONG MIGRANTS IN VULNERABLE SITUATIONS

Category	Characteristics
Asylum seekers	Are awaiting a decision on application for a asylum residence permit and stay in an asylum seekers' centre (AZC). COA staff of these centres are trained in detecting domestic violence and they can also call in help from Veilig Thuis and women's shelters.
Status holders	Have received a residence permit and been allocated a residence or are still staying in an AZC pending allocation of a residence. Family members can travel with status holders for the purpose of family reunification. These family members are granted a derived residence permit.
Marriage migrants / Partner migrants	Living with the partner and having a residence permit that is dependent on that partner. Only after 5 years can they obtain an independent licence.
Europeans	Can be here as a partner migrant or be here to work.
Undocumented migrants	Undocumented migrants are migrants who entered the Netherlands illegally, migrants who stayed longer than their visa granted, rejected asylum seekers, or migrants who stayed after their right of residence was terminated on other grounds. They are largely invisible, very vulnerable and highly dependent on the person who shelters and feeds them. If the government comes into contact with them, they run the risk of being arrested and deported. That is why they are afraid of contact with formal authorities and often do not know their rights. As victims of domestic violence, they do have the right to protection, medical care, help and shelter under international treaties.

ADVICE/REPORTING

For advice, for reporting victims or perpetrators, and/or for referring someone to care (including shelters), call:

- Veilig Thuis ("Veilig Thuis" means "Safe at Home" in Dutch, it is the organization in the Netherlands for advice on, referrals to and reporting of any type of (domestic) violence, abuse, neglect or exploitation, or other types of harm in power-imbalanced relationships). Telephone: **0800 20 00**, free of charge and always open (24 hours per day, 7 days a week). It is possible to call anonymously and/or to call for advice or information only, without reporting someone.

In case of acute danger call the emergency services at the phone number **112**.

For advice about undocumented migrants, their rights and advice about available assistance, call:

- Stichting LOS **010 74 70 156**

DUTCH TRANSLATION

See here.

This fact sheet was developed by Federatie Opvang and Stichting LOS in collaboration with various chain partners.

DOMESTIC VIOLENCE AGAINST/AMONG MIGRANTS IN VULNERABLE SITUATIONS

DOMESTIC VIOLENCE AGAINST/AMONG MIGRANTS IN VULNERABLE SITUATIONS

Domestic violence is violence committed by someone from the domestic or family circle. It is about violence in a power-imbalanced relationship. The resulting dependence often makes it difficult for the victim to stop or escape from the violence. Migrants in vulnerable situations are often more dependent, see "Risk factors".

According to international law (Istanbul Convention and European Directive for victims), assistance and shelter for victims of domestic violence must be accessible, regardless of the residence status.

Domestic violence and subsequent separation can affect the right of residence:

Category	Characteristics
Status holders	Refugees and any family members who accompany them on their way to the Netherlands will retain their asylum status even after divorce.
Marriage migrants / Partner migrants	In the event of domestic violence, the dependent partner is granted a temporary permit. This is only extended if return would mean a risk of human rights violations in the country of origin.
Asylum seekers	If the asylum statement is linked to the other partner, the partner may no longer be able to obtain an asylum permit in the event of divorce. If separation in the country of origin leads to a risk of human rights violations, this may be an independent reason for an asylum permit.
Europeans	People from EU countries can stay in the Netherlands if they have their own means of existence. Contact with IND is important for Europeans who claim benefits and/or shelter.
Undocumented migrants	Undocumented migrants may be granted a permit as victims of domestic violence if their return would represent a risk of human rights violations in the country of origin.

DOMESTIC VIOLENCE AGAINST/AMONG MIGRANTS IN VULNERABLE SITUATIONS

POSSIBLE SIGNS/INDICATORS: HOW TO IDENTIFY IT

In principle, the indicators of domestic violence for migrants in vulnerable situations are the same as for people with a Dutch background. However, it is particularly important for professionals working with migrants to pay attention to these signs because they are **more often hidden.** This is true especially for migrants who live in isolation, particularly undocumented migrants, who are afraid of institutions and who are not easily open about problems for cultural reasons.

RISK FACTORS: WHO IS EXTRA VULNERABLE?

Migrants in vulnerable situations may face an increased risk of domestic violence arising from various risk factors:

- Uncertainty about the rights of their residence status can make people even more dependent on partner or family and can make people reluctant to be open to authorities and other official agencies. This applies especially to people with no residency rights.
- Economic and social dependence can be high if one is not financially independent, has a low level of education and/or does not speak the language.
- Cultural aspects can play a role, for example the male-female relationships and/or reluctance to go to institutions with problems, unfamiliarity with institutions and/or shame about the situation.
- Unfamiliarity with the Dutch system of care and welfare.
- Migrants often have a more limited social network and therefore lack the protective factor that this provides.

POINTS OF ATTENTION WHEN GOING THROUGH THE 5 STEPS IN THE REPORTING CODE

For any form of (domestic) violence, abuse, neglect or exploitation, professionals in the Netherlands are required to use the Reporting Code. For general reporting code guidelines (such as the 5 steps in this code) visit the link; these are not described in this fact sheet. We do describe here points of attention in going through the 5 steps that are specific to the topic of this fact sheet. These are:

- Try to speak to the victim alone.
- Call in an interpreter if the victim has insufficient command of the Dutch language.
- Migrants with an uncertain right of residence and certainly undocumented migrants are often afraid of authorities and especially the police.
- Discuss carefully what the possibilities are to leave the situation of violence and what the possible consequences could be for the right of residence. For more information about these consequences see the Table about "Effects on right of residence".
- Often, after reporting a domestic violence situation, shelter needs to be arranged. It is important to start making arrangements for this immediately.

This fact sheet was developed by Federatie Opvang and Stichting LOS in collaboration with various chain partners.

SOURCES DOMESTIC VIOLENCE AGAINST/AMONG MIGRANTS IN VULNERABLE SITUATIONS

ORGANISATIONS INVOLVED

The following organisations were involved in making this fact sheet:

- Federatie Opvang (now Valente) and Stichting LOS. For questions and/or remarks about the fact sheet, please email the main author: Rian Ederveen, rian.ederveen@stichtinglos.nl

SOURCES

- The following documents and other sources provide more information about the topic of this fact sheet:
- Significant: Toegang tot de opvang van slachtoffers zonder eerdere verblijfsstatus, 3.11.17
- Ombudsman : Vrouwen in de knel (rapport 2017/075), 7.7.17
- KIS: Huiselijk geweld en veiligheid in asielopvangcentra
- Keygnaert, I. et al: Hidden Violence is a Silent Rape: Prevention of Sexual & Gender-Based Violence against Refugees & Asylum Seekers in Europe: a Participatory Approach Report. ICRH,Ugent, Ghent, 2008
- IND : Evaluatie gendergerelateerd vreemdelingenbeleid in Nederland, 2008
- WODC: Schattingen illegaal in Nederland verblijvende vreemdelingen 2012-2013, 2015

This fact sheet was developed by Federatie Opvang and Stichting LOS in collaboration with various chain partners.

(SEXUAL) VIOLENCE AGAINST PEOPLE WITH INTELLECTUAL DISABILITIES

This fact sheet is part of a series about *(domestic) violence, abuse, neglect, exploitation* and other types of harm that may be inflicted onto someone in a power-imbalanced relationship. Power-imbalanced relationships can exist with anyone, for example: an (ex-)partner, a child, a parent, a sibling, another family member, an informal or a professional carer, a friend, a flatmate or neighbour, a teacher, a colleague or supervisor, or just someone you know. These fact sheets describe different types of harm that can be inflicted in these relationships. They are meant as an add-on to the Dutch Reporting Code for these issues (English version here) and were developed for two reasons: 1) To provide professionals with an overview of all the types of harm that exist, to aid them in identifying both well-known and lesser-known types (see the Overview). 2) Signs/indicators may vary greatly by type of harm and certain types of harm require specific courses of action; the fact sheets help professionals with identifying the signs/indicators and risk factors of *each specific type* of harm and with acting appropriately when they do. Note: the general 5 steps in the Reporting Code are applicable to all types of harm in power-imbalanced relationships; the factsheets provide more guidance within these 5 steps – they are an add-on, not a replacement.

Below is a brief introduction to this topic, an overview of the signs/indicators and risk factors associated with this type of harm, and points of attention for when you encounter it.

ALWAYS USE THE REPORTING CODE WHEN YOU ENCOUNTER A FORM OF (DOMESTIC) VIOLENCE, ABUSE, NEGLECT OR EXPLOITATION! ▶

WHAT FORMS OF VIOLENCE ARE WE TALKING ABOUT?

Like people without intellectual disabilities, people with intellectual disabilities may be subject to any type of (domestic) violence, abuse, neglect and/or exploitation, such as financial exploitation, physical abuse, psychological and emotional abuse, sexual abuse, sexual misconduct, neglect, violation of human and civil rights, and discrimination. They may experience this anywhere. The leading factor for whether or not the violence is reported to the police or Veilig Thuis ("Veilig Thuis" means "Safe at Home" in Dutch, it is the organization in the Netherlands for advice on, referrals to and reporting of any type of (domestic) violence, abuse, neglect or exploitation, or other types of harm in power-imbalanced relationships) is the relationship between the perpetrator and the victim, not the location (it does not matter whether it is committed inside the home or elsewhere). Examples of types of harm that people with mild intellectual disabilities may be subject to are: child abuse, partner violence, abuse by informal carers, elder abuse, child-parent abuse and honour-based violence. Young girls with a (light) mental disability are especially vulnerable to human trafficking and youth sex work.

ADVICE/REPORTING

For advice on this type of harm, reporting victims or perpetrators, or referring someone to care (including shelters), call:

- Veilig Thuis. Telephone: **0800 20 00**, free of charge and always open (24 hours per day, 7 days a week). It is possible to call anonymously and/or to call for advice or information only, without reporting someone.

In case of acute danger call the emergency services at the phone number **112**.

Sexual (or other) misconduct **by professional care providers** can be reported to:

- the 'Landelijk Meldpunt Zorg' of the 'Inspectie voor Gezondheidszorg en Jeugdzorg (IGJ)' **088 120 50 20**

DUTCH TRANSLATION

See here.

MORE INFORMATION

See the Sources.

This fact sheet was developed by Movisie in collaboration with various chain partners. september 2020

(SEXUAL) VIOLENCE AGAINST PEOPLE WITH INTELLECTUAL DISABILITIES

WHO IS THE TARGET GROUP?

A disability may be congenital or not (e.g. non-Congenital Brain Injury). In addition to their intellectual disability, people in this group may also suffer from other disabilities. In that case we speak of multiple disabilities. Finally, the intellectual disability or the combination of disabilities may vary in severity. In this fact sheet we limit ourselves to people with a congenital mental disability. Because of their limitations, these people are less resilient. They are often (strongly) dependent on care and support, especially in terms of social care. Because of their intellectual disability, they are less able to properly assess social situations (EQ), their ability to understand is less (IQ), and they often have more trouble expressing themselves verbally. Setting boundaries can therefore be difficult for them. People with intellectual disabilities are also often overestimated. Partly because their limitations are less visible and they themselves hide their limitations, and partly because they have no insight into their limitations.

FACTS AND FIGURES: HOW COMMON IS (SEXUAL) VIOLENCE AGAINST PEOPLE WITH INTELLECTUAL DISABILITIES?

The increased vulnerability of people with mental disabilities to various forms of violence is confirmed in a large number of domestic and foreign studies (see, among others, Platt et al. , 2017; Van der Heijden, 2014; Hughes et al., 2011; Van Berlo et al., 2011). Violence by acquaintances (including between people with intellectual disabilities) is most common (e.g. FRA, 2015).

- The UN suspects that children with disabilities face four times more violence than children without disabilities (FRA, 2015).
- A separate problem is witnessing violence between parents or other family members.
- According to foreign research, the prevalence of abuse among people with intellectual disabilities is 42% for boys and 39% for girls, 44% for adults men and 46% for adult women (Platt et al., 2017). An earlier review shows that people with intellectual disabilities are the most vulnerable group. This review shows that 26-90% of women and 29-86% of men with intellectual disabilities have at some point in their lives experienced some form of abuse (Hughes et al., 2011).
- Van Berlo et al. (2011)'s research into sexual misconduct among people with a disability is the most recent in our country in this field. It shows that when people are more specifically asked about different forms of sexual misconduct, the reported prevalence of such misconduct increases. The life-time prevalence for adult women with a (mild) mental disability was 72% and 44% for men in this study. It also concludes that 23% of women have ever been raped compared to 7% of men. Below the age of 16, the percentages for girls are 28% and for boys 19%.
- For bullying, figures that compare bullying prevalence between pupils with and without disabilities (see 'Kennisplein handicap sector') state that handicapped pupils are bullied five times more often: 10.8% against 2.2%. Children with disabilities are bullied 2 to 3 times as often as other children. Half of the children with a disability are afraid of their peers. 82% of children and young people (7-19 years) with a learning disorder have experienced bullying.

This fact sheet was developed by Movisie in collaboration with various chain partners. september 2020

(SEXUAL) VIOLENCE AGAINST PEOPLE WITH INTELLECTUAL DISABILITIES

POSSIBLE SIGNS/INDICATORS: HOW TO IDENTIFY IT

Signs with the victim

The most important signs of physical abuse are injuries, bruises spread over the body, and a combination of old and new bone fractures. Burns also occur. In people with intellectual disabilities, even sudden behavioural changes, such as withdrawal behaviour and aggression, are signs. For sexual misconduct, it can be a sign when the victim themselves start to engage in sexual misconduct and/or the use of sexual words that are not age-appropriate. Nevertheless, identifying violence is difficult among people with intellectual disabilities. They avoid making eye contact a little more because their limitations in general and in particular because of their limitations in communication and assessment of experiences, according to the research by Van Berlo et al. (2011). People with intellectual disabilities generally have less self-confidence, so this cannot be seen as a specific indicator of violence.

Signs in the offender

Signs or indicators that someone is a perpetrator of violence are the same as for perpetrators of violence against people without disabilities.

RISK FACTORS: WHO IS EXTRA VULNERABLE?

There are risk factors for violence at victim level (severity of the disability and deviant behaviour), perpetrator level (e.g. stress, parents with a slight mental disability) and - if the person with a mental disability stays in a care institution or receives support from an institution - at the organisation level of the care institution. For example, a lack of competent staff increases the risk of (sexual) misconduct among service users and also of family violence. Also, in such institutions, the violence committed is less often identified.

POINTS OF ATTENTION WHEN GOING THROUGH THE 5 STEPS IN THE REPORTING CODE

For any form of (domestic) violence, abuse, neglect or exploitation, professionals in the Netherlands are required to use the Reporting Code. For general reporting code guidelines (such as the 5 steps in this code) visit the link; these are not described in this fact sheet. We do describe here points of attention in going through the 5 steps that are specific to the topic of this fact sheet. These are:

- Special attention should be paid to the 'Child Check' when you are dealing with parents with a (mild) mental disability and/or low literacy.

This fact sheet was developed by Movisie in collaboration with various chain partners. september 2020

ORGANISATIONS INVOLVED

The following organisations were involved in making this fact sheet:

- Movisie – for questions and/or remarks about the fact sheet, please email the main author: Nico van Oosten, N.vanOosten@movisie.nl
- Bertine Spooren – GGD Amsterdam
- Sandra Hamming – GGD GHOR Nederland
- Hilair Balsters – Vilans
- Jolanda den Hartog – SIEN
- Marijke Lammers – Lammers Advies en Training
- Rianne van Beurden – Prisma
- Susan Dijkman – Veilig Thuis Kennemerland
- Wilma Schakenraad – Movisie

SOURCES

The following documents and other sources provide more information about the topic of this fact sheet:

- Berlo, W. van, Haas. S. de., Oosten, N. van, Dijk, L. van, Brants, L., Tonnon, S., & Storms, O. (2011). Beperkt Weerbaar - Een onderzoek naar seksueel geweld bij mensen met een lichamelijke, zintuiglijke of verstandelijke beperking, Utrecht: Rutgers WPF/MOVISIE.
- Casteel, C. Martin, S.L., Smith, J.B., Gurka, K.K. en Kupper, L.L. (2008). National study of physical and sexual assault among women with disabilities. Injury Prevention; vol. 14: 87-90
- FRA (2015). Violence against children with disabilities: easy read version.
- Heijden, I. van der (2014). What works to prevent violence against women with disabilities.
- Hughes, K., Bellis, M. A., Jones, L., Wood, S., Bates, G., Eckley, L., et al. (2012). Prevalence and risk of violence against adults with disabilities: a systematic review and meta-analysis of observation-al studies. Lancet, 379(9826), 1621-1629.
- Hughes, R. B., Lund, E. M., Gabrielli, J., Powers, L. E., & Curry, M. A. (2011). Prevalence of interper-sonal violence against community-living adults with disabilities: a literature review. Rehabilitation Psy-chology, 56(4), 302-319.
- Jones, L., Bellis, M. A., Wood, S., Hughes, K., McCoy, E., Eckley, L., et al. (2012). Prevalence and risk of violence against children with disabilities: a systematic review and meta-analysis of observa-tional studies. The Lancet, 380(9845), 899-907.
- Khemka, I., Hickson, L., Reynolds, G. (2005). Evaluation of a Decision-Making Curriculum Designed to Empower Women With Mental Retardation to Resist Abuse. American Journal On Mental Retardation, Volume 110, Number 3: 193–204, May 2005.
- Krnjacki, L., Emerson, E., Llewellyn, G., Kavanagh, A.M. (2016). Prevalence and risk of violence against people with and without disabilities: findings from an Australian population-based study. Australian and New Zealand Journal of Public Health, 40:16-21.
- Marsland, D., Oakes, P., & White, C. (2007). Abuse in care? The identification of early indicators of the abuse of people with learning disabilities in residential settings. The Journal of Adult Protection, 9(4), 6-20.
- Platt, L., Powers, L., Leotti, S., Hughes, R.B., Robinson, Whelen, S., Osborn, S. Ashkenazy, E., Beers, L., Lund, E. Nicolaidis, Ch., Partnering With People With Disabilities to Address Violence
- Consortium (2017). The Role of Gender in Violence Experienced by Adults With Developmental Disa-bilities. Journal of Interpersonal Violence, Vol. 32(1) 101–129.
- Plummer, S.B., Findley, P.A. (2012). Women With Disabilities' Experience With Physical and Sexual Abuse: Review of the Literature and Implications for the Field. Trauma Violence Abuse, 13(1) 15-29.
- Robinson-Whelen, S., Hughes, R.B., Gabrielli, J., Lund, E.M., Abramson, W., Swank, P.R. (2014)
- Sobsey, D. (2005). Violence & disability. In: W. M. Nehring (Ed.), Health promotion for persons with intellectual/developmental disabilities: The state of scientific evidence. Washington, DC: American Association on Mental Retardation.
- Strand, M.L., Benzein, E., Saveman, B. (2004). Violence in the care of adult persons with intellectual disabilities. Journal of Clinical Nursing, 13, 506–514.
- Trevellion, K., Oram, S., Feder, G., Howard, L.M. (2012). Experiences of Domestic Violence and Men-tal Disorders: A Systematic Review and Meta-Analysis. PLOS ONE, December, Volume 7 Issue 12.

PARENT ABUSE

ALWAYS USE THE REPORTING CODE WHEN YOU ENCOUNTER A FORM OF (DOMESTIC) VIOLENCE, ABUSE, NEGLECT OR EXPLOITATION! ▶

This fact sheet is part of a series about *(domestic) violence, abuse, neglect, exploitation* and other types of harm that may be inflicted onto someone in a power-imbalanced relationship. Power-imbalanced relationships can exist with anyone, for example: an (ex-)partner, a child, a parent, a sibling, another family member, an informal or a professional carer, a friend, a flatmate or neighbour, a teacher, a colleague or supervisor, or just someone you know. These fact sheets describe different types of harm that can be inflicted in these relationships. They are meant as an add-on to the Dutch Reporting Code for these issues (English version here) and were developed for two reasons: 1) To provide professionals with an overview of all the types of harm that exist, to aid them in identifying both well-known and lesser-known types (see the Overview). 2) Signs/indicators may vary greatly by type of harm and certain types of harm require specific courses of action; the fact sheets help professionals with identifying the signs/indicators and risk factors of *each specific type* of harm and with acting appropriately when they do. Note: the general 5 steps in the Reporting Code are applicable to all types of harm in power-imbalanced relationships; the factsheets provide more guidance within these 5 steps – they are an add-on, not a replacement.

Below is a brief introduction to this topic, an overview of the signs/indicators and risk factors associated with this type of harm, and points of attention for when you encounter it.

WHAT IS PARENT ABUSE?

Parent abuse (or "parental abuse" or "child-to-parent abuse/violence", but not to be confused with elder abuse) is: non-incidental violence in the family which is aimed at (one of) the parents, committed by a youth or young adult, who usually lives at home and is dependent on the parent. Parent abuse concerns repeated and serious violence (not the 'normal' puberty conflicts). Violence can be psychological, physical and sexual, with financial extortion and material damage.

POSSIBLE SIGNS/INDICATORS: HOW TO IDENTIFY IT

There is a lot of shame with parents (feelings of failure). Parents usually only ask for help at an advanced stage. Possible signs:

- Parents are afraid of their own child.
- Increase in verbal violence (often around 12-14 years) and escalation to serious threat and physical violence.
- Arson, (traces of) material damage.
- Severe outbursts of anger due to triggers (e.g. alcohol, drugs, internet and gaming addiction).
- Reversal of day-night rhythm and hardly any daytime activities.
- (Serious) behavioural problems, aggression and anger in connection with psychopathology.

FACTS AND FIGURES

- It is estimated that 10% of all incidents of family violence with police involvement concerns parents as victims.
- More than 80% of the perpetrators are boys/young men.
- In two thirds of cases, the biological mother is the victim.

ADVICE/REPORTING

For advice, for reporting victims or perpetrators, and/or for referring someone to care (including shelters), call:

- Veilig Thuis ("Veilig Thuis" means "Safe at Home" in Dutch, it is the organization in the Netherlands for advice on, referrals to and reporting of any type of (domestic) violence, abuse, neglect or exploitation, or other types of harm in power-imbalanced relationships). Telephone: **0800 20 00**, free of charge and always open (24 hours per day, 7 days a week). It is possible to call anonymously and/or to call for advice or information only, without reporting someone.

In case of acute danger call the emergency services at the phone number **112**.

DUTCH TRANSLATION

See here.

This fact sheet was developed by TNO in collaboration with various chain partners. september 2020

PARENT ABUSE

- Parent(s) feel the behavioural change of their child had a sudden onset ("he/she was not like this in the past").
- Co-occurrence of family violence such as sibling abuse.
- Violence also outside the family (e.g. dating violence).
- Negative communication and interaction patterns in the family.
- Male dominance as a social norm within the family.
- Problematic behaviour with peers, truancy and long term absenteeism, suspensions, bullying or being bullied.
- Debts, financial exploitation and extortion of parents.
- Restraining orders, report(s) to the police by parents or family.
- Parents isolate themselves and feelings of exhaustion.

RISK FACTORS: WHO IS EXTRA VULNERABLE?

Little is known about risk factors for parent abuse. The general picture from both practice and scientific literature is that parent abuse is mostly committed by boys, from 14/15 years of age, living at home. Violence is mostly aimed at single (divorced) mothers of all socio-economic backgrounds. Young people with psychiatric problems, especially when this only manifests itself in puberty, with behavioural problems, low frustration tolerance, low empathy and adaptability, seem to form a specific group of perpetrators.

A disrupted parent-child interaction is also a risk factor, for example because of: a very permissive upbringing without boundaries, lack of family cohesion or extreme protection, shift in authority relationships within the family (e.g. due to divorce), family violence, unpredictability of parents.

POINTS OF ATTENTION WHEN GOING THROUGH THE 5 STEPS IN THE REPORTING CODE

For any form of (domestic) violence, abuse, neglect or exploitation, professionals in the Netherlands are required to use the Reporting Code. For general reporting code guidelines (such as the 5 steps in this code) visit the link; these are not described in this fact sheet. We do describe here points of attention that are specific to the topic of this fact sheet. These are:

- Ask parents how they are doing and how they feel, listen, ask for details and take them seriously.
- When treating a young person with a mental health problem, check whether there is aggression and remember that parent abuse is a potential consequence of that situation.
- Also pay attention to the other children in the family (as victims or perpetrators).
- In order to stop serious violence it may be necessary to call in the police (resulting in an official report or a restraining order).
- Consult Veilig Thuis about the possibility of out-of-home placement of the young person.
- Consider the following programmes: parenting support, MST, Relational Family Therapy, MDFT, Non-Violent Resistance, or an Aggression Regulation programme.

MORE INFORMATION

See the Sources and:

- The website Holes in the wall
- The database of Effective interventions for family violence and sexual violence
- This report by TNO and Movisie about parent abuse

This fact sheet was developed by TNO in collaboration with various chain partners. september 2020

SOURCES PARENT ABUSE

ORGANISATIONS INVOLVED

The following organisations were involved in making this fact sheet:

- TNO (For questions and/or remarks about the fact sheet, please email the main author Remy Vink, remy.vink@tno.nl)
- MOVISIE
- GGD GHOR Nederland
- Augeo Foundation
- Veilig Thuis
- VVAK
- Het Lorentzhuis

SOURCES

The following documents and other sources provide more information about the topic of this fact sheet:

- Beckmann, L., Bergmann, M. C., Fischer, F. and Mosel, T. (2017) Risk and Protective Factors of Child-to-Parent Violence: A Comparison Between Physical and Verbal Aggression, Journal of Interpersonal Violence, first published online 12th December 2017
- Biehal, N. (2012). Parent abuse by young people on the edge of care: a child welfare perspective. Social policy & society, 11(2),251-263.
- Calvete E, Orue I, Gamez-Guadix M, Bushman BJ. (2015) Predictors of child-to-parent aggression: A 3-year longitudinal study. Dev Psychol. 2015 May;51(5):663-76. doi: 10.1037/a0039092. Epub 2015 Mar 30.
- Cottrell, B. & Monk, P. (2004). Adolescent-to-parent abuse: A qualitative overview of common themes. J.Fam. Issues, 25(8), 1072-1095.
- Eckstein, N.J. (2004). Emergent issues in families experiencing adolescent-to-parent abuse. Western Journal of Communication, 68(4), 365-388.
- Holt, A. (2013). Adolescent-to-Parent Abuse: Current Understandings in Research, Policy and Practice. Policy Press, Bristol University.
- Holt, A. (2015) Working with Adolescent Violence and Abuse Towards Parents: Approaches and Contexts for Intervention. London. Routledge.
- Ibabe, I. & Jaureguizar, J. (2010). Child-to-parent violence: Profile of abusive adolescents and their families. Journal of Criminal Justice, 38(4), 616-624.
- Kennair, N. & Mellor, D. (2007). Parent abuse: a review. Child Psychiatry Hum. Dev., 38(3), 203-219.
- Weinblatt, U. & Omer, H. (2008). Geweldloos verzet: een behandeling voor ouders van kinderen met ernstige gedragsproblemen. Gezinstherapie Wereldwijd, 19(4): 389-418.
- Pagani, L., Larocque, D., Vitaro, F., & Tremblay, R.E. (2003). Verbal and Physical Abuse Toward Mothers: The Role of Family Configuration, Environment, and Coping Strategies. Journal of Youth and Adolescence,32(3), 215-222.
- Routt, G., & Anderson L. (2011). Adolescent violence towards parents. Journal of Aggression, Maltreatment and Trauma, 20(1), 1-18.
- Ulman, A., & Straus, M.A. (2003). Violence by children against mothers in relation to violence between parents and corporal punishment by parents. J of Comparative Family Studies 34(1), 41-60.
- Vink, R.M., Goes, A., Doornink, N., Broerse, A., Pannebakker, F., Zwan, van der R., Schakenraad, W. (2014) Huiselijk geweld door kinderen en jongeren tegen hun ouders. Utrecht/Leiden. Movisie/TNO

ELDER ABUSE AND ABUSE BY INFORMAL CARERS

ALWAYS USE THE REPORTING CODE WHEN YOU ENCOUNTER A FORM OF (DOMESTIC) VIOLENCE, ABUSE, NEGLECT OR EXPLOITATION! ▶

This fact sheet is part of a series about *(domestic) violence, abuse, neglect, exploitation* and other types of harm that may be inflicted onto someone in a power-imbalanced relationship. Power-imbalanced relationships can exist with anyone, for example: an (ex-)partner, a child, a parent, a sibling, another family member, an informal or a professional carer, a friend, a flatmate or neighbour, a teacher, a colleague or supervisor, or just someone you know. These fact sheets describe different types of harm that can be inflicted in these relationships. They are meant as an add-on to the Dutch Reporting Code for these issues (English version here) and were developed for two reasons: 1) To provide professionals with an overview of all the types of harm that exist, to aid them in identifying both well-known and lesser-known types (see the Overview). 2) Signs/indicators may vary greatly by type of harm and certain types of harm require specific courses of action; the fact sheets help professionals with identifying the signs/indicators and risk factors of *each specific type* of harm and with acting appropriately when they do. Note: the general 5 steps in the Reporting Code are applicable to all types of harm in power-imbalanced relationships; the factsheets provide more guidance within these 5 steps – they are an add-on, not a replacement.

Below is a brief introduction to this topic, an overview of the signs/indicators and risk factors associated with this type of harm, and points of attention for when you encounter it.

WHAT IS ELDER ABUSE?

Elder abuse occurs when any person who has a recurring personal or professional relationship with the older person (aged 65 or over) acts or neglects to act in such a manner that it results in physical and/or psychological and/or material harm to the older person, while there is some form of partial or total dependency on the part of the older person (a power imbalance).

In the event of abuse by informal carers, the informal carer transgresses the boundaries of good care because of stress, powerlessness, incompetence or ignorance. A characteristic of abuse by informal carers is often the lack of intent.

FACTS AND FIGURES

- Gezondheidsmonitor 2016
  This monitor (spanning 12 months) shows that psychological abuse is the most common form of elder abuse and affects 4.0% of the elderly aged 65 and over in the Netherlands, followed by financial abuse (1.1%) and neglect (0.6%).
- Regioplan 2018
  This interview study (conducted in Rotterdam, Tilburg and Boxtel) concludes that 1 in 20 elderly people aged 65 and over living at home ever experience elder abuse at one point in their lives and 1 in 50 will experience elder abuse on an annual basis. In both cases, the most commonly reported form appears to be financial abuse, followed by psychological and physical abuse.

ADVICE/REPORTING

For advice, for reporting victims or perpetrators, and/or for referring someone to care (including shelters), call:

- Veilig Thuis ("Veilig Thuis" means "Safe at Home" in Dutch, it is the organization in the Netherlands for advice on, referrals to and reporting of any type of (domestic) violence, abuse, neglect or exploitation, or other types of harm in power-imbalanced relationships).

This fact sheet was developed by Nederlandse Vereniging voor Klinische Geriatrie (NVKG) in collaboration with various chain partners. september 2020

ELDER ABUSE AND ABUSE BY INFORMAL CARERS

Telephone: **0800 20 00,** free of charge and always open (24 hours per day, 7 days a week). It is possible to call anonymously and/or to call for advice or information only, without reporting someone.
In case ofcute danger call the emergency services at the phone number 112.

MORE INFORMATION

See the Sources.

DUTCH TRANSLATION

See here.

FORMS AND POSSIBLE SIGNS/INDICATORS OF ELDER ABUSE

Forms	Possible signs/indicators: how to identify it
Physical abuse (e.g. hitting, pushing, kicking, threatening or injuring)	unexplained subcutaneous bruising, fractures, injuries of different ages, injuries do not fit with reported circumstancesa
Psychological abuse (including repeated insults and belittling, restrictions on movement, denial of visits, withholding mail)	gedragsveranderingen, depressieve- en angstklachten
Financial abuse (e.g. changing the will, selling goods without permission, unauthorized use of someone's debit or credit card, abuse of personal care budgets)	lack of standard (medical) facilities, disappearance of belongings, sudden debts
Neglect (e.g. withholding nutrition, physical care or access to medical care, affective neglect)	untreated bedsores, malnutrition, poor physical hygiene, blemishes, inadequate supervision of older people
Sexual abuse (including verbal harassment, unwanted sexual acts with or in the presence of the older person)	unexplained subcutaneous bruising in genital area, bloodstains in underwear, unexplained sexually transmitted diseases

ELDER ABUSE AND ABUSE BY INFORMAL CARERS

POSSIBLE SIGNS/INDICATORS OF ABUSE BY INFORMAL CARERS

- The carer being overburdened and frustrated, e.g. manifesting itself in boundary-transgressing behaviour towards the elderly or others
- Compassion fatigue on the part of the carer
- Refusing help or care for the elderly person, isolating the elderly person

RISK FACTORS: WHO IS EXTRA VULNERABLE?

On the part of victims:

- presence of cognitive disorders
- psychological / psychiatric disorders
- poor physical health
- functional limitations and dependency on care
- limited social network or loneliness
- lower socio-economic status

On the part of perpetrators:

- psychological / psychiatric disorders
- alcohol and substance use
- being overburdened in the informal care situation
- violence, abuse, neglect or exploitation in family history

POINTS OF ATTENTION WHEN GOING THROUGH THE 5 STEPS IN THE REPORTING CODE

For any form of (domestic) violence, abuse, neglect or exploitation, professionals in the Netherlands are required to use the Reporting Code. For general reporting code guidelines (such as the 5 steps in this code) visit the link; these are not described in this fact sheet. We do describe here points of attention in going through the 5 steps that are specific to the topic of this fact sheet. These are:

- Shame / taboo with victim and perpetrator may play a role

This fact sheet was developed by Nederlandse Vereniging voor Klinische Geriatrie (NVKG) in collaboration with various chain partners.

SOURCES ELDER ABUSE AND ABUSE BY INFORMAL CARERS

ORGANISATIONS INVOLVED

The following organisations were involved in making this fact sheet:

- Nederlandse Vereniging voor Klinische Geriatrie (NVKG) | Dutch Geriatrics Society NVKG (lead author of the factsheet on behalf of the NVKG: Ms Drs. M.E. van Houten, chairwoman of the Dutch guideline on suspected elder abuse in specialist healthcare services)
- Augeo
- GGD GHOR Nederland
- Movisie
- Veilig thuis
- Verenso

For questions and/or remarks about the fact sheet, please email info@nvkg.nl.

SOURCES

The following documents and other sources provide more information about the topic of this fact sheet:

- Actieplan 'Ouderen in veilige handen'. Brief aan de Tweede Kamer van 11 maart 2011, Kamerstukken II 2010/11, 29389, 30
- Comijs HC, Pot AM, Smit JH, et al. Elder abuse in the community: prevalence and consequences. J Am Geriatr Soc. 1998;46(7):885-8.PubMed PMID:9670877.
- Comijs, HC 1999, 'Elder mistreatment: prevalence, risk indicators and consequences', PhD, Vrije Universiteit Amsterdam.
- Gezondheidsmonitor Volwassenen en ouderen, 2016: bronnen.zorggegevens.nl/Bron?naam=Gezondheidsmonitor-Volwassenen-en-Ouderen%2C-GGD%E2%80%99en%2C-CBS-en-RIVM
- www.rijksoverheid.nl/binaries/rijksoverheid/documenten/rapporten/2015/06/15/ontspoorde-mantelzorg/ontspoorde-mantelzorg.pdf
- www.movisie.nl/sites/movisie.nl/files/publication-attachment/Factsheet%20Ouderenmishandeling%20III%20Financieel%20misbruik%20%5BMOV-287580-0.2%5D.pdf
- www.movisie.nl/sites/movisie.nl/files/publication-attachment/Factsheet%20ontspoorde%20mantelzorg%20%5BMOV-695455-1.1%5D.pdf
- www.movisie.nl/sites/movisie.nl/files/publication-attachment/Signalenkaart-Ontspoorde-Mantelzorg%20%5BMOV-458810-1.1%5D.pdf
- www.movisie.nl/sites/movisie.nl/files/publication-attachment/Factsheet-Ouderenmishandeling-Algemeen%20%5BMOV-225838-0.7%5D.pdf
- Dong X, Simon MA. Elder abuse as a risk factor for hospitalization in older persons. JAMA Intern Med. 2013;173(10):911-7. doi: 10.1001/jamainternmed.2013.238. PubMed PMID: 23567991.
- Dong XQ. Elder Abuse: Systematic Review and Implications for Practice. J Am Geriatr Soc. 2015;63(6):1214-38. doi: 10.1111/jgs.13454. Epub 2015 Jun 11. Review. PubMed PMID: 26096395.
- Handleiding ouderenmishandeling mei 2017, Samen Veilig Midden-Nederland, Utrecht
- Johannesen M, LoGiudice D. Elder abuse: a systematic review of risk factors in community-dwelling elders. Age Ageing. 2013;42(3):292-8. doi:10.1093/ageing/afs195. Epub 2013 Jan 22. Review. PubMed PMID: 23343837.
- McCausland B, Knight L, Page L, et al. A systematic review of the prevalence and odds of domestic abuse victimization among people with dementia. Int Rev Psychiatry. 2016;28(5):475-484. Epub 2016 Aug 26. PubMed PMID:27564566.
- Naughton C, J. Drennan, M.P. Treacy, A. Lafferty, I. Lyons, A. Phelan, S. Quin, O'Loughlin, A., Delaney, L. (2010) Abuse and Neglect of Older People in Ireland.
- Publicatie ouderen Veilig thuis, een beschrijving van de nieuwe werkwijze rondom ouderenmishandeling. Veilig thuis Utrecht. 2015,
- Prevalentieonderzoek naar aard en omvang van ouderenmishandeling, Regioplan, 2018
- Pillemer K, Burnes D, Riffin C, et al. Elder Abuse: Global Situation, Risk Factors, and Prevention Strategies. Gerontologist. 2016;56Suppl2:S194-205. doi: 10.1093/geront/gnw004. Review. PubMed PMID: 26994260; PubMed Central PMCID: PMC5291158.
- SCP-rapport Ouderenmishandeling in Nederland, 2015
- Yon Y, Mikton CR, Gassoumis ZD, Wilber KH. Elder abuse prevalence in community settings: a systematic review and meta-analysis. Lancet Glob Health. 2017 Feb;5(2):e147-e156. doi: 10.1016/S2214-109X(17)30006-2. Review. PubMed PMID: 28104184.

This fact sheet was developed by Nederlandse Vereniging voor Klinische Geriatrie (NVKG) in collaboration with various chain partners.

TRAFFICKING IN HUMAN BEINGS

This fact sheet is part of a series about *(domestic) violence, abuse, neglect, exploitation* and other types of harm that may be inflicted onto someone in a power-imbalanced relationship. Power-imbalanced relationships can exist with anyone, for example: an (ex-)partner, a child, a parent, a sibling, another family member, an informal or a professional carer, a friend, a flatmate or neighbour, a teacher, a colleague or supervisor, or just someone you know. These fact sheets describe different types of harm that can be inflicted in these relationships. They are meant as an add-on to the Dutch Reporting Code for these issues (English version here) and were developed for two reasons: 1) To provide professionals with an overview of all the types of harm that exist, to aid them in identifying both well-known and lesser-known types (see the Overview). 2) Signs/indicators may vary greatly by type of harm and certain types of harm require specific courses of action; the fact sheets help professionals with identifying the signs/indicators and risk factors of *each specific type* of harm and with acting appropriately when they do. Note: the general 5 steps in the Reporting Code are applicable to all types of harm in power-imbalanced relationships; the factsheets provide more guidance within these 5 steps – they are an add-on, not a replacement.

Below is a brief introduction to this topic, an overview of the signs/indicators and risk factors associated with this type of harm, and points of attention for when you encounter it.

ALWAYS USE THE REPORTING CODE WHEN YOU ENCOUNTER A FORM OF (DOMESTIC) VIOLENCE, ABUSE, NEGLECT OR EXPLOITATION! ▶

WHAT IS TRAFFICKING IN HUMAN BEINGS?

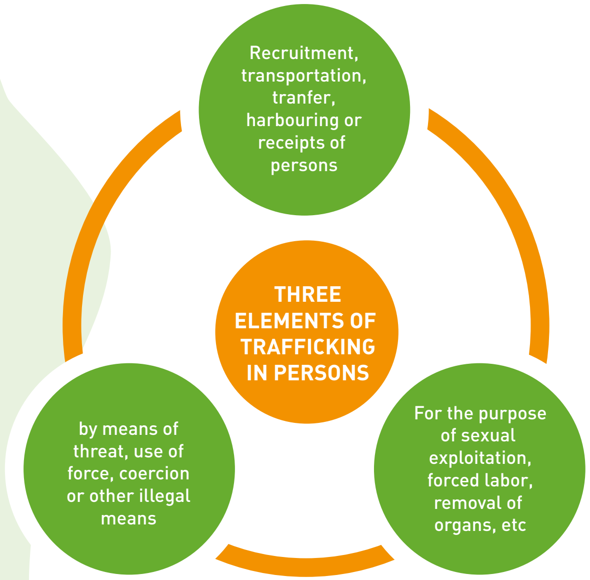

Figure 1. The three elements of Trafficking in Persons. from: www.sb.gov.hk/eng/special/bound/iimm.htm

FACTS AND FIGURES

- The number of victims of human trafficking in the Netherlands per year is estimated to be 6,250.
- This is 6 times more than the number of reported victims; human trafficking is often a hidden problem.
- 56% of all trafficking in human beings is **domestic** (meaning it happens to Dutch nationals within Dutch borders); the vast majority of this group is trafficked for the purpose of sexual exploitation
- About half of all domestic victims are minors
- Foreign victims often come from Romania, Poland, Hungary, Bulgaria and Nigeria.
- There are ICD-10 codes for trafficking in human beings
- Foreign victims of trafficking in human beings always have the right to a temporary residence permit
- There are shelters for foreign and domestic victims
- For information on organisations who work on this topic see www.wegwijzermensenhandel.nl

This fact sheet was developed by the Dutch national coordination centre against human trafficking (CoMensha) in collaboration with various chain partners.

TRAFFICKING IN HUMAN BEINGS

In the Netherlands, human trafficking (or 'trafficking in human beings' or 'trafficking in person') is understood to mean:

- **what:** recruiting (for work), transporting or housing someone,
- **how:** using coercion, violence, deception or abuse of a vulnerable position (such as with children or people with a mental disability),
- **the goal:** to reduce or not pay out someone's income (*exploitation*).

This takes place in the sex industry, as well as in many other labour sectors (such as agriculture, construction, domestic work, forced begging or crime (e.g. forced cannabis cultivation or drug dealing). Organ trafficking and forced surrogacy are also forms of trafficking in human beings. Human trafficking can take place across borders or within the Netherlands ('domestic human trafficking'). Domestic human traffickers for the purpose of sexual exploitation are often called 'loverboys' in the Netherlands (although we believe this term to be too euphemistic).

POSSIBLE SIGNS/INDICATORS: HOW TO IDENTIFY IT

All forms

- there is usually a companion with the person
- vague explanations for injuries
- threats (to the victim him- or herself or to relatives, e.g. younger sisters or parents)
- work related health problems (e.g. accidents at work in construction and multiple pregnancies, abortions, STIs or vaginal and genital complaints in sex work)
- psychosomatic health problems (psychological complaints that express themselves physically)
- drug/alcohol addiction
- tattoos of the human trafficker (such as the name or an icon)

Trafficking in human beings WITHIN borders (origin and destination country are the same)

- increasing isolation from family/friends + dependency on others
- suddenly having a lot of money / other clothing
- behavioural changes
- extortion (e.g. with movies)
- deteriorating school or work performance

TRAFFICKING IN HUMAN BEINGS ACROSS BORDERS:

- living in the workplace
- passport not in own possession
- did not arrange one's own travel
- work address not known
- accrued 'debts' (not really debts, but the traffickers say they have debts for the travel arrangements and possibly other matters)
- being undocumented

RISK FACTORS: WHO IS EXTRA VULNERABLE?

Previous victims of (sexual) violence, persons with psychological complaints (such as mild intellectual disabilities), originating from multi-problem or broken families, orphans, low self-esteem, homelessness, history of violence or trauma / youth support past, refugees / undocumented people, poverty, being easy to influence, age 12-24 years, LHBTI+ (for boys).

ADVICE/REPORTING

For advice, for reporting victims or perpetrators, and/or for referring someone to care (including shelters), call:

- Veilig Thuis ("Veilig Thuis" means "Safe at Home" in Dutch, it is the organization in the Netherlands for advice on, referrals to and reporting of any type of (domestic) violence, abuse, neglect or exploitation, or other types of harm in power-imbalanced relationships). Telephone: 0800 20 00, free of charge and always open (24 hours per day, 7 days a week). It is possible to call anonymously and/or to call for advice or information only, without reporting someone.
- the Dutch national coordination centre against human trafficking (CoMensha), telephone 033 44 81 186
- a domestic trafficking hotline

In case of acute danger call the emergency services at the phone number 112.

MORE INFORMATION

See the Sources.

DUTCH TRANSLATION

See here.

This fact sheet was developed by the Dutch national coordination centre against human trafficking (CoMensha) in collaboration with various chain partners.

POINTS OF ATTENTION WHEN GOING THROUGH THE 5 STEPS IN THE REPORTING CODE

For any form of (domestic) violence, abuse, neglect or exploitation, professionals in the Netherlands are required to use the Reporting Code. For general reporting code guidelines (such as the 5 steps in this code) visit the link; these are not described in this fact sheet. We do describe here points of attention that are specific to the topic of this fact sheet. These are:

- Human traffickers are often family members/friends/partners/acquaintances (in 68% of all cases of human trafficking in the Netherlands!).
- Speak to someone alone! If you do not speak the same language, engage an external interpreter
- Men also become victims of human trafficking, including of sexual exploitation.
- Pay extra attention to your own safety, that of the victim and his/her family
- People do not always see themselves as 'being trafficked', even when according to our standards and values they are. Regularly, given the context of their origin country, this point of view is understandable (which does not mean you should not act). At other times, people are not able (or refuse) to see the nature of their situation because of the influences of their trafficker. On top of this, even when you think someone is a 'victim', people rarely conceptualize themselves as such, so be hesitant in using this label.
- Human trafficking and human smuggling are not the same!
- Foreign people who have been trafficked often have little knowledge of their rights and little confidence in the police

This fact sheet was developed by the Dutch national coordination centre against human trafficking (CoMensha) in collaboration with various chain partners.

SOURCES TRAFFICKING IN HUMAN BEINGS

ORGANISATIONS INVOLVED

The following organisations were involved in making this fact sheet:

- CoMensha, the national coordination centre against human trafficking. For questions and/or remarks about the fact sheet, please email the main authors: Rik Viergever, rik@pro-bono.nl and Sandra van den Berg, s.vandenberg@comensha.nl
- Augeo Foundation
- Bureau Nationaal Rapporteur Mensenhandel en Seksueel Geweld tegen Kinderen
- Centrum tegen Kinderhandel en Mensenhandel (CKM)
- FairWork
- GGD GHOR Netherlands
- Nederlands Jeugdinstituut (NJi)

SOURCES

The following documents and other sources provide more information about the topic of this fact sheet:

- E-learning 'Herkenning Mensenhandel'. Het Rode Kruis. www.rodekruis.nl/elearnings/herkenning-mensenhandel/story_html5.html
- E-learning 'Signaleren van dwang en uitbuiting in de prostitutie'. SOAAIDS Nederland. moodle-sanl.nl/
- E-learning 'signalering mensenhandel'. CCV. hetccv.nl/onderwerpen/mensenhandel/e-learning-signalering-mensenhandel/
- www.signalenkaart.nl
- Signalerings-protocol mensenhandel. Veilig Thuis en Moviera. 2016. Ede. www.moviera.nl/wp-content/uploads/2016/07/Signaleringsprotocol-mensenhandel-augustus-2016.pdf
- Marjan Wijers en Marcia Albrecht. Handreiking Signalering Mensenhandel voor werkers in de gezondheidszorg. SOA aids, 2014. www.soaaids.nl/sites/default/files/documenten/Prostitutie/0153HandreikingMensenhandel_web2.pdf
- Signaleringsprotocol Loverboys. Steunpunt Huiselijk Geweld. 2014. www.moviera.nl/wp-content/uploads/2014/02/20140224-signaleringsprotocol-loverboysdefinitief.pdf
- Hoe signaleer je slachtoffers? Stappenplan voor professionals. Aanpak van loverboy/mensenhandel problematiek in de zorg voor jeugd. Nederlands Jeugdinstituut, 2015. www.jeugdzorgnederland.nl/contents/documents/2016-actieplan-azough---handreiking-en-stappenplan-signalering.pdf
- Minderjarige jongens die hun lichaam exploiteren: Jongensprostitutie. Ruilseks signaleren, bespreekbaar maken en motiveren tot stoppen. MOVISIE, 2013. www.movisie.nl/publicaties/jongensprostitutie-minderjarige-jongens-die-hun-lichaam-exploiteren
- Slachtoffers loverboys: signalering. NJi. www.nji.nl/nl/Kennis/Dossier/Slachtoffers-loverboys/Aanpak/Signalering
- Slachtoffers loverboys: Risicoprofiel slachtoffers. NJi. www.nji.nl/nl/Kennis/Dossier/Slachtoffers-loverboys/Achtergrond/Risicoprofiel-slachtoffers
- Seksuele uitbuiting van jongens in Nederland. Paul van Gelder et al, SHOP Den Haag, Amsterdam, 2017. www.rijksoverheid.nl/documenten/rapporten/2017/05/08/tk-bijlage-1-eindrapport-seksuele-uitbuiting-van-jongens-in-nederland
- Mensenhandel: vijfde rapportage van de Nationaal Rapporteur. Bureau Nationaal Rapporteur Mensenhandel, Den Haag. www.nationaalrapporteur.nl/binaries/rapportage-5-(ned)-2006_tcm23-34835.pdf
- Sue Berelowitz et al. "I thought I was the only one. The only one in the world" The Office of the Children's Commissioner's Inquiry into Child Sexual Exploitation In Gangs and Groups. Interim report. Office of the Childrens'Commissioner, November 2012. static.lgfl.net/LgflNet/downloads/online-safety/LGfL-OS-Research-Archive-2012-Childrens-Commissioner-CSE.pdf
- Anika Boersma et al. Signalenkaart mannelijke slachtoffers in de seksuele uitbuiting.
- Slachtoffermonitor mensenhandel 2012-2016. Nationaal Rapporteur Mensenhandel en Seksueel Geweld tegen Kinderen, 2017, Den Haag. www.nationaalrapporteur.nl/binaries/Slachtoffermonitor%20mensenhandel%202012-2016_Nationaal%20Rapporteur%20(i)_tcm23-285357.pdf
- Repetur, L.; Veenstra, J. Vrijbuiters uitgebuit. SWP, 2010.
- Raad voor de Kinderbescherming en Jeugdbescherming en Reclassering. Landelijke aanpak Raad voor de Kinderbescherming en Gecertificeerde Instellingen bij vermoedelijke slachtoffers van criminele uitbuiting. ttps://hetccv.nl/fileadmin/Bestanden/Onderwerpen/Multiprobleem_gezin/fact sheet_Aanpak_Criminele_kinderuitbuiting.pdf

This fact sheet was developed by the Dutch national coordination centre against human trafficking (CoMensha) in collaboration with various chain partners.

SOURCES TRAFFICKING IN HUMAN BEINGS

- BNRM, 2012. Mensenhandel: met het oogmerk van orgaanverwijdering en gedwongen commercieel draagmoederschap. www.nationaalrapporteur.nl/binaries/mensenhandel-met-het-oogmerk-van-orgaanverwijdering-en-gedwongen-commercieel-draagmoederschap-(2012)_tcm23-34769.pdf (Nederlands) www.nationaalrapporteur.nl/binaries/human-trafficking-for-the-purpose-of-the-removal-of-organs-and-forced-commercial-surrogacy_tcm23-34770.pdf (engels)
- Orgaanhandel en mensenhandel met het oogmerk van orgaanverwijdering: Een verkennend onderzoek naar de betrokkenheid van Nederland en Europa. Jessica de Jong. 2014, Politie, Woerden. www.politie.nl/binaries/content/assets/politie/algemeen/publicaties-archief/orgaanhandel-en-mensenhandel-in-nederland-en-europa.pdf
- Wat is mensenhandel? CoMensha. www.comensha.nl/pagina/wat-is-mensenhandel . Accessed 2 oktober 2018.
- Oosterse teelt: Vietnamezen in de hennepteelt. Yvette Schoenmakers, Bo Bremmers, Anton van Wijk. 2012, Bureau Beke. www.beke.nl/doc/2012/download_Oosterse_teelt.pdf
- Mensenhandel in en uit beeld II. Cijfermatige rapportage 2008-2012. Nationaal Rapporteur Mensenhandel en Seksueel Geweld tegen Kinderen, Den Haag, 2014. www.nationaalrapporteur.nl/Publicaties/MensenhandelinenuitbeeldII/index.aspx
- Mensensmokkel en mensenhandel. Amnesty international. www.amnesty.nl/encyclopedie/mensensmokkel-en-mensenhandel . Accessed 2 oktober 2018.
- CoMensha. Hoeveel slachtoffers van mensenhandel zijn er? www.comensha.nl/pagina/hoeveel-slachtoffers-van-mensenhandel-zijn-er Accessed 2 oktober 2018.
- CoMensha. Mensenhandel in Nederland: het beeld van 2016. www.comensha.nl/download/jaarverslagen
- Office on trafficking in persons. CDC Adds New Human Trafficking Data Collection Fields for Health Care Providers. www.acf.hhs.gov/otip/news/icd-10 Accessed 2 oktober 2018.
- Vreemdelingencirculaire 2000 (B). wetten.overheid.nl/BWBR0012289/2018-05-23#Circulaire.divisieB8_Circulaire.divisie3 Accessed 2 juni 2018.
- Een veilige opvangplek vanwege mensenhandel. Slachtofferwijzer.nl www.slachtofferwijzer.nl/hulppagina/mensenhandel/een-veilige-opvangplek-vanwege-mensenhandel/ . Accessed 2 oktober 2018.
- Wegwijzer mensenhandel. www.wegwijzermensenhandel.nl Accessed 2 oktober 2018.

ONLINE SEXUAL INTIMIDATION

ALWAYS USE THE REPORTING CODE WHEN YOU ENCOUNTER A FORM OF (DOMESTIC) VIOLENCE, ABUSE, NEGLECT OR EXPLOITATION! ▶

This fact sheet is part of a series about *(domestic) violence, abuse, neglect, exploitation* and other types of harm that may be inflicted onto someone in a power-imbalanced relationship. Power-imbalanced relationships can exist with anyone, for example: an (ex-)partner, a child, a parent, a sibling, another family member, an informal or a professional carer, a friend, a flatmate or neighbour, a teacher, a colleague or supervisor, or just someone you know. These fact sheets describe different types of harm that can be inflicted in these relationships. They are meant as an add-on to the Dutch Reporting Code for these issues (English version here) and were developed for two reasons: 1) To provide professionals with an overview of all the types of harm that exist, to aid them in identifying both well-known and lesser-known types (see the Overview). 2) Signs/indicators may vary greatly by type of harm and certain types of harm require specific courses of action; the fact sheets help professionals with identifying the signs/indicators and risk factors of *each specific type* of harm and with acting appropriately when they do. Note: the general 5 steps in the Reporting Code are applicable to all types of harm in power-imbalanced relationships; the factsheets provide more guidance within these 5 steps – they are an add-on, not a replacement.

Below is a brief introduction to this topic, an overview of the signs/indicators and risk factors associated with this type of harm, and points of attention for when you encounter it.

WHAT IS ONLINE SEXUAL HARASSMENT?

The European Union Agency for Fundamental Rights (FRA) defines online sexual harassment as: receiving unwanted, offensive, sexually oriented e-mails or text messages or experiencing inappropriate sexual advances through social media or online chat rooms.[1] There is a continuum of behaviours that differ in nature and seriousness, with opinions varying about what is or is not socially or legally permissible.[2]

Forms of online sexual harassment are:

- Shame-sexting:[3] images of a sexual nature (often created by the person depicted) are shared with third parties via social media without the person's consent.[4]
- Grooming: the active approach and seduction of minors by adults via the Internet and social networking sites, chat rooms or webcams for the purpose of developing sexual contacts.
- Revenge pornography: shame-sexting with revenge as motive, often after a relationship ended.
- Sextortion: images of a sexual nature (often obtained under false pretences) are used to blackmail the person depicted
- Online distribution of sexual violence: recordings or images of forced sexual acts are distributed online to a wider audience
- Unsolicited sending or posting of messages of a sexual nature: this may involve gossip or bullying of a sexual nature.

FACTS AND FIGURES

Various studies carried out in the Netherlands show that:

- 14% of girls and 6% of boys aged 12-25 years had at least one experience with sexting that they found annoying[9].
- girls have received unwanted sexual advances on the internet about three times more often than boys [10]
- girls find sexually-tinged communication more annoying than boys[11]

This fact sheet was developed by Atria, Kennisinstituut voor Emancipatie en Vrouwengeschiedenis in collaboration with various chain partners.

ONLINE SEXUAL INTIMIDATION

POSSIBLE SIGNS/INDICATORS: HOW TO IDENTIFY IT

Victims regularly experience feelings of shame, so they often do not report an incident. Fear of how loved ones will respond makes identification difficult. Victims often show psychological problems such as anxiety and stress,[5] they withdraw and avoid social activities.[6] In addition, victims often experience a decrease in productivity at school/work and young people may be absent from school frequently.[7]

RISK FACTORS: WHO IS EXTRA VULNERABLE?

Young people and girls are at increased risk of sexual harassment online. International studies indicate that girls and women are targeted up to three times more often for sexual and unwanted advances than boys and men. In addition, various studies have shown that young people who run the risk of unwanted sexual advances 'offline' will also be more likely to be affected online.[8]

POINTS OF ATTENTION WHEN GOING THROUGH THE 5 STEPS IN THE REPORTING CODE

For any form of (domestic) violence, abuse, neglect or exploitation, professionals in the Netherlands are required to use the Reporting Code. For general reporting code guidelines (such as the 5 steps in this code) visit the link; these are not described in this fact sheet. We do describe here points of attention in going through the 5 steps that are specific to the topic of this fact sheet. These are:

- Preferably speak to someone alone
- Victims often do not know where to turn to: therefore, explain where they can get help.
- Victims may have to overcome shame and guilt.
- Victims are often unaware of their rights: explain these clearly
- For minors: discuss how parents can support. Follow the professional code when considering whether it can be kept secret from parents.
- For advice you can always contact Veilig Thuis.
- In some cases online sexual harassment is covered by the reporting code domestic violence and child abuse and in some cases not: contact Veilig Thuis for advice.

ADVICE/REPORTING

For advice, for reporting victims or perpetrators, and/or for referring someone to care (including shelters), call:

- Veilig Thuis ("Veilig Thuis" means "Safe at Home" in Dutch, it is the organization in the Netherlands for advice on, referrals to and reporting of any type of (domestic) violence, abuse, neglect or exploitation, or other types of harm in power-imbalanced relationships). Telephone: **0800 20 00**, free of charge and always open (24 hours per day, 7 days a week). It is possible to call anonymously and/or to call for advice or information only, without reporting someone.
- or see the websites under "More information"

In case of acute danger call the emergency services at the phone number **112**.

MORE INFORMATION

See the Sources, the online sexual harassment fact sheet by Atria and the following websites:

- Atria
- Centrum Seksueel Geweld
- Meldknop
- Politie

DUTCH VERSION

See here.

This fact sheet was developed by Atria, Kennisinstituut voor Emancipatie en Vrouwengeschiedenis in collaboration with various chain partners. september 2020

NOTE

For all forms of domestic violence and child abuse, the Dutch Reporting Code (English version here) for these issues must be applied in the Netherlands by all groups of professionals named in the Reporting Code law. Sexual behaviour between young people that crosses boundaries does not fall under the definition of domestic violence or child abuse in the Netherlands and therefore, it is not legally required to use the reporting code when you encounter it as a professional. However, the reporting code may be used. Because we feel it is a useful guideline for professionals for this type of harm as well, and because it is important that professionals (e.g. general practitioners) can identify this type of harm and take the right steps, this factsheet was compiled.

ORGANISATIONS INVOLVED

The following organisations were involved in making this fact sheet:

- Movisie. For questions and/or remarks about the fact sheet, please email the main author: Wilma Schakenraad, w.schakenraad@movisie.nl
- Veilig Thuis
- Kennisinstituut voor Emancipatie en Vrouwengeschiedenis (Atria)

SOURCES

The following documents and other sources provide more information about the topic of this fact sheet:

Publications

- Berlo van, W. & Beek I. van (2015). Whitepaper Seksuele grensoverschrijding en seksueel geweld. Feiten en cijfers Utrecht: Rutgers en Movisie.
- Graaf, H. de, Borne, M. van den, Nikkelen, S., Twisk, D., & Meijer, S. (2017). Seks onder je 25e. Utrecht / Amsterdam: Rutgers / Soa Aids Nederland.
- Haas, S. de (2012). Seksueel grensoverschrijdend gedrag onder jongeren en volwassenen in Nederland. Tijdschrift voor Seksuologie, 36-2, p. 136-145.
- Höing, M., & Janssen, J. (2017). Seksueel grensoverschrijdend gedrag. In: Höing, M., & Janssen, J., Boer, A., & Liebregts, M. (red.). Bespreekbaar maken van seksualiteit en intimiteit. Handboek voor professionals in zorg en welzijn. Bussum: Coutinho.
- Nationaal Rapporteur Mensenhandel en Seksueel Geweld tegen Kinderen (2014). Op goede grond. De aanpak van seksueel geweld tegen kinderen. Den Haag: Nationaal Rapporteur.
- Römkens, R. (2017). Factsheet Online seksuele intimidatie. Amsterdam: Atria. Zie: www.atria.nl/sites/atria/files/atoms/files/factsheet-cybergeweld-onlineversiedef.pdf
- Storms, O. & Doornink, N. (2016). Vlaggensysteem: Reageren op seksueel (grensoverschrijdend) gedrag van kinderen en jongeren. Effectieve sociale interventies en Effectieve interventies huiselijk en seksueel geweld. Utrecht: Movisie.

Websites

- seksonderje25e.nl (Rutgers)
- Kennisdossier seksuele grensoverschrijding van Rutgers: www.rutgers.nl/feiten-en-cijfers/kennisdossiers/kennisdossier-seksuele-grensoverschrijding
- www.seksueelgeweld.info Website for victims of sexual violence, and for those involved and referrers. See also the social map with an overview of the available help for victims and perpetrators of sexual violence.
- www.vlaggensysteem.nl
- www.act4respect.nl
- www.atria.nl

This fact sheet was developed by Atria, Kennisinstituut voor Emancipatie en Vrouwengeschiedenis in collaboration with various chain partners.

STALKING

This fact sheet is part of a series about *(domestic) violence, abuse, neglect, exploitation* and other types of harm that may be inflicted onto someone in a power-imbalanced relationship. Power-imbalanced relationships can exist with anyone, for example: an (ex-)partner, a child, a parent, a sibling, another family member, an informal or a professional carer, a friend, a flatmate or neighbour, a teacher, a colleague or supervisor, or just someone you know. These fact sheets describe different types of harm that can be inflicted in these relationships. They are meant as an add-on to the Dutch Reporting Code for these issues (English version here) and were developed for two reasons: 1) To provide professionals with an overview of all the types of harm that exist, to aid them in identifying both well-known and lesser-known types (see the Overview). 2) Signs/indicators may vary greatly by type of harm and certain types of harm require specific courses of action; the fact sheets help professionals with identifying the signs/indicators and risk factors of *each specific type* of harm and with acting appropriately when they do. Note: the general 5 steps in the Reporting Code are applicable to all types of harm in power-imbalanced relationships; the factsheets provide more guidance within these 5 steps – they are an add-on, not a replacement.

Below is a brief introduction to this topic, an overview of the signs/indicators and risk factors associated with this type of harm, and points of attention for when you encounter it.

ALWAYS USE THE REPORTING CODE WHEN YOU ENCOUNTER A FORM OF (DOMESTIC) VIOLENCE, ABUSE, NEGLECT OR EXPLOITATION! ▶

WHAT IS STALKING?

Stalking (also known as stalking) is a deliberate and systematic harassment of someone, as a result of which that person no longer feels safe. It is often an invisible and difficult to understand terror; an accumulation of behaviour that is not always punishable in itself. Examples of stalking are chasing, threatening with violence, making unwanted phone calls and sending messages or ordering things in the name of someone else. Stalking can take place at home, elsewhere and/or online. Stalking is punishable in the Netherlands.[1]

Stalking is by definition a form of violence because it is a major violation of the perception of security in and on the lives of victims.

A stalker may have (had) an (intimate) relationship with the victim, but this is not necessary.

POSSIBLE SIGNS/INDICATORS: HOW TO IDENTIFY IT

The privacy of the victim is constantly and intentionally infringed, for example, with the aim of forcing the victim to react (for example, or to restore the relationship), frighten or hinder something.[2] Stalking concerns a pattern of behaviour in which every incident does not have to be very violent and/or punishable, but the combination of all those incidents is. For service providers it is important to realise that stalking has consequences for the feeling of safety and well-being of the victim. There is tension and stress. The victim is hindered in his daily functioning.

MORE INFORMATION

See the Sources and the publication "If you are stalked".

ADVICE/REPORTING

For advice, for reporting victims or perpetrators, and/or for referring someone to care (including shelters), call:

- Veilig Thuis ("Veilig Thuis" means "Safe at Home" in Dutch, it is the organization in the Netherlands for advice on, referrals to and reporting of any type of (domestic) violence, abuse, neglect or exploitation, or other types of harm in power-imbalanced relationships). Telephone: **0800 20 00**, free of charge and always open (24 hours per day, 7 days a week). It is possible to call anonymously and/or to call for advice or information only, without reporting someone.

In case of acute danger call the emergency services at the phone number **112.**

This fact sheet was developed by Slachtofferhulp Nederland in collaboration with various chain partners.

STALKING

To estimate the risk that the person being stalked runs, several questions can be helpful (see p4 and 5 of the publication "If you are stalked").

POINTS OF ATTENTION WHEN GOING THROUGH THE 5 STEPS IN THE REPORTING CODE

For any form of (domestic) violence, abuse, neglect or exploitation, professionals in the Netherlands are required to use the Reporting Code. For general reporting code guidelines (such as the 5 steps in this code) visit the link; these are not described in this fact sheet. We do describe here points of attention in going through the 5 steps that are specific to the topic of this fact sheet. These are:

- An important rule of thumb: take the victim's feelings seriously: if they are really afraid, then there is actually a risk of danger/ threat.
- There are a number of rules of thumb for victims themselves in what they can do when they are stalked: see p7 and 8 of the publication "If you are stalked".
- It is important to gather evidence.3
- Tune in with the Police. In an acute dangerous or threatening situation, call the police immediately.
- Victims of stalking are entitled to protection. For example, the public prosecutor or judge can impose an area and/or contact ban on the stalker. Read: more about rights in case of stalking.4
- Involve neighbours, family members, friends and colleagues who can watch out and support.

If necessary, a mobile alarm system (AWARE) or a placement within the shelter can be realized.

- Slachtofferhulp Nederland (this name means "Victim Support in the Netherlands" in Dutch), telephone 0900 01 01, offers practical support but can also refer to help with processing or groups of fellow sufferers.

DUTCH TRANSLATION

See here.

This fact sheet was developed by Slachtofferhulp Nederland in collaboration with various chain partners. september 2020

SOURCES STALKING

NOTE

For all forms of domestic violence and child abuse, the Dutch Reporting Code (English version here) for these issues **must** be applied in the Netherlands by all groups of professionals named in the Reporting Code law. Stalking does not fall under the definition of domestic violence or child abuse in the Netherlands and therefore, it is not legally required to use the reporting code when you encounter it as a professional. However, the reporting code **may** be used. Because we feel it is a useful guideline for professionals for this type of harm as well, and because it is important that professionals (e.g. general practitioners) can identify this type of harm and take the right steps, this factsheet was compiled.

ORGANISATIONS INVOLVED

The following organisations were involved in making this fact sheet:

- Slachtofferhulp Nederland. For questions and/or remarks about the fact sheet, please email the main author: Franck Wagemakers, f.wagemakers@slachtofferhulp.nl
- AROSA: Magda Vogelezang
- Nationale politie: Cleo Brandt en Berdien Zuurveen
- Veilig Thuis: Inge Sauvé

SOURCES

The following documents and other sources provide more information about the topic of this fact sheet:

- www.slachtofferhulp.nl/gebeurtenissen/stalking
- www.movisie.nl/publicatie/u-wordt-gestalkt "Als u wordt gestalkt", Movisie
- www.slachtofferhulp.nl/gebeurtenissen/stalking/bewijs-verzamelen-gestalkt
- www.slachtofferhulp.nl/gebeurtenissen/stalking/rechten/#bekijk-uw-rechten

This fact sheet was developed by Slachtofferhulp Nederland in collaboration with various chain partners. september 2020

HONOUR-BASED VIOLENCE

This fact sheet is part of a series about *(domestic) violence, abuse, neglect, exploitation* and other types of harm that may be inflicted onto someone in a power-imbalanced relationship. Power-imbalanced relationships can exist with anyone, for example: an (ex-)partner, a child, a parent, a sibling, another family member, an informal or a professional carer, a friend, a flatmate or neighbour, a teacher, a colleague or supervisor, or just someone you know. These fact sheets describe different types of harm that can be inflicted in these relationships. They are meant as an add-on to the Dutch Reporting Code for these issues (English version here) and were developed for two reasons: 1) To provide professionals with an overview of all the types of harm that exist, to aid them in identifying both well-known and lesser-known types (see the Overview). 2) Signs/indicators may vary greatly by type of harm and certain types of harm require specific courses of action; the fact sheets help professionals with identifying the signs/indicators and risk factors of *each specific type* of harm and with acting appropriately when they do. Note: the general 5 steps in the Reporting Code are applicable to all types of harm in power-imbalanced relationships; the factsheets provide more guidance within these 5 steps – they are an add-on, not a replacement.

Below is a brief introduction to this topic, an overview of the signs/indicators and risk factors associated with this type of harm, and points of attention for when you encounter it.

ALWAYS USE THE REPORTING CODE WHEN YOU ENCOUNTER A FORM OF (DOMESTIC) VIOLENCE, ABUSE, NEGLECT OR EXPLOITATION! ▶

HONOUR

Honour has to do with the reputation of integrity and reliability.

In some families and communities family honour plays an important role. Moral norms regarding sexuality determine the relationship between men and women. If these standards are used in one's family, then 'you are part of it'. When a person's reputation is not good owing to moral misconduct, he/she is considered immoral and therefore a bad person. As a result, the individual can be excluded. Immoral behaviour of a family member can also seriously damage the reputation of the integrity and reliability (family honour) of other family members with the risk of family exclusion by the community. The family will do everything in its power to prevent exclusion.

Individual choices thus have a direct influence on the position of family members and the position in the community. In these families the sense of shame of "undesirable" behaviour and the fear that this behaviour will be known to third parties are important factors.

FACTS AND FIGURES

Every year the Dutch police see 2,500 - 3,000 offences in which family honour plays a (presumed) role. An average of 460 offences is referred to the 'Landelijk Expertisecentrum Eergerelateerd Geweld' because of their complexity. Of these, 7 to 17 end fatally (murder, manslaughter or suicide).

ADVICE/REPORTING

For advice, for reporting victims or perpetrators, and/or for referring someone to care (including shelters), call:

- Veilig Thuis ("Veilig Thuis" means "Safe at Home" in Dutch, it is the organization in the Netherlands for advice on, referrals to and reporting of any type of (domestic) violence, abuse, neglect or exploitation, or other types of harm in power-imbalanced relationships). Telephone: **0800 20 00**, free of charge and always open (24 hours per day, 7 days a week). It is possible to call anonymously and/or to call for advice or information only, without reporting someone.

In case of acute danger call the emergency services at the phone number **112**.

This fact sheet was developed by Fier in collaboration with various chain partners. september 2020

HONOUR-BASED VIOLENCE

HONOUR-BASED VIOLENCE

Honour-based violence is the umbrella term for forms of intimidation, coercion and psychological and physical violence committed from an honour-based motive. The purpose of the violence is to prevent a family member from behaving in a way that could harm the family honour. There are often orthodox views on the role of women and men, and on the sexuality and reproductive rights of women. The honour of the woman is linked to her sexuality. And the sexual honour of the woman is linked to the family honour. The violence always concerns behaviour that has become known to people in the environment of the family.
A commonly used definition: 'Honour-based violence includes any form of mental or physical violence, committed from a collective mentality in response to a (threat of) violation of the honour of a man or a woman and thus of his or her family, of which the outside world is aware or is at risk of becoming aware' (Working definition Ministry of Justice, Beke 2005).

Honour-based violence can take place both between an individual and the group and between different groups. If there is a risk of honour violations, these must be prevented by the family. When honour is violated, it must be restored by the family. The family is addressed by the community if it does not respond sufficiently to an infringement of the family honour. Girls and women have the responsibility to uphold the family honour; parents, men and boys to guard and if necessary restore it.

FORMS OF HONOUR-BASED VIOLENCE

Honour related violence has different manifestations and degrees of seriousness, for example threatening, great psychological pressure, restricting freedoms, controlling, isolating, healing rituals and incantations, (forced) abortion / abandonment of a child, physical violence, forced marriage, marital imprisonment, rejection, forced abandonment abroad, (female genital) mutilation, being forced to commit suicide, and honour killings.

It is important that a distinction is made between: honour-based violence; domestic violence, violence for the sake of the individual's honour; incapacity to educate; and child abuse. In the case of honour-based violence a motive of honour is explicitly present in a collective context; other interventions are appropriate in those situations.

POSSIBLE SIGNS/INDICATORS: HOW TO IDENTIFY IT

Signs/indicators are non-specific, i.e. there are no specific signals of honour-based violence.

Signs that *may* indicate honour-based violence are:
A fear of family violence, behavioural changes and or complaints such as depression, negative self-image, self-harm, eating problems and bad sleep, walking away from home, (structural) absenteeism from school and or daytime activities, reduced performance in school or at work, not looking forward to upcoming holidays, (suddenly) being met and/or brought by family members (surveillance), withholding medical care, breaking social contacts without clear reason, change of clothing, sudden announcement of engagement and / or marriage, gossip in the community, no longer having access to identity documents.

Shelter and help can be arranged in the region. For consultation / advice and specific shelter and treatment, call national one of the expertise centres: Sterk Huis at 013 54 33 073 or Fier **088 20 80 000** or www.chatmetfier.nl

For advice on marriage coercion and abandonment, call:

- Landelijk Knooppunt Huwelijksdwang en Achterlating (LKHA) at **070 34 54 319**

MORE INFORMATION

See the Sources and the 4 other fact sheets about in this series about specific forms of Honour-based violence:

- marriage coercion
- forced abandonment
- female genital mutilation
- hidden women

DUTCH TRANSLATION

See here.

This fact sheet was developed by Fier in collaboration with various chain partners. september 2020

Please note: there are almost always feelings of guilt and shame and conflicts of loyalty.

RISK FACTORS: WHO IS EXTRA VULNERABLE?

- Women: a 'supposed' relationship, sex before marriage, a [supposed] extramarital relationship, unmarried pregnancy, refusing an arranged marriage, lesbian identity and/or relationship;
- Men: relationship/sex with a girl who is not allowed this by her family, relationship/sex with a married woman, refusing an arranged marriage, homosexual identity and/or relationship, refusing to carry out honour-based violence with regard to sister, niece or other family member;
- Bad contacts who, for example, threaten to place compromising images on the internet (sexting / digital blackmail);
- Sharp social control, gossip about (alleged) misconduct, misconduct is known in the area;
- (Threatening) honour-based violence is discussed with police / assistance (this can become known in the community);
- High interdependence within a family where family honour is important;
- Previous honour-based violence in the family;
- Strict / traditional moral standards with respect to moral family honour;
- Socio-economic problems, social isolation, multi-problem families.

POINTS OF ATTENTION WHEN GOING THROUGH THE 5 STEPS IN THE REPORTING CODE

For any form of (domestic) violence, abuse, neglect or exploitation, professionals in the Netherlands are required to use the Reporting Code. For general reporting code guidelines (such as the 5 steps in this code) visit the link; these are not described in this fact sheet. We do describe here points of attention in going through the 5 steps that are specific to the topic of this fact sheet. These are:

- Risk assessment and analysis always by police experts using a checklist for honour-based violence;
- It is very important not to involve or inform parents directly because of the risk of escalation and potential shame;
- There is a specific reporting code for honour-based violence;
- Via the police and Veilig Thuis a consultation, valuation and analysis can be performed by the 'Landelijk Expertisecentrum Eergerelateerd geweld'
- If in doubt: see at "Advice/reporting".

This fact sheet was developed by Fier in collaboration with various chain partners. september 2020

SOURCES HONOUR-BASED VIOLENCE

ORGANISATIONS INVOLVED

The following organisations were involved in making this fact sheet:

- Fier - expertise en behandelcentrum op het terrein van geweld in afhankelijkheidsrelaties. For questions and/ or remarks about the fact sheet, please email the main author: Gerda de Groot, GdeGroot@fier.nl
- Samen Veilig (Veilig Thuis regio Utrecht)
- Suzanne Tan, bureau TANGRAM
- Diny Flierman, Landelijk Knooppunt Huwelijksdwang en Achterlating
- Sandra Hamming, GGD GHOR
- Marga Haagmans, AUGEO
- Janine Janssen, LEC EGG

SOURCES

The following documents and other sources provide more information about the topic of this fact sheet:

Background

- Bakker, H., Storms, O. (2016). Factsheet De meldcode bij (vermoedens van) eergerelateerd geweld. Utrecht: Movisie.
- Brekelmans, I. & Groot, G. de (2014). De kleuren van Eva en Zahir. Amsterdam: SWP Uitgeverij.
- Dijke, A. van & Terpstra, L. (2010). De dochters van Zahir. Tussen traditie en wereldburgerschap. Amsterdam: SWP Uitgeverij.
- Ermers, R. (2018). Honor Related Violence. A new social psychological Perspective). Routledge, www.routledge.com
- Ferwerda, H.B & Leiden, I. van (2005). Eerwraak of eergerelateerd geweld? Naar een werkdefinitie. Arnhem: Advies- en Onderzoekgroep Beke.
- Janssen, J. & Sanberg, R. (2013) Uniformiteit in cijfers. Mogelijke eerzaken in 2010, 2011 en 2012. Den Haag: Landelijk Expertise Centrum Eer Gerelateerd Geweld.
- Janssen, J. (2013). De rol van religie bij het afbakenen, verklaren en aanpakken van eergerelateerd geweld. Tijdschrift voor Religie, Recht en Beleid (4)1: 5-15.
- Janssen, J. (2017). Focus op eer. Een verkenning van eerzaken voor politieambtenaren en andere professionals. Den Haag: Boom Criminologie.
- Vlamings, B., Geijn. R. van, Brekelmans, I. (2012). Methodiek Safe and Streetwise. Tilburg: Kompaan en De Bocht.
- Werson, S., Lamers, F., Pers, M. van der, & Dijke, A. van (2015). Fier en verder. Meiden over hun leven na de hulpverlening. Amsterdam: Uitgeverij SWP.

Fact sheets on specific forms of honour-based violence

- Female Genital Mutilation (FGM)
- Forced marriages
- Forced abandonment
- Forced isolation

National expertise and treatment centres

- www.fier.nl
- www.sterkhuis.nl

Internet

- www.huiselijkgeweld.nl/dossiers/eergerelateerdgeweld
- www.rijksoverheid.nl/onderwerpen/eergerelateerd-geweld
- www.huwelijksdwangenachterlating.nl
- www.movisie.nl/publicatie/eergerelateerd-geweld-seksuele-genderdiversiteit
- www.politie.nl/themas/eergerelateerd-geweld.html
- www.politie.nl/themas/eergerelateerd-geweld-voor-professionals.html
- www.eerenvrijheid.nl
- www.kis.nl/trefwoorden/eergerelateerd-geweld
- www.huiselijkgeweld.nl/nieuws/2018/270218_-eergerelateerd-geweld-is-geen-huiselijk-geweld-
- www.huiselijkgeweld.nl/doc/feiten/factsheet_EG_20p_aug%202011.pdf

Signs/indicators

- www.leceergerelateerdgeweld.nl/herkennen

Reporting and referral code

- www.meldcode.nu/eergerelateerd-geweld

HIDDEN WOMEN

ALWAYS USE THE REPORTING CODE WHEN YOU ENCOUNTER A FORM OF (DOMESTIC) VIOLENCE, ABUSE, NEGLECT OR EXPLOITATION! ▶

This fact sheet is part of a series about *(domestic) violence, abuse, neglect, exploitation* and other types of harm that may be inflicted onto someone in a power-imbalanced relationship. Power-imbalanced relationships can exist with anyone, for example: an (ex-)partner, a child, a parent, a sibling, another family member, an informal or a professional carer, a friend, a flatmate or neighbour, a teacher, a colleague or supervisor, or just someone you know. These fact sheets describe different types of harm that can be inflicted in these relationships. They are meant as an add-on to the Dutch Reporting Code for these issues (English version here) and were developed for two reasons: 1) To provide professionals with an overview of all the types of harm that exist, to aid them in identifying both well-known and lesser-known types (see the Overview). 2) Signs/indicators may vary greatly by type of harm and certain types of harm require specific courses of action; the fact sheets help professionals with identifying the signs/indicators and risk factors of *each specific type* of harm and with acting appropriately when they do. Note: the general 5 steps in the Reporting Code are applicable to all types of harm in power-imbalanced relationships; the factsheets provide more guidance within these 5 steps – they are an add-on, not a replacement.

Below is a brief introduction to this topic, an overview of the signs/indicators and risk factors associated with this type of harm, and points of attention for when you encounter it.

WHO/WHAT ARE HIDDEN WOMEN?

Hidden women are women who are forced by their partner, family and/or in-laws to live in isolation. This is often accompanied by psychological intimidation and/or (threat of) physical violence. These women are often locked up in their home against their own will and may have no or only very limited contact with others. In cases of contact outside the home, this often happens under the supervision and control of a partner, family and/or in-laws. Hidden women have little to no opportunity to participate in society, are not or hardly accessible for aid workers, and do not know how to access aid themselves (or only after a long time).

POSSIBLE SIGNS/INDICATORS: HOW TO IDENTIFY IT

- The woman does not or hardly gets out of the house.
- The woman does not show up for appointments.
- The woman is checked or accompanied by partner, family members or in-laws when she goes outside.
- The person who accompanies the woman always speaks on her behalf in conversations with professionals.
- Psychosomatic complaints.
- Domestic violence.
- The door of the house remains closed and windows are darkened.
- The woman looks anxious, timid and/or makes a lifeless impression.
- Neighbourhood children or classmates of children are never allowed to come and play.
- Children appear unkempt, anxious and/or avoiding.

FACTS AND FIGURES

Figures

It is estimated that there are a total of 600-900 hidden women living in the Netherlands in the big cities of Rotterdam, Amsterdam and The Hague alone. National figures are missing.

Facts

- Most of the hidden women come from Morocco and Turkey.
- Hidden women are women of all ages.
- The husband and his family often play an important role in keeping the woman hidden.
- The arrival or presence of children makes it even more difficult for women to break through their isolation.
- Hidden women are more likely to experience psychological harassment and physical violence.

MORE INFORMATION

See the Sources.

This fact sheet was developed by Verwey-Jonker Instituut / Kennisplatform Integratie en Samenleving in collaboration with various chain partners. september 2020

HIDDEN WOMEN

- The woman indicates that she is afraid of her husband or family.
- The woman does not want help, out of fear of more problems or violence.
- Forced residence with in-laws or family.

RISK GROUPS: AMONG WHICH GROUPS IS IT MORE PREVALENT?

- Forced residence with in-laws or family.
- Women from a closed community in which traditional ideas prevail about the role and position of women. Also communities where traditions and family honour play a major role.
- Women who migrate to the Netherlands for marriage.
- Women without a residency status or who are dependent on their partner for a residency permit.
- Women for whom a marriage is arranged with a man with a mental or physical disability.
- Women who marry a partner under pressure from the family (forced marriage).
- Women who are married to a partner with psychiatric problems, such as extreme suspicion, jealousy or paranoid behavior.

RISK FACTORS: WHO IS EXTRA VULNERABLE?

- Social pressure and control by partner, family, in-laws and/or community.
- Family honour.
- Low literacy, illiteracy and/or not being able to speak the language of the country where she lives.
- Financial dependence.
- Living in a house with the in-laws.
- Being new in a country and not knowing their way around.
- Being dependent on someone else (e.g. a partner) for their residency permit.

POINTS OF ATTENTION WHEN GOING THROUGH THE 5 STEPS IN THE REPORTING CODE

For any form of (domestic) violence, abuse, neglect or exploitation, professionals in the Netherlands are required to use the Reporting Code. For general reporting code guidelines (such as the 5 steps in this code) visit the link; these are not described in this fact sheet. We do describe here points of attention in going through the 5 steps that are specific to the topic of this fact sheet. These are:

- Professionals in primary care and education are the most important potential signers of hidden women, because these women hardly participate in society.
- Both the women themselves, and their potential children, should remain in view of social workers.
- If a hidden woman is in view of these professionals, then it is important for a professional to connect to the needs of the woman and any children: she determines the pace.
- A professional can make it explicit that men and women in the Netherlands have equal rights to participate in society (in accordance with the UN Convention on Women). Some hidden women do not know that forced isolation is punishable in the Netherlands and help is available.
- If the woman explicitly indicates that she wants to end her isolation, then, as a professional, make sure that the next safe steps are taken in consultation with Veilig Thuis ("Veilig Thuis" means "Safe at Home" in Dutch, it is the organization in the Netherlands for advice on, referrals to and reporting of any type of (domestic) violence, abuse, neglect or exploitation, or other types of harm in power-imbalanced relationships).

ADVICE/REPORTING

For advice on this type of harm, reporting victims or perpetrators, or referring someone to care (including shelters), call:

- Veilig Thuis. Telephone: **0800 20 00**, free of charge and always open (24 hours per day, 7 days a week). It is possible to call anonymously and/or to call for advice or information only, without reporting someone.

In case of acute danger call the emergency services at the phone number **112.**

DUTCH TRANSLATION

See here.

This fact sheet was developed by Verwey-Jonker Instituut / Kennisplatform Integratie en Samenleving in collaboration with various chain partners.

SOURCES HIDDEN WOMEN

ORGANISATIONS INVOLVED

The following organisations were involved in making this fact sheet:

- Verwey-Jonker Institute. For questions and/or remarks about the fact sheet, please email the main authors: Lisanne Drost and Eliane Smits van Waesberghe, at secr@verwey-jonker.nl
- Sterk Huis, Diane de Winter
- Bureau Tangram, Suzanne Tan
- Politie, Janine Janssen
- Movisie, Oka Storms / Wilma Schakenraad
- Landelijk Knooppunt Huwelijksdwang en Achterlating, Diny Flierman
- Veilig Thuis, Juliette Heetman
- GGD GHOR, Sandra Hamming

SOURCES

The following documents and other sources provide more information about the topic of this fact sheet:

- Drost, L., Smits van Waesberghe, E., Los, V. (2015). Opgesloten in eigen huis. Een onderzoek naar aard en omvang van verborgen vrouwen in Den Haag. Utrecht: Verwey-Jonker Instituut. www.verwey-jonker.nl/doc/2015/115002-verborgen-vrouwen-denhaag.pdf
- Drost, L., Goderie, M., Flikweert, M., & Tan, S. (2012). Leven in gedwongen isolement: Een verkennend onderzoek naar verborgen vrouwen in Amsterdam. Utrecht: Verwey-Jonker Instituut. www.verwey-jonker.nl/doc/vitaliteit/Verborgen_vrouwen_7249_web.pdf
- Informatieblad verborgen vrouwen gemeente Rotterdam: www.huiselijkgeweld.nl/doc/Informatieblad%20verborgen%20vrouwen%202016.pdf
- J. Janssen, Focus op eer. Een verkenning van eerzaken voor politieambtenaren en andere professionals, Den Haag: Boom Criminologie, 2017.
- Musa, S., Diepenbrock, E. (2013). Verborgen vrouwen: een vergeten groep. Een verkennend onderzoek naar aard, omvang en aanpak van de problematiek van verborgen vrouwen in de deelgemeente Delfshaven (Rotterdam). S.l.: Stichting Femmes For Freedom.
- Nieuwsbrief gemeente Rotterdam verborgen vrouwen, juli 2017 www.rotterdam.nl/wonen-leven/schadelijke-praktijken/NB2-verborgen-vrouwen.pdf
- www.politie.nl/themas/eergerelateerd-geweld-voor-professionals.html

This fact sheet was developed by Verwey-Jonker Instituut / Kennisplatform Integratie en Samenleving in collaboration with various chain partners. september 2020

ABANDONMENT

ALWAYS USE THE REPORTING CODE WHEN YOU ENCOUNTER A FORM OF (DOMESTIC) VIOLENCE, ABUSE, NEGLECT OR EXPLOITATION! ▶

This fact sheet is part of a series about *(domestic) violence, abuse, neglect, exploitation* and other types of harm that may be inflicted onto someone in a power-imbalanced relationship. Power-imbalanced relationships can exist with anyone, for example: an (ex-)partner, a child, a parent, a sibling, another family member, an informal or a professional carer, a friend, a flatmate or neighbour, a teacher, a colleague or supervisor, or just someone you know. These fact sheets describe different types of harm that can be inflicted in these relationships. They are meant as an add-on to the Dutch Reporting Code for these issues (English version here) and were developed for two reasons: 1) To provide professionals with an overview of all the types of harm that exist, to aid them in identifying both well-known and lesser-known types (see the Overview). 2) Signs/indicators may vary greatly by type of harm and certain types of harm require specific courses of action; the fact sheets help professionals with identifying the signs/indicators and risk factors of *each specific type* of harm and with acting appropriately when they do. Note: the general 5 steps in the Reporting Code are applicable to all types of harm in power-imbalanced relationships; the factsheets provide more guidance within these 5 steps – they are an add-on, not a replacement.

Below is a brief introduction to this topic, an overview of the signs/indicators and risk factors associated with this type of harm, and points of attention for when you encounter it.

WHAT IS ABANDONMENT?

Abandonment entails that a child, young person, woman or man is send back or left behind against his or her will in another country, while the rest of the family returns to the Netherlands. There is an involuntary break with the social environment in the Netherlands. Abandonment occurs at the initiative of spouse, parent(s) or family. An attempt is made to prevent repatriation by taking away the victim's travel or residency documents. The victim is often in a dependent and/or isolated position abroad. The motives for abandoning a young person are often a fear of westernising or to prevent misconduct (e.g., bad friends, LGBTI+). Marital problems or issues of family honour are often the basis for the abandonment of women. Abandonment may be a way to get rid of a wife and make a new marriage possible.

POSSIBLE SIGNS/INDICATORS: HOW TO IDENTIFY IT

Prevention of abandonment requires that you are alert to warning signs and discuss them with the client. The signs are non-specific. Possible signs are:

- Not possessing one's own identity documents
- Suddenly going on holiday or earlier than planned
- Afraid of going on holiday, not returning from holiday / family visit

KEY FACTS

- The estimated number of victims is between 182 and 815 per year. This is more than the number of reports (178 per year).
- The majority of the victims are between 11 and 20 years old and adult women between 20 and 35 years old.
- Abandonment occurs in countries in North Africa (Morocco, Algeria, Egypt), West Africa (Guinea, Ghana, Nigeria), East Africa (Somalia, Kenya, Ethiopia, Sudan) and the Middle East (Iraq, Iran, Afghanistan, Turkey, Pakistan, India, Saudi Arabia, Syria).
- Educational level: both young people / women with a middle or high level of education, as well as those with little or no formal schooling, can become victims.

ADVICE/REPORTING

For advice, for reporting victims or perpetrators, and/or for referring someone to care (including shelters), call:

- Veilig Thuis ("Veilig Thuis" means "Safe at Home" in Dutch, it is the organization in the Netherlands for advice on, referrals to and reporting of any type of (domestic) violence, abuse, neglect or exploitation, or other types of harm in power-imbalanced relationships). Telephone: **0800 20 00**, free of charge and always open (24 hours per day, 7 days a week).

This fact sheet was developed by Landelijk Knooppunt Huwelijksdwang en Achterlating in collaboration with various chain partners. september 2020

ABANDONMENT

- Unexplained absence
- Behaviour change: withdrawn, shy, afraid, angry
- Puberty: growing up between cultures with conflicting values / norms
- Bad friends in the eyes of parents
- Conflicts between parents and child
- Running away from home
- Care provision does not sufficiently meet the demands of parents
- Ongoing investigations into the necessity of youth care

RISK FACTORS: WHO IS EXTRA VULNERABLE?

There is an increased risk of abandonment if a girl/woman or boy/man falls into one or more risk groups:

- Children, young people or married women with and without children from a closed community where traditional ideas prevail on the role, position, education and sexuality of women and men
- Young women with a partner-dependent residence permit (and their young children)
- Young people with mild mental disabilities or who are in special education

POINTS OF ATTENTION WHEN GOING THROUGH THE 5 STEPS IN THE REPORTING CODE

For any form of (domestic) violence, abuse, neglect or exploitation, professionals in the Netherlands are required to use the Reporting Code. For general reporting code guidelines (such as the 5 steps in this code) visit the link; these are not described in this fact sheet. We do describe here points of attention that are specific to the topic of this fact sheet. These are:

- Ask for advice and always consult an expert. Preventing and responding to abandonment requires specific expertise.
- Contact the police immediately if there is an acute threat to safety.
- Talk to the partner, parents/family only AFTER the situation and risks of such a conversation have been assessed with the help of an expert.
- When the victim is already abroad: do not talk to parents, partner or family for safety reasons

It is possible to call anonymously and/or to call for advice or information only, without reporting someone.

- The Landelijk Knooppunt Huwelijksdwang en Achterlating **070 34 54 319**

If you, or the person you know, is already abroad:

- If the victim is abroad, contact the Landelijk Knooppunt Huwelijksdwang en Achterlating directly.
- If you need help abroad, please contact the Dutch embassy.

Young people:

- Young people can chat anonymously; Eva and Zahir are national expertise and treatment centres.

Acute danger:

- In case of acute danger call the emergency services at the phone number **112**.

MORE INFORMATION

See the Sources.

DUTCH TRANSLATION

See here.

This fact sheet was developed by Landelijk Knooppunt Huwelijksdwang en Achterlating in collaboration with various chain partners.

SOURCES ABANDONMENT

ORGANISATIONS INVOLVED

The following organisations were involved in making this fact sheet:

- The Landelijk Knooppunt Huwelijksdwang en Achterlating. For questions and/or remarks about the fact sheet, please email the main author: Diny Flierman, d.flierman@veiligthuishaaglanden.nl
- Augeo Foundation, Edith Geurts
- Bureau Tangram, Suzanne Tan
- CoMensha, Rik Viergever
- Fier - expertise en behandelcentrum op het terrein van geweld in afhankelijkheidsrelaties, Achille van Hees
- GGD GHOR Nederland, Annette Duenk en Sandra Hamming
- Landelijk Expertisecentrum Eergerelateerd Geweld, Korps nationale politie, Janine Janssen
- Leger des Heils Jeugdbescherming & Reclassering, Juul Polders
- Movisie, Oka Storms
- Sterk Huis, Diane de Winter
- Veilig Thuis, Sabina van der Meer
- Vereniging Vertrouwensartsen Kindermishandeling (VVAK)/ Veilig Thuis, Juliette Heetman
- Verwey-Jonker Instituut, Eliane Smits van Waesberghe

SOURCES

The following documents and other sources provide more information about the topic of this fact sheet:

Documents

- Bakker, H., Storms, O. (2015). De Meldcode bij (vermoedens van) eergerelateerd geweld. www.movisie.nl/publicatie/meldcode-vermoedens-eergerelateerd-geweld
- Bartels, E. A. C. (2005). Onderzoeksnotitie over migrantenvrouwen en kinderen die gedwongen zijn achtergelaten in landen van herkomst. (Voorstudie; No. 6). Den Haag: Advies Commissie Vreemdelingenzaken.
- Checklist EGG (eergerelateerd geweld).
- www.politie.nl/themas/eergerelateerd-geweld-voor-professionals.html
- Corringting, P. (2013). Going back 'home' Somali parents sending back their children from the Netherlands to Somalia. Master's thesis Social and Cultural Anthropology. Amsterdam: VU University.
- Herken de signalen en ga in gesprek. Tips voor professionals.
- www.huwelijksdwangenachterlating.nl/sites/www.huwelijksdwangenachterlating.nl/files/downloads/signaalkaart.pdf
- Janssen, J. (2017). Focus op eer. Een verkenning van eerzaken voor politieambtenaren en andere professionals. Den Haag: Boom criminologie. Onder meer voor relatie tussen eergerelateerd geweld en achterlating.
- Smits van Waesberghe, E., Sportel, I., Drost, E., Eijk, E. van, & Diepenbrock, E. (2014). Zo zijn we niet getrouwd. Een onderzoek naar omvang en aard van huwelijksdwang, achterlating en huwelijkse gevangenschap. Utrecht: Verweij-Jonker Instituut. www.verwey-jonker.nl/doc/vitaliteit/7414_Zo%20zijn%20we%20niet%20getrouwd_web.pdf

Websites

- Website of the Dutch Centre of forced marriage and abandonment
- www.politie.nl/themas/eergerelateerd-geweld.html
- www.nederlandwereldwijd.nl/hulp-bij-nood/huwelijksdwang

This fact sheet was developed by Landelijk Knooppunt Huwelijksdwang en Achterlating in collaboration with various chain partners.

FORCED MARRIAGE

This fact sheet is part of a series about *(domestic) violence, abuse, neglect, exploitation* and other types of harm that may be inflicted onto someone in a power-imbalanced relationship. Power-imbalanced relationships can exist with anyone, for example: an (ex-)partner, a child, a parent, a sibling, another family member, an informal or a professional carer, a friend, a flatmate or neighbour, a teacher, a colleague or supervisor, or just someone you know. These fact sheets describe different types of harm that can be inflicted in these relationships. They are meant as an add-on to the Dutch Reporting Code for these issues (English version here) and were developed for two reasons: 1) To provide professionals with an overview of all the types of harm that exist, to aid them in identifying both well-known and lesser-known types (see the Overview). 2) Signs/indicators may vary greatly by type of harm and certain types of harm require specific courses of action; the fact sheets help professionals with identifying the signs/indicators and risk factors of *each specific type* of harm and with acting appropriately when they do. Note: the general 5 steps in the Reporting Code are applicable to all types of harm in power-imbalanced relationships; the factsheets provide more guidance within these 5 steps – they are an add-on, not a replacement.

Below is a brief introduction to this topic, an overview of the signs/indicators and risk factors associated with this type of harm, and points of attention for when you encounter it.

ALWAYS USE THE REPORTING CODE WHEN YOU ENCOUNTER A FORM OF (DOMESTIC) VIOLENCE, ABUSE, NEGLECT OR EXPLOITATION! ▶

WHAT IS FORCED MARRIAGE?

A forced marriage is a marriage where the consent of the bride, groom or both is obtained through coercion by someone's parents, family or community. One or both spouses have no control over and do not consent to the marriage. There is no free partner choice or they must marry before a certain age. The marriage can be formal (legal) or informal. Family members or the community may exert pressure, for instance, emotional pressure or physical abuse. This pressure can be subtle to very compelling. Forced marriage is a form of domestic violence, can be honour-based, illegal and a criminal offence.

POSSIBLE SIGNS/INDICATORS: HOW TO IDENTIFY IT

Forced marriages are usually hidden from view. Sometimes victims show slight resistance, but out of fear, shame or loyalty to the family, they usually comply with the marital choice of the parents or community. These signs can be an indication of forced marriage:

- Behaviour change: withdrawn, afraid, angry, aggressive
- Spending less time with friends
- Does not answer phone or messages
- Suddenly different clothing
- Signs of abuse or violence
- Self-injury, suicide attempts, eating disorder
- Early or unwanted pregnancy

KEY FACTS

- The number of victims of forced marriage in the Netherlands is estimated to be between 338 and 957 per year. That is more than the number of reports of 181 per year.
- Forced marriage occurs especially among young people between 16 and 25.
- Forced marriage occurs in different ethnic and religious communities: Afghanistan, Bosnia, Bulgaria, Egypt, India, Indonesia, Iraq, Iran, Kurdish, Morocco, Pakistan, Poland, Somalia, Turkey, Suriname/Hindustani and among Sikhs and Roma
- Forced marriage takes place within family traditions, for interests' sake or solving a problem

ADVICE/REPORTING

For advice, for reporting victims or perpetrators, and/or for referring someone to care (including shelters), call:

- Veilig Thuis ("Veilig Thuis" means "Safe at Home" in Dutch, it is the organization in the Netherlands for advice on, referrals to and reporting of any type of (domestic) violence, abuse, neglect or exploitation, or other types of harm in power-imbalanced relationships). Telephone: **0800 20 00**, free of charge and always open (24 hours per day, 7 days a week).

This fact sheet was developed by Landelijk Knooppunt Huwelijksdwang en Achterlating in collaboration with various chain partners.

Warning signs at school:
- Frequent or long-term absence
- Request long family visits abroad
- Unexpected trip abroad
- Is being controlled Suddenly drop out of school
- No extracurricular activities
- Poor school performance

Warning signs at work:
- Not allowed to work or not flexible
- Frequent or long-term absence
- Poor performance
- Limited career opportunities
- Financial control
- Forced to hand over salary
- Is picked up at work

RISK FACTORS: WHO IS EXTRA VULNERABLE?

Forced marriage is more likely to occur to:
- Young women (more often) and men from closed communities where traditional ideas prevail on the role, position and sexuality of women and men.
- Migration background, honour culture, orthodox religious or high socio-economic status
- Young women and men in a highly dependent position, without a network outside the family or own income
- Persons with a dependent residence permit (see also the fact sheet about vulnerable migrants)

POINTS OF ATTENTION WHEN GOING THROUGH THE 5 STEPS IN THE REPORTING CODE

For any form of (domestic) violence, abuse, neglect or exploitation, professionals in the Netherlands are required to use the Reporting Code. For general reporting code guidelines (such as the 5 steps in this code) visit the link; these are not described in this fact sheet. We do describe here points of attention that are specific to the topic of this fact sheet. These are:
- Ask for advice and always consult an expert (see "Advice/report" below). The collective nature of marriage coercion requires specific expertise.
- Contact the police immediately if there is an acute threat to security.
- Talk to the partner, parents/family only AFTER the situation and risks of such a conversation have been assessed with the help of an expert.
- When the victim is already abroad: do not talk to parents, partner or family for safety reasons.

MORE INFORMATION

See the Sources.

It is possible to call anonymously and/or to call for advice or information only, without reporting someone.
- The Landelijk Knooppunt Huwelijksdwang en Achterlating 070 34 54 319

IF YOU, OR THE PERSON YOU KNOW, IS ALREADY ABROAD:

- If the victim is abroad, contact the Landelijk Knooppunt Huwelijksdwang en Achterlating directly.
- If you need help abroad, please contact the Dutch embassy.

YOUNG PEOPLE:

- Young people can chat anonymously; Eva and Zahir are national expertise and treatment centres.

ACUTE DANGER:

- In case of acute danger call the emergency services at the phone number **112**.

DUTCH TRANSLATION

See here.

This fact sheet was developed by Landelijk Knooppunt Huwelijksdwang en Achterlating in collaboration with various chain partners. september 2020

SOURCES FORCED MARRIAGE

ORGANISATIONS INVOLVED

The following organisations were involved in making this fact sheet:

- The Landelijk Knooppunt Huwelijksdwang en Achterlating. For questions and/or remarks about the fact sheet, please email the main author: Diny Flierman, d.flierman@veiligthuishaaglanden.nl
- Augeo Foundation, Edith Geurts
- Bureau Tangram, Suzanne Tan
- CoMensha, Rik Viergever
- Fier - expertise and treatment centre in the field of violence in power-imbalanced relationships, Achille van Hees
- GGD GHOR Netherlands, Annette Duenk and Sandra Hamming
- Landelijk Expertisecentrum Eergerelateerd Geweld, Korps nationale politie, Janine Janssen
- Leger des Heils Jeugdbescherming & Reclassering, Juul Polders
- Movie, Oka Storms
- Sterk Huis, Diane de Winter
- Veilig Thuis, Sabina van der Meer
- Vereniging Vertrouwensartsen Kindermishandeling (VVAK)/ Veilig Thuis, Juliette Heetman
- Verwey-Jonker Instituut, Eliane Smits van Waesberghe

SOURCES

The following documents and other sources provide more information about the topic of this fact sheet:

Documents

- Bakker, H. & Noor, S. (2015). Factsheet huwelijksdwang. Kennisplatform Integratie en Samenleving. www.kis.nl/publicatie/fact sheet-huwelijksdwang
- Bakker, H., Storms, O. (2015). De Meldcode bij (vermoedens van) eergerelateerd geweld. www.movisie.nl/publicatie/meldcode-vermoedens-eergerelateerd-geweld
- Checklist EGG (eergerelateerd geweld). www.politie.nl/themas/eergerelateerd-geweld-voor-professionals.html
- Herken de signalen en ga in gesprek. Tips voor professionals. www.huwelijksdwangenachterlating.nl/sites/www.huwelijksdwangenachterlating.nl/files/downloads/signaalkaart.pdf
- Janssen, J. (2017). Focus op eer. Een verkenning van eerzaken voor politieambtenaren en andere professionals. Den Haag: Boom criminologie. Onder meer voor relatie tussen eergerelateerd geweld en huwelijksdwang.
- Ministerie Sociale Zaken en Werkgelegenheid (2017). Handreiking kindhuwelijken en informele huwelijken. www.rijksoverheid.nl/documenten/publicaties/2017/06/30/handreiking-kindhuwelijke-en-informele-huwelijken
- Smits van Waesberghe, E., Sportel, I., Drost, E., Eijk, E. van, & Diepenbrock, E. (2014). Zo zijn we niet getrouwd. Een onderzoek naar omvang en aard van huwelijksdwang, achterlating en huwelijkse gevangenschap. Utrecht: Verwey-Jonker Instituut. www.verwey-jonker.nl/doc/vitaliteit/7414_Zo%20zijn%20we%20niet%20getrouwd_web.pdf
- Warning signs of victim of forced marriage. westyorkscb.proceduresonline.com/pdfs/warning_signs_diagram.pdf

Websites

- Website Landelijk Knooppunt Huwelijksdwang en Achterlating: www.huwelijksdwangenchterlating.nl
- www.rijksoverheid.nl/onderwerpen/huwelijksdwang/huwelijksdwang-voorkomen
- www.huiselijkgeweld.nl/dossiers/huwelijksdwang
- www.fier.nl/kennis-en-expertise/eergerelateerd-geweld/huwelijksdwang
- www.augeo.nl/Huwelijksdwang
- www.augeo.nl/thema/huwelijksdwang/leren-over-huwelijksdwang
- www.politie.nl/themas/eergerelateerd-geweld.html
- www.nederlandwereldwijd.nl/hulp-bij-nood/huwelijksdwang

FINANCIAL ABUSE

ALWAYS USE THE REPORTING CODE WHEN YOU ENCOUNTER A FORM OF (DOMESTIC) VIOLENCE, ABUSE, NEGLECT OR EXPLOITATION! ▶

This fact sheet is part of a series about *(domestic) violence, abuse, neglect, exploitation* and other types of harm that may be inflicted onto someone in a power-imbalanced relationship. Power-imbalanced relationships can exist with anyone, for example: an (ex-)partner, a child, a parent, a sibling, another family member, an informal or a professional carer, a friend, a flatmate or neighbour, a teacher, a colleague or supervisor, or just someone you know. These fact sheets describe different types of harm that can be inflicted in these relationships. They are meant as an add-on to the Dutch Reporting Code for these issues (English version here) and were developed for two reasons: 1) To provide professionals with an overview of all the types of harm that exist, to aid them in identifying both well-known and lesser-known types (see the Overview). 2) Signs/indicators may vary greatly by type of harm and certain types of harm require specific courses of action; the fact sheets help professionals with identifying the signs/indicators and risk factors of *each specific type* of harm and with acting appropriately when they do. Note: the general 5 steps in the Reporting Code are applicable to all types of harm in power-imbalanced relationships; the factsheets provide more guidance within these 5 steps – they are an add-on, not a replacement.

Below is a brief introduction to this topic, an overview of the signs/indicators and risk factors associated with this type of harm, and points of attention for when you encounter it.

WHAT IS FINANCIAL ABUSE?

Financial abuse is the unwanted and unauthorised use of money and/or goods and/or other possessions of another person by someone in the household. The gravity varies betweenthe victim not realising what's happening, to a criminal offence. This fact sheet is specifically about financial abuse that victimises the elderly.

Examples of financial abuse include: shopping for oneself with someone else's debit card, transferring sums of money to one's own account, theft of - valuable and precious - property, debit card fraud, forced changes in someone's will or mortgage, sale of property, abuse of authorisations, purchases in the name of the victim, keeping someone on a 'short financial leash', i.e. limiting someone's financial freedom.

WHO ARE THE PERPETRATORS?

The perpetrators can be family members, such as (ex-)partners and (grand)children, but also house friends. In addition, they can also be persons the victim is dependent of, such as professional care workers or volunteers.

FACTS AND FIGURES

The 2016 Health Monitor i shows that nationwide, 1.1% of surveyed elderly people report to have experienced financial abuse. There are currently 3.1 million elderly people in the Netherlands, so this computes to 34,100 elderly people. Regional numbers ranged from 0.7 - 2.0%.

In a prevalence study on elder abuse by Regioplan ii one in twenty elderly people experience some form of elder abuse from the age of 65 years onwards. The most commonly reported form of elder abuse by elderly people themselves is financial abuse (3% of all elderly people). When looking at financial abuse *in the past* year, the study found an incidence of 0.9%.

The results of the studies are very similar. Both studies use the same definition for financial abuse and the same age limit of 65 years.

MORE INFORMATION

See the Sources.

FINANCIAL ABUSE

POSSIBLE SIGNS/INDICATORS:

The signs/indications for financial abuse are as follows:

- Disappearance of money, goods or valuables
- Unexplained cash withdrawals or charges
- Sudden lack of money
- Delays on payments: rent, energy, bills
- Disconnected from gas and electricity
- Increasing number of creditors
- Letters from collection agencies
- Sudden requests for changes of wills in the presence of a family member (the suspected perpetrator)
- Avoiding or refusing to provide information about financial circumstances by an elder or caregiver (the suspected perpetrator)
- Resistance and/or verbal abuse (by the suspected perpetrator) when suspicions are discussed
- Verbal violence or threats (by the suspected perpetrator) against the victim when he or she resists
- Addiction or financial problems with the suspected perpetrator
- Neglected environment and/or appearance with the victim
- Absence of food in the house, signs of malnutrition

N.B. These last two signals can also indicate other problems, like mental decline and a poor social network and/or self-neglect (when someone is no longer able to take care of him-/herself).

RISK FACTORS: WHO IS EXTRA VULNERABLE?

The most important risk factors for financial abuse are the degree of (care) dependency, the relationship that exists between the perpetrator and the victim and the degree of loyalty there is in that relationship, and the circumstances of the perpetrator, such as debts, psychological problems and addiction. Another factor is that elderly people can be too trusting, and have not properly arranged their financial affairs before their health deteriorates to such an extent that they are no longer able to take care of their finances themselves. There is a checklist iii that elderly people can fill in themselves in this regard. On the basis of this checklist, they can decide, potentially in consultation with their relatives, the bank or a notary, whether they want or need to make further arrangements in order to reduce the risk of financial abuse in the future.

The risk factors can broadly be categorized into:

- **Ignorance**: No knowledge and skills of and in digital banking, and consequently leaving financial and administrative matters to others; too little up-to-date knowledge of increasingly complex regulations.
- **Loyalty and family ties:** Through loyalty, family ties, shame, guilt, insecurity (am I right in seeing this?) and/or fear of diminished contact the elderly person does not discuss his/her concerns and signs of abuse with a professional or volunteer. There are harmonious and high-conflict families. Financial abuse partly depends on how family members interact with and relate to one another. This has to do with loyalty and attachment. Parents can allow their children to exploit them for a longer period of time because of feelings of guilt about having failed their children in the past (e.g., because of illness).
  There are children who believe that they have not been given what they are due, that they have been neglected, or abused, by their parents. They may take revenge by financially damaging their parent(s).
  Family disputes can also arise because of claims that children believe they have. With family feuds it often happens that one of the children starts taking up a central role and excludes the other family members. This carries the risk of financial abuse of the parent(s).

ADVICE/REPORTING

For advice, for reporting victims or perpetrators, and/or for referring someone to care (including shelters), call:

- Veilig Thuis ("Veilig Thuis" means "Safe at Home" in Dutch, it is the organization in the Netherlands for advice on, referrals to and reporting of any type of (domestic) violence, abuse, neglect or exploitation, or other types of harm in power-imbalanced relationships). Telephone: **0800 20 00**, free of charge and always open (24 hours per day, 7 days a week). It is possible to call anonymously and/or to call for advice or information only, without reporting someone.

In case of acute danger call the emergency services at the phone number **112**.

DUTCH TRANSLATION

See here.

- **Physical limitations:** When an elderly person has limitations in terms of hearing, vision and/or mobility, they can run an increased risk because they are not (or no longer) able to (properly) look after their own financial interests.
- **Social limitations:** People with a small social network and few contacts outside the home are at increased risk of becoming victims of financial abuse. The desire for contact sometimes results in a lack of critical assessment about the person with whom the contact is made. This carries the risk that they will hire or engage with an unreliable person, and that this person will abuse their dependency and needs.
- **Psychological problems:** Elderly people are often still mourning the loss of a partner and independence, with depressive feelings influencing their energy to manage their finances properly. In addition, a risk factor is a person's mental decline.
- **Cognitive limitations:** The elderly are more likely to experience financial abuse because they no longer have an overview and lack the skills to control their own finances.

POINTS OF ATTENTION WHEN GOING THROUGH THE 5 STEPS IN THE REPORTING CODE

For any form of (domestic) violence, abuse, neglect or exploitation, professionals in the Netherlands are required to use the Reporting Code. For general reporting code guidelines (such as the 5 steps in this code) visit the link; these are not described in this fact sheet. We do list points of attention in going through the 5 steps that are specific to the topic of this fact sheet. These are:

- Banks and notaries already play an important role in preventing financial abuse.
- Assigning an administrator/custodian and/or reporting the abuse to Veilig Thuis or the police are (extreme) options to stop financial abuse.
- When it comes to theft by fellow clients in an intramural institution, the mental state of the perpetrator should always be taken into account. Psychogeriatric problems, such as dementia, can underlie the behaviour. Objects often reappear.
- This fact sheet has focused on financial abuse among the elderly, however, financial abuse can also occur among other groups of vulnerable citizens such as people with intellectual disabilities or other people who have a strongly power-imbalanced relationship with the perpetrator.

Financial exploitation can also be committed by a professional or by a volunteer. Both fall under the Dutch Quality, Complaints and Disputes in Health Care Act (Wkkgz) and serious incidents must be reported to the Health Care and Youth Care Inspectorate (IGJ).

SOURCES FINANCIAL ABUSE

ORGANISATIONS INVOLVED

The following organisations were involved in making this fact sheet:

- Movisie. For questions and/or remarks about the fact sheet, please email the main author: Nico van Oosten, N.vanOosten@movisie.nl
- Veilig Thuis. Marianne van der Krans, Cathelijne Berkvens, Sigrid van den Boer, Anne-Marie Raat, Laura van der Voorn en Gerda Rosman

SOURCES

The following documents and other sources provide more information about the topic of this fact sheet:

- www.volksgezondheidenzorg.info/onderwerp/sociale-omgeving/ouderenmishandeling#node-financieel-benadeeld-de-thuissituatie-ggd-regio (2016)
- Bakker, L., Witkamp, B., Timmermans, M., Janssen, J., Lindenberg, J. (2018). Aard en omvang ouderenmishandeling. Amsterdam: Regioplan, Avans Hogeschool, Leyden Academy on Vitality and Ageing. www.regioplan.nl/publicaties/slug/type/rapporten/slug/aard_en_omvang_ouderenmishandeling
- Checklist voor ouderen ten behoeve van het voorkomen vanfinanciële uitbuiting. April 2016. Ministerie van Volksgezondheid, Welzijn en Sport in samenwerkingmet de Brede Alliantie 'Veilig financieel ouder worden'. www.aanpak-ouderenmishandeling.nl/doc/Checklist-ouder-voorkomen-van-financiele-uitbuiting.pdf

BULLYING

ALWAYS USE THE REPORTING CODE WHEN YOU ENCOUNTER A FORM OF (DOMESTIC) VIOLENCE, ABUSE, NEGLECT OR EXPLOITATION! ▶

This fact sheet is part of a series about *(domestic) violence, abuse, neglect, exploitation* and other types of harm that may be inflicted onto someone in a power-imbalanced relationship. Power-imbalanced relationships can exist with anyone, for example: an (ex-)partner, a child, a parent, a sibling, another family member, an informal or a professional carer, a friend, a flatmate or neighbour, a teacher, a colleague or supervisor, or just someone you know. These fact sheets describe different types of harm that can be inflicted in these relationships. They are meant as an add-on to the Dutch Reporting Code for these issues (English version here) and were developed for two reasons: 1) To provide professionals with an overview of all the types of harm that exist, to aid them in identifying both well-known and lesser-known types (see the Overview). 2) Signs/indicators may vary greatly by type of harm and certain types of harm require specific courses of action; the fact sheets help professionals with identifying the signs/indicators and risk factors of *each specific type* of harm and with acting appropriately when they do. Note: the general 5 steps in the Reporting Code are applicable to all types of harm in power-imbalanced relationships; the factsheets provide more guidance within these 5 steps – they are an add-on, not a replacement.

Below is a brief introduction to this topic, an overview of the signs/indicators and risk factors associated with this type of harm, and points of attention for when you encounter it.

WHAT IS BULLYING?

Bullying is systematic aggressive behaviour in which one or more persons in a dominant position try to harm another person. Bullying can take the form of physical, verbal or psychological behaviour. Systematic exclusion is also included in the definition of bullying. Bullying often takes place in a group, for example in the classroom, or digitally, but can also take place in other places such as the sports club.

Bullying can lead to all kinds of negative consequences, including psychosomatic complaints, anxiety and depression, low self-confidence, loneliness, poor school performance and absenteeism. Some of these consequences can continue into adulthood. Besides the victims of bullying, perpetrators of bullying can also have problems in their development. The unadjusted behaviour and lack of empathy increases the chance of problematic behaviour in later life.

FACTS AND FIGURES

Every year, about 10% of primary-school pupils are victims of bullying. In secondary education this is about 8%.

Girls are bullied about as often as boys.

Homosexual, lesbian and transgender young people are twice as likely to be bullied and are also **much more likely to experience violent behaviour**. Various school programs are effective in reducing bullying.

MORE INFORMATION

See the Sources and:

- www.pestweb.nl
- the JGZ guideline Bullying
- *Pesten op school*. Vermande, M., van der Meulen, M. & Reijntjes, A. (Red.) Boom Uitgevers Amsterdam.
- Possible interventions

DUTCH TRANSLATION

See here.

This fact sheet was developed by TNO in collaboration with various chain partners.

BULLYING

POSSIBLE SIGNS/INDICATORS: HOW TO IDENTIFY IT

The most important signs that may indicate that someone is being bullied are listed here below. Professionals can ask about these complaints and about someone's social functioning in general in order to start a conversation about bullying:

- Psychosomatic complaints such as abdominal pain, headache, bedwetting, eczema
- Anxious or depressed behaviour
- Withdrawn, quiet, sub-assertive behaviour
- Not enjoying school, not wanting to go to school
- Not having friends

RISK FACTORS: WHO IS EXTRA VULNERABLE?

The most important factors that can make children and young people more vulnerable to becoming a **victim** of bullying are:

- Being anxious, depressed, withdrawn or shy
- Being awkward physically or stuttering
- Being overweight, having ADHD or having autism
- Having few or no good friends
- Being LHBTQ
- Being non-average in terms of intelligence (gifted children or children with a mild intellectual disability)

In terms of **perpetrators**: children of parents with an aggressive/ authoritarian parenting style are more likely to become perpe-trators of bullying.

5-STEP PLAN FOR DEALING WITH BULLYING

When it is suspected that a child is being bullied, the child should be explicitly asked whether he or she is being bullied. If the child requires individual attention, an individual intervention can be implemented. Bullying is often a problem of the group and usually needs to be solved there, also in case of cyber-bullying. It is therefore important to involve parents and the school so that they take responsibility in solving the bullying problem. The Richtlijn voor Jeugdgezondheidszorg (the Youth Health Care Directive) recommends the following 5-step plan:

1. Estimation of needs of the child
2. Consultation with parents
3. Consultation with school, sports club or other organisation
4. Refer for individual help
5. Follow-up

This fact sheet was developed by TNO in collaboration with various chain partners.

SOURCES BULLYING

ORGANISATIONS INVOLVED

The following organisations were involved in making this fact sheet:

- TNO. For questions and/or remarks about the fact sheet, please email the main author: Minne Fekkes, minne.fekkes@tno.nl.
- Augeo, Edith Geurts
- Radboud umc, Karin van Rosmalen-Nooijens
- GGD GHOR, Sandra Hamming
- Veilig Thuis / VVAK, Juliette Heetman

SOURCES

The following documents and other sources provide more information about the topic of this fact sheet:

- www.pestweb.nl
- the JGZ guideline on Bullying www.ncj.nl/richtlijnen/alle-richtlijnen/richtlijn/pesten
- for interventions: www.uu.nl/sites/default/files/eindrapport-wat-werkt-tegen-pesten.pdf
- Vermande, M., van der Meulen, M. & Reijntjes, A. (Red.) *Pesten op school,* Boom Uitgevers Amsterdam.

SELF-HARM

ALWAYS USE THE REPORTING CODE WHEN YOU ENCOUNTER A FORM OF (DOMESTIC) VIOLENCE, ABUSE, NEGLECT OR EXPLOITATION! ▶

This fact sheet is part of a series about *(domestic) violence, abuse, neglect, exploitation* and other types of harm that may be inflicted onto someone in a power-imbalanced relationship. Power-imbalanced relationships can exist with anyone, for example: an (ex-)partner, a child, a parent, a sibling, another family member, an informal or a professional carer, a friend, a flatmate or neighbour, a teacher, a colleague or supervisor, or just someone you know. These fact sheets describe different types of harm that can be inflicted in these relationships. They are meant as an add-on to the Dutch Reporting Code for these issues (English version here) and were developed for two reasons: 1) To provide professionals with an overview of all the types of harm that exist, to aid them in identifying both well-known and lesser-known types (see the Overview). 2) Signs/indicators may vary greatly by type of harm and certain types of harm require specific courses of action; the fact sheets help professionals with identifying the signs/indicators and risk factors of *each specific type* of harm and with acting appropriately when they do. Note: the general 5 steps in the Reporting Code are applicable to all types of harm in power-imbalanced relationships; the factsheets provide more guidance within these 5 steps – they are an add-on, not a replacement.

Below is a brief introduction to this topic, an overview of the signs/indicators and risk factors associated with this type of harm, and points of attention for when you encounter it.

WHAT IS SELF-HARM?

Self-harm is the self-inflicted physical injury or damage in a repeating pattern, without conscious suicidal intent. It is often an expression of intense, overwhelming emotions or feelings of emptiness that are hard to bear for those who damage themselves. Usually it has little to do with suicidality; self-harm is a survival strategy for people when they lack better alternatives.

POSSIBLE SIGNS/INDICATORS: HOW TO IDENTIFY IT

It is often not visible that someone is harming him- or herself. Specific signs are scars of (e.g.) cuts and burns, wearing covering clothing (e.g. long sleeves in summer) and not participating in activities that require a person to change clothes, such as sports and swimming. There may also be general signs that someone is not doing well, such as withdrawal, emotional outbursts and a deterioration in concentration and performance.

FACTS AND FIGURES

- The prevalence of self-harm among the adult population is 5.5%, among adolescents it is 15% and among youths it is 4%.
- Women are 1.5 times more likely than men to self-harm.
- The age of onset is usually between 12 and 14 years; the peak is in adolescence.
- The most common methods are cutting, scratching and burning.
- There are no figures on the course or prognosis of self-harming behaviour. In practice, the majority of people stop self-harming before the age of 30.

ADVICE AND HELP

For more information and advice on self-harm, please contact:

- Your general practitioner for basic care and referral
- the Stichting Zelfbeschadiging (the "Self-harm Foundation") for getting in touch with people with lived experience
- Fivoor (info@fivoor.nl, 088 02 82 220, ask for Nienke Kool) for professional and specific questions

Usually, self-harm is not a matter for Veilig Thuis ("Veilig Thuis" means "Safe at Home" in Dutch, it is the organization in the Netherlands for advice on, referrals to and reporting of any type

This fact sheet was developed by Fivoor in collaboration with various chain partners. september 2020

SELF-HARM

RISK FACTORS: WHO IS EXTRA VULNERABLE?

There is no single reason why people harm themselves. Examples of risk factors that *can* make people vulnerable to self-harming behaviour are:

- Early childhood trauma (child abuse, whatever form it takes).
- The lack of support in difficult phases of life.
- Perfectionism and impulsivity.
- Being bullied.
- Being unable to express feelings.
- Slight intellectual disability.
- All kinds of psychiatric disorders such as psychotic, mood and personality disorders, addiction, eating disorder, post-traumatic stress disorder and autism

.

COURSE OF ACTION: WHAT TO DO WHEN THERE ARE (SUSPICIONS OF) SELF-HARM?

Someone who harms himself is often ashamed and anxious about being open towards others. Therefore, in the first contact moment with someone who harms him- or herself, it is essential that there is an open, empathic, non-judgmental attitude, with understanding of the underlying suffering. The contact moment should be as equal as possible and be aimed at building a collaboration.

In addition:

- Discuss (a suspicion of) self-harm directly with the person who is harming him- or herself.
- If someone has just harmed themselves, check together if (medical) care is needed for the wound(s) and agree who will provide that care.
- Usually there is no suicidality, but ask about it. This can also be done by directly asking: do you want to die? If this is the case, see also the "Handreiking 113 zelfmoordpreventie".
- Search together for meaning and follow-up steps, sometimes this can entail contact with people with lived experience (Stichting Zelfbeschadiging) and/or a few coaching conversations, but it may also entail a long therapeutic process. If referral is needed, supervise this process and stay involved in the background until someone has landed well (a warm transfer).
- Keep an eye for the impact on family and loved ones, if they are in the picture. Support them too.

MORE INFORMATION

See the Sources.

of (domestic) violence, abuse, neglect or exploitation, or other types of harm in power-imbalanced relationships) and the Reporting Code. However, in the following situations it is important to contact Veilig Thuis at **0800 20 00**:

- The cause of self-harm is related to an unsafe domestic situation/domestic violence and/or child abuse. It is quite possible that this will only become clear after a few conversations, because a trusting relationship needs to be established before people speak about these issues. If it is clear that there is an unsafe home, the reporting code must be followed.
- There may be doubts as to whether the injuries were self-made or made by someone else. If you have any doubts about this, discuss this with the person in question. If there is violence by others, contact Veilig Thuis for advice or reporting. The following signals may indicate violence by others:
  - when someone's story does not match their injuries,
  - injuries have been inflicted in places that are difficult to access for the person in question.
- If the person who self-harms is an adult, it is important to check whether they have children. In that case, consider a consultation with Veilig Thuis.

In case of acute danger call the emergency services at the phone number **112**.

This fact sheet was developed by Fivoor in collaboration with various chain partners. september 2020

SOURCES SELF-HARM

ORGANISATIONS INVOLVED

The following organisations were involved in making this fact sheet:

- Fivoor, forensic and intensive psychiatric care. For questions and/or remarks about the fact sheet, please email the main author: Nienke Kool, n.kool@fivoor.nl , nurse and researcher in the area of self-harm.
- Stichting Zelfbeschadiging, organisatie voor en door mensen die zichzelf beschadigen en hun omgeving, Dwayne Meijnckens, dwayne@zelfbeschadiging.nl / www.zelfbeschadiging.nl
- Veilig Thuis, Juliette Heetman, heetman@xs4all.nl
- Nadine Callens, nadine.callens1@telenet.be, author of the book "zelfverwonding bij jongeren, een gids voor leerkrachten, leerlingenbegeleiders, ouders en vrienden", uitgeverij Garant-Maklu.

SOURCES

The following documents and other sources provide more information about the topic of this fact sheet:

- Handreiking 113 zelfmoordpreventie voor de huisartsenpraktijk. www.113.nl/sites/default/files/113/preventie/Handreiking%20113%20zefmoordpreventie%20huisartsen.pdf
- Nienke Kool, 2011. Bejegening. – te verkrijgen op verzoek bij de hoofdauteur Nienke Kool.
- Landelijke Stichting Zelfbeschadiging. Alternatieven voor zelfbeschadiging. www.zelfbeschadiging.nl/alternatieven-voor-zelfbeschadiging zelfbeschadiging.nl/wp-content/uploads/2016/07/Alternatieven-voor-zelfbeschadiging-1.pdf (bekeken op 16 aug 2018).
- Landelijke Stichting Zelfbeschadiging. Tips voor lotgenoten. – te verkrijgen op verzoek bij de hoofdauteur Nienke Kool.
- Landelijke Stichting Zelfbeschadiging. Zelfbeschadiging: wat kun jij doen om te helpen? www.zelfbeschadiging.nl/wat-kun-jij-doen-om-te-helpen/
- zelfbeschadiging.nl/wp-content/uploads/2017/01/Zelfbeschadiging-wat-kun-jij-doen-om-te-helpen-2.pdf (bekeken op 16 aug 2018)
- www.zelfbeschadiging.nl
- www.sameninmijnschoenen.nl A website to provide easy access information about self-harm for young people who self-harm, next of kin and professionals.

LINE-CROSSING SEXUAL BEHAVIOUR AMONG YOUNG PEOPLE

ALWAYS USE THE REPORTING CODE WHEN YOU ENCOUNTER A FORM OF (DOMESTIC) VIOLENCE, ABUSE, NEGLECT OR EXPLOITATION! ▶

This fact sheet is part of a series about *(domestic) violence, abuse, neglect, exploitation* and other types of harm that may be inflicted onto someone in a power-imbalanced relationship. Power-imbalanced relationships can exist with anyone, for example: an (ex-)partner, a child, a parent, a sibling, another family member, an informal or a professional carer, a friend, a flatmate or neighbour, a teacher, a colleague or supervisor, or just someone you know. These fact sheets describe different types of harm that can be inflicted in these relationships. They are meant as an add-on to the Dutch Reporting Code for these issues (English version here) and were developed for two reasons: 1) To provide professionals with an overview of all the types of harm that exist, to aid them in identifying both well-known and lesser-known types (see the Overview). 2) Signs/indicators may vary greatly by type of harm and certain types of harm require specific courses of action; the fact sheets help professionals with identifying the signs/indicators and risk factors of *each specific type* of harm and with acting appropriately when they do. Note: the general 5 steps in the Reporting Code are applicable to all types of harm in power-imbalanced relationships; the factsheets provide more guidance within these 5 steps – they are an add-on, not a replacement.

Below is a brief introduction to this topic, an overview of the signs/indicators and risk factors associated with this type of harm, and points of attention for when you encounter it.

WHAT IS LINE-CROSSING SEXUAL BEHAVIOUR BETWEEN YOUNGSTERS?

Line- crossing sexual behaviour refers to behaviour or approaches that are sexual in nature and cross the boundaries of one or more of the people involved. This may or may not be physical (De Haas, 2012).

To determine whether or not sexual behaviour crosses someone's boundaries, we start from a number of fundamental criteria or social values, based on rights, legislation and regulation. The point of view here is that of the person responsible for the behaviour (Frans, De Wilde, Janssens, van Berlo & Storms, 2016): (1) mutual consent; (2) voluntary action; (3) equivalence; (4) appropriate to age or development or age; (5) appropriate to the context; (6) self-respect (see also the Sensoa Flagsystem© , Frans & Franck; 2010; 2014).

This fact sheet is concerned with sexual behaviours among children and adolescents. In the case of positive and acceptable sexual behaviour of children and adolescents, all criteria are met. When one or more of the criteria are not met, this is defined as sexual behaviour that crosses boundaries. We speak of sexual abuse for any form of sexual behaviour that crosses boundaries in a verbal or physical sense, whether intentional or unintentional, which clearly does not have mutual consent; and/or which is somehow enforced and/or where the victim is much younger or

NUMBERS

The study Seks onder je 25e by Rutgers (De Graaf et al. , 2017) shows that 2% of boys and 11% of girls are sometimes forced into sexual acts that they did not want to engage in.

MORE INFORMATION

See the Sources and these websites:

- www.act4respect.nl
- www.rutgers.nl
- www.atria.nl
- www.vlaggensysteem.nl

in a dependent relationship (Frans et a. , 2016; Frans & Franck, 2014).

Hands-off and hands-on

When discussing line-crossing sexual behaviour, we distinguish between so-called hands-off and hands-on behaviour. Hands-off behaviour does not involve physical contact and involves, for example, unwanted and/or offensive sexual remarks or sexting (making and sending sexually-tinged messages or spicy photos and videos) or being forced to watch porn. In the case of hands-on behaviour, there is physical contact. This concerns sexual touching, intrusion, ranging from kissing (forced tongue kissing) to rape, the most serious form of line-crossing sexual behaviour, which is a form of sexual assault.

Young people experiment and sometimes cross boundaries

When young people's sexual boundaries are crossed it is often by peers.

A part of the developmental stage in young people's lives is (sexual) experimentation. Sometimes young people go beyond their own boundaries or those of another. They are sometimes insufficiently aware of their own limits and those of others, they do not know how to recognise these limits in someone else, or they find it difficult to say out loud what they like or dislike. Group pressure can also play a role; and/or there can be the influence of alcohol or other drugs.

(Professional) educators and parents therefore have the task of guiding young people in this, and to make young people aware of the importance of understanding sexual preferences and boundaries.

AT-RISK GROUPS: AMONG WHICH GROUPS IS IT MORE PREVALENT?

At-Risk groups for line-crossing sexual behaviour between young people:

- Girls are generally more at risk than boys.
- Lesbian, homosexual, bisexual or transgender youth.
- Young people with a low level of education
- Young people with a disability (physical or mental, also a mild mental disability).
- Young people staying in institutions.
- Young people with negative childhood experiences of physical abuse, emotional neglect and/or sexual abuse.

RISK FACTORS: WHO IS EXTRA VULNERABLE?

In addition to the risk groups mentioned above, there are also *individual and relational risk factors*. These include a lack of knowledge and skills among children and adolescents to assess sexual behaviour, a relationship with parents that is lacking in trust, and previous experiences with sexual behaviour that crossed boundaries (so-called revictimization). Young people with a low self-esteem, ADHD, or an autism spectrum disorder that results in insufficient awareness of what another person is experiencing are also at greater risk.

ADVICE/REPORTING

When the sexual violence is recent, contact:

- The Centrum Seksueel Geweld (CSG) (this name means "Centre for Sexual Violence" in Dutch) is the expertise centre of the Netherlands for victims of sexual violence in the acute phase (<7 days). At CSG, a team of doctors, nurses, police, psychologists, social workers and sexologists work together to provide specialist care to victims of assault and rape. Call 0800-0188.

When the sexual violence is less recent, contact:

- Veilig Thuis ("Veilig Thuis" means "Safe at Home" in Dutch, it is the organization in the Netherlands for advice on, referrals to and reporting of any type of (domestic) violence, abuse, neglect or exploitation, or other types of harm in power-imbalanced relationships). Telephone: **0800 20 00**, free of charge and always open (24 hours per day, 7 days a week). It is possible to call anonymously and/or to call for advice or information only, without reporting someone.

In case of acute danger call the emergency services at the phone number **112**.

DUTCH TRANSLATION

See here.

This fact sheet was developed by Movisie in collaboration with various chain partners. september 2020

LINE-CROSSING SEXUAL BEHAVIOUR AMONG YOUNG PEOPLE

Social norms and developments that increase the incidence of sexual behaviour that crosses boundaries include: traditional gender roles, gender stereotypes, double standards and changing realities. What also plays a role in this regard is that sexuality is more prevalent in the mass media today (Römkens, 2017) and that 'biological maturation' starts earlier in children/young people now than in the past (Storms & Doornink, 2016). Another social development of relevance is that young people today show more experimental behaviour and (ab)use alcohol and drugs more often.

GUIDELINES FOR PROFESSIONALS

The Sensoa Flagsystem© (Frans & Franck, 2010; 2014) offers professional educators tools to adequately assess sexual behaviour, to opening it up to discussing and to react appropriately. This is done on the basis of six criteria to determine whether the behaviour is healthy or crosses boundaries: consent, voluntary action, equivalence, age or development adequacy, context adequacy and self-respect. Specific attention is paid to gender and cultural aspects; as well as to children and young people with disabilities and/or trauma (Frans et al. , 2016).

What does the law say?

Sex with a minor (defined for this law as <16 years of age) is punishable in principle, even if there was mutual consent. However, between the ages of 12 and 16, the Public Prosecution Service asks the young person for his or her opinion, 'if possible' and in certain cases (art. 167a of the Code of Criminal Procedure). A 17-year-old boy who has sexual contact with a 15-year-old girl who has no objection to this, will most likely not be charged with an offence. Sex with a child under 12 is always punishable.

NOTE

For all forms of domestic violence and child abuse, the Dutch Reporting Code (English version here) for these issues must be applied in the Netherlands by all groups of professionals named in the Reporting Code law. Sexual behaviour between young people that crosses boundaries does not fall under the definition of domestic violence or child abuse in the Netherlands and therefore, it is not legally required to use the reporting code when you encounter it as a professional. However, the reporting code may be used. Because we feel it is a useful guideline for professionals for this type of harm as well, and because it is important that professionals (e.g. general practitioners) can identify this type of harm and take the right steps, this factsheet was compiled.

ORGANISATIONS INVOLVED

The following organisations were involved in making this fact sheet:

- Movisie. For questions and/or remarks about the fact sheet, please email the main author: Wilma Schakenraad, w.schakenraad@movisie.nl
- Veilig Thuis
- Kennisinstituut voor Emancipatie en Vrouwengeschiedenis (Atria)

SOURCES

The following documents and other sources provide more information about the topic of this fact sheet:

Publications

- Berlo van, W. & Beek I. van (2015). Whitepaper Seksuele grensoverschrijding en seksueel geweld. Feiten en cijfers Utrecht: Rutgers en Movisie.
- Graaf, H. de, Borne, M. van den, Nikkelen, S., Twisk, D., & Meijer, S. (2017). Seks onder je 25e. Utrecht / Amsterdam: Rutgers / Soa Aids Nederland.
- Haas, S. de (2012). Seksueel grensoverschrijdend gedrag onder jongeren en volwassenen in Nederland. Tijdschrift voor Seksuologie, 36-2, p. 136-145.
- Höing, M., & Janssen, J. (2017). Seksueel grensoverschrijdend gedrag. In: Höing, M., & Janssen, J., Boer, A., & Liebregts, M. (red.). Bespreekbaar maken van seksualiteit en intimiteit. Handboek voor professionals in zorg en welzijn. Bussum: Coutinho.
- Nationaal Rapporteur Mensenhandel en Seksueel Geweld tegen Kinderen (2014). Op goede grond. De aanpak van seksueel geweld tegen kinderen. Den Haag: Nationaal Rapporteur.
- Römkens, R. (2017). Factsheet Online seksuele intimidatie. Amsterdam: Atria. Zie: www.atria.nl/sites/atria/files/atoms/files/factsheet-cybergeweld-onlineversiedef.pdf
- Storms, O. & Doornink, N. (2016). Vlaggensysteem: Reageren op seksueel (grensoverschrijdend) gedrag van kinderen en jongeren. Effectieve sociale interventies en Effectieve interventies huiselijk en seksueel geweld. Utrecht: Movisie.

Websites

- seksonderje25e.nl (Rutgers)
- Kennisdossier seksuele grensoverschrijding van Rutgers: www.rutgers.nl/feiten-en-cijfers/kennisdossiers/kennisdossier-seksuele-grensoverschrijding
- www.seksueelgeweld.info Website for victims of sexual violence, and for those involved and referrers. See also the social map with an overview of the available help for victims and perpetrators of sexual violence.
- www.vlaggensysteem.nl
- www.act4respect.nl
- www.atria.nl

SEXUAL VIOLENCE AGAINST ADULTS BY STRANGERS

ALWAYS USE THE REPORTING CODE WHEN YOU ENCOUNTER A FORM OF (DOMESTIC) VIOLENCE, ABUSE, NEGLECT OR EXPLOITATION! ▶

This fact sheet is part of a series about *(domestic) violence, abuse, neglect, exploitation* and other types of harm that may be inflicted onto someone in a power-imbalanced relationship. Power-imbalanced relationships can exist with anyone, for example: an (ex-) partner, a child, a parent, a sibling, another family member, an informal or a professional carer, a friend, a flatmate or neighbour, a teacher, a colleague or supervisor, or just someone you know. These fact sheets describe different types of harm that can be inflicted in these relationships. They are meant as an add-on to the Dutch Reporting Code for these issues (English version here) and were developed for two reasons: 1) To provide professionals with an overview of all the types of harm that exist, to aid them in identifying both well-known and lesser-known types (see the Overview). 2) Signs/indicators may vary greatly by type of harm and certain types of harm require specific courses of action; the fact sheets help professionals with identifying the signs/indicators and risk factors of *each specific type* of harm and with acting appropriately when they do. Note: the general 5 steps in the Reporting Code are applicable to all types of harm in power-imbalanced relationships; the factsheets provide more guidance within these 5 steps – they are an add-on, not a replacement.

Below is a brief introduction to this topic, an overview of the signs/indicators and risk factors associated with this type of harm, and points of attention for when you encounter it.

WHAT IS SEXUAL VIOLENCE?

Sexual violence is defined by law as a form of assault and rape, i.e. penetration (rape) or other sexual acts (assault) in which violence is used, a threat of violence, or abuse of a vulnerable situation or condition that made it impossible for someone to refuse (e.g. substance use) (Van Beek & Van Berlo, 2015).*

In the Istanbul Convention the following definition is used: The vaginal, anal or oral penetration of another person's body with a body part or object without mutual consent. This means that the sexual act is performed without the consent of the other person.

WHY IS IT DIFFICULT TO RECOGNISE SIGNS OF SEXUAL VIOLENCE?

It is difficult to recognise signs of sexual violence because most signs are non-specific: signs such as unusual behaviour, but also behaviour that is too "quiet" or too "active", do not indicate one cause specifically. However, the combination of signs can reinforce a suspicion. Victims usually do not disclose sexual violence, not even to their GP.

RISK FACTORS: WHO IS EXTRA VULNERABLE?

Sexual violence (which in this fact sheet is about sexual violence by strangers, so such violence by intimate partners or other people known to the victim lies outside its scope) can affect anyone, but there are groups that run a greater risk. This applies to women and lesbians, homosexuals, bisexuals, transgender persons and intersex persons (LHBTI), but also to people with negative childhood experiences. Previous experiences with sexual violence are a risk factor: almost a quarter of men and almost half

FACTS AND FIGURES

Reporting and declaration

Research by the Centraal Bureau voor de Statistiek (CBS) shows that the vast majority of sexual offences are not reported to the police (9% in 2011). If the perpetrator is an unknown person, the victim waits on average 10 days to report; if the perpetrator is a known person, however, it takes an average of 8 months (National Rapporteur on Trafficking in Human Beings and Sexual Violence against Children, 2014).

Relationship with the perpetrator

About 30% of the perpetrators of sexual violence in women are their own partner or ex-partner, about 15% of the perpetrators are met during nightlife. Approximately 19% of the female victims experience sexual violence by unknown perpetrators. Men are mainly victims of sexual violence where a friend is the perpetrator, not necessarily a (former) intimate relationship (28%). Partners or ex-partners are the perpetrators of sexual violence against men in 18.5% of cases and 17.4% of the male victims are violated by unknown perpetrators (De Haas, 2012).

LGBTI's experience relatively high levels of sexual violence (Act4Respect). One in five gay and bisexual men have experienced a form of sexual violence

* This fact sheet follows the terminology of the Whitepaper on sexual harassment and sexual violence of Movisie and Rutgers (2015). This differs from the terminology used by Veilig Thuis.

This fact sheet was developed by Movisie in collaboration with various chain partners in the fight against sexual violence.

of women who have experienced sexual violence experience this again later in life (revictimisation). In addition, social norms (such as socially accepted gender-stereotypical views, broadly supported double standards, general negative views about women and girls, and traditional views on male and female roles) can increase the risk of sexual violence (Act4Respect). Finally, drug use is a risk factor: for 31% of women and 23% of men, alcohol or drugs were used by the victim and/or the perpetrator before or during the event of sexual violence (De Graaf & Wijsen, 2017).

ASSISTANCE

In the event of a recent assault or rape victims are advised to report to the Centrum Seksueel Geweld (CSG) | the Sexual Violence Centre via 0800-0188 (preferably within the first seven days after the assault occurred). The CSG offers medical, forensic and psychological support after sexual violence in 16 regions A team of doctors, police, and emergency workers collaborate in the CSGs. This makes it an integrated care system; the victim preferably only has to come to one location. The victim receives medical assistance, a forensic analysis can be performed, a report can be made to the police (only if the victim wants to do this) and, if needed, psychological assistance is offered after a period of watchful waiting (monitoring the recovery process). Because of this integrated approach, the victim does not have to recount the violence more often than necessary (Bicanic, Engelhard & Sijbrandij, 2014).

If the sexual violence did not occur *recently*, the victim can contact the police or Slachtofferhulp Nederland (the Dutch Support organization for victims of violence). The Verbreek de Stilte helpline is part of the Dutch Victim Support Service and can be reached by telephone or chat. Here, social care professionals offer victims a listening ear; they also look for suitable help. Victim Support also offers legal, practical and emotional support; the assistance is free and always nearby.

Victims can also contact Veilig Thuis.

at some point in their lives, as compared to 6% of heterosexual men. Lesbian women are also relatively often victims of sexual violence: 37% of lesbian women have experiences with a form of sexual violence (De Haas, 2014; Rutgers WPF, 2013).

OTHER FIGURES

- Almost three quarters (73%) of all Dutch women have been sexually intimidated at some point in their lives;
- One in ten women have been raped at some point in their lives (FRA, 2014);
- 11% of all Dutch women have experienced sexual violence by a (former) partner;
- 12% of all Dutch women have been a victim of sexual violence by someone other than their partner.

MORE INFORMATION

See the Sources.

Separate fact sheets have been developed on other sexual violence topics:

- violence in the (ex-)partner relationship (including sexual violence)
- sexual behaviour that crosses boundaries between young people
- boundary-crossing behaviour against people with a disability
- online sexual harassment

ADVICE/REPORTING

When the sexual violence occurred recently, contact:

- The Centrum Seksueel Geweld (CSG) | the Sexual Violence Centre is the expertise centre of the Netherlands for victims of sexual violence in the acute phase (<7 days). At CSG, a team of doctors, nurses, police, psychologists, social workers and sexologists work together to provide specialist care to victims of assault and rape. Call **0800-0188**.

When the sexual violence occurred less recently, contact:

- Slachtofferhulp Nederland: telephone **0900-0101**
- Veilig Thuis ("Veilig Thuis" means "Safe at Home" in Dutch, it is the organization in the Netherlands for advice on, referrals to and reporting of any type of (domestic) violence, abuse, neglect or exploitation, or other types of harm in power-imbalanced relationships). Telephone: **0800 20 00**, free of charge and always open (24 hours per day, 7 days a week). It is possible to call anonymously and/or to call for advice or information only, without reporting someone.

In case of acute danger call the emergency services at the phone number **112**.

DUTCH TRANSLATION

See here.

This fact sheet was developed by Movisie in collaboration with various chain partners in the fight against sexual violence.

SOURCES SEXUAL VIOLENCE AGAINST ADULTS BY STRANGERS

NOTE

For all forms of domestic violence and child abuse, the Dutch Reporting Code (English version here) for these issues **must** be applied in the Netherlands by all groups of professionals named in the Reporting Code law. Sexual violence against adults by strangers does not fall under the definition of domestic violence or child abuse in the Netherlands and therefore, it is not legally required to use the reporting code when you encounter it as a professional. However, the reporting code **may** be used. Because we feel it is a useful guideline for professionals for this type of harm as well, and because it is important that professionals (e.g. general practitioners) can identify this type of harm and take the right steps, this factsheet was compiled.

ORGANISATIONS INVOLVED

The following organisations were involved in making this fact sheet:

- Movisie. For questions and/or remarks about the fact sheets, please email the lead author: Wilma Schakenraad, w.schakenraad@movisie.nl
- Veilig Thuis
- Atria, kennisinstituut voor emancipatie en vrouwengeschiedenis

SOURCES

The following documents and other sources provide more information about the topic of this fact sheet:

Websites

- Kennisdossier seksuele grensoverschrijding van Rutgers: www.rutgers.nl/feiten-en-cijfers/kennisdossiers/kennisdossier-seksuele-grensoverschrijding
- www.seksueelgeweld.info . Website voor slachtoffers van seksueel geweld, en voor betrokkenen en verwijzers. Zie hierop ook de sociale kaart met hulpaanbod in Nederland voor slachtoffers en plegers van seksueel geweld.
- Dossier seksueel geweld op www.huiselijkgeweld.nl/dossiers/seksueel-geweld
- www.act4respect.nl (website wordt eind 2018 gelanceerd).

Publications

- Berlo van, W. & Beek I. van (2015). Whitepaper Seksuele grensoverschrijding en seksueel geweld. Feiten en cijfers Utrecht: Rutgers en Movisie.
- Bicanic, I., Jongh, A., de, Lagro-Janssen, T. & Leusink, P. (2016). Centrum seksueel geweld voor acute slachtoffers. Huisarts & Wetenschap. 59 (6), 265-267.
- Centraal Bureau voor de Statistiek (2012). Integrale Veiligheidsmonitor 2011. Landelijke rapportage. Den Haag: Centraal Bureau voor de Statistiek.
- European Union Agency for Fundamental Rights (FRA) (2014). Violence against women: an EU-wide survey. Luxemburg: publications Office of the European Union.
- Graaf, H. de , & Wijsen, C. (red.) (2017). Seksuele gezondheid in Nederland. Utrecht: Rutgers i.s.m. RIVM. Zie: www.rutgers.nl/sites/rutgersnl/files/PDF-Onderzoek/Seksuele_Gezondheid_in_NL_2017_23012018.pdf
- Haas, S. de (2012). Seksueel grensoverschrijdend gedrag onder jongeren en volwassenen in Nederland. In Tijdschrift voor Seksuologie, 36(2), 136-145.
- Haas, S. de (2014). Seksueel geweld en seksuele grensoverschrijding. In H. de Graaf, B. Bakker & C. Wijsen, Een wereld van verschil. Seksuele gezondheid van LHBT's in Nederland 2013. Utrecht: Rutgers WPF. Kühl, M., Schakenraad, W., & Beek, I. van (2017). Werken met volwassen slachtoffers van seksueel geweld. In: Höing, M., & Janssen, J., Boer, A., & Liebregts, M. (red.). Bespreekbaar maken van seksualiteit en intimiteit. Handboek voor professionals in zorg en welzijn. Bussum: Coutinho.
- Nationaal Rapporteur Mensenhandel en Seksueel Geweld tegen Kinderen (2014). Op goede grond. De aanpak van seksueel geweld tegen kinderen. Den Haag: Nationaal Rapporteur.
- Römkens, R., Jong, T. de en Harthoorn, H. (2014). Geweld tegen vrouwen. Europese onderzoeksgegevens in de Nederlandse context. Amsterdam: Atria.
- Rutgers WPF (2013). Wat maakt het verschil? Diversiteit in de seksuele gezondheid van LHBT's, een verkenning. Utrecht: Rutgers WPF

This fact sheet was developed by Movisie in collaboration with various chain partners in the fight against sexual violence.

RADICALISATION

This fact sheet is part of a series about *(domestic) violence, abuse, neglect, exploitation* and other types of harm that may be inflicted onto someone in a power-imbalanced relationship. Power-imbalanced relationships can exist with anyone, for example: an (ex-)partner, a child, a parent, a sibling, another family member, an informal or a professional carer, a friend, a flatmate or neighbour, a teacher, a colleague or supervisor, or just someone you know. These fact sheets describe different types of harm that can be inflicted in these relationships. They are meant as an add-on to the Dutch Reporting Code for these issues (English version here) and were developed for two reasons: 1) To provide professionals with an overview of all the types of harm that exist, to aid them in identifying both well-known and lesser-known types (see the Overview). 2) Signs/indicators may vary greatly by type of harm and certain types of harm require specific courses of action; the fact sheets help professionals with identifying the signs/indicators and risk factors of *each specific type* of harm and with acting appropriately when they do. Note: the general 5 steps in the Reporting Code are applicable to all types of harm in power-imbalanced relationships; the factsheets provide more guidance within these 5 steps – they are an add-on, not a replacement.

Below is a brief introduction to this topic, an overview of the signs/indicators and risk factors associated with this type of harm, and points of attention for when you encounter it.

ALWAYS USE THE REPORTING CODE WHEN YOU ENCOUNTER A FORM OF (DOMESTIC) VIOLENCE, ABUSE, NEGLECT OR EXPLOITATION! ▶

WHAT IS RADICALISATION?

Since the mid-20th century, radicalisation and extremism have been studied extensively in relation to various forms of violent extremism. A central insight that has emerged from this research is that radicalisation is a difficult phenomenon to describe in a definite fashion. Violent forms may arise from any political or religious ideology. Some organise themselves into terrorist or paramilitary groups, others into small activist groups, and yet others become dangerous loners. In addition, not every form of radicalisation expresses itself through violence. Civil rights activists such as Martin Luther King were also considered radical. Certain ecological or religious lifestyles can be seen as radical or polarising, but are not necessarily illegal. What is considered radical is therefore very subjective. This fact sheet is limited to radicalisation that develops towards a form of (violent) extremism. It presents a general discussion of four main forms of extremism: religious extremism, left-wing extremism, right-wing extremism, and environmental extremism. Some examples of contemporary definitions of radicalisation and extremism are:

> "Radicalisation is an erratic process. A young person who is taken by radical ideas drifts away from democracy and grows towards violent extremism. A young person is inspired and starts to believe in an extremist narrative. He is going to take on more personal consequences and glorifies violence in order to achieve his ideals. Radicalisation is one way to become unhinged." (Stichting School & Veiligheid)

FACTS AND FIGURES

There are no direct figures on the number of radicalised persons within Dutch society, but there are figures on radicalised persons who have committed (violent) extremist crimes. There are, for example, several hundreds of people from the Netherlands, especially young people, who have travelled or tried to travel to conflict zones.[13] There are also multiple convictions for extremist activism such as threats, vandalism and arson. The percentage of people who commit criminal offences because of extremism is not high, but often these people come from extremist networks consisting of people who have not (yet) started (violent) extremist activities themselves. The number of people who have been radicalised in a worrying manner is therefore much higher than the number of people who have committed a known criminal offence.

MORE INFORMATION

See the Sources. In addition:

- PlatformJEP provides answers to the most important questions and provides an overview of different sources of information: www.platformjep.nl

This fact sheet was developed by Landelijk Steunpunt Extremisme (LSE) in collaboration with various chain partners.

RADICALISATION

"Extremism is the phenomenon wherein individuals or groups willingly cross the limits of the law in pursuit of their ideals. Extremism is different from activism. Activists can be vocal but without applying violence and abide by the limits of the law. Activism becomes extremism when criminal offences, such as violent threats and destruction of property, are deliberately committed." (NCTV)

There are several descriptions of radicalisation processes; some focus on psychological factors and others on sociological or economic factors. Because radicalisation is a capricious and dynamic phenomenon, especially among young people, these descriptions cannot be applied to all situations. Therefore, there are no checklists one can use to identify radicalisation.

POSSIBLE SIGNS/INDICATORS: HOW TO IDENTIFY IT

Because there is no checklist for radicalisation, it is difficult to indicate which signs indicate worrying behaviour. We often see various factors can influence the radicalisation process and the process not following a fixed pattern. There are all kinds of signs that in themselves are not indicative of a problem (so-called 'weak signs'), but that taken together can indicate that a situation is worrying:

- The person isolates himself from family and old friends, and socialises with a new network.
- The person visits worrying social media groups, websites or meetings.
- The person is intensively engaged in a new ideological or religious identity.[12]
- The person applies us-against-them-language, lecturing others, especially on political topics.
- Excessive truancy or quitting study, work, sports, or other hobbies.
- There has recently been a major event in the person's life, ranging from a move or a change of school or loss of work to death.

RISK FACTORS: WHO IS EXTRA VULNERABLE?

Because of the various forms of radicalisation there is no fixed profile of persons who are more likely to radicalise, but the risk of someone radicalising is increased when psychopathology is involved (psychosocial problems, mild intellectual disability, behavioural problems) and unstable living conditions (disrupted family/relationship, work and housing problems, drinking and/or drug use). Additionally, radicalisation mainly occurs among young people. A possible factor is brain development with regard to rational decision making which develops more slowly during the adolescent phase than other brain regions. This makes it difficult for young people between the ages of 14 and 23 to make informed choices, to think in abstract terms, and to understand the consequences of their choices.

STEPS TO FOLLOW WHEN YOU ENCOUNTER SIGNS OF RADICALISATION

Stay in contact and do not push away the person for his behaviour and statements. This may strengthen the behaviour and cause a person to (further) isolate himself. Engaging with non-radical persons is an important protecting factor against (further) radicalisation, and the present non-radical network also allows other persons and professionals to come into contact with the person. If there is radicalisation, several factors and approaches will have to be considered in order to reach the person and apply protective factors. Additionally, there are conversational techniques and interventions that can be used.

ADVICE

Employees of the Landelijk Steunpunt Extremisme (LSE) (the Dutch National Extremism Support Centre) are available to talk to you and discuss situations involving radicalisation. They provide advice on what you can do yourself and are able to indicate where professional help and possible reporting to the authorities are needed. If the situation does indeed turn out to be worrying, the case managers can help you. They are specialised in supporting and assisting people, families and professionals who are dealing with radicalisation. Web address: www.landelijksteunpuntextremisme.nl
Phone number: **088 - 20 80 080**.

DUTCH TRANSLATION

See here.

DEVELOPED BY

This fact sheet was developed by the Landelijk Steunpunt Extremisme (LSE) | National Support Centre for Extremism (LSE) in partnership with other organisations (see Organisations involved).

DOWNLOADS

This fact sheet can be accessed for free online and downloaded as a pdf, both in Dutch and in English.

This fact sheet was developed by Landelijk Steunpunt Extremisme (LSE) in collaboration with various chain partners.

SOURCES RADICALISATION

ORGANISATIONS INVOLVED

The following organisations were involved in making this fact sheet:

- The Landelijk Steunpunt Extremisme (LSE) | National Support Centre for Extremism (LSE), also a member of the JeP Platform, was in charge of developing this fact sheet. For questions and/or remarks about the fact sheet, please email: info@hetlse.nl
- Stichting School en Veiligheid
- Veilig Thuis

SOURCES

The following documents and other sources provide more information about the topic of this fact sheet:

- An ideology is a package of ideas on worldviews and existential meaning which focuses on perceptions on being human and the organization of society, such as fascism, anarchism, nationalism, (neo)liberalism, socialism, Islamism, Christian democracy, and conservatism. An ideology is therefore always politically oriented, but is not always an antiliberal or violent movement.
- "Terrorism is the ideologically motivated act of violence against humans or social disruptive damage to public property with the aim of undermining and destabilising society, seriously terrorizing the population or influencing political decision-making. CT Strategy, NCTV, www.nctv.nl/binaries/CT-strategie%202016-2020_tcm31-80007.pdf.
- For an overview see: PlatformJEP, www.platformjep.nl/documenten/vragen-en-antwoorden/wat-zijn-de-definities-van-radicalisering-extremisme-en-polarisatie. NCTV: www.nctv.nl/organisatie/ct/terrorismebestrijding/extremisme/extremisme.aspx.
- For examples of radicalisation processes, see: Understanding Radicalisation: Review of Literature, Dzhekova et al., Center for the Study of Democracy, 2016, www.csd.bg/artShow.php?id=17560 . Trigger factors in the radicalisation process, Feddes et al. Expertise unit Social Stability and University of Amsterdam, 2015, www.socialestabiliteit.nl/professionals/documenten/publicaties/2015/10/13/triggerfactoren-in-het-radicaliseringsproces . Genesis of radicalisation, Wienke and Ramadan, NJI, 2011, www.nji.nl/nl/Producten-en-diensten/Publicaties/NJi-Publicaties/Polarisatie-en-radicalisering-bij-jongeren.html .
- For an overview of signals and factors, see: Trigger factors Radicalisation, www.socialestabiliteit.nl/professionals/triggerfactoren . And Recognition and interpretation, Wienke and Ramadan, NJI, 2011, www.nji.nl/nl/Download-NJi/Publicatie-NJi/Pol_Rad_Herkenning_duiding.pdf.
- Conversion to a new religion or other ideological identity can be experienced as polarising, but is not in itself a sign of radicalisation.
- For a discussion on this, see: NJI, www.nji.nl/nl/Kennis/Dossier/Radicalisering/Achtergrond/Ontwikkeling , and, www.nji.nl/nl/Download-NJi/Publicatie-NJi/Pol_Rad_Ontstaan_radicalisering.pdf .
- For examples, see: NJI, www.nji.nl/nl/Kennis/Dossier/De-rol-van-jeugdhulp-bij-het-tegengaan-van-radicalisering-van-jongeren.
- These figures mainly concern foreign fighters who joined Jihadist groups in Syria and Iraq as of 2013. Of the several hundred people who travelled to Syria and Iraq, a few dozen have now returned. These returnees may include people who, through their experience, have become disillusioned with the extremist ideology and network, and others who are still active. This fact sheet focuses primarily on identifying radicalisation among persons who have not yet committed any criminal offences, and in which radicalisation is considered in its full breadth and not only from the perspective of religious extremism such as Jihadism.

This fact sheet was developed by Landelijk Steunpunt Extremisme (LSE) in collaboration with various chain partners. september 2020
