# Supplementary material for: Lesser-known types of violence: Helping nurses and midwives to signal and act
Source: Int J Nurs Stud Adv. 2022 Sep 17;4:100098. doi: 10.1016/j.ijnsa.2022.100098 (PMC11080451; doi:10.1016/j.ijnsa.2022.100098)
Supplement: Supplementary file 1 [file mmc1.zip › Overview of types of violence - Dutch - horizontal.pdf]

SPECIFIEKE **DOELGROEPEN** EN **VORMEN** VAN **HUISELIJK GEWELD** EN **KINDERMISHANDELING**

factsheets en websites voor professionals die werken met de meldcode

TEGEN PARTNER
- (EX-)PARTNERGEWELD

TEGEN KIND
- KINDERMISHANDELING
- GEWELD TEGEN HET ONGEBOREN KIND
- SHAKEN BABY SYNDROOM
- VROUWELIJKE GENITALE VERMINKING
- PEDIATRIC CONDITION FALSIFICATION
- JEUGDPROSTITUTIE MEISJES
- JEUGDPROSTITUTIE JONGENS
- DE KINDCHECK
- KINDEREN IN EEN CONFLICTSCHEIDING

TEGEN OUDEREN
- OUDEREN MISHANDELING

TEGEN EEN OUDER
- GEWELD TEGEN EEN OUDER DOOR EEN KIND

TEGEN...
- GEWELD TEGEN MANNEN
- GEWELD TEGEN KWETSBARE MIGRANTEN
- GEWELD TEGEN MENSEN MET EEN BEPERKING
- ONTSPOORDE MANTELZORG

>1 DOELGROEP
- MENSENHANDEL
- ONLINE SEKSUELE INTIMIDATIE
- STALKING
- EERGERELATEERD GEWELD
- VERBORGEN VROUWEN
- ACHTERLATING
- HUWELIJKSDWANG

PLEGERS
- PLEGERS

OVERIG
overig huiselijk geweld, zoals tegen een broer of zus, of een volwassen huisgenoot of een bekende

Ook bij andere typen geweld is het nuttig kennis te hebben van signalen en wat te doen bij signalen.
- pesten
- zelfbeschadiging
- seksueel grensoverschreidend gedrag door kinderen/jongeren
- seksueel geweld door onbekenden
- radicalisering

GEBRUIK BIJ ELKE VORM VAN HUISELIJK GEWELD EN KINDER-MISHANDELING DE MELDCODE!

SOORTEN GEWELD
vaak spelen er meerdere soorten geweld bij huiselijk geweld of kindermishandeling
- LICHAMELIJK
- EMOTIONEEL
- SEKSUEEL
- VERWAARLOZING
- FINANCIEEL
- GETUIGE VAN
