# Supplementary material for: Lesser-known types of violence: Helping nurses and midwives to signal and act
Source: Int J Nurs Stud Adv. 2022 Sep 17;4:100098. doi: 10.1016/j.ijnsa.2022.100098 (PMC11080451; doi:10.1016/j.ijnsa.2022.100098)
Supplement: Supplementary file 1 [file mmc1.zip › Overview of types of violence - Dutch - vertical.pdf]

SPECIFIEKE **DOELGROEPEN** EN **VORMEN** VAN **HUISELIJK GEWELD** EN **KINDERMISHANDELING**

factsheets en websites voor professionals die werken met de meldcode

TEGEN KIND

- KINDERMISHANDELING
- GEWELD TEGEN HET ONGEBOREN KIND
- SHAKEN BABY SYNDROOM
- VROUWELIJKE GENITALE VERMINKING
- PEDIATRIC CONDITION FALSIFICATION
- JEUGDPROSTITUTIE MEISJES
- JEUGDPROSTITUTIE JONGENS
- DE KINDCHECK
- KINDEREN IN EEN CONFLICTSCHEIDING

TEGEN PARTNER

- (EX-)PARTNERGEWELD

TEGEN OUDEREN

- OUDEREN MISHANDELING

TEGEN EEN OUDER

- GEWELD TEGEN EEN OUDER DOOR EEN KIND

TEGEN...

- GEWELD TEGEN MANNEN
- GEWELD TEGEN KWETSBARE MIGRANTEN
- GEWELD TEGEN MENSEN MET EEN BEPERKING
- ONTSPOORDE MANTELZORG

>1 DOELGROEP

- MENSENHANDEL
- ONLINE SEKSUELE INTIMIDATIE
- STALKING
- EERGERELATEERD GEWELD
- VERBORGEN VROUWEN
- ACHTERLATING
- HUWELIJKSDWANG

GEBRUIK BIJ ELKE VORM VAN HUISELIJK GEWELD EN KINDER-MISHANDELING DE MELDCODE!

OVERIG

overig huiselijk geweld, zoals tegen een broer of zus, of een volwassen huisgenoot of een bekende

PLEGERS

- PLEGERS

SOORTEN GEWELD

vaak spelen er meerdere soorten geweld bij huiselijk geweld of kindermishandeling

- LICHAMELIJK
- EMOTIONEEL
- SEKSUEEL
- VERWAARLOZING
- FINANCIEEL
- GETUIGE VAN

Ook bij andere typen geweld is het nuttig kennis te hebben van signalen en wat te doen bij signalen.

- pesten
- zelfbeschadiging
- seksueel grensoverschreidend gedrag door kinderen/jongeren
- seksueel geweld door onbekenden
- radicalisering
