# Supplementary material for: Lesser-known types of violence: Helping nurses and midwives to signal and act
Source: Int J Nurs Stud Adv. 2022 Sep 17;4:100098. doi: 10.1016/j.ijnsa.2022.100098 (PMC11080451; doi:10.1016/j.ijnsa.2022.100098)
Supplement: Supplementary file 1 [file mmc1.zip › Overview of types of violence - English.pdf]

SPECIFIC TYPES AND TARGET POPULATIONS OF (DOMESTIC) VIOLENCE, ABUSE, NEGLECT, EXPLOITATION

in power-imbalanced relationships

Fact sheets and websites for professionals who work with the Dutch Reporting Code

AGAINST PARTNER

- (EX-)PARTNER VIOLENCE

AGAINST CHILD

- CHILD ABUSE
- PROTECTION OF THE UNBORN CHILD
- SHAKEN BABY SYNDROOM
- FEMALE GENITAL MUTILATION
- PEDIATRIC CONDITION FALSIFICATION
- GIRLS <18 YEARS WHO SELL SEX
- BOYS <18 YEARS WHO SELL SEX
- THE CHILD CHECK
- CHILDREN IN A HIGH-CONFLICT SEPARATION

AGAINST ELDERLY

- ELDER ABUSE

AGAINST PARENT

- PARENT ABUSE

AGAINST ...

- MALE ABUSE
- VIOLENCE AGAINST/ AMONG MIGRANTS
- VIOLENCE AGAINST PEOPLE WITH INTELLECTUAL DISABILITIES
- ABUSE BY INFORMAL CARERS

>1 TARGET POPULATION

- HUMAN TRAFFICKING
- ONLINE SEXUAL INTIMIDATION
- STALKING
- HONOUR-BASED VIOLENCE
- HIDDEN WOMEN
- ABANDONMENT
- FORCED MARRIAGE

PERPETRATORS

- PERPETRATORS

OTHER

Other types of violence in power-imbalanced relation-ships, such as against a brother or sister, an adult housemate or an acquaintance

This overview is intended to help professionals respond to violence in power-imbalanced relationships. There are also other types of violence, for which it is also useful to have knowledge of signs and what to do when you see signs. Examples are:

- Bullying
- Self-harm
- Sexual behaviour between young people that crosses boundaries
- Sexual violence against adults by strangers
- Radicalization

TYPES OF VIOLENCE

With the types of (domestic) violence, abuse, neglect and exploitation listed here above, there are six broader types of violence that often play a role

- PHYSICAL
- EMOTIONAL
- SEXUAL
- NEGLECT
- FINANCIAL
- BEING A WITNESS

ALWAYS USE THE REPORTING CODE WHEN YOU ENCOUNTER A FORM OF (DOMESTIC) VIOLENCE, ABUSE, NEGLECT OR EXPLOITATION!
